# Importance of base-pair opening for mismatch recognition

**DOI:** 10.1093/nar/gkaa896

**Published:** 2020-10-20

**Authors:** Tomáš Bouchal, Ivo Durník, Viktor Illík, Kamila Réblová, Petr Kulhánek

**Affiliations:** CEITEC - Central European Institute of Technology, Masaryk University, Kamenice 5, 625 00 Brno, Czech Republic; National Centre for Biomolecular Research, Faculty of Science, Masaryk University, Kamenice 5, 625 00 Brno, Czech Republic; CEITEC - Central European Institute of Technology, Masaryk University, Kamenice 5, 625 00 Brno, Czech Republic; National Centre for Biomolecular Research, Faculty of Science, Masaryk University, Kamenice 5, 625 00 Brno, Czech Republic; National Centre for Biomolecular Research, Faculty of Science, Masaryk University, Kamenice 5, 625 00 Brno, Czech Republic; CEITEC - Central European Institute of Technology, Masaryk University, Kamenice 5, 625 00 Brno, Czech Republic; CEITEC - Central European Institute of Technology, Masaryk University, Kamenice 5, 625 00 Brno, Czech Republic; National Centre for Biomolecular Research, Faculty of Science, Masaryk University, Kamenice 5, 625 00 Brno, Czech Republic

## Abstract

Mismatch repair is a highly conserved cellular pathway responsible for repairing mismatched dsDNA. Errors are detected by the MutS enzyme, which most likely senses altered mechanical property of damaged dsDNA rather than a specific molecular pattern. While the curved shape of dsDNA in crystallographic MutS/DNA structures suggests the role of DNA bending, the theoretical support is not fully convincing. Here, we present a computational study focused on a base-pair opening into the minor groove, a specific base-pair motion observed upon interaction with MutS. Propensities for the opening were evaluated in terms of two base-pair parameters: Opening and Shear. We tested all possible base pairs in anti/anti, anti/syn and syn/anti orientations and found clear discrimination between mismatches and canonical base-pairs only for the opening into the minor groove. Besides, the discrimination gap was also confirmed in hotspot and coldspot sequences, indicating that the opening could play a more significant role in the mismatch recognition than previously recognized. Our findings can be helpful for a better understanding of sequence-dependent mutability. Further, detailed structural characterization of mismatches can serve for designing anti-cancer drugs targeting mismatched base pairs.

## INTRODUCTION

Watson–Crick (canonical) nucleobase pairs are essential for the integrity of double-stranded DNA (dsDNA) and fidelity of genetic information. The appearance of mismatched base pairs (mismatches) can result in the development of inherited genetic diseases, cancer and aging (1,2). Therefore, genetic information is continuously maintained by repair pathways that scan dsDNA and resolve inappropriate base pairing. Proteins responsible for mismatch recognition are very effective despite structural similarities between some mismatches and canonical base pairs. Understanding how such delicate discrimination is achieved on the molecular level can be beneficial for biomedical applications providing early diagnosis of inherited diseases (3,4). Further, the molecular origin of the recognition can be helpful in the development of more efficient anti-cancer drugs (5–8) targeting damaged DNA.

The mismatch repair pathway (MMR) is specialized in the repair of mismatches and short insertion/deletion loops. Experimental studies revealed that a mismatch is recognized by MutS protein (9–16). MutS recognizes multiple types of mismatches, while recognition proteins from the base excision repair (BER) (17,18) pathway are specialized on a single type of base lesion, and nucleotide excision repair (NER) (19) pathway targets bulky damages. The versatility of MutS and its ability to detect small structural changes suggests that the recognition is based on sensing an altered property of dsDNA instead of a specific molecular pattern (20). Moreover, the change must be significant enough to discriminate mismatches and canonical base pairs unambiguously. MMR is highly conserved both in prokaryotes and eukaryotes (21,22) showing its paramount importance for the fidelity of DNA replication. Dysfunction of MMR has thus severe impacts on the stability of genetic information and is often connected with cancer, e.g. Hereditary Non-Polyposis Colorectal Cancer (23).

**Figure 1.**
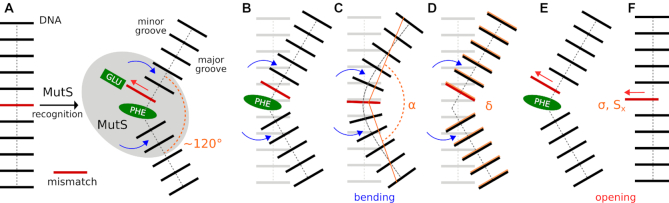
(**A**) Schematic representation of mismatch recognition by MutS. A mismatch is red, and conserved amino acids from the GLU-X-PHE motif are green. Upon mismatch recognition, two main motions within DNA can be detected in experimental structures of MutS/DNA complexes: sharp DNA bending at a site of mismatch (blue arrows) and mismatch opening into the minor groove (red arrow). Models describing the DNA bending in the absence of MutS are: (**B**) DNA bending in the presence of PHE intercalator; (**C**) DNA bending induced by a simple bending angle α (28), which results in a smooth DNA bending; (**D**) DNA bending induced by a root-mean-square distance δ (3) from the DNA in the MutS/DNA complex (orange), which recovers correct DNA shape. Models C and D are deficient due to the absence of the PHE intercalator. Models describing the base-pair opening in the absence of MutS are: (**E**) opening in DNA bent due to the presence of the PHE intercalator, and (**F**) opening in a relaxed DNA facilitated by *simple* base-pair parameters Opening σ and Shear S_x_ (this study). Despite the simplicity of the model F, the stacking from the intercalator is effectively substituted by the adjacent canonical base pair. Moreover, the locality of Opening and Shear allows a direct transfer of results from the model F (straight DNA) to the model E (bent DNA).

In the MutS/DNA complex, DNA is sharply bent at the site of the mismatch. Moreover, the corrupted site interacts with two conserved amino acid residues from the PHE-X-GLU motif. In the case of human MutSα, these residues are PHE432 and GLU434 (9). While phenylalanine intercalates into the mismatched base step, glutamate forms a specific contact with a base of the mismatch. As a result, the mismatched pair is opened into the minor groove of DNA (Figure 1A) (10).

Since MutS significantly bends DNA, it was hypothesized that MutS tests the flexibility of DNA altered by the presence of a mismatch (24,25). The flexibility of DNA upon bending has been studied by several theoretical approaches from pioneering works of Curuksu *et al.* (26,27) to Sharma *et al.* (28), who evaluated DNA bending propensities in the presence of a mismatch. While they showed that DNA with a mismatch could be bent more easily than DNA containing canonical G:C or A:T pairs, discrimination between the G/T mismatch and canonical base pairs was rather small (28). Ruzicka *et al.* studied DNA bending in various sequence contexts. Despite the limitation of the employed model, their results revealed the relation between the mutability of DNA sequences and their bending properties. Mainly, coldspots were found to be more flexible than hotspots suggesting that altered flexibility can influence the effectivity of MMR (3,4).

The two studies differ mainly in the way how DNA was bent. Sharma's approach employing a simple bending angle (α) resulted in relatively smooth DNA bending, which is not observed in MutS/DNA complexes (Figure 1C). On the contrary, Ruzicka achieved more natural bending employing a root-mean-square distance (δ) to the DNA geometry observed experimentally in the MutS/DNA complex (Figure 1D). Other computational studies were focused on nucleobase flipping either in the various sequence context or in the presence of a mismatch (29–31). However, due to the extent of base-pair disruption, these studies are probably more relevant for BER than MMR.

Despite a diverse range of phenomena investigated, a common feature of these computational studies is the usage of simple geometry parameters. Usually, these are combinations of distances, angles, or dihedral angles, which attempt to describe very complex geometrical changes. Also, these geometry parameters are often non-local. For example, the α and δ parameters depend on the geometry of the entire DNA, while they try to rationalize DNA flexibility occurring on a site with the mismatch.

Since previous computational studies have not fully supported DNA bending as a key element of the mismatch recognition, in this work, we focused on the base-pair opening. We calculated thermodynamic stabilities of canonical as well as mismatched base pairs in a short dsDNA as a function of two geometry parameters employing atomistic molecular dynamics (MD) simulations. Based on a detailed analysis of experimental structures of MutS/DNA complexes, we selected two *simple* base-pair parameters: Opening and Shear (32,33). Opening is directly related to the studied base-pair motion, while Shear had to be included to facilitate the unspecific reorganization of hydrogen bonds observed in several mismatches. Moreover, the geometry definition of both parameters was strictly local, which provided better resolution of thermodynamic states, more straightforward interpretation, and transferability of the results to other environments such as bent DNA in the MutS/DNA complexes.

## MATERIALS AND METHODS

### Studied system

The opening of the central base pair and bending of DNA were evaluated in models containing a short dsDNA without MutS. We considered 8 different sequence classes differing in the composition of the central base pair X/Y (Table 1). Each system was built *in silico* as a standard B-DNA by Nucleic Acid Builder (NAB) (34). For sequence class **I**, we considered 16 possible combinations of nucleotides A, G, T and C at the X and Y positions. Also, we considered *anti* (≈−120°) and *syn* (≈70°) orientations at N-glycosidic bonds (χ torsion angle, Supplementary Figure SA1) and their *anti*/*anti*, *anti*/*syn* and *syn*/*anti* combinations. Since the class **I** has a palindromic sequence, only 26 unique systems were modeled out of 48 possible (Equivalences are shown in Supplementary Figure SA2). In the following text, the systems will be annotated in the form of (a/s)X/(a/s)Y (mismatches) or aX:aY (canonical pairs), where ‘a’ stands for *anti* and ‘s’ for *syn* orientation.

**Table 1:** Summary of 47 systems of dsDNAs containing different X/Y base pairs in the central part grouped into 8 sequence classes.

Class	Chain A / Chain B	Systems (X/Y)	Comment (Central 5-nt sequence)
**I**	5′-GGTTAA**X**TTAACC-3′	anti/anti: aA/aA, aA/aC, aA/aG, aA:aT, aC/aC; aC/aT, aG:aC, aG/aG, aG/aT, aT/aT	Palindromic sequence, used in ref. (35)
	3′-CCAATT**Y**AATTGG-5′	anti/syn: aA/sA, aA/sC, aA/sG, aA/sT, aC/sC, aC/sT, aG/sC, aG/sG, aG/sT, aT/sT	(AA**X**TT)
		syn/anti: sA/aC, sA/aG, sA/aT, sC/aT, sG/aC, sG/aT	
**II**	5′-GAACCGC**X**CGCTAGG-3′	aA:aT, aG:aC, aG/aT	Derived from MutS/DNA X-ray structure (9)
	3′-CTTGGCG**Y**GCGATCC-5′		(GC**X**CG)
**III**	5′-GAACCAA**X**TTCTAGG-3′	aA:aT, aG:aC, aG/aT	Derived from class **II** as
	3′-CTTGGTT**Y**AAGATCC-5′		(AA**X**TT)
**C1**	5′-GAACCAA**X**AACTAGG-3′	aA:aT, aG:aC, aG/aT	Derived from class **II** as coldspot (3)
	3′-CTTGGTT**Y**TTGATCC-5′		(AA**X**AA) P-value:^a^ 1.3e-6
**C2**	5′-GAACCCA**X**TGCTAGG-3′	aA:aT, aG:aC, aG/aT	Derived from class **II** as coldspot (3)
	3′-CTTGGGT**Y**ACGATCC-5′		(CA**X**TG) P-value:^a^ 9.2e-5
**H1**	5′-GAACCAG**X**TACTAGG-3′	aA:aT, aG:aC, aG/aT	Derived from class **II** as hotspot (3)
	3′-CTTGGTC**Y**ATGATCC-5′		(AG**X**TA) P-value:^a^ 1.3e-11
**H2**	5′-GAACCTC**X**CACTAGG-3′	aA:aT, aG:aC, aG/aT	Derived from class **II** as hotspot (3)
	3′-CTTGGAG**Y**GTGATCC-5′		(TC**X**CA) P-value:^a^ 2.7e-8
**H3**	5′-GAACCTG**X**AACTAGG-3′	aA:aT, aG:aC, aG/aT	Derived from class **II** as hotspot (3)
	3′-CTTGGAC**Y**TTGATCC-5′		(TG**X**AA) P-value:^a^ 6.6e-4

^a^Fisher combined P-values classifying the 5-nt long segment as a hotspot or coldspot sequence taken from the 2016 dataset of HGMD database for X as guanine (3).

The sequence class **II** was adopted from DNA taken from the experimental X-ray structure of the MutS/DNA complex (9). The sequence class **III** was derived from the sequence class **II** by changing its central 5-nt long segment to be the same as in the class **I**. The **C1**-**C2** and **H1**-**H3** classes were derived from sequences from our previous classification of DNA motifs for coldspots and hotspots (3). In particular, **C1** and **C2** represent sequences rarely associated with germinal mutations, while **H1**–**H3** are frequently associated with germinal mutations. In this classification, we considered germinal mutations in the HGMD database. For more details, see our previous study (3).

Molecular dynamics simulations were performed in the Amber 16 package (36,37). DNA can be described by several force fields such as parmbsc1 (38), parmOL15 (39), CHARMM27 (40,41), and CHARMM36 (42). Since recent benchmarks showed similar performance of parmbsc1 and parmOL15 (43–45) and overall better reliability than CHARMM force fields (44,46,47), we employed parmbsc1 in this work. Each DNA was immersed into a truncated octahedral box filled by the TIP3P water, (48) and 24 (class **I**) or 28 (classes **II, III, C1, C2, H1, H2, H3**) sodium (49) cations to maintain electroneutrality. The concentration of Na^+^ was approximately 0.18 M. Except for net charge neutralization, we also tested physiological salt concentration (c_0_(NaCl) = 0.15 M), but we found no significant impact on the obtained results (Supplementary Figure SA16). The radius of the largest inscribed sphere into the box was about 27 Å, which ensured enough space for all deformations of DNA tested in this work. Temperature and pressure were kept at 300 K and 100 kPa, respectively.

### Biased molecular dynamics simulations

Free energy calculations were performed in the modified pmemd program from AMBER connected with PMFLib (50) (https://pmflib.ncbr.muni.cz). The free energy ΔG_r_ as a function of collective variables was calculated by the Adaptive Biasing Force (ABF) method (51,52) enhanced by Multiple-Walker Approach (MWA) (53,54). The subscript ‘r’ indicates the relative free energy, which is referenced to the most stable thermodynamic state of each simulated system. ABF/MWA simulations with one and two collective variables were 200 ns and 1 μs long each (if not stated otherwise), respectively (Supplementary Tables SA5–SA7). Our tests (Supplementary Figure SA9) confirmed that this sampling time is long enough to obtain converged free energies. Due to the numerical complexity of some CVs and PMFLib design, all ABF/MWA simulations were run on CPUs with >47 μs of sampling acquired in total. The calculated mean forces were integrated using Gaussian Process Regression (GPR) (55,56) to get the final free energy landscapes. The GPR integration was thoroughly tested (Supplementary Figures SA7–SA9, Supplementary Tables SA8–SA13) with GPR hyperparameters calculated by maximizing the logarithm of the marginal likelihood (57). Statistical inefficiency due to correlation in time series was evaluated by integrated autocorrelation time implemented in the pymbar package (58), the blocking method (59), unbiased estimation of the variance of the sample mean (60), and optimizing a GPR hyperparameter (Supplementary Figure SA10). The Gaussian process was also employed for evaluation of the standard errors of the free energies. In this work, the free energies are reported with confidence intervals provided at three standard deviations.

If necessary, the free energy surfaces Δ*G*_r_(X,Y) were reduced to the free energy profiles Δ*G*_w_(X), where the ‘w’ subscript indicates the statistical averaging. The statistical averaging employed the definite integral over the partition function (61) evaluated numerically at a temperature of 300 K, including propagation of uncertainties.

**Figure 2.**
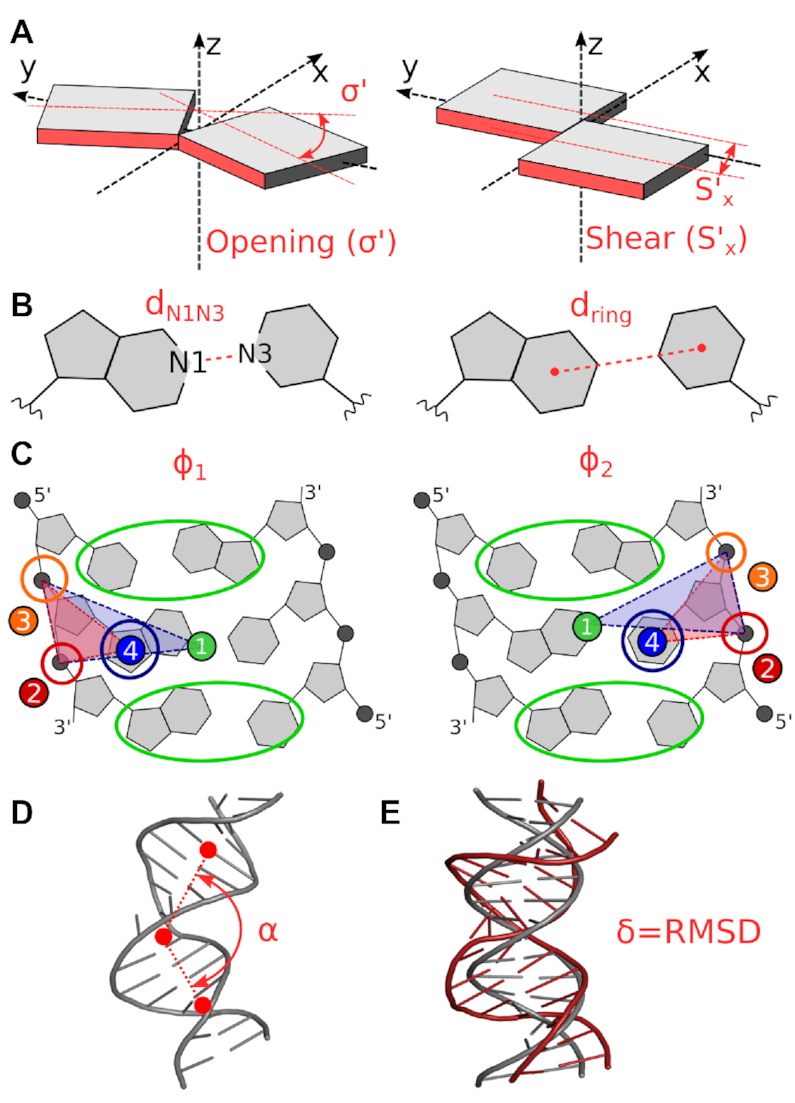
Schematic representation of collective variables describing the base-pair opening and DNA bending. (**A**) The transformed version of *simple* base-pair parameters: Opening σ′ and Shear *S*′_x_; (**B**) distances d_N1N3_ and d_ring_, (**C**) pseudo-dihedral angles ϕ_1_ and ϕ_2_ calculated from centers of masses (COMs) of encircled atom groups; (**D**) bending angle α calculated from COMs of three DNA segments and (**E**) root-mean-square distance δ of DNA (gray) to the experimental structure of MutS/DNA complex (red).

Collective variables (CVs) are geometry parameters, which were actively biased during ABF/MWA simulations. In this work, we employed a wide range of collective variables (Figure 2). The geometry of the central base pair X/Y was described by two *simple* base-pair parameters Opening σ′ and Shear *S*′_x_. They are identical to the definition of *simple* base-pair parameters with the same name as employed by 3DNA (32,33), except they were transformed to keep their meaning for other than *anti*/*anti* orientations (Supplementary Figures SA3 and SA4, Supplementary Table SA1). Other CVs describing base-pair geometry were distances *d*_N1N3_ and *d*_ring_ (31) and pseudo-dihedral angles ϕ_1_ and ϕ_2_ (30). The bending angle α was calculated as the angle between arms and the central part of DNA (28). In contrary to the original work, this CV was adapted to the shorter length of dsDNA (class **I**) using centers of masses of residues 1–4, 23–26; 5–9, 18–22 and 10–17 excluding hydrogen atoms. The bending was also described by the root-mean-square distance from the target structure (δ) (3). The target dsDNA structure was taken from the experimental MutS/DNA complex (PDB ID: 2O8B (9)). Due to different sequences than in the original work (3), only atoms from the sugar-phosphate backbone, excluding hydrogen atoms, were targeted (the atom set C from the original work).

### Analysis

The trajectories were processed and analyzed using the cpptraj module (62) of AMBER, the CATs package (63) (https://cats.ncbr.muni.cz) and 3DNA. Hydrogen bonds were analyzed by cpptraj with the default criteria. Base-pair parameters and base-pair step parameters were obtained by 3DNA. The CATs package connected with PMFLib served for the filtering of the ABF/MWA trajectories by values of collective variables and calculated free energies. Thermodynamic states corresponding to local minima found on the free energy surfaces were represented by ensembles of structures sampled in the ABF/MWA trajectories that are ≤ 0.3 kcal mol^−1^ (0.5RT at 300 K) above the free energy minima (Supplementary Figure SA11). Representative structures for each thermodynamic state were calculated as average structures from these ensembles, and then partially optimized to fix the position of hydrogen atoms. Obtained structures were visualized using PyMol (64).

Supplementary data – Part A contains a detailed setup of unbiased molecular dynamics simulations, ABF/MWA simulations, employed collective variables, and analysis.

## RESULTS AND DISCUSSIONS

### Base-pair opening in the absence of MutS

The geometry of a base pair is best described by six base-pair parameters (Shear, Stretch, Stagger, Buckle, Propeller and Opening – Supplementary Figure SA3) (32,65,66). In this work, we used the *simple* base-pair parameters as introduced in the 3DNA package (67). The parameters were designed for the description of structural variations of non-canonical base pairs. We analyzed all available experimental structures of mismatched DNA bound and unbound to MutS. The comparison revealed that the most significant geometry change of the mismatched base pair during the mismatch recognition is described by Opening (Supplementary Tables SA2–SA4).

We attempted to quantify this movement on a model of DNA in the absence of MutS (Figure 1F). Although this model is simplified, it can be used to investigate intrinsic properties of DNA. Moreover, we simulated all possible mismatches and thus complemented missing information about their geometries as experimental MutS/DNA complexes exist only for the aG/aT, aA/sA, aG/sG and sA/aC mismatches.

The thermodynamic stabilities of base-pairs were quantified by the free energy change calculated as a function of Opening (σ′) and also Shear (*S*′_x_). The second collective variable, Shear, had to be included because it describes changes in hydrogen bonding for some mismatches. We found that these changes are rare events on timescales of MD simulations. A typical example is the aG/aT mismatch, where only a few reorganizations of hydrogen bonding occurred spontaneously during 5 μs long unbiased simulation (Supplementary Figure SA6). Besides, we had to employ different reference nucleobase frames for comparison of mismatches in other than *anti* orientation. The usage of non-standard reference frames (Supplementary Figure SA4) is indicated by an apostrophe in parameter symbols (σ′, *S*′_x_).

In total, we ran 26 biased MD simulations (class **I**, see Materials and Methods). We considered all combinations of *anti*/*syn* nucleobase orientations on *N*-glycosidic bonds except *syn*/*syn* because it has not been observed experimentally. On the calculated free energy surfaces (FES), we have identified and characterized 94 local minima (thermodynamic states) (Supplementary Tables SB1.1–SB26.1 and Supplementary Figures SB1.1–SB26.1), 37 for *anti*/*anti* (Supplementary Figure SB0a), 35 for *anti*/*syn* (Supplementary Figure SB0b), and 22 for *syn*/*anti* (Supplementary Figure SB0c) orientations. For each thermodynamic state, we calculated average hydrogen bonding (Supplementary Tables SB1.2–SB26.2), base-pair (Supplementary Tables SB1.3–SB26.3), and base-pair step parameters (Supplementary Tables SB1.4–SB26.4), together with the representative structures (Supplementary Figures SB1.2–SB26.2). The analysis revealed that the canonical base pairs, which neighbor with the mismatched base pair, kept canonical hydrogen bonding, except a few cases, in which the mismatch was significantly perturbed from the global minima.

**Figure 3.**
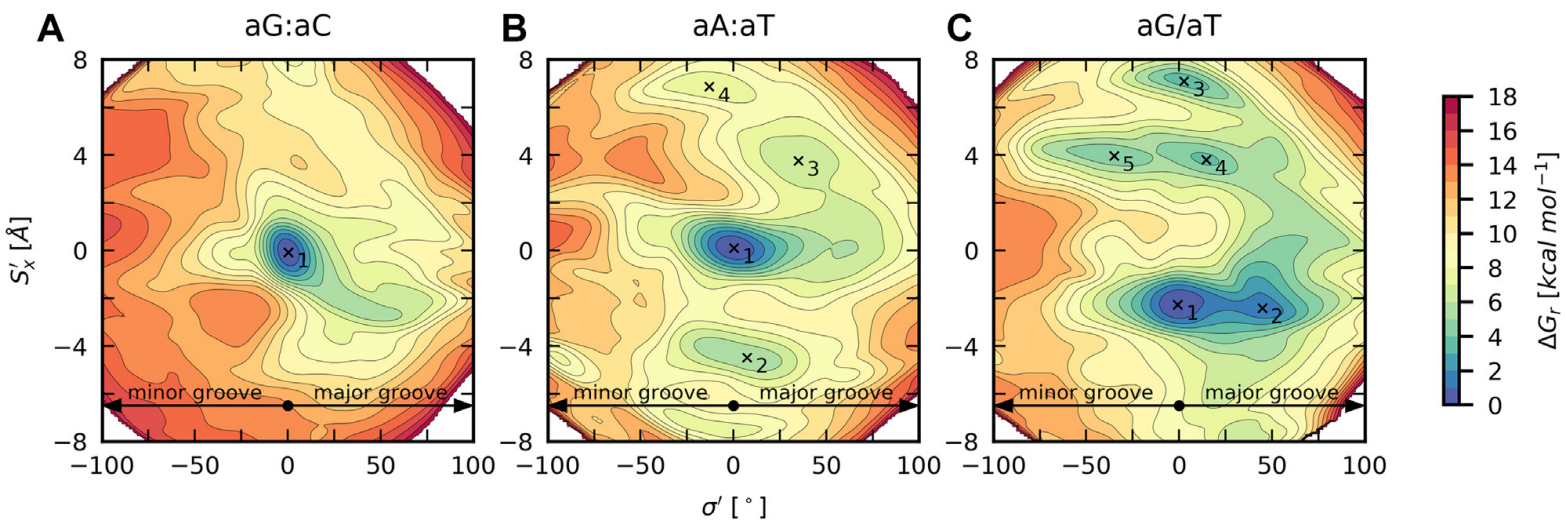
Relative free energy surfaces for (**A**) aG:aC, (**B**) aA:aT and (**C**) aG/aT (class **I**). Free energy minima are labeled in ascending order. Free energy isolines are spaced by 1 kcal mol^−1^.

**Figure 4.**
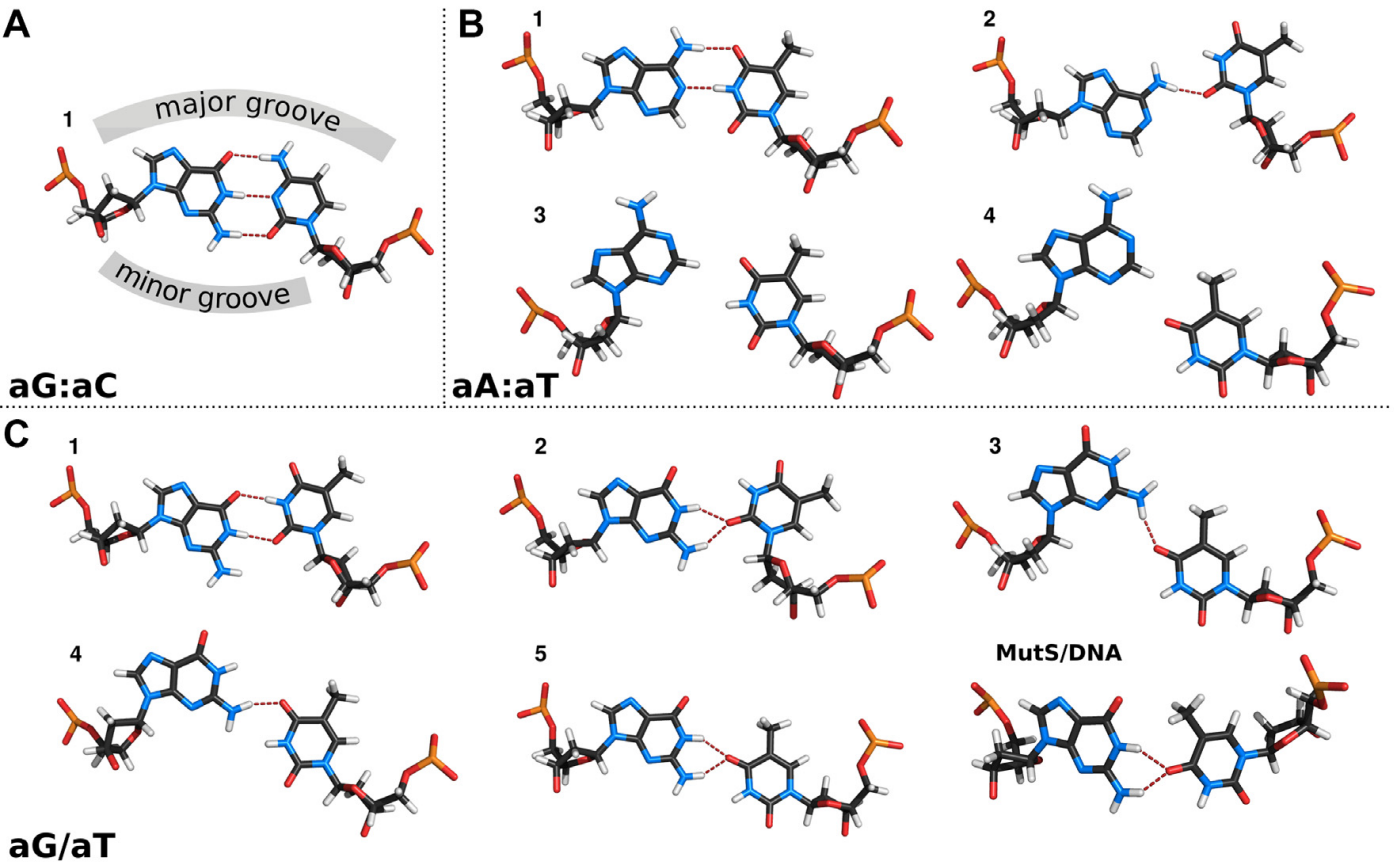
Representative geometries for (**A**) aG:aC, (**B**) aA:aT and (**C**) aG/aT. Structure labels coincide with the free energy minima shown in Figure 3. The structure of aG/aT from the MutS/DNA complex was taken from PBD ID: 2O8B (9). Hydrogen bonds are depicted with thin red lines. View direction is along the z-axis of DNA.

Here, we will demonstrate our results on the canonical base pairs aG:aC, aA:aT and mismatch aG/aT (Figure 3). The FES for aG:aC reveals the canonical structure as the most thermodynamically stable state (Figure 4A). Similar behavior is observed for aA:aT, but here we also found additional states in the higher free energy regions (Figure 4B). They result from improper hydrogen bonding (structure **2**) or weak steric clashes between two bases (structures **3** and **4**). On the contrary, the aG/aT mismatch exhibits a much softer free energy landscape with states stabilized by different hydrogen bonding (Figure 4C). Structures **1** and **2** are well-known from past studies (35), but structures **3–5** are new. They are more than 3.7 kcal mol^−1^ above the global minimum and are separated by a 5.4 kcal mol^−1^ barrier, which quantitatively confirms that the transitions in Shear are rare events. Among found structures, the most interesting is the structure **5**, which adopts similar Opening and Shear as the aG/aT mismatch in the MutS/DNA complex (Figure 4C) (9).

### Opening and Shear are irreplaceable by other geometrical parameters

The selection of proper collective variables (CVs) is all but the trivial task. Their choice ultimately determines which portion of the configurational space will be sampled, and the resolution of detected thermodynamic states. In this section, we will compare the performance of Shear and Opening to other CVs employed in the past (30,31,68). As a test case, we selected the aG/aT mismatch, which contains several thermodynamic states separated by barriers high enough to make some movements slow on the timescale of MD simulations. Such behavior poses many challenges for accurate description by collective variables.

**Figure 5.**
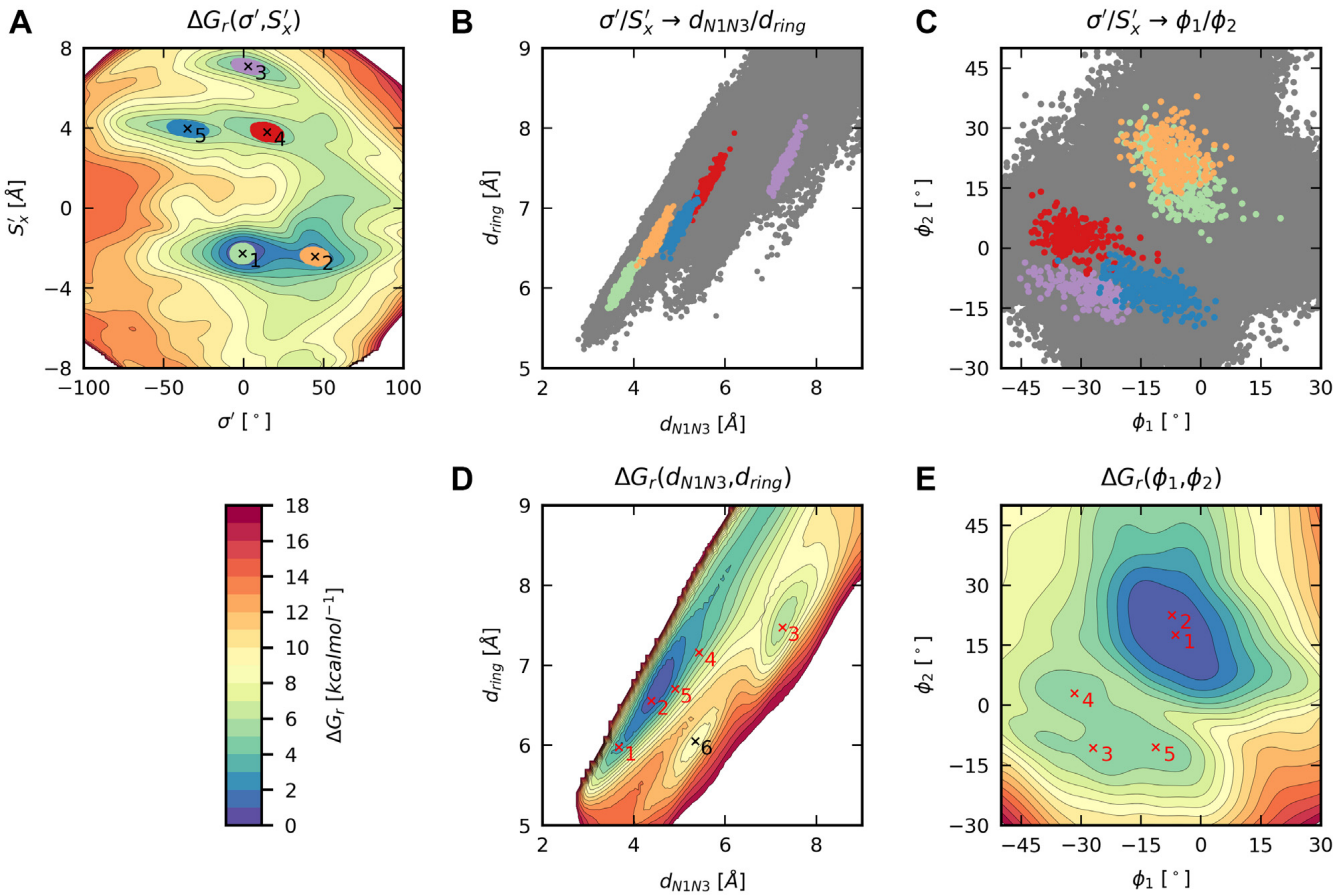
Various approaches for studying thermodynamic stability of the aG/aT mismatch (class **I**): (**A**) free energy surface Δ*G*_r_(σ′,*S*′_x_), colored ellipses represent structures in close vicinity of free energy minima (thermodynamic states) numbered from **1** to **5**; (**B**) structures **1**–**5** projected to *d*_N1N3_/*d*_ring_; (**C**) structures **1**–**5** projected to φ_1_/φ_2_; gray points are all structures from Δ*G*_r_(σ′,*S*′_x_); (**D**) free energy surface Δ*G*_r_(*d*_N1N3_,d_ring_); (**E**) free energy surface Δ*G*_r_(φ_1_,φ_2_); The expected positions of states **1**–**5** are shown in red, the additional minimum found on Δ*G*_r_(*d*_N1N3_,d_ring_) is shown in black. Free energy isolines are spaced by 1 kcal mol^−1^.

The first tested CVs were the distances *d*_N1N3_ and *d*_ring_ (Figure 2B) (31). These CVs are local, i.e. their values depend solely on the geometry of a base pair. First, we were interested if *d*_N1N3_/*d*_ring_ can distinguish states found on the free energy surface Δ*G*_r_(σ′,*S*′_x_). These states were represented by sets of structures extracted in close vicinity of five detected free energy minima (Figure 5A). The structures are well separated on Δ*G*(σ′,*S*′_x_), but they overlap when projected on *d*_N1N3_/*d*_ring_ (Figure 5B). Because of these overlaps, states **1**, **2**, **4** and **5** fell into one prolonged free energy minimum on the Δ*G*_r_(*d*_N1N3_,*d*_ring_) free energy surface (Figure 5D), while only the state **3** appeared as a separate free energy minimum. Further analysis showed that the inability for resolving states is caused by an ambiguous mapping of *S*′_x_ on *d*_N1N3_. Even though the inclusion of *d*_ring_ allowed to explore some higher energy stacked states (additional minimum **6**, Figure 5D), its combination with *d*_N1N3_ is insufficient for motions involving changes of Shear.

The second tested CVs were pseudo-dihedral angles φ_1_/φ_2_ (Figure 2C) (30). Opposed to *d*_N1N3_/d_ring_ and σ′/S′_x_, these CVs are non-local because the position of nucleobases is described relative to the rest of the DNA. Again, we observed several overlaps (Figure 5C) but only for states, which are close to each other on Δ*G*_r_(σ′,*S*′_x_). One covers states **1** and **2**, and the second contains states **3**, **4** and **5**. As a result, φ_1_/φ_2_ shows two broad free energy minima on the free energy surface (Figure 5E). The failure of φ_1_/φ_2_ can be rationalized by releasing tension to base-pair surroundings (pivot and anchor points, Figure 2C) when attempting to deform the base pair into high energy configurations.

The success of *d*_N1N3_/*d*_ring_ in the original work (31) was most likely caused by focusing on a different base pair (aA:aT), which exhibits less complicated free energy surface (deep free energy minimum) than aG/aT. While collective variables based on pseudo-dihedral angles as φ_1_/φ_2_ can be beneficial for studies of base flipping out of double helix as observed in BER (18), subtle changes significant for base-pair opening as seen in MutS/DNA complexes are not fully captured.

### Base-pair opening into the minor groove discriminates mismatches from canonical pairs

Because Opening is the specific motion observed in MutS/DNA complexes, we reduced Δ*G*_r_(σ′,*S*′_x_) to Δ*G*_w_(σ′) to get a more straightforward framework for the mismatch comparison. The reduction was achieved by statistical averaging for each family of conformations (*anti*/*anti*, *anti*/*syn*, *syn*/*anti)* (see Supplementary data – Part A for further details and Supplementary Figure SA13 for all data). Since MutS tests a base pair from the most stable structure, we used this structure as a reference for calculation of the relative Opening (Δσ′). Then, the propensity for the base-pair opening was quantified as Δ*G*_w_(Δσ′). Here, we would like to recall that the direct calculation of Δ*G*_w_(σ′) from biased MD simulations employing Opening as the only collective variable would be troublesome because changes in Shear are rare events on the timescale of MD simulations.

**Figure 6.**
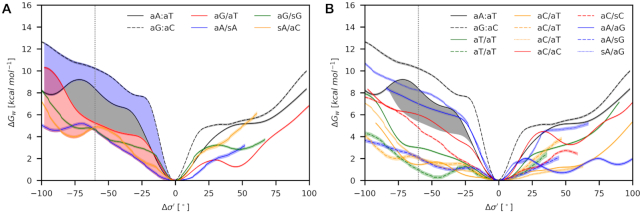
The propensity for the base-pair opening quantified as Δ*G*_w_(Δσ′) for the movement towards the minor (Δσ′ < 0) and major (Δσ′ > 0) grooves for (**A**) best-repaired mismatches and (**B**) less effectively repaired mismatches (class **I**). The vertical line at −60° corresponds to the average experimental value (see Figure 9, Supplementary Table SA3). A comparison between the canonical base pairs (blue zone) and best-repaired mismatches (red zone) reveals the discrimination gap (gray zone). Confidence intervals (errors) of calculated propensities are shown as light color strips.

We divided mismatches into two groups based on their experimental characterization (Table 2). Best repaired mismatches are G/T, A/C, G/G and A/A, while the repair efficiency for other mismatches A/G, C/T, C/C and T/T is lower (28,69,70). First, we focused on the canonical base pairs and the best-repaired mismatches whose orientations on *N*-glycosidic bonds (aG/aT, aA/sA, aG/sG, sA/aC) are experimentally known (Figure 6A). Our results revealed no unique separation between canonical base pairs and mismatches for opening towards the major groove. On the contrary, we found a clear dissection between them for Δσ′ in the range from −40 to −65°. The size of the gap between canonical and mismatched base pairs is ∼3 kcal mol^−1^ at Δσ′ around −60°. Since aG/aT mismatch is the best-recognized mismatch by MutS, but its propensity for the opening found in our study provided the worst separation from the canonical base pairs, we will consider their difference as a discrimination gap between mismatches and canonical base pairs.

**Table 2:** The binding affinity of mismatches and canonical base pairs into MutS (71), experimentally observed nucleobase orientations on *N*-glycosidic bonds, and calculated propensities for opening and bending for the sequence class **I**

	Experimental orientation			Propensities (kcal mol^−1^)			
bp	DNA	MutS/DNA	*K*_d_ (μM)	Model orient.	Opening Δ*G_w_* (Δσ′ = −60°)	Bending Δ*G*_r_ (α = 123°)	Bending Δ*G*_r_ (δ = 1.55 Å)
A:T	*anti*/*anti*		15.00 ± 2.30	*anti*/*anti*	8.36 ± 0.08	5.76 ± 0.26	14.49 ± 0.29
G:C	*anti*/*anti*		15.00 ± 2.30	*anti*/*anti*	10.21 ± 0.10	5.33 ± 0.19	15.54 ± 0.36
C/C	*anti*/*anti*		6.90 ± 2.40	*anti*/*anti*	5.99 ± 0.07		
C/C				*anti*/*syn*	4.77 ± 0.08		
A/G	*anti*/*anti*		4.80 ± 0.90	*anti*/*anti*	7.00 ± 0.12		
A/G	*syn*/*anti*			*anti*/*syn*	2.08 ± 0.17		
A/G				*syn*/*anti*	8.20 ± 0.11		
A/C	*anti*/*anti*	*syn*/*anti*	3.40 ± 0.40	*anti*/*anti*	3.70 ± 0.08	5.15 ± 0.27	11.91 ± 0.24
A/C				*syn*/*anti*	4.87 ± 0.14	7.43 ± 0.31	18.02 ± 0.34
C/T	*anti*/*anti*		1.30 ± 0.30	*anti*/*anti*	1.44 ± 0.09		
C/T				*anti*/*syn*	2.01 ± 0.17		
C/T				*syn*/*anti*	2.11 ± 0.10		
A/A		*anti*/*syn*	1.00 ± 0.10	*anti*/*syn*	4.60 ± 0.16	5.47 ± 0.29	13.52 ± 0.26
T/T	*anti*/*anti*		1.00 ± 0.10	*anti*/*anti*	2.99 ± 0.08		
T/T				*anti*/*syn*	0.95 ± 0.18		
G/G	*anti*/*syn*	*anti*/*syn*	0.62 ± 0.07	*anti*/*syn*	4.61 ± 0.12	5.13 ± 0.25	15.44 ± 0.31
G/T	*anti*/*anti*	*anti*/*anti*	0.19 ± 0.03	*anti*/*anti*	5.25 ± 0.11	4.65 ± 0.26	17.40 ± 0.26

Due to a lack of experimental evidence, the situation is unclear with less effectively repaired mismatches (Figure 6B). Here, we can only speculate in which *anti*/*syn* orientation the opening takes place (Table 2). We found that sA/aG is at the upper boundary of the discrimination gap, while aA/aG and aC/aC lie within the gap. The other orientations, aA/sG, aC/sC, aC/aT, and aT/aT, are below the gap. Altogether, we found that from 24 possible mismatches, 18 is below the gap, 4 are within the gap (aA/aG; aC/aC; aA/sC; sG/aC), and only 2 are above the discrimination gap (sA/aG; aG/sC). More importantly, there is always at least one mismatch variant (orientation on N-glycosidic bonds), which is below or within the discrimination gap.

### Bending does not discriminate mismatches from canonical base pairs

To get data comparable with the base-pair opening, we calculated the propensity for the bending using the angle α (Figure 2D) employing the sequence class **I**. We also applied the root-mean-square-distance to target δ (Figure 2E), in which the DNA is deformed into the geometry observed in the MutS/DNA complex. We considered only best-repaired mismatches and canonical base pairs.

**Figure 7.**
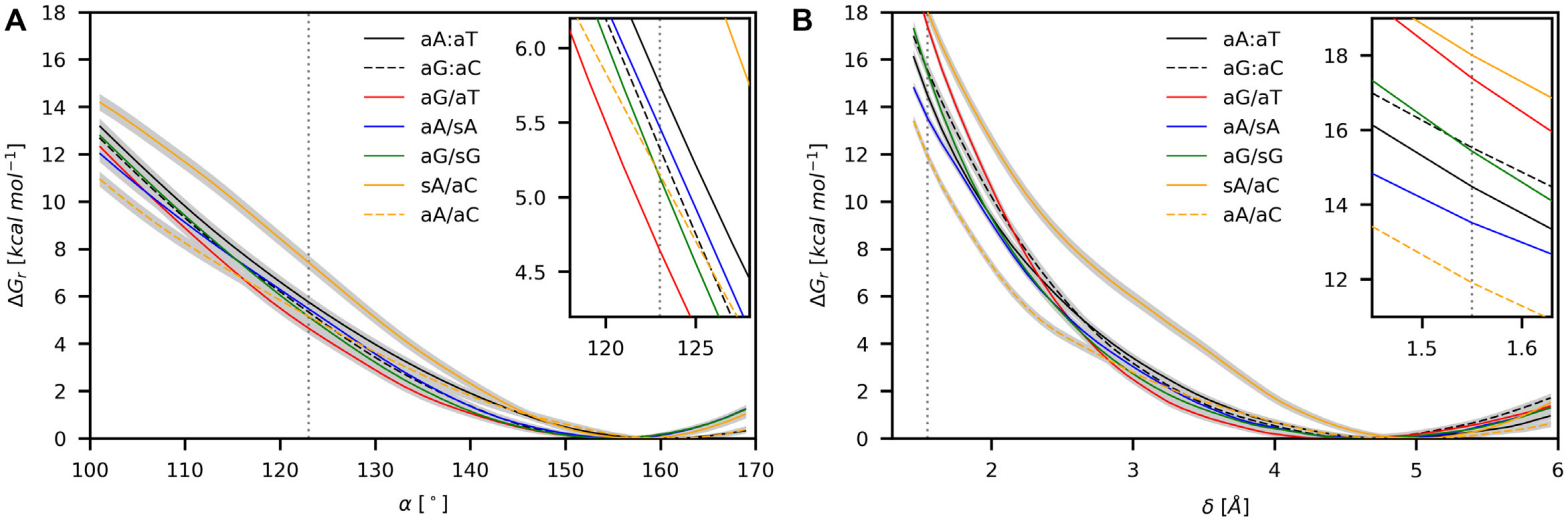
Bending free energies using (**A**) α and (**B**) δ as collective variables for selected base pairs (class **I**). The threshold values of 123° and 1.55 Å (dashed vertical lines) were taken from the experimental structure of MutSα/DNA (PDB ID: 2O8B) and Ruzicka *et al.* (3), respectively. Confidence intervals (errors) of calculated propensities are shown as light color strips.

We found no significant discrimination between canonical and mismatched systems within the error of the simulations using either approach (Figure 7, Table 2). We found that bending employing α is linear-elastic in the range of 130–170°. Stiffness constants in the limit of Hook's law showed that all mismatches except aA/aC are stiffer than aA:aT (Supplementary Table SA14). The observed order of bending propensities at experimental value α = 123° is then the results of several factors, including different equilibrium angles α_0_ (noticeably different for aA:aT) and non-linear elasticity below 130°. Bending using δ does not appear to be Hookean at all.

Among all tested mismatches, sA/aC showed significant resistance to the bending. The sA/aC mismatch is oriented into the major groove (see the global minimum in Supplementary Figure SB21.1), where it can clash with adjacent canonical base pairs during the bending. However, the same feature was exhibited by aG/sG (Supplementary Figure SB16.1) and aA/sA (Supplementary Figure SB11.1), which did not show such resistance. A probable reason is the width of the minor groove. We found that the sA/aC minor groove is narrowed already in the relaxed state. Thus, its expansion during bending (Supplementary Figure SA15) can be responsible for observed resistance.

### The discrimination gap is preserved across different sequence contexts

**Figure 8.**
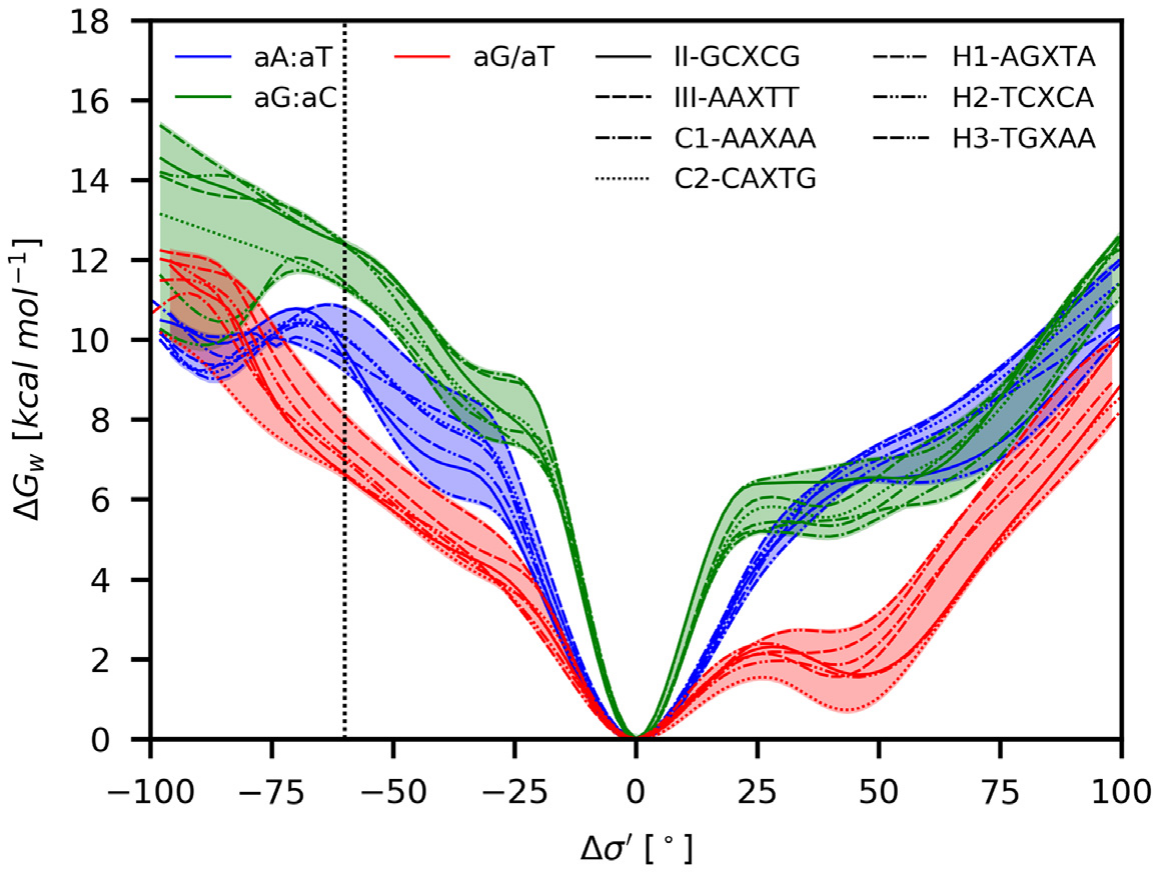
The propensity for the opening quantified as Δ*G*_w_(Δσ′) for the movement towards the minor (Δσ′ < 0) and major (Δσ′ > 0) grooves in the various sequence contexts. The vertical line at −60° corresponds to the average experimental value (see Figure 9 and Supplementary Table SA3).

We added seven sequence classes (Table 1) to test the impact of nucleobases surrounding the mismatch on the discrimination gap. We tested only canonical base pairs and the aG/aT mismatch, which was shown to be the most resilient for the opening. The obtained results are shown in Figure 8 and Table 3.

**Table 3:** Propensities for the opening of the aG/aT mismatch and canonical base pairs in various sequence contexts and discrimination gaps between the mismatched and canonical base pairs

		Propensities (kcal mol^−1^)			
		Opening	Gap		
Class	bp	Δ*G*_w_ (Δσ′ = −60°)	aG:aC↔aG/aT	aA:aT↔aG/aT	min(aA:aT)^b^↔aG/aT^a^
**I**	aA:aT	8.36 ± 0.08	4.96 ± 0.14	3.11 ± 0.14	N/A
	aG:aC	10.21 ± 0.10			
	aG/aT	5.25 ± 0.11			
**II**	aA:aT	9.76 ± 0.07	5.77 ± 0.18	3.14 ± 0.13	2.60 ± 0.13
	aG:aC	12.38 ± 0.13			
	aG/aT	6.61 ± 0.11			
**III**	aA:aT	10.80 ± 0.07	4.98 ± 0.15	3.40 ± 0.14	1.82 ± 0.13
	aG:aC	12.37 ± 0.10			
	aG/aT	7.40 ± 0.12			
**C1**	aA:aT	9.56 ± 0.06	4.32 ± 0.14	2.59 ± 0.13	2.24 ± 0.13
	aG:aC	11.29 ± 0.08			
	aG/aT	6.97 ± 0.11			
**C2**	aA:aT	10.08 ± 0.07	4.71 ± 0.14	3.47 ± 0.13	2.61 ± 0.13
	aG:aC	11.32 ± 0.08			
	aG/aT	6.60 ± 0.11			
**H1**	aA:aT	10.03 ± 0.07	4.30 ± 0.16	1.95 ± 0.14	1.13 ± 0.14
	aG:aC	12.38 ± 0.10			
	aG/aT	8.08 ± 0.12			
**H2**	aA:aT	9.51 ± 0.07	5.35 ± 0.14	2.44 ± 0.14	2.14 ± 0.13
	aG:aC	12.43 ± 0.08			
	aG/aT	7.07 ± 0.11			
**H3**	aA:aT	9.22 ± 0.06	4.8 ± 10.13	2.58 ± 0.12	2.58 ± 0.12
	aG:aC	11.44 ± 0.08			
	aG/aT	6.64 ± 0.08			
**min**			4.30	1.95	1.13
**max**			5.77	3.47	2.61

^a^Only II, III, C1, C2, H1, H2, H3, which uses 15-nt long DNA models.

^b^The minimum value from the opening propensities of aA:aT, which was found for H3.

While the class **I** is 13-nt long, the additional classes are 15-nt long. Comparison of classes **I** and **III**, which have the same central 5-nt log segment, revealed nearly the same discrimination gap of 3.1 and 3.4 kcal mol^−1^, respectively. While the width of the gap is maintained, the longer DNA exhibited gap shifted up by about 2.2 kcal mol^−1^ (Supplementary Figure SA14). This finding suggests that the resistance for the opening is mainly mediated by π-stacking, which is stronger in the longer DNA. The discrimination gap was also detected for other sequence motifs, which were selected with different susceptibility for a mutation in genes associated with common inherited disorders (3).

### Base-pair opening and mismatch recognition by MutS

In the previous sections, we have shown that the base-pair opening into the minor grove can discriminate between mismatched and canonical base pairs in the absence of MutS, while the bending of DNA did not exhibit such behavior. We can see some agreement with the experimental work of Wang *et al.* (24), who determined the bending of DNA by MutS via Atomic Force Microscopy. It was found that MutS bends DNA regardless of mismatch presence. However, when MutS detects a mismatch, its state is changed into a sliding clamp that does not bend DNA anymore (72). Later, similar conclusions were drawn by Hura *et al.* using X-ray scattering (73). Thus, we can speculate that the base-pair opening represents an intrinsic feature of damaged dsDNA that could have been exploited during the evolution of MMR.

**Figure 9.**
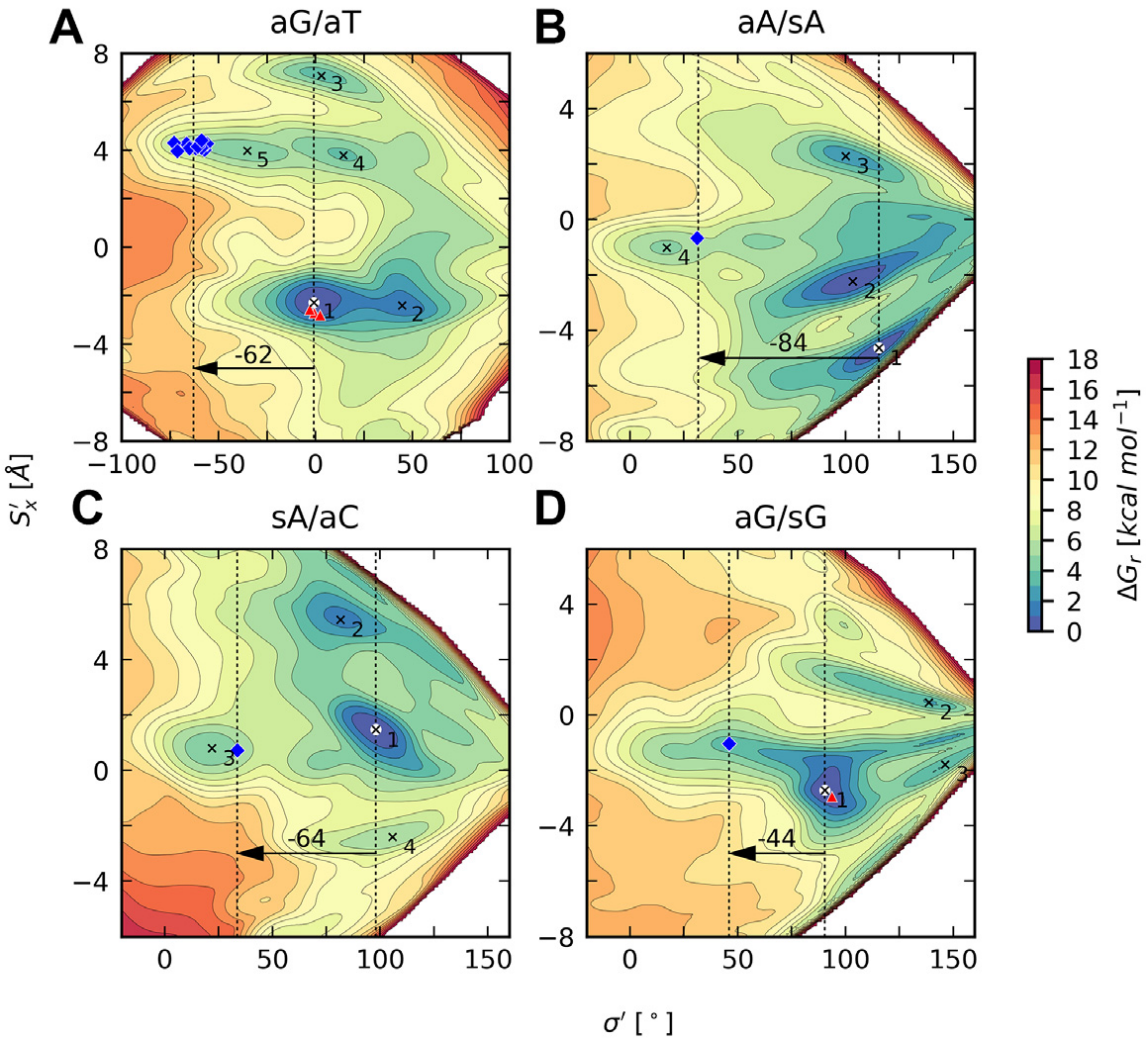
Free energy surfaces for the selected class **I** sequences overlaid with crystallographic geometries of mismatches in MutS/DNA complexes (blue diamonds) and unbound DNAs (red triangles). Vertical dotted lines highlight values of Opening in the most stable structure (white point) and structures of flipped out base pairs. Arrows show the necessary change of Opening during the mismatch recognition by MutS. Free energy minima are labeled in ascending order. Free energy isolines are spaced by 1 kcal mol^−1^.

Our conclusions are derived from a simplified model (Figure 1F), but its validity seems to be supported by experimental geometries of mismatched base-pairs in both free and bound forms. In the free dsDNA, geometries of mismatches fit into the calculated free energy surfaces Δ*G*_r_(σ′,*S*′_x_), i.e. geometries are close to the free energy minima (Supplementary Table SA4 and Supplementary Figure SA12). Interestingly, a similar fit was also found for mismatches in dsDNA bound to MutS (Supplementary Table SA3 and Figure 9). Here, geometries are located either in a shallow valley or close to a minimum on the free energy surfaces. We would like to recall that the free energy surfaces were calculated for relaxed dsDNA in the absence of MutS, while the experimental geometries are from significantly bent dsDNA induced by the complexation with MutS. This observation indicates some transferability of results obtained from the simplified model (Figure 1F) to a description that would be more realistic (Figure 1E). We think this is mainly caused by the usage of Opening and Shear parameters, whose values depend solely on mutual rearrangements of mismatched nucleobases, i.e. their geometrical definition is local. This locality makes σ′ and *S*′_x_ largely invariant to geometry changes in their surroundings. Consequently, the calculated free energy surface Δ*G*_r_(σ′,*S*′_x_) will not be too much different (at least positions of free energy minima) in the relaxed and bent states if other interactions perpendicular to the base-pair opening are properly maintained. In our model, this is achieved by the adjacent nucleobase pair, which establishes stacking interaction otherwise provided by conserved PHE intercalator (compare Figure 1E and F).

Experimental evidence indicates that the mismatch recognition by MutS is not the only factor playing a role in MMR (71). The repair efficiencies correlate with the affinity towards the MutS, but exceptions are A/C and T/T, which bind weaker and tighter than expected from their repair efficiency, respectively. When we tried to compare our results with the experimentally observed *K*_d_ (Table 2), no clear correlation was found. Also, the discrimination gap observed for the base-pair opening was found to be smaller than the difference between the experimental binding affinities of G/T and A:T. The most likely cause of this observation is the usage of a very simplified model. The significant portion of the missing energy will be from the specific interactions with MutS and necessary *anti*↔*syn* transitions occurring during recognition of some mismatches. The later can be expected to be always positive, i.e. making the recognition less effective. On the contrary, the interaction with the conserved GLU-X-PHE motif can improve (boost) the recognition. The boosting will probably differ between mismatches due to different protonation state of the conserved GLU, as suggested by experimental structures of MutS/DNA complexes (Supplementary Figure SA5). Also, different protonation forms or tautomers of nucleobases can play a role as well.

Another limitation of our model comes from the stabilization of some thermodynamic states by direct interactions with water molecules or sodium cations due to the absence of MutS. The amount of data collected for each thermodynamic state did not allow us to perform detailed analysis, but we expected that such interactions could occur only for significantly distorted base pairs when nucleobases are fully exposed to the bulk.

Mutation in genes associated with common inherited disorders were analyzed to get sequence motives with different susceptibility for mutations (3). We want to highlight that the obtained hotspot/coldspot classification is based on medical data, which reflect mutations detected in monitored patients. Their emergence is influenced by many factors, including all ways of mismatch formation and their resolving by all repair pathways and not necessarily by MutS. Since the MutS does not compare mismatches with their canonical counterparts, MutS must apply some internal threshold, which discriminates the mismatches. In our simplified model, we selected this threshold as the highest propensity (lowest Δ*G*_w_(Δσ′)) for the canonical base-pairs opening with the same DNA length (Table 3, **H3**). All tested aG/aT mismatches are below this threshold. Due to the accuracy of our simulations, we do not want to speculate about the order or correlation with hotspot/coldspot classification. Nevertheless, the **H1** hotspot, which had remarkably low combined Fisher *P*-value in the bioinformatics analysis (Table 1) (3), showed the smallest discrimination from the canonical base pairs for the opening into the minor groove. This finding suggests that MutS could be less effective in the detection of errors in the sequence context of **H1**, explaining its experimentally observed high mutability.

## CONCLUSIONS

The mismatch recognition is a crucial step of the mismatch repair. Experimental structures of MutS/DNA complexes revealed noticeably bent DNA suggesting that easier bendability of mismatched DNA is a key feature exploited by MutS. In this work, we calculated propensities for DNA bending. Even though we used different DNA sequences, force field, and methodology for free energy calculations than in the previous study (28), we obtained similar results showing no clear dissection between the canonical and mismatched base pairs. We also attempted to bend DNA into the shape experimentally observed in the MutS/DNA complexes, but despite a more sound bending approach (4), calculated propensities led to similar conclusions.

Detailed analysis of available crystal structures of MutS/DNA complexes revealed other motion, which could be sensed by MutS. This motion is the opening of a mismatched base pair into the minor groove, stabilized by glutamate from the conserved GLU-X-PHE motif. For the description of this motion, we employed a simplified model. In this model, we evaluated thermodynamic stabilities of all possible mismatches and canonical base pairs by extensive biased MD simulations employing two *simple* base-pair parameters Opening and Shear (67).

Base-pair parameters have been used in structural analysis for over 30 years (74), and they are also widely used in the analysis of nucleic acid deformability employing unbiased MD simulations (75). However, up to our best knowledge, their importance in the free energy evaluation of base pairs from biased MD simulations was recognized only recently (76,77). In this study, we have shown that Opening and Shear are irreplaceable by other geometrical parameters. Interestingly, two pseudo-dihedral angles, ϕ_1_ and ϕ_2_, determining respective positions of two bases towards the DNA skeleton (30) counterintuitively failed to resolve thermodynamic states revealed by Opening and Shear.

A comparison of calculated thermodynamic states with available experimental structures showed a good agreement for mismatches in unbound DNA and, more importantly, in DNA bound to MutS as well. Especially in the minor groove, mismatches exhibit stable, albeit energetically less favorable structures while the canonical base pairs do not. The calculated propensity for the opening in various sequence contexts in the absence of MutS showed a clear discrimination gap between mismatches and canonical pairs of about 2.6–3.1 kcal mol^−1^ wide, revealing intrinsic property of DNA, which could be exploited by MutS during recognition.

While our results showed a clear dissection between mismatched and canonical base pairs for the opening, the observed discrimination gap is smaller than it would follow from experimentally determined affinities of DNA towards MutS. This observation suggests that MutS can boost the gap by specific interactions with conserved GLU-X-PHE motif. In the follow-up studies, we would like to study these phenomena in more detail.

Additionally, we characterized about 94 thermodynamic states of all nucleobase combinations in *anti*/*anti*, *anti*/*syn*, *syn*/*anti* orientations in a consistent manner. This catalog can be used for further improvements of empirical force fields describing nucleic acids and help structural biologists in the determination of experimental structures containing base pairs in distorted geometries. Further, provided structures can be employed in tuning properties of chemical compounds that selectively bind to mismatches (5), which can act as blockers of corrupted DNA replication. Moreover, adequately calculated propensities for base-pair opening together with developed methodology can help in a better understanding of sequence-dependent mutability (3,4).

## Supplementary Material

gkaa896_Supplemental_Files
