# Supplementary material for: Importance of base-pair opening for mismatch recognition
Source: Nucleic Acids Res. 2020 Oct 20;48(20):11322–34. doi: 10.1093/nar/gkaa896 (PMC7672436; doi:10.1093/nar/gkaa896)
Supplement: gkaa896_Supplemental_Files [file gkaa896_supplemental_files.zip › DNAMismatches006_SD_PartA.pdf]

Importance of Base-Pair Opening for Mismatch Recognition

Tomáš Bouchal,[1,2] Ivo Durník,[1,2] Viktor Illík,[2] Kamila Réblová,[1] and Petr Kulhánek[1,2,*]

1) CEITEC - Central European Institute of Technology, Masaryk University, Kamenice 5, 625 00 Brno, Czech Republic

2) National Centre for Biomolecular Research, Faculty of Science, Masaryk University, Kamenice 5, 625 00 Brno, Czech Republic

*) Corresponding author: e-mail: kulhanek@chemi.muni.cz; phone: +420 549 495 459; ORCID: 0000-0002-4152-6514

SUPPLEMENTARY DATA - PART A

Contents

I. MODELS ... 3
Figure SA1 ... 3
Figure SA2 ... 3
II. SIMPLE BASE-PAIR PARAMETERS ... 4
Figure SA3 ... 4
Figure SA4 ... 5
Table SA1 ... 5
III. BASE-PAIR OPENING IN EXPERIMENTAL MUTS/DNA COMPLEXES ... 6
Table SA2 ... 6
Figure SA5 ... 7
Table SA3 ... 8
Table SA4 ... 9
IV. UNBIASED MOLECULAR DYNAMICS SIMULATIONS ... 10
Molecular Dynamics Simulations ... 10
Figure SA6 ... 10
Unbiased MD Simulations and Rare Events ... 10
V. BIASED MOLECULAR DYNAMICS SIMULATIONS ... 11
Adaptive Biasing Force (ABF) Method ... 11
Multiple-walker Approach (MWA) ... 12
Opening and Shear as Collective Variables ... 13
Table SA5 ... 13
Wall Restraints ... 14
Table SA6 ... 14
Table SA7 ... 15

Integration of Mean Forces by Gaussian Process Regression (GPR) 16
Gaussian Process Kernels 17
Optimization of GPR Hyperparameters 18
Figure SA7 18
Figure SA8 19
Figure SA9 20
Table SA8 21
Table SA9 21
Table SA10 22
Table SA11 22
Table SA12 22
Table SA13 22
Error Analysis 23
Figure SA10 23
Statistical Averaging 24
Final Remarks on the Reconstruction of the Free Energies 24
VI. ANALYSIS OF ABF/MWA SIMULATIONS 25
Analysis of Free Energy Minima 25
Figure SA11 25
Figure SA12 26
Base-pair Opening 27
Figure SA13 27
Figure SA14 27
DNA Bending Employing α 28
Table SA14 28
Figure SA15 28
Impact of Salt on DNA Bending 29
Figure SA16 29
VII. REFERENCES 30

I. MODELS

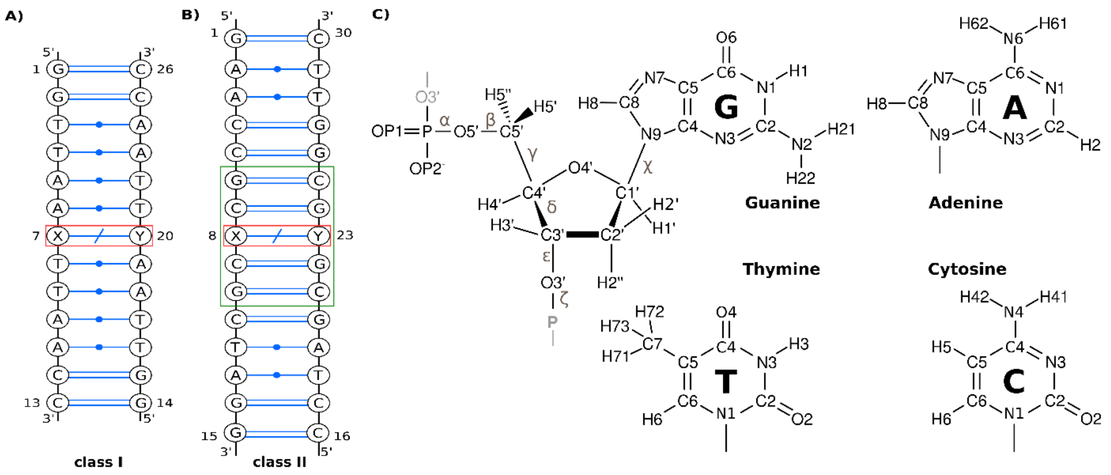

Figure SA1. Studied models of dsDNAs with the sequence class **I** (A) and class **II** (B) and various central base pair X/Y highlighted in the red box. Classes **III**, **C1**, **C2**, **H1**, **H2**, and **H3** were derived from the class **II** by changing the sequence in the green box, see the main text for further details. (C) Structures of nucleotides with the atom numbering and definition of backbone and glycosidic bond torsion angles.

anti-anti

	A	G	C	T
**A**	aA/aA	aA/aG	aA/aC	aA:aT
**G**	aG/aA (aA/aG)	aG/aG	aG:aC	aG/aT
**C**	aC/aA (aA/aC)	aC:aG (aG:aC)	aC/aC	aC/aT
**T**	aT:aA (aA:aT)	aT/aG (aG/aT)	aT/aC (aC/aT)	aT/aT

anti-syn

	A	G	C	T
**A**	aA/sA	aA/sG	aA/sC	aA/sT
**G**	aG/sA (sA/aG)	aG/sG	aG/sC	aG/sT
**C**	aC/sA (sA/aC)	aC/sG (sG/aC)	aC/sC	aC/sT
**T**	aT/sA (sA/aT)	aT/sG (sG/aT)	aT/sC (sC/aT)	aT/sT

syn-anti

	A	G	C	T
**A**	sA/aA (aA/sA)	sA/aG	sA/aC	aA/sT
**G**	sG/aA (aA/sG)	sG/aG (aG/sG)	sG/aC	sG/aT
**C**	sC/aA (aA/sC)	sC/aG (aG/sC)	sC/aC (aC/sC)	sC/aT
**T**	sT/aA (aA/sT)	sT/aG (aGsT)	sTaC (aCsT)	sTaT (aTsT)

Figure SA2. Equivalences between simulated combinations of nucleobases and orientations on *N*-glycosidic bonds of X/Y base pair due to class **I** palindromic sequence. Simulated systems are in gray cells. White cells show systems, which are equivalent to the systems provided in the parentheses.

II.SIMPLE BASE-PAIR PARAMETERS

The geometry description of base pair and its change during base-pair opening can be quantified in more than one way. However, the most established mathematical description is provided by six base-pair parameters (Shear, Stretch, Stagger, Buckle, Propeller, and Opening - Figure SA3). These parameters, together with the step and helical parameters, are available through the well-recognized Curves (1), Curves+ (2), and 3DNA (3, 4) programs.

Depending on used software, they exist in several variants (*global*, *local*, *simple*) (2, 3, 5). In this work, we used the *simple* base-pair parameters as introduced in the 3DNA package because they seem to be most suitable for the description of structural variations of non-canonical base pairs (6).

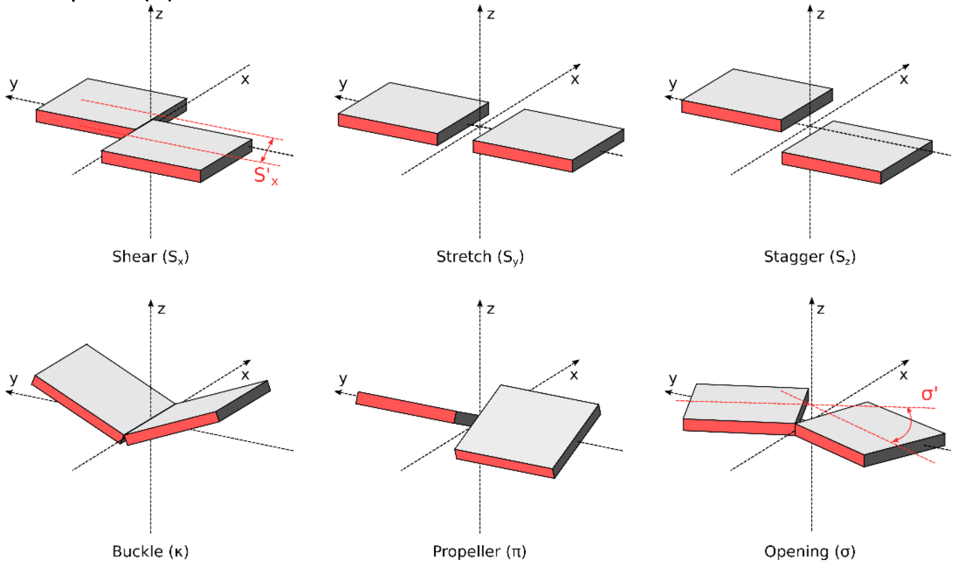

Figure SA3. Schematic representation of six rigid-body parameters commonly used for characterization of base-pair geometry. The sugar edge of nucleobases is highlighted in red, indicating *anti*/*anti* orientations. Base-pair parameters for other than *anti*/*anti* orientations were transformed.

While canonical base pairs contain both nucleobases in *anti*-orientation on the *N*-glycosidic bonds, some mismatches contain nucleobases in *syn*-orientation. Unfortunately, *anti*/*syn* transition has a significant impact on a sign and values of *simple* base-pair parameters, which make their comparison difficult between different mismatches. Since the parameters are calculated in the context of nucleobase reference frames (5), we employed two different sets of reference frames. The standard reference frames were used for nucleobases in *anti*-orientation, while their counterparts employed for *syn*-nucleobases were obtained by the rotating of the standard reference frames by 180° around the y-axis, mimicking *anti*→syn transition (Figure SA4). Employed transformations of the reference frames do not scale but only shift values of the base-pair parameters; see a relationship between the standard and transformed parameters provided in Table SA1.

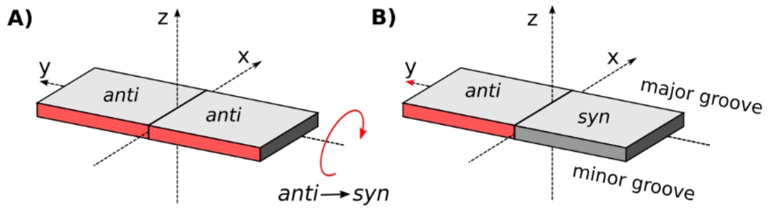

Figure SA4. The rationale for the usage of transformed reference frames for nucleobases in the *syn* orientation on the *N*-glycosidic bond. The sugar edge of nucleobases is highlighted in red.

Transformed parameters are denoted by an apostrophe, for example, as σ' and S'_x for Opening and Shear, respectively. The outcome of these transformations is the following. Positive values of Opening σ' show the base pair opening towards the major groove, while negative values show movement towards the minor groove regardless of *anti*/*syn*-orientation of nucleobase. In the case of S'_x, positive values show a movement of the chain A base into the major groove and the chain B base into the minor groove, while negative values show the opposite behavior.

Table SA1. The relation between the transformed *simple* base-pair parameters (denoted with an apostrophe, S'_x, S'_y, S'_z, κ', π', and σ') employed in biasing simulations and standard *simple* base-pair parameters (S_x, S_y, S_z, κ, π, and σ) provided by 3DNA.

orientations	S'_x	S'_y	S'_z	κ'	π'	σ'
anti/anti	S_x	S_y	S_z	κ	π	σ
anti/syn	S_x	S_y	S_z	κ	π	σ+180°
syn/anti	$-S_x$	S_y	$-S_z$	-κ	π	-(σ+180°)

III. BASE-PAIR OPENING IN EXPERIMENTAL MUTS/DNA COMPLEXES

Experimental MutS/DNA complexes revealed that the mismatch opening is accompanied by movement of its nucleobases towards the minor groove of DNA, where they interact with the recognition domain of MutS containing PHE-X-GLU motive (Figure 1). We attempted to quantify this movement by comparing experimental geometries of unbound DNA with DNA bound to MutS. Structures were extracted from the PDB (7) and NDB (8) databases. We kept only those containing isolated mismatches. Structures with mismatches close to insertion/deletion loops or structures containing multiple mismatches next to each other were excluded as massive damages. Altogether, we were able to find 20 structures solved by X-ray and 6 by NMR; among them, 14 structures were MutS/DNA complexes (all structures are summarized in Tables SA3 and SA4). Available MutS/DNA complexes revealed information about four types of mismatches: aG/aT, aA/sA, aG/sG, and sA/aC.

A comparison of the *simple* base-pair parameters of the mismatches in bound and unbound DNAs are summarized in Table SA2. In the case of aA/sA and sA/aC mismatches, values for the unbound state were taken from unbiased MD simulations because no experimental structures were found. Here, we assume that the opening occurs on a geometry, which already adopts the orientation observed in the bound state. Then, the necessary *anti*/*syn* change for some mismatches is assumed to occur before the opening as a result of thermodynamic equilibrium, most likely by nucleobase flipping into the grooves, where there is enough space for the transition, as suggested by experimental and computational studies (9).

Table SA2. *Simple* base-pair parameters for mismatches in unbound and MutS bound states. Cells highlighted in a gray show the most substantial changes during mismatch recognition.

			Shear		Stretch		Stagger		Buckle		Propeller		Opening	
mismatch	state	method	S'_x	$s(S'_x)$	S'_y	$s(S'_y)$	S'_z	$s(S'_z)$	κ'	$s(\kappa')$	π'	$s(\pi')$	σ'	$s(\sigma')$
aG/aT	DNA	X-ray	-2.69	0.08	0.00	0.08	-0.10	0.04	1.9	7.0	-11.9	0.4	-0.5	1.9
	MutS/DNA	X-ray	4.16	0.14	2.66	0.18	-0.39	0.40	-28.7	6.0	-16.8	5.2	-63.0	5.5
	Δ		6.86		2.66		-0.29		-30.7		-4.9		-62.5	
aA/sA	DNA	MD	-2.24	0.01	3.61	0.01	-0.02	0.01	3.8	0.1	-7.2	0.1	104.0	0.1
	MutS/DNA	X-ray	-0.71	0.04	1.84	0.22	-0.46	0.22	-16.7	2.5	-28.5	0.9	31.7	0.7
	Δ		1.53		-1.77		-0.44		-20.5		-21.3		-72.3	
aG/sG	DNA	X-ray	-2.91	0.03	2.24	0.01	0.12	0.26	13.2	3.3	-13.6	1.8	91.2	2.5
	MutS/DNA	X-ray	-1.04		2.41		0.12		-25.6		-20.0		46.0	
	Δ		1.87		0.17		0.00		-38.8		-6.4		-45.3	
sA/aC	DNA	MD	1.63	0.01	1.70	0.00	1.13	0.01	10.4	0.1	-18.5	0.1	95.9	0.1
	MutS/DNA	X-ray	0.72		0.28		0.47		11.6		-14.9		33.6	
	Δ		-0.91		-1.42		-0.66		1.2		3.6		-62.3	

Δ – difference between bound and unbound state; s(X) – standard deviation if there is more than one structure; structures employed in the analysis are listed in Table SA3 and SA4; MD – 150 ns long unbiased molecular dynamics simulation.

The most substantial changes between bound and unbound states were observed for Opening with the average change of about -60°. This change can be attributed to the interaction of mismatch with conserved GLU, which drags the mismatch towards the minor groove. A large change in Buckle of aG/aT, aA,sA, and aG/sG is caused by the intercalation of conserved PHE into the DNA. Interestingly, such change is not observed for aA/aC, even though the intercalation takes place as well. In the case of aG/aT, a significant change in Shear is also observed. The shift in Shear is a result of the reorganization of the hydrogen network in the mismatch upon binding by MutS.

Moreover, a detailed analysis of individual mismatches in the active site of MutS (Figure SA5) also suggested that the GLU residue is most likely in the deprotonated (aG/aT) or protonated (aA/sA, aG/sG, sA/aC) form.

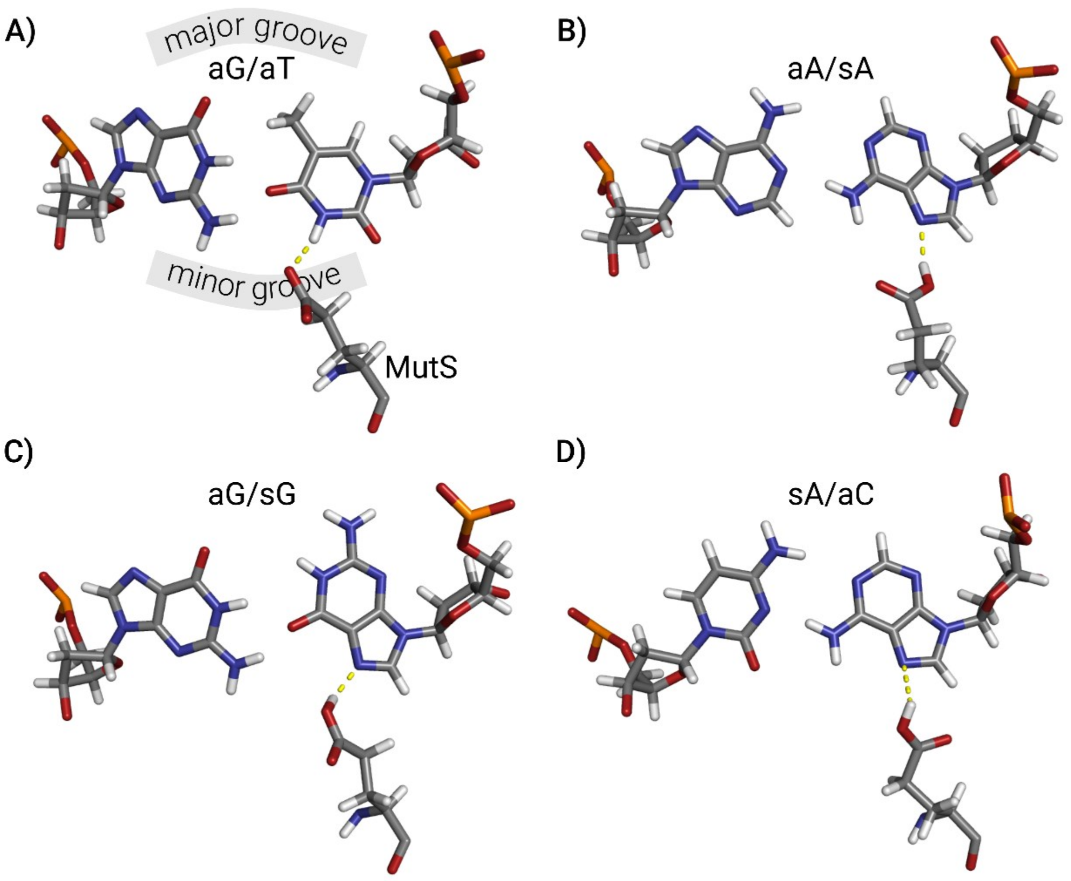

Figure SA5. Structures of mismatches and their interaction with the GLU from the conserved GLU-X-PHE motif in available crystal structures of MutS/DNA complexes: (A) aG/aT (PDB ID: 2O8B), (B) aA/sA (PDB ID: 1OH6), (C) aG/sG (PDB ID: 1OH7), and (D) sA/aC (aC/sA, PDB ID: 1OH5). Hydrogen atoms were added *in silico*. View direction is along the z-axis of DNA.

Table SA3. *Simple* base-pair parameters of mismatches in experimental structures of MutS/DNA complexes. All structures were determined by X-ray crystallography. Parameters are transformed for other than *anti*/*anti* orientations, see Table SA1 for further details.

		**Shear**	**Stretch**	**Stagger**	**Buckle**	**Propeller**	**Opening**
**mismatch**	**PDBID**	S'_x	S'_y	S'_z	κ'	π'	σ'
aA/sA	1OH6	-0.67	2.07	-0.24	-19.3	-27.6	31.0
	2WTU	-0.75	1.62	-0.68	-14.2	-29.4	32.5
aG/aT	1E3M	4.01	2.35	-0.71	-27.2	-17.7	-57.2
	1NG9	4.27	2.51	-0.75	-22.7	-12.5	-56.0
	1W7A	4.10	2.47	-0.51	-29.4	-19.4	-58.9
	1WB9	4.08	2.65	-0.56	-25.7	-19.7	-61.9
	1WBB	4.27	2.64	-0.55	-24.1	-15.3	-66.7
	1WBD	4.08	2.81	-0.64	-27.6	-21.7	-65.0
	2O8B	4.30	2.94	0.31	-41.8	-12.2	-72.9
	2O8E	3.96	2.55	0.45	-36.3	-5.2	-71.3
	3K0S	4.15	2.82	-0.61	-31.0	-22.6	-60.9
	3ZLJ	4.42	2.83	-0.32	-21.6	-21.6	-58.9
aG/sG	1OH7	-1.04	2.41	0.12	-25.6	-20.0	46.0
sA/aC	1OH5	0.72	0.28	0.47	11.6	-14.9	33.6

Table SA4. *Simple* base-pair parameters of mismatches in experimental structures of DNA. The parameters are transformed for other than *anti/anti* orientations, see Table SA1 for further details.

			Shear		Stretch		Stagger		Buckle		Propeller		Opening	
mismatch	PBDID	method	S'_x	$s(S'_x)$	S'_y	$s(S'_y)$	S'_z	$s(S'_z)$	κ'	$s(\kappa')$	π'	$s(\pi')$	σ'	$s(\sigma')$
aA/aC	1D99	X-ray	-1.77		-0.14		0.22		9.3		-10.7		-2.1	
	1D99	X-ray	-2.17		0.21		0.27		7.4		-14.2		9.0	
	2MO7	NMR	-2.24	1.18	0.37	0.15	-0.06	0.16	8.5	4.4	-24.2	4.5	2.3	2.5
	2MO7	NMR	-2.25	0.14	0.39	0.06	-0.10	0.11	6.3	2.8	-22.5	1.5	2.8	1.4
aA/aG	1ONM	NMR	-0.18	0.25	1.52	0.07	-0.26	0.26	-3.8	3.4	-1.3	5.9	-12.7	3.0
aC/aC	1FKZ	NMR	1.14		-1.90		1.67		0.6		-14.5		-4.3	
	1FKZ	NMR	-1.79		-1.49		1.30		2.1		-6.1		5.6	
aC/aT	1FKY	NMR	0.30		-1.71		0.72		3.2		-7.8		5.8	
	1FKY	NMR	0.65		-0.35		0.01		-4.3		-5.3		-24.1	
aG/aT	1BJD	X-ray	-2.71		-0.08		-0.14		-5.0		-11.7		-0.6	
	1BJD	X-ray	-2.71		-0.08		-0.14		-4.9		-11.7		-0.6	
	113D	X-ray	-2.57		0.02		-0.04		10.8		-11.5		-3.1	
	113D	X-ray	-2.80		0.12		-0.10		6.7		-12.6		2.3	
	1KKW	NMR	-1.23	0.01	-0.31	0.01	0.83	0.06	18.6	1.4	-14.0	0.4	-12.8	0.8
	1KKW	NMR	-1.22	0.01	-0.29	0.01	0.83	0.06	19.6	1.6	-15.0	0.4	-12.8	0.6
aG/sG	1D80	X-ray	-2.94		2.25		0.38		16.5		-15.4		93.8	
	1D80	X-ray	-2.88		2.23		-0.14		9.8		-11.8		88.7	
aT/aT	2LL9	NMR	-2.88	0.24	-0.89	0.19	-0.03	0.29	5.7	1.7	-8.6	2.0	1.4	2.7
sA/aG	112D	X-ray	3.85		2.61		0.08		-15.7		-13.7		93.8	
	112D	X-ray	3.27		2.64		0.12		-9.1		-10.3		98.8	
	1DNM	X-ray	3.61		2.46		0.46		-6.9		-19.5		88.3	
	1DNM	X-ray	3.54		2.37		0.39		5.7		-15.2		85.6	

s(X) is the standard deviation calculated from all available NMR models.

IV.UNBIASED MOLECULAR DYNAMICS SIMULATIONS

Molecular Dynamics Simulations. We employed the Amber 16 package (10, 11), the parmbsc1 (12) force field for DNA, sodium (13) cations to maintain electroneutrality, and TIP3P water (14). All simulations were performed in an explicit solvent under the periodic boundary conditions employing the truncated octahedral box. Long-range interactions were treated with the particle-mesh Ewald method (15), with a direct summation cutoff set to 8.0 Å. The same cutoff was used for Lennard-Jones interactions. Equations of motions were integrated with a time step of 2 fs and constrained lengths of bonds containing hydrogen atoms by SHAKE (16).

Each system was equilibrated by geometry optimization followed by heating (100 ps) to 300 K at a constant volume employing the Langevin thermostat with a collision frequency (γ) of 1.0 ps^{-1}. Finally, the proper density was adjusted by short simulation (500 ps) at the constant temperature (the same thermostat as in the previous step) and pressure maintained by the barostat set to 100 kPa with a feedback time constant (t_p) of 1.2 ps.

After equilibration, MD simulations were performed at a constant temperature of 300 K (Berendsen thermostat, t_T=5 ps) and a pressure of 100 kPa (weak coupling barostat, t_p=6 ps). Unbiased simulations were run on GPU accelerators (11) and were 150 ns long each except for the aG/aT system from the class **I,** which was prolonged to 5 μs.

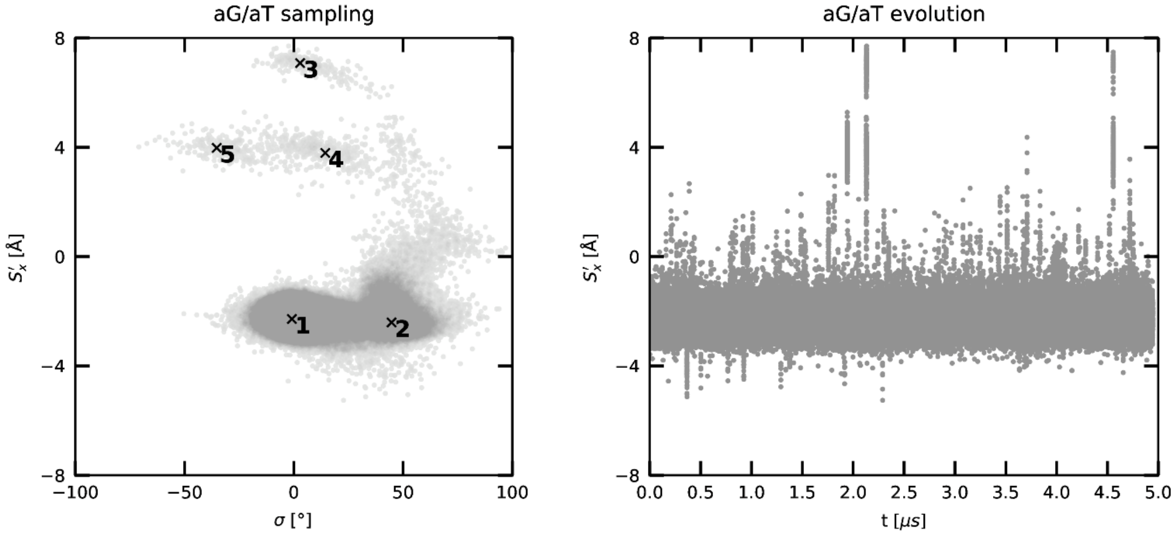

Figure SA6. (A) A sampling of Opening σ' and Shear S'_x of the aG/aT mismatch (class **I**) during 5 µs long unbiased MD simulation. (B) The time evolution of S'_x during the simulation indicates rare events for a transition to positive values of S'_x.

Unbiased MD Simulations and Rare Events. Unbiased simulations suffer from an inadequate sampling of thermodynamic states. This problem is demonstrated for the aG/aT system (class **I**) in Figure SA6, which reports values of Opening σ' and Shear S'_x along 5 μs long unbiased MD simulation. Regions with positive values of S'_x are infrequently visited as they are higher in the free energy (ΔG_r > 3.7 kcal mol^{-1}) and separated from the most stable state by the free energy barrier of about 5.4 kcal mol^{-1}. Infrequent transitions on time scale of MD simulations are rare events, and their appearance discourages sampling of thermodynamically important states and evaluation of their respective free energies. In our work, we overcame the problem by employing the adaptive biasing force method.

V.BIASED MOLECULAR DYNAMICS SIMULATIONS

Adaptive Biasing Force (ABF) Method is an approach, which calculates the free energy as a function of selected collective variables (17, 18). Here, we will provide the necessary overview of ABF and its implementation in the PMFLib package (19), which was used in this work. The free energy calculation is achieved by introducing a bias, which removes barriers or higher free energy regions alongside predefined collective variables ξ. As a result, the system evolves alongside these collective variables by free diffusions. The bias is derived from the free energy ($G(\boldsymbol{\xi})$), which is projected in the form of biasing force ($\boldsymbol{F}_{bias}$) to the Cartesian space and removed from force ($\boldsymbol{F}_{pot}$) originated from interatomic interaction potential ($V(\boldsymbol{x})$). The application of the bias thus leads to the modified equations of motions:

$$m_i \frac{d^2 x_i}{dt^2} = \boldsymbol{F}_{pot,i}(\boldsymbol{x}_i) - \boldsymbol{F}_{bias,i}(\boldsymbol{x}_i) = -\left(\frac{\partial V(x)}{\partial x_i} - \frac{\partial G(\xi)}{\partial \xi}\frac{\partial \xi}{\partial x_i}\right) \quad (1)$$

where m_i is mass of atom i, $\boldsymbol{x}_i$ is atom position, t is time.

The free energy gradient $\partial G(\boldsymbol{\xi})/\partial \boldsymbol{\xi}$ is calculated from a mean of instantaneous collective force ($\boldsymbol{F}_{ICF}$) derived from the time evolution of collective variables:

$$\frac{\partial G(\xi^*)}{\partial \xi} = -\langle \boldsymbol{F}_{ICF} \rangle_{\xi=\xi^*} \quad (2)$$

$$\boldsymbol{F}_{ICF} = \frac{d}{dt}\left(\boldsymbol{Z}^{-1}\frac{d\xi}{dt}\right) \quad (3)$$

where $\boldsymbol{Z}$ is the matrix in the form:

$$[\boldsymbol{Z}]_{ij} = \sum_k^{atoms} \frac{1}{m_k}\frac{\xi_i}{\boldsymbol{x}_k}\frac{\xi_j}{\boldsymbol{x}_k} \quad (4)$$

The analytical calculation of instantaneous collective force by Equation 3 requires the second derivatives of collective variables with respect to Cartesian coordinates. Since this can be prohibitive for complex collective variables such as the *simple* base-pair parameters, we evaluated Equation 3 numerically by a finite-difference, as suggested by Darve *et al.* (18). Then, only values of collective variables and their gradients are necessary for evaluation of Equations 1, 3, and 4.

The free energy gradient provided by Equation 2 is a function of $\boldsymbol{\xi}^*$. Due to numerical reasons, this function is calculated for discrete values of $\boldsymbol{\xi}_b^*$ by averaging instantaneous collective force in small intervals centered at these discrete values:

$$\frac{\partial G(\xi_b^*)}{\partial \xi} = -\boldsymbol{f}(\xi_b^*) = -\langle \boldsymbol{F}_{ICF} \rangle_{\xi=\xi_b^* \pm \boldsymbol{h}/2} \quad (5)$$

$$\boldsymbol{\sigma}_{\boldsymbol{e}}\left(\frac{\partial G(\xi_b^*)}{\partial \xi}\right) = \boldsymbol{\sigma}_{\boldsymbol{e}}\left(\bar{\boldsymbol{f}}(\xi_b^*)\right) = \sqrt{\frac{N_{corr}}{N_b}}\,\boldsymbol{\sigma}(\boldsymbol{F}_{ICF})_{\xi=\xi_b^* \pm \boldsymbol{h}/2} \quad (6)$$

where $\boldsymbol{h}$ is the interval size. Through the text, we will call these intervals as bins. The standard error of the free energy derivative $\boldsymbol{\sigma}_{\boldsymbol{e}}$ can be estimated from the standard deviation of instantaneous collective force $\boldsymbol{\sigma}$, where N_b is the number of samples collected in a bin centered at $\boldsymbol{\xi}_b^*$, and N_{corr} is statistical inefficiency due to correlation in time series. The determination of statistical inefficiency will be described later.

Since the free energy gradient is unknown before the simulation, the simulation is started without any bias. In the course of the simulation, the free energy gradients are calculated by

Equations 5 and 3. Once enough samples are accumulated in bins, the free energy gradients are employed as bias (Equation 1). The bias is applied as soon as possible, using scaled forces to promote sampling further in the early stages of the simulation. In our work, we used a linear ramp:

$$\left.\frac{\partial G(\xi_b^*)}{\partial \xi}\right|_{applied} = \frac{\partial G(\xi_b^*)}{\partial \xi} \qquad N_b \geq N_{r,max} \tag{7}$$

$$\left.\frac{\partial G(\xi_b^*)}{\partial \xi}\right|_{applied} = \frac{\partial G(\xi_b^*)}{\partial \xi}\frac{N_b - N_{r,min}}{N_{r,max} - N_{r,min}} \qquad N_b \geq N_{r,min},\, N_b < N_{r,max} \tag{8}$$

$$\left.\frac{\partial G(\xi_b^*)}{\partial \xi}\right|_{applied} = 0 \qquad N_b < N_{r,min} \tag{9}$$

where $N_{r,max}$, and $N_{r,min}$ are maximum and minimum numbers of samples in bins for application of the scaling ramp, respectively. The setup of the linear ramp is provided in Table SA5.

Application of the bias flattens the potential energy surface and allows visiting other bins. With the increasing number of samples in each bin, the free energy gradients become more accurate, which promotes sampling further. Thus, the information about the free energy surface is improved adaptively in the course of the ABF simulation.

Multiple-walker Approach (MWA) was employed to further improve the sampling in the space of collective variables during the ABF simulations. The MWA follows the same strategy employed in Metadynamics simulations (20) and later also used in the other ABF implementation (21). In the PMFLib package, MWA employs a server/client architecture with a fully asynchronous communication pattern. The server collects the mean force (Equation 5) from individual clients (walkers), averages them with already stored mean force, and then sends updated mean force back to the clients. A client is a regular ABF molecular dynamics simulation. If the walkers are fully independent and run concurrently, then the mean force converges M-times faster than in a single ABF simulation, where M is the number of walkers.

In this work, the mean force was exchanged between the server and a client every 40 ps (20000 time-steps). Depending on the number of collective variables, we usually employed either 5 (for one CV) or 10 walkers (for two CVs), see Table SA5. To avoid coherency between walkers, each walker started from different configuration taken from the unbiased MD simulations as a restart file with a minimum time separation of at least 10 ns from the other restart files. Moreover, a stochastic Langevin thermostat with a low collision frequency of γ=0.1 ps^{-1} was employed in the course of ABF simulations to avoid spontaneous synchronizations of walkers. Besides, each walker was run in a 2 ns batches with generating a new seed for the pseudo-random generator during the restart of MD simulation. Change of seed numbers was required for the proper functionality of Langevin thermostat in frequently restarted MD runs (22), and it further improved stochastic behavior and avoided possible synchronizations of walkers. The reproducibility of this procedure was tested on selected systems, where repeating of ABF/MWA simulations provided the same free energy surfaces within the error predicted by GPR integration.

Opening and Shear as Collective Variables. The base-pair parameters (Shear, Stretch, Stagger, Buckle, Propeller, and Opening) are commonly used in the analysis of experimental structures and MD trajectories. Unfortunately, none of the available programs (Curves, 3DNA) provide gradients of base-pair parameters, which are necessary for the biased simulation. Therefore, we implemented these parameters together with their gradients[1] into PMFLib. Our implementation provides values of *simple* base-pair parameters fully compatible with the 3DNA implementation. While evaluation of these parameters requires very complex algebra primarily due to the superimposition of ideal nucleobases (reference frames) to the analyzed structure (23), our benchmarks revealed that only about one percent of the computational time is spent on their calculation employing the code implemented in Fortran running on CPU. Thus, our implementation makes *simple* base-pair parameters suitable for everyday use in biased MD simulations.

Table SA5. Setup of the ABF/MWA simulations.

							σ'			S'$_x$		
**Target**	**Classes**	**Systems**	**Tot len [ns]**	**# walkers (M)**	**$N_{r,min}$**	**$N_{r,max}$**	min [°]	max [°]	# bins	min [Å]	max [Å]	# bins
$\Delta G_r(\sigma',S'_x)$	I,II,III, C1, C2, H1, H2, H3	anti/anti	1000	10	1000	2000	-100	100	100	-8	8	160
		anti/syn	1000	10	1000	2000	-20	160	100	-8	6	160
		syn/anti	1000	10	1000	2000	-20	160	100	-6	8	160

							**φ$_1$**			**φ$_2$**		
**Target**	**Classes**	**Systems**	**Tot len [ns]**	**# walkers (M)**	**$N_{r,min}$**	**$N_{r,max}$**	min [°]	max [°]	# bins	min [°]	max [°]	# bins
$\Delta G_r(\phi_1,\phi_2)$	I	aG/aT	400	10	1000	2000	-50	30	80	-30	50	80

							**d_{N1N3}**			**d_{ring}**		
**Target**	**Classes**	**Systems**	**Tot len [ns]**	**# walkers (M)**	**$N_{r,min}$**	**$N_{r,max}$**	min [Å]	max [Å]	# bins	min [Å]	max [Å]	# bins
$\Delta G_r(d_{N1N3},d_{ring})$	I	aG/aT	400	10	1000	2000	-50	30	80	-30	50	80

							α		
**Target**	**Classes**	**Systems**	**Tot len [ns]**	**# walkers (M)**	**$N_{r,min}$**	**$N_{r,max}$**	min [°]	max [°]	# bins
$\Delta G_r(\alpha)$	I	selected	200	5	10000	20000	100	175	35

							δ		
**Target**	**Classes**	**Systems**	**Tot len [ns]**	**# walkers (M)**	**$N_{r,min}$**	**$N_{r,max}$**	min [Å]	max [Å]	# bins
$\Delta G_r(\delta)$	I	selected	200	5	10000	20000	1.4	6.0	46

							**S'$_x$**		
**Target**	**Classes**	**Systems**	**Tot len [ns]**	**# walkers (M)**	**$N_{r,min}$**	**$N_{r,max}$**	min [Å]	max [Å]	# bins
$\Delta G_r(S'_x)$	I	aG/aT	300	10	1000	2000	-3.0	5.0	80

[1] https://github.com/kulhanek/pmflib/blob/master/src/lib/fpmf/common/cvs/na/cv_nasbpp.f90

Wall Restraints. During all MD simulations, the dynamics of terminal base pairs was limited by wall-restraints (flat bottom harmonic potentials) imposed on hydrogen bonds between bases to avoid the formation of flanking bases that would influence the remaining DNA. We also restricted rotation around the *N*-glycosidic bonds of X/Y base pair by wall-restraints to keep systems in requested conformational space, which was otherwise altered by rare switches from less favorable *syn* to *anti* orientations (Table SA6).

During ABF/MWA simulations, some *simple* base-pair parameters were restricted by wall-restraints to keep the systems stable. The wall restraints ranges were derived from natural fluctuations of these parameters observed during 1 µs unbiased simulations and biased trial simulations. Their aim was to prevent irreversible destruction of the biased base pair and its surroundings, which was usually accompanied by the formation of alternative base pairing and base bulges (Table SA7).

Table SA6. Wall-restraints to keep terminal base pairs intact and prevent *anti*↔*syn* transitions on N-glycosidic bonds of the central base pair X/Y during all MD simulations.

Classes	Type	Ranges[a]				Force const.[b]	Atoms[c]	
		v_1	v_2	v_3	v_4			
I	H-bond	0	0	3	5	50	:1@O2	:26@H21
	H-bond	0	0	3	5	50	:1@N3	:26@H1
	H-bond	0	0	3	5	50	:1@H41	:26@O6
	H-bond	0	0	3	5	50	:13@H21	:14@O2
	H-bond	0	0	3	5	50	:13@H1	:14@N3
	H-bond	0	0	3	5	50	:13@O6	:14@H41
	χ, aX or aY	-240	-190	-50	0	50	:7[d] or :20[d]	
	χ, sX or sY	-70	-10	130	180	50	:7[d] or :20[d]	
II, III, C1, C2, H1, H2, H3	H-bond	0	0	3	5	50	:1@H21	:30@O2
	H-bond	0	0	3	5	50	:1@H1	:30@N3
	H-bond	0	0	3	5	50	:1@O6	:30@H41
	H-bond	0	0	3	5	50	:15@H21	:16@O2
	H-bond	0	0	3	5	50	:15@H1	:16@N3
	H-bond	0	0	3	5	50	:15@O6	:16@H41
	χ, aX or aY	-240	-190	-50	0	50	:8[d] or :23[d]	

a) harmonic restraints for $v < v_2$ and $v > v_3$; linear restraints for $v < v_1$ and $v > v_4$; distances in Å; angles in deg

b) either kcal mol^{-1} Å^{-2} or kcal mol^{-1} rad^{-2} depending on the restrained value

c) atoms in AMBER mask notation, see Figure SA1 for residue and atom numbering

d) purines χ(O4',C1',N9,C4) and pyrimidines χ(O4',C1',N1,C2)

Table SA7. Employed wall-restraints to keep systems in predefined configuration space during ABF/MWA simulations.

<table>
<tr><th rowspan="2">Classes</th><th rowspan="2">Systems</th><th rowspan="2">Type</th><th colspan="2">Ranges[a]</th><th rowspan="2">Force const.[b]</th><th rowspan="2" colspan="4">Atoms[c]</th></tr>
<tr><th>v_1</th><th>v_2</th></tr>
<tr><td rowspan="15">I</td><td>all</td><td>S'_y</td><td>-0.5</td><td>8</td><td>40</td><td colspan="4">:7/:20[d]</td></tr>
<tr><td>all</td><td>S'_z</td><td>-2</td><td>2</td><td>40</td><td colspan="4">:7/:20[d]</td></tr>
<tr><td>aX/sY, sX/aY</td><td>κ'</td><td>-50</td><td>50</td><td>0.1</td><td colspan="4">:7/:20[d]</td></tr>
<tr><td>aX/sY, sX/aY</td><td>π'</td><td>-50</td><td>50</td><td>0.1</td><td colspan="4">:7/:20[d]</td></tr>
<tr><td>aX/aY</td><td>S'_x</td><td>-8</td><td>8</td><td>40</td><td colspan="4">:7/:20[d]</td></tr>
<tr><td>aX/sY</td><td>S'_x</td><td>-8</td><td>6</td><td>40</td><td colspan="4">:7/:20[d]</td></tr>
<tr><td>sX/aY</td><td>S'_x</td><td>-6</td><td>8</td><td>40</td><td colspan="4">:7/:20[d]</td></tr>
<tr><td>aX/aY</td><td>σ'</td><td>-100</td><td>100</td><td>0.1</td><td colspan="4">:7/:20[d]</td></tr>
<tr><td>aX/sY, sX/aY</td><td>σ'</td><td>-20</td><td>160</td><td>0.1</td><td colspan="4">:7/:20[d]</td></tr>
<tr><td>aG/aT</td><td>φ_1</td><td>-50</td><td>30</td><td>0.1</td><td>:6,8,19,21[d]</td><td>:7@O3|:8[e]</td><td>:7[e]|:6@O3'</td><td>:7[f]</td></tr>
<tr><td>aG/aT</td><td>φ_2</td><td>-30</td><td>50</td><td>0.1</td><td>:6,8,19,21[d]</td><td>:20[e]|:19@O3</td><td>:20@O3'|:21</td><td>:20[f]</td></tr>
<tr><td>aG/aT</td><td>d_{N1N3}</td><td>2</td><td>9</td><td>50</td><td colspan="2">:7@N1</td><td colspan="2">:20@N3</td></tr>
<tr><td>aG/aT</td><td>d_{ring}</td><td>5</td><td>9</td><td>50</td><td colspan="2">:7[g]</td><td colspan="2">:20[g]</td></tr>
<tr><td>all(α)</td><td>α</td><td>100</td><td>170</td><td>0.1</td><td>:1-4,23-26 & (! @H=)</td><td colspan="2">:5-9,18-22 & (! @H=)</td><td>:10-17 & (! @H=)</td></tr>
<tr><td>all (δ)</td><td>δ</td><td>1.4</td><td>6</td><td>40</td><td colspan="4">:1-26@P,OP1,OP2,O3',O5',C3',C4',C5',C1',C2',O4' & (! @H=)</td></tr>
<tr><td rowspan="4">II, III, C1, C2, H1, H2, H3</td><td>all</td><td>S'_y</td><td>-0.5</td><td>8</td><td>40</td><td colspan="4">:8/:23[d]</td></tr>
<tr><td>all</td><td>S'_z</td><td>-2</td><td>2</td><td>40</td><td colspan="4">:8/:23[d]</td></tr>
<tr><td>all</td><td>S'_x</td><td>-8</td><td>8</td><td>40</td><td colspan="4">:8/:23[d]</td></tr>
<tr><td>all</td><td>σ'</td><td>-100</td><td>100</td><td>0.1</td><td colspan="4">:8/:23[d]</td></tr>
</table>

a) harmonic restraints for $v < v_1$ and $v > v_2$; distances in Å; angles in deg
b) either kcal mol^{-1} $Å^{-2}$ or kcal mol^{-1} deg^{-2} depending on the restrained value
c) atoms in AMBER mask notation, see Figure SA1 for residue and atom numbering
d) purines (@N1,C2,N3,C4,C5,C6,N7,C8,N9) and pyrimidines (@N1,C2,N3,C4,C5,C6)
e) @P,OP2,O5',OP1
f) purines (@C4,C5,N7,C8,N9) and pyrimidines (@N1,C2,N3,C4,C5,C6)
g) six-membered rings of purines and pyrimidines (@N1,C2,N3,C4,C5,C6)

Integration of Mean Forces by Gaussian Process Regression (GPR). ABF simulations do not provide free energy but mean forces (derivatives of the free energy). To get the free energies, mean forces need to be integrated. The integration is trivial for one collective variable, but it becomes challenging for two and more (*N*) collective variables. Since there are more data (*N*-times mean force) for each data point on the free energy, the problem is overdetermined. Therefore, the reconstruction of free energy usually employs Radial Basis Functions (RBF), whose weighs are found by least-square minimization (18). Another problem arises due to sampling. The required simulation time grows exponentially with the number of collective variables, but the available simulation time is subject to a balance between accuracy and available computational resources. As a result, mean forces can be determined with non-negligible errors if more the one collective variable is used. Consequently, usage of mean forces in RBF without considering their errors can lead to overfitting.

In this work, we employed Gaussian Process Regression, which puts the mean force errors into consideration. GPR is, therefore, less susceptible to overfitting than RBF. Here, we will provide an overview of the GPR method. Further details related to ABF simulations can be found in the original papers (24, 25). A general description of the Gaussian process (GP) in a broader context of machine learning can be found in ref (26).

The Gaussian process is formally defined as:

$$\Delta G(\boldsymbol{\xi}) \sim GP(m(\boldsymbol{\xi}), K(\boldsymbol{\xi}, \boldsymbol{\xi}')) \tag{10}$$

where $\Delta G(\boldsymbol{\xi})$ is the free energy as a function of collective variables $\boldsymbol{\xi}$**,** $m(\boldsymbol{\xi})$ is the mean function and $K(\boldsymbol{\xi}, \boldsymbol{\xi}')$ is the covariance function (kernel) of a real process. An interesting feature of GP is that the derivative of a Gaussian process is another Gaussian process. Thus, we can use a GP to make predictions of the free energies from their derivatives (mean forces).

The method consists of model training from mean forces (Equation 11) and the prediction of the free energies (Equation 12).

$$\boldsymbol{b} = (\boldsymbol{K}'' + \boldsymbol{\sigma}_e^2 \boldsymbol{I})^{-1} \boldsymbol{f} \tag{11}$$

$$G(\boldsymbol{\xi}^*) = -\boldsymbol{k}'(\boldsymbol{\xi}^*)^T \boldsymbol{b} \tag{12}$$

where $\boldsymbol{f}$ is a vector of mean forces (Equation 5) from all sampled bins, $\boldsymbol{\sigma}_e$ represents their errors (Equation 6), $\boldsymbol{I}$ is a unitary matrix, $\boldsymbol{b}$ is a vector with the model parameters, $\boldsymbol{\xi}^*$ is the collective variable position where we predict the free energy, and $\boldsymbol{K}''$and $\boldsymbol{k}'$ are a matrix and vector, respectively, obtained from the covariance function K by differentiation:

$$[\boldsymbol{K}'']_{ij} = \nabla_{\boldsymbol{\xi}} \nabla_{\boldsymbol{\xi}'} K(\boldsymbol{\xi}^{(i)}, \boldsymbol{\xi}'^{(j)}) \tag{13}$$

$$[\boldsymbol{k}'(\boldsymbol{\xi}^*)]_i = \nabla_{\boldsymbol{\xi}'} K(\boldsymbol{\xi}^*, \boldsymbol{\xi}'^{(i)}) \tag{14}$$

In both steps, we took the mean function to be zero. Since the free energy was reconstructed from derivatives, the information about additive constant (integration constant) was not known. Thus, obtained free energy surfaces were adjusted after prediction by shifting their global minima to zero.

Gaussian Process Kernels. The proper selection of a kernel (a covariance function) is essential because the kernel determines the quality of the free energy reconstruction. In this work, we tested the squared exponential kernel (Equation 15). This kernel is probably the most widely used kernel within the kernel machine fields. However, its strong smoothness assumption might be unrealistic for modeling real physical processes. Therefore, we also included the Matérn class kernel with v=5/2 (Equation 16). A variant with v=3/2 was discarded due to incompatibility with the second derivatives in $\boldsymbol{K}''$. Both kernels employed automatic relevance determination (ARD), which uses different scaling factors (characteristic length scales) for each collective variable (Equation 17). These length scales determine the relevancies of collective variables in the selected GP model.

$$K_{SE}(\boldsymbol{\xi},\boldsymbol{\xi}') = \sigma_G^2 \exp\left(-r^2\right) \tag{15}$$

$$K_{MC}(\boldsymbol{\xi},\boldsymbol{\xi}') = \sigma_G^2 \left(1 + \sqrt{5}r + \frac{5}{3}r^2\right) \exp\left(-\sqrt{5}r\right) \tag{16}$$

$$r = \sqrt{\sum_j \frac{(\xi_j - \xi_j')^2}{l_j^2}} \tag{17}$$

where r is scaled Euclidean distance between two positions $\boldsymbol{\xi}$ and $\boldsymbol{\xi}'$, l_j is the characteristic length scale of collective variable j, σ_G^2 is the expected variance of the reconstructed free energy surface.

Due to technical reasons, we did not work directly with the characteristic length scales, but instead, we employed unitless factors w_j and bin sizes (Equation 5) from ABF simulations:

$$l_j = w_j h_j \tag{18}$$

Optimization of GPR Hyperparameters. σ_G^2, w_j, and N_{corr} are called hyperparameters because they need to be known before training of the GP model. In this work, we derived hyperparameters by maximizing the marginal likelihood. This is a standard procedure employed in machine learning for estimation of the statistical significance of GP models (27). The logarithm of the marginal likelihood ($logP_{ML}$) is defined as:

$$logP_{ML}(\sigma_G^2, \boldsymbol{w}, N_{corr}) = -\frac{1}{2}\boldsymbol{f}^T(\boldsymbol{K}'' + \boldsymbol{\sigma}_e^2\boldsymbol{I})^{-1}\boldsymbol{f} - \frac{1}{2}log|\boldsymbol{K}'' + \boldsymbol{\sigma}_e^2\boldsymbol{I}| - \frac{n}{2}\log(2\pi) \quad (19)$$

where n is the number of training data.

We found that the $logP_{ML}$ function is very smooth for all hyperparameters. The smoothness of $logP_{ML}$ is demonstrated for the aG/aT system (class **I**) in Figure SA7. Further analysis revealed that σ_G^2 has minimal impact on $logP_{ML}$ probably due to the undetermined value of integration constant. Therefore, the hyperparameters except for σ_G^2 were searched by local optimization employing the L-BFGS algorithm (28) and analytic gradients (27) of $logP_{ML}$. In all these optimizations, the value of σ_G^2 was kept fixed at a value higher than the real variance of the reconstructed free energy $\sigma_{G,real}^2$ provided by

$$\sigma_{G,real}^2 = \sum_i^{sampled} \frac{1}{N}(G_i - \bar{G})^2 \quad (20)$$

where the summation runs over all sampled bins N, G_i is the predicted free energy and $\bar{G}$ is the average of the predicted free energy.

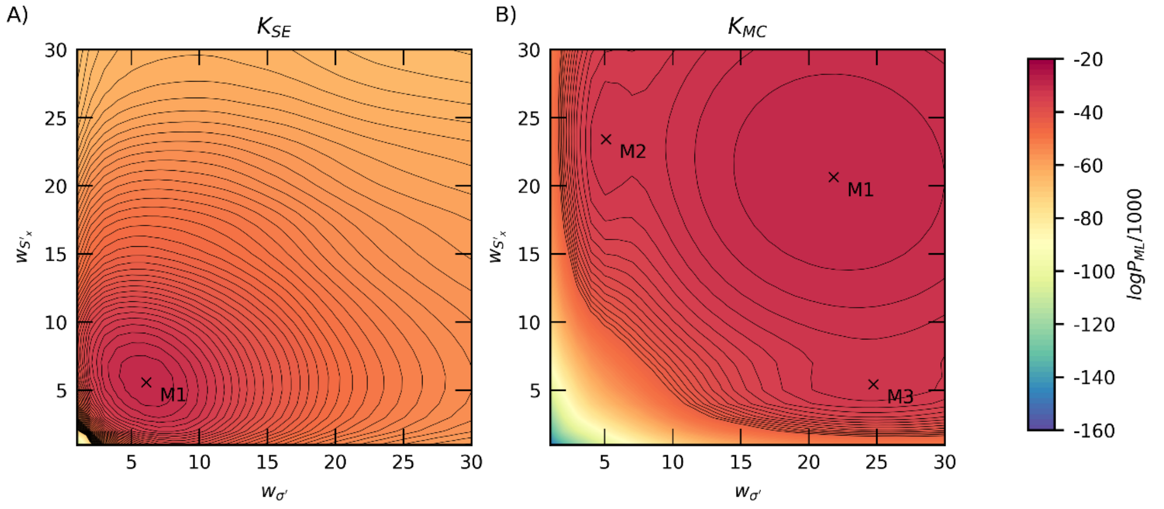

Figure SA7. Progress of $logP_{ML}(w_{\sigma'}, w_{S'_x})$ with $\sigma_G^2 = 15\,kcal^2mol^2$ and N_{corr} maximizing $logP_{ML}$ for (A) the squared exponential and (B) the Matérn class kernels employed for the reconstruction of $\Delta G_r(\sigma', S'_x)$ free energy surface of the aG/aT system (class **I**). Labeled crosses indicate found maxima of $logP_{ML}$ representing statistically significant GP models for the reconstruction of the free energy. Isolines are separated by 1000.

The obtained GP models were also evaluated in terms of mean force errors. The errors were calculated for each collective variable j independently using the input $\boldsymbol{f}$ and predicted $\boldsymbol{f_p}$ mean forces:

$$Err(f_j) = \sqrt{\sum_i^{sampled} \frac{1}{N}(f_{p,j,i} - f_{j,i})^2} \quad (21)$$

The predicted mean forces were obtained from trained GP model:

$$\boldsymbol{f}_p(\boldsymbol{\xi}^*) = \boldsymbol{k}''(\boldsymbol{\xi}^*)^T \boldsymbol{b} \tag{22}$$

where $\boldsymbol{k}''$ is defined as:

$$[\boldsymbol{k}''(\boldsymbol{\xi}^*)]_i = \nabla_{\boldsymbol{\xi}^*}\nabla_{\boldsymbol{\xi}'} K(\boldsymbol{\xi}^{*(i)}, \boldsymbol{\xi}'^{(i)}) \tag{23}$$

The optimized hyperparameters and performance of GP models are summarized in Tables SA8, SA9, SA10, SA11, SA12, and SA13.

First, we will describe all systems except $\Delta G_r(d_{N1N3}, d_{ring})$, which behaved differently. In the case of K_{SE}, we found only one statistically significant GP model M1 as the maximum on $logP_{ML}$. Since K_{MC} can describe less smooth GP better, two additional models M2, M3 appeared as maxima on $logP_{ML}$ (Figure SA7). In these models, the impact of one collective variable is more emphasized (it has a shorter characteristic length-scale than the other). A comparison shown in Figure SA8 revealed noticeable differences in the free energy surfaces reconstructed by the RBF method and the GPR method employing the M1, M2, and M3 GP models. However, the overall shape of reconstructed free energy surfaces is maintained in all methods and models.

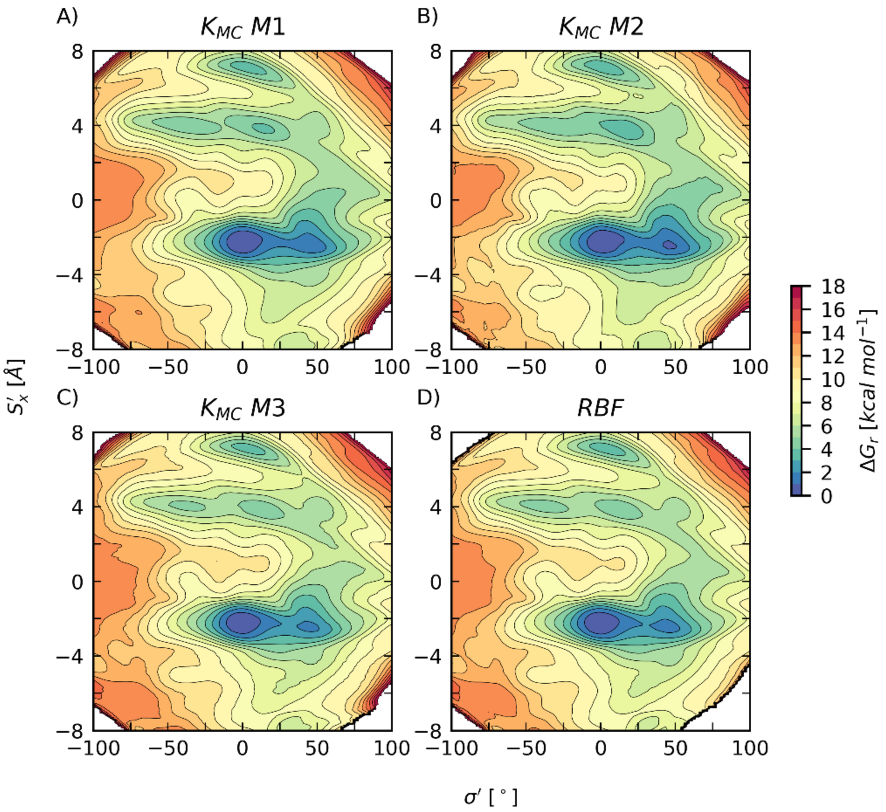

Figure SA8. Comparison of the free energy surfaces for the aG/aT system (class **I**) reconstructed by the RBF method and the GPR method employing the Matérn class kernel with the M1, M2, and M3 GP models.

For easier comparison of the free energy surfaces, we employed the reduced free energy profiles ΔG$_w$(S'$_x$), which were obtained by the statistical averaging from ΔG$_r$(σ', S'$_x$), see the Statistical averaging section for more details. These profiles were compared with very well converged ABF/MWA simulation ΔG$_r$(S'$_x$) employing only one collective variable. The average number of snapshots per bin in the ΔG$_r$(S'$_x$) ABF/MWA simulation was about 1 800 000, while it was only about 30 000 for the ΔG$_r$(σ', S'$_x$) ABF/MWA simulation. Thus, the mean forces obtained during ΔG$_r$(S'$_x$) were about eight times more accurate than in the case of ΔG$_r$(σ', S'$_x$).

We found that the GP model M3 and the RBF method overestimated the free energy profile, whereas the M2 model underestimated it. The GP model M1 provided nearly the same result as ΔG$_r$(S'$_x$) except for a small region near the transition state (Figure SA9). Nevertheless, the root-mean-squared difference between ΔG$_r$(S'$_x$) and ΔG$_w$(S'$_x$) K_{MC}/M1 was only 0.07 kcal mol^{-1}, which is within the confidence intervals.

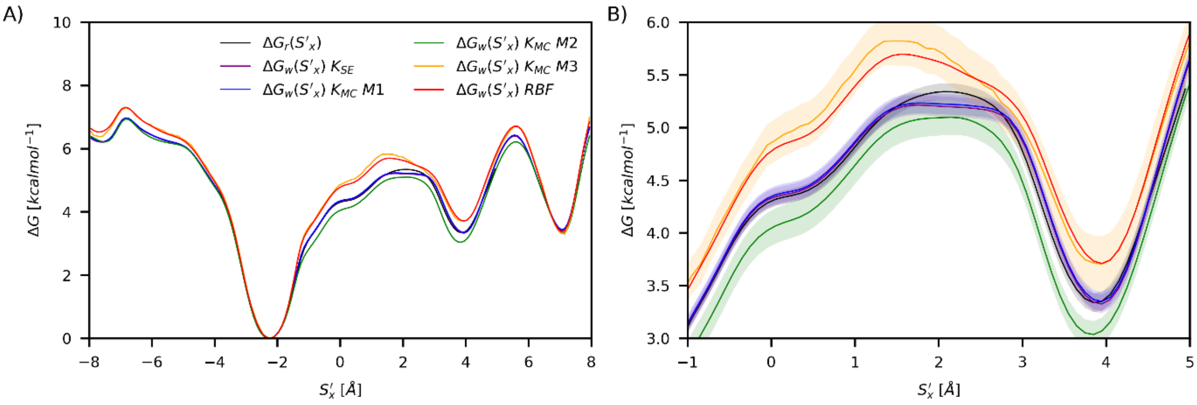

Figure SA9. Comparison of the free energy profiles from ABF/MWA simulations employing one $\Delta G_r(S'_x)$ and two collective variables $\Delta G_r(\sigma', S'_x)$, which was reduced by the statistical averaging into $\Delta G_w(S'_x)$. (A) the full range of S'$_x$. (B) zoomed range of S'$_x$ with confidence intervals shown at the three-sigma interval.

In general, the model M1 provided nearly the same reconstructed free energy surfaces regardless of the employed kernel, see average values of $\sigma^2_{G,real}$ in Tables SA8, SA10, SA11, SA12, and SA13. Since K_{MC} provided slightly higher values of $logP_{ML}$ (better statistical significance) and lower errors in the mean forces $Err(f_j)$ than K_{SE} , data obtained using the Matérn class kernel were employed for the final free energy reconstruction, if not stated otherwise.

In the case of ΔG$_r$(d$_{N1N3}$,d$_{ring}$), we found three GP models for each tested kernel (Table SA9). Since d$_{N1N3}$ and d$_{ring}$ are not suitable collective variables for the description of base-pair geometries, the interpretation of found GP models was difficult. In Figure 5D, we employed the M2 model and the K_{MC} kernel. This model showed a higher relevance of d$_{N1N3}$ over d$_{ring}$, and better matching of free energy minima to the thermodynamic states **1** and **2**.

Table SA8. Optimized GPR hyperparameters at $\sigma_G^2 = 15\ kcal^2 mol^2$ for the reconstruction of $\Delta G_r(\sigma', S'_x)$ and characterization of obtained GP models.

		K_{SE}							$K_{MC(v=5/2)}$						
CLASS	SYS	N_{corr}	$w_{\sigma'}$	$w_{S'x}$	$logP'_{ML}$ [a]	$Err(f_{\sigma'})$ [b]	$Err(f_{S'x})$ [c]	$\sigma^2_{G,real}$	N_{corr}	$w_{\sigma'}$	$w_{S'x}$	$logP'_{ML}$ [a]	$Err(f_{\sigma'})$ [b]	$Err(f_{S'x})$ [c]	$\sigma^2_{G,real}$
I	aA/aA	9.3	6.4	5.0	-31.4	1.03	0.40	5.31	9.1	25.0	19.6	-30.7	0.98	0.39	5.22
	aA/aC	10.8	6.1	6.0	-29.0	1.04	0.34	4.54	10.6	24.2	22.8	-28.4	0.98	0.32	4.39
	aA/aG	14.3	5.3	5.4	-40.3	1.25	0.52	6.89	14.6	17.1	18.0	-41.0	1.21	0.50	6.83
	aA:aT	11.0	5.6	5.4	-32.6	1.03	0.35	6.16	10.9	20.2	20.2	-32.8	0.99	0.34	6.15
	aC/aC	12.8	5.7	6.7	-25.5	0.89	0.31	4.88	12.5	23.7	26.4	-25.1	0.87	0.30	4.86
	aC/aT	11.3	6.7	7.3	-24.1	0.90	0.29	5.36	10.9	25.9	27.8	-24.0	0.86	0.28	5.30
	aG:aC	10.9	6.3	5.9	-31.3	1.11	0.36	7.67	10.6	22.3	23.0	-31.0	1.05	0.34	7.65
	aG/aG	15.0	4.7	4.9	-44.1	1.35	0.50	5.15	15.6	14.9	15.8	-45.3	1.28	0.49	5.15
	aG/aT	9.9	6.1	5.7	-30.7	1.09	0.36	8.51	9.9	21.3	21.0	-31.0	1.05	0.34	8.47
	aT/aT	13.9	7.2	6.1	-29.3	1.08	0.34	6.70	13.5	27.1	26.4	-28.6	1.04	0.33	6.65
	aA/sA	14.9	4.0	4.5	-35.4	1.24	0.47	6.67	14.4	18.4	17.4	-33.0	1.05	0.43	6.60
	aA/sC	22.9	6.2	6.0	-34.9	1.47	0.53	7.28	22.3	24.3	22.0	-34.4	1.38	0.49	7.33
	aA/sG	12.2	4.2	4.5	-34.0	1.23	0.44	4.57	11.4	18.7	16.9	-31.6	1.08	0.41	4.41
	aA/sT	14.0	4.1	4.7	-33.2	1.28	0.43	4.09	14.4	16.8	19.4	-32.5	1.22	0.42	4.09
	aC/sC	18.7	6.3	6.1	-26.1	1.19	0.37	7.90	18.1	25.4	26.2	-24.9	1.07	0.33	7.84
	aC/sT	15.4	6.4	5.8	-25.6	0.97	0.37	4.48	15.2	24.7	23.0	-25.9	0.95	0.36	4.59
	aG/sC	23.5	4.5	4.2	-39.7	1.41	0.48	8.86	22.9	18.8	16.4	-38.0	1.35	0.45	8.75
	aG/sG	17.2	4.0	3.8	-39.2	1.42	0.51	8.97	16.8	17.8	13.8	-37.2	1.32	0.48	8.98
	aG/sT	18.5	4.2	4.3	-38.1	1.47	0.49	8.29	17.3	17.0	16.8	-36.1	1.36	0.45	8.30
	aT/sT	17.9	3.6	5.0	-35.2	1.23	0.41	5.94	18.6	12.6	18.0	-36.0	1.17	0.39	6.01
	sA/aC	22.2	5.3	5.4	-35.3	1.34	0.47	10.88	21.7	19.2	20.3	-35.1	1.28	0.45	10.88
	sA/aG	13.4	3.7	4.2	-35.9	1.30	0.46	7.99	13.8	13.5	14.1	-37.0	1.19	0.43	8.02
	sA/aT	14.1	4.7	4.8	-29.3	1.12	0.38	11.34	14.5	19.4	19.0	-29.2	1.13	0.37	11.31
	sC/aT	12.3	6.5	5.9	-21.0	0.91	0.29	8.49	12.4	24.5	23.8	-21.8	0.90	0.28	8.64
	sG/aC	14.3	5.3	4.8	-30.6	1.24	0.41	6.29	13.7	19.9	19.1	-29.8	1.14	0.38	6.00
	sG/aT	22.7	5.5	5.4	-36.4	1.46	0.53	8.70	22.7	19.0	19.8	-36.6	1.42	0.51	8.64
II	aA:aT	9.8	6.2	6.1	-28.4	1.00	0.32	8.09	9.6	23.6	22.2	-28.5	0.96	0.31	8.09
	aG:aC	12.2	5.7	6.2	-31.3	1.11	0.39	9.48	12.0	21.3	22.4	-31.3	1.07	0.38	9.49
	aG/aT	13.4	5.7	5.9	-34.0	1.25	0.40	11.20	13.2	22.4	22.4	-33.3	1.19	0.38	11.07
III	aA:aT	10.8	5.98	5.39	-31.4	1.06	0.35	7.29	10.4	22.0	22.0	-30.8	1.01	0.33	7.28
	aG:aC	12.0	5.65	6.69	-31.9	1.10	0.39	9.75	11.8	21.7	24.5	-31.7	1.06	0.38	9.73
	aG/aT	12.9	5.86	6.15	-36.3	1.26	0.40	10.89	12.9	18.8	21.3	-36.9	1.21	0.38	10.90
C1	aA:aT	9.4	5.7	6.0	-28.8	1.02	0.32	6.86	9.3	21.0	21.3	-29.5	0.99	0.32	6.89
	aG:aC	12.6	5.7	5.7	-32.1	1.18	0.37	8.25	12.2	21.3	22.2	-31.6	1.14	0.35	8.24
	aG/aT	10.9	6.4	6.1	-31.8	1.15	0.37	8.91	10.6	23.8	23.0	-31.3	1.11	0.35	8.84
C2	aA:aT	9.7	6.4	6.1	-28.4	1.01	0.34	6.39	9.4	24.8	23.1	-28.1	0.96	0.33	6.37
	aG:aC	10.6	6.3	6.5	-30.0	1.02	0.35	8.11	10.3	23.3	24.2	-29.8	0.98	0.34	8.10
	aG/aT	12.3	7.1	6.3	-34.6	1.25	0.39	8.57	12.0	25.1	23.6	-34.3	1.21	0.38	8.51
H1	aA:aT	11.0	6.3	6.1	-31.6	1.11	0.36	7.82	10.7	22.6	23.0	-31.6	1.08	0.35	7.93
	aG:aC	16.4	5.4	6.0	-36.4	1.29	0.42	10.38	16.0	20.2	21.8	-36.0	1.23	0.41	10.38
	aG/aT	12.5	6.4	6.5	-33.5	1.19	0.41	10.25	12.4	24.0	24.1	-33.1	1.16	0.40	10.19
H2	aA:aT	9.9	6.0	6.1	-29.9	1.04	0.34	7.75	9.8	23.1	22.9	-30.0	1.01	0.33	7.79
	aG:aC	10.9	5.9	6.1	-29.9	1.04	0.36	9.24	10.5	22.1	22.8	-29.5	0.99	0.34	9.23
	aG/aT	11.5	5.9	5.9	-33.3	1.19	0.39	10.43	11.2	21.4	21.5	-33.0	1.13	0.37	10.33
H3	aA:aT	7.9	5.9	6.0	-26.3	0.94	0.31	6.97	7.7	22.8	22.6	-26.2	0.90	0.30	6.96
	aG:aC	10.7	6.1	6.0	-29.6	1.12	0.36	9.31	10.3	21.8	23.3	-29.3	1.07	0.35	9.27
	aG/aT	10.3	6.6	6.2	-30.9	1.07	0.36	10.03	10.0	24.3	23.5	-30.8	1.03	0.35	10.02
Average		13.4	5.7	5.7	-32.1	1.16	0.39	7.83	13.2	21.3	21.4	-31.8	1.10	0.38	7.80

a) $logP'_{ML} = logP_{ML}/1000$; b) kcal mol^{-1} deg^{-1}; c) kcal mol^{-1} Å^{-1}

Table SA9. Optimized GPR hyperparameters at $\sigma_G^2 = 36\ kcal^2 mol^2$ for the reconstruction of $\Delta G_r(d_{N1N3}, d_{ring})$ and characterization of obtained GP models.

			K_{SE}							$K_{MC(v=5/2)}$						
CLASS	SYS	GP Model	N_{corr}	w_{dN1N3}	w_{dring}	$logP_{ML}$	$Err(f_{dN1N3})$ [a]	$Err(f_{dring})$ [a]	$\sigma^2_{G,real}$	N_{corr}	w_{dN1N3}	w_{dring}	$logP_{ML}$	$Err(f_{dN1N3})$ [a]	$Err(f_{dring})$ [a]	$\sigma^2_{G,real}$
I	aG/aT	M1	26.2	2.8	0.8	-5888	0.27	0.40	12.68	1.0 [b]	9.0	3.2	-5313	0.01	0.01	13.23
		M2	253.8	2.9	2.6	-6223	1.18	1.92	18.08	9.0	3.8	8.1	-5816	0.05	0.17	18.20
		M3	72.7	0.8	2.8	-7269	0.50	0.70	17.91	183.1	7.0	6.7	-6226	0.93	1.66	17.78

a) kcal mol^{-1} Å^{-1}; b) N_{corr} was kept fixed at 1.0

Table SA10. Optimized GPR hyperparameters at $\sigma_G^2 = 15\ kcal^2 mol^2$ for the reconstruction of $\Delta G_r(\phi_1,\phi_2)$ and characterization of obtained GP models.

		K_{SE}							$K_{MC(v=5/2)}$						
CLASS	SYS	N_{corr}	$w_{\phi 1}$	$w_{\phi 2}$	$logP_{ML}$	$Err(f_{\phi 1})^a$	$Err(f_{\phi 2})^a$	$\sigma^2_{G,real}$	N_{corr}	$w_{\phi 1}$	$w_{\phi 2}$	$logP_{ML}$	$Err(f_{\phi 1})^a$	$Err(f_{\phi 2})^a$	$\sigma^2_{G,real}$
I	aG/aT	16.5	7.6	5.3	-33113	2.68	3.38	10.54	15.7	26.6	19.2	-32697	2.59	3.23	10.58

a) kcal mol^{-1} deg^{-1}

Table SA11. Optimized GPR hyperparameters at $\sigma_G^2 = 60\ kcal^2 mol^2$ for the reconstruction of $\Delta G_r(\alpha)$ and characterization of obtained GP models.

		K_{SE}					$K_{MC(v=5/2)}$				
CLASS	SYS	N_{corr}	w_α	$logP_{ML}$	$Err(f_\alpha)^a$	$\sigma^2_{G,real}$	N_{corr}	w_α	$logP_{ML}$	$Err(f_\alpha)^a$	$\sigma^2_{G,real}$
I	aA:aT	2.7	17.7	-48.2	0.54	17.11	2.6	44.0	-50.9	0.49	17.10
	aG:aC	4.1	19.4	-54.0	0.68	15.69	2.3	35.7	-53.5	0.44	15.62
	aG/aT	4.8	20.9	-55.3	0.73	14.47	3.1	33.9	-57.5	0.50	14.45
	aA/sA	4.4	13.0	-59.0	0.66	14.64	3.9	35.7	-59.0	0.56	14.63
	aG/sG	3.3	14.0	-53.6	0.56	16.04	3.2	36.9	-55.8	0.50	16.03
	sA/aC	4.6	22.7	-54.1	0.72	22.61	3.5	34.8	-58.5	0.56	22.61
	aA/aC	3.8	13.7	-55.7	0.66	11.78	3.1	37.2	-54.8	0.56	11.79
**Average**		4.0	17.4	-54.3	0.65	16.05	3.1	36.9	-55.7	0.52	16.03

a) kcal mol^{-1} deg^{-1}

Table SA12. Optimized GPR hyperparameters at $\sigma_G^2 = 60\ kcal^2 mol^2$ for the reconstruction of $\Delta G_r(\delta)$ and characterization of obtained GP models.

		K_{SE}					$K_{MC(v=5/2)}$				
CLASS	SYS	N_{corr}	w_δ	$logP_{ML}$	$Err(f_\delta)^a$	$\sigma^2_{G,real}$	N_{corr}	w_δ	$logP_{ML}$	$Err(f_\delta)^a$	$\sigma^2_{G,real}$
I	aA:aT	4.9	9.0	-35.5	0.19	18.80	3.3	23.8	-41.0	0.11	18.81
	aG:aC	7.2	12.1	-35.7	0.23	21.55	4.3	26.9	-38.3	0.14	21.58
	aG/aT	5.0	6.9	-52.1	0.20	26.42	2.6	21.4	-50.7	0.13	26.43
	aA/sA	33.7	16.7	-58.3	0.51	16.92	2.3	19.7	-49.4	0.08	16.87
	aG/sG	6.0	10.3	-36.7	0.22	20.46	4.6	23.2	-46.8	0.15	20.45
	sA/aC	3.9	7.1	-42.0	0.17	30.03	3.6	23.4	-44.6	0.13	30.03
	aA/aC	23.7	13.4	-57.1	0.41	12.24	2.0	21.9	-42.0	0.08	12.22
**Average**		12.1	10.8	-45.3	0.28	20.92	3.2	22.9	-44.7	0.12	20.91

a) kcal mol^{-1} deg^{-1}

Table SA13. Optimized GPR hyperparameters at $\sigma_G^2 = 10\ kcal^2 mol^2$ for the reconstruction of $\Delta G_r(S'_x)$ and performances of obtained GP models in terms of the mean force errors (Err) and variances of reconstructed free energy ($\sigma^2_{G,real}$).

		K_{SE}					$K_{MC(v=5/2)}$				
CLASS	SYS	N_{corr}	$w_{S'x}$	$logP_{ML}$	$Err(f_{S'x})^a$	$\sigma^2_{G,real}$	N_{corr}	$w_{S'x}$	$logP_{ML}$	$Err(f_{S'x})^a$	$\sigma^2_{G,real}$
I	aG/aT	10.2	5.3	28.1	0.035	2.838	1.0[b]	24.9	48.2	0.002	2.839

a) kcal mol^{-1} Å^{-1}; b) N_{corr} was kept fixed at 1.0

Error Analysis. In contrast to RBF, GPR provides a rigorous way for estimation of errors of the reconstructed free energies. Due to unknown integration constant, the errors σ_e must be determined relative to a specified point (24). In this work, this point was set to the position of the global minimum $\boldsymbol{\xi}^{\boldsymbol{g}}$.

$$\sigma_e^2\left(\Delta G(\boldsymbol{\xi}^*)\right) = var\left(G(\boldsymbol{\xi}^*) - G(\boldsymbol{\xi}^{\boldsymbol{g}})\right) \tag{24}$$

$$var\left(G(\boldsymbol{\xi}^*) - G(\boldsymbol{\xi}^{\boldsymbol{g}})\right) = var\left(G(\boldsymbol{\xi}^*)\right) + var\left(G(\boldsymbol{\xi}^{\boldsymbol{g}})\right) - 2cov(G(\boldsymbol{\xi}^*), G(\boldsymbol{\xi}^{\boldsymbol{g}})) \tag{25}$$

with variances and covariances provided by GPR as:

$$cov\left(G(\boldsymbol{\xi}_1^*), G(\boldsymbol{\xi}_2^*)\right) = k(\boldsymbol{\xi}_1^*, \boldsymbol{\xi}_2^*) - \boldsymbol{k}'(\boldsymbol{\xi}_1^*)^T(\boldsymbol{K}'' + \boldsymbol{\sigma}_e^2\boldsymbol{I})^{-1}\boldsymbol{k}'(\boldsymbol{\xi}_2^*) \tag{26}$$

$$var\left(G(\boldsymbol{\xi}_1^*)\right) = cov\left(G(\boldsymbol{\xi}_1^*), G(\boldsymbol{\xi}_1^*)\right) \tag{27}$$

Errors of the free energy provided by GPR depends solely on the values of hyperparameters (see Equation 26). Therefore, the proper determination of their values is critical for the correct error estimate. Since our implementation of ABF did not store time progress of instantaneous collective forces, we were not able to determine N_{corr} directly from the simulations. Instead, we included N_{corr} into a set of hyperparameters as a single effective parameter, which is the same for all bins and collective variables.

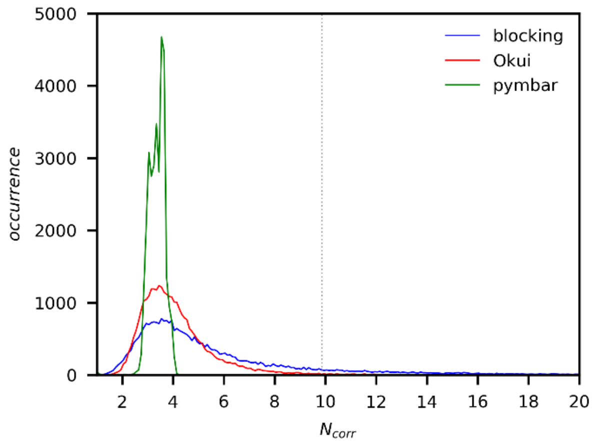

Figure SA10. Distribution of N_{corr} determined by the integrated autocorrelation time implemented in the pymbar package, the blocking method, and unbiased estimation of the variance of the sample mean (Okui). Gray dashed line is the value of N_{corr} determined by optimizing N_{corr} as a GPR hyperparameter.

Since the hyperparameters were found by maximizing the marginal likelihood of the reconstructed data, the obtained value of N_{corr} did not have to reflect real correlation in instantaneous collective forces. We tested the impact of this approximation by employing more appropriate approaches to N_{corr} determination. For this purpose, we re-calculated ABF/MWA simulation for the G/T mismatch and captured the time progress of instantaneous collective forces. N_{corr} was then determined from the integrated autocorrelation time implemented in the pymbar package (29), the blocking method (30), and unbiased estimation of the variance of the sample mean (31). We analyzed the progress of instantaneous collective forces in each bin, and for each collective variable. In total, 32 000 time-series were analyzed (Figure SA10).

All methods provided distributions with the highest occurrence at N_{corr} of about 3.5, while GPR optimized value was 9.9. This comparison indicates that N_{corr} determined by GPR is not

underestimated for most bins. Since GPR considers all input data, a higher value of N_{corr} might indicate some inconsistencies caused by the discretization of collective variables employed in determinations of mean forces (size of $\boldsymbol{h}$, see Equations 5). Meaning, smaller bins and more extended simulations would provide more accurate results. Nevertheless, the setup of ABF/MWA simulations employed in this study provided already sufficiently accurate data.

Statistical Averaging was employed for the reduction of calculated free energy surfaces (two dimensional) to free energy profiles (one dimensional). Here, we applied the statistical averaging for evaluation of the propensity for the opening by evaluating the definite integral: (32)

$$\Delta G_w(\sigma') = -RTln \int_{S'_{x,1}}^{S'_{x,2}} e^{-\frac{\Delta G_r(\sigma', S'_x)}{RT}} \mathrm{d}S'_x + C \quad (28)$$

where R is the universal gas constant, T is the absolute temperature (300 K), C is an arbitrary constant which was set to a value making $\Delta G_w(\sigma')$ zero at the global minimum. The value of normalization integral is included in C.

The integral was evaluated numerically using only properly sampled bins from ABF/MWA simulations:

$$\Delta G_w(\sigma'_n) = -RTln \sum_m^{sampled} e^{-\frac{\Delta G_r(\sigma'_n, S'_{x,m})}{RT}} + C' \quad (29)$$

where n and m are bin indexes from sampled space of collective variables.

We also attempted to determine the error of $\Delta G_w(\sigma'_n)$. Since GPR can provide all necessary covariances (Equation 26), method of moments was employed in error propagation in Equation 30 from uncertain data $\Delta G_r(\sigma'_n, S'_{x,m})$ to $\Delta G_w(\sigma'_n)$:

$$\sigma^2\big(\Delta G_w(\sigma'_n)\big) = \sum_l \sum_k \frac{\partial \Delta G_w(\sigma'_n)}{\partial S'_{x,k}} \frac{\partial \Delta G_w(\sigma'_n)}{\partial S'_{x,l}} cov\left(\Delta G_r\big(\sigma'_n, S'_{x,k}\big), \Delta G_r\big(\sigma'_n, S'_{x,l}\big)\right) \quad (30)$$

where l and k are bin indexes in an integrated slice of the free energy surface. Due to unfavorable ln/exp relations in Equation 29, the error estimate provided by Equation 30 can be underdetermined. To compensate for this possible deficiency, the reported confidence intervals in calculated free energies are provided at three standard deviations.

Final Remarks on the Reconstruction of the Free Energies. Hyperparameter optimization was performed in the abf-opthyprms program from the PMFLib. Type of found maxima on $logP_{ML}$ was determined by analyzing Hessian calculated numerically, which revealed all negative eigenvalues confirming stationary points as the local maxima. For systems described by one collective variable, all mean forces were considered. In the case of two collective variables, only properly sampled bins were considered. Properly sampled bins had to contain at least 5 000 snapshots, the resulting free energy should not be higher than 18 kcal mol^{-1} above the global minima. Moreover, apparent outliers in the input mean forces were removed, employing a z-test at the four-sigma level. The integration of the mean forces including the statistical averaging was performed in the abf-integrate program from PMFLib.

VI.ANALYSIS OF ABF/MWA SIMULATIONS

Analysis of Free Energy Minima. Minima on the free energy surface revealed thermodynamic states. For the characterization of these states, we employed geometries of DNA from ABF/MWA trajectory snapshots, which are in the proximity of the free energy minima. The proximity of each free energy minimum was specified by an ellipse centered at the local minima. The semi-major and semi-minor axes of ellipse followed the principal curvature of the minima calculated as eigenvectors of the free energy Hessian. The Hessian was calculated at the local minimum numerically from the free energy surface. Sizes of semi-major and semi-minor axes were then determined in such a way that the free energy at the vertex and co-vertex of the ellipse were 0.5RT above the local minima. The value of this free energy threshold was calculated for the same temperatures as used during ABF/MWA simulations and was ca 0.3 kcal mol^{-1} large. The outcome of the entire procedure is demonstrated for one selected system in Figure SA11. The majority of trajectory processing was done in the CATs package (33) connected with PMFLib. CATs provide tools for conditional filtering of the ABF/MWA trajectories by values of collective variables and calculated free energies.

Geometries representing states were employed in the hydrogen bond analysis by ccptraj and nucleic acid parameters provided by 3DNA. We used default thresholds for parameters of hydrogen bonds. All these data are summarized in Supplementary data – Part B.

We also attempted to perform analysis of specific interactions of DNA with water molecules and sodium cations. Unfortunately, the number of snapshots characterizing thermodynamic states was low (typically about hundreds of snapshots). Increasing the size of ellipses increased the number of snapshots, but it also blurred the density maps, and results were not conclusive.

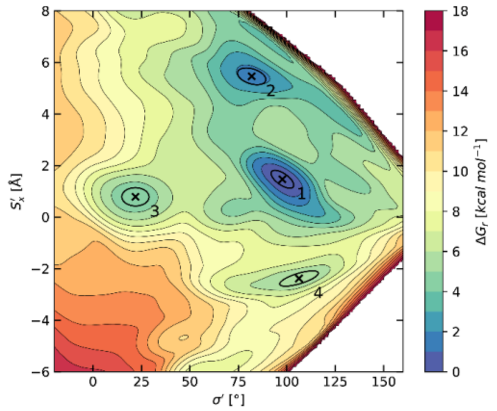

Figure SA11. Ellipses are encircling geometries in the proximity of the free energy minima shown for sA/aC (class **I**), which were employed in the characterization of thermodynamic states **1** to **4**. Free energy minima are labeled in ascending order. Free energy isolines are spaced by 1 kcal mol^{-1}.

Besides, structures representing thermodynamic states were created as average geometries from geometries in the close vicinity of the minima. The average structures were then post-processed by partial optimization. The aim was to optimize positions of hydrogen atoms while the rest of the structure was kept fixed by positional restraints with a force constant of 5 kcal mol^{-1} Å^{-2}.

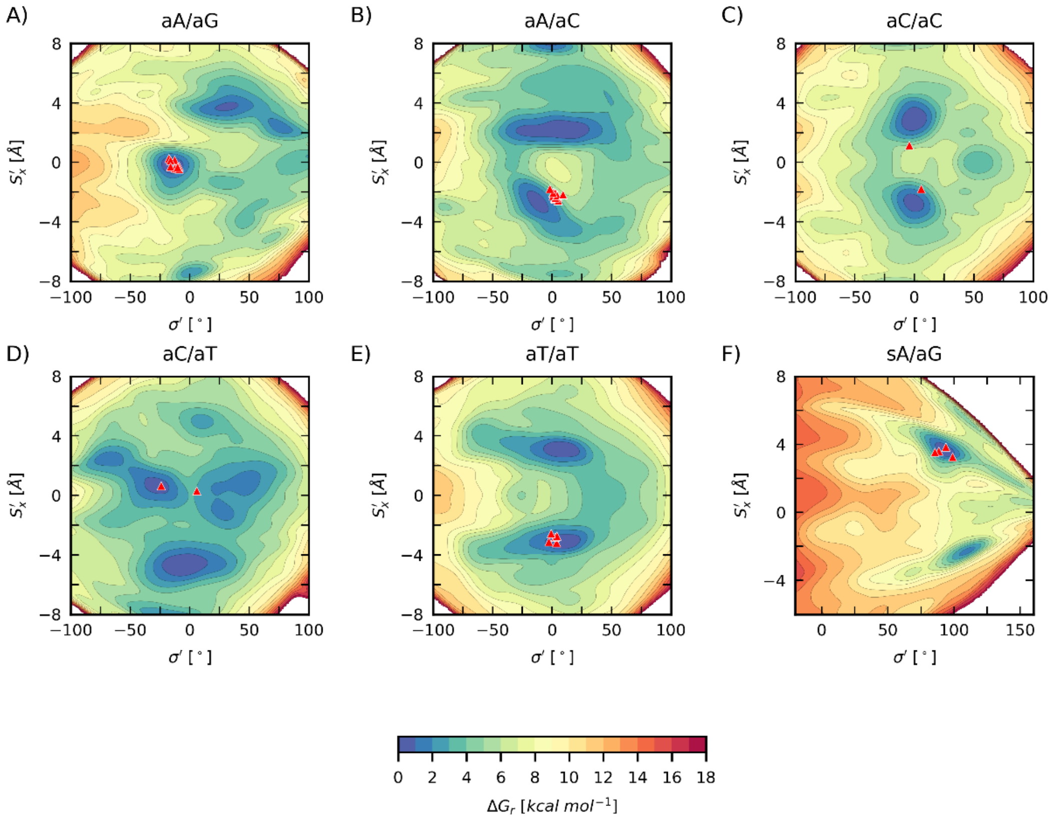

Figure SA12. Free energy surfaces for the sequence class **I** and their comparison with experimental geometries of mismatches in unbound DNAs (red triangles). Free energy isolines are spaced by 1 kcal mol^{-1}.

Base-pair Opening

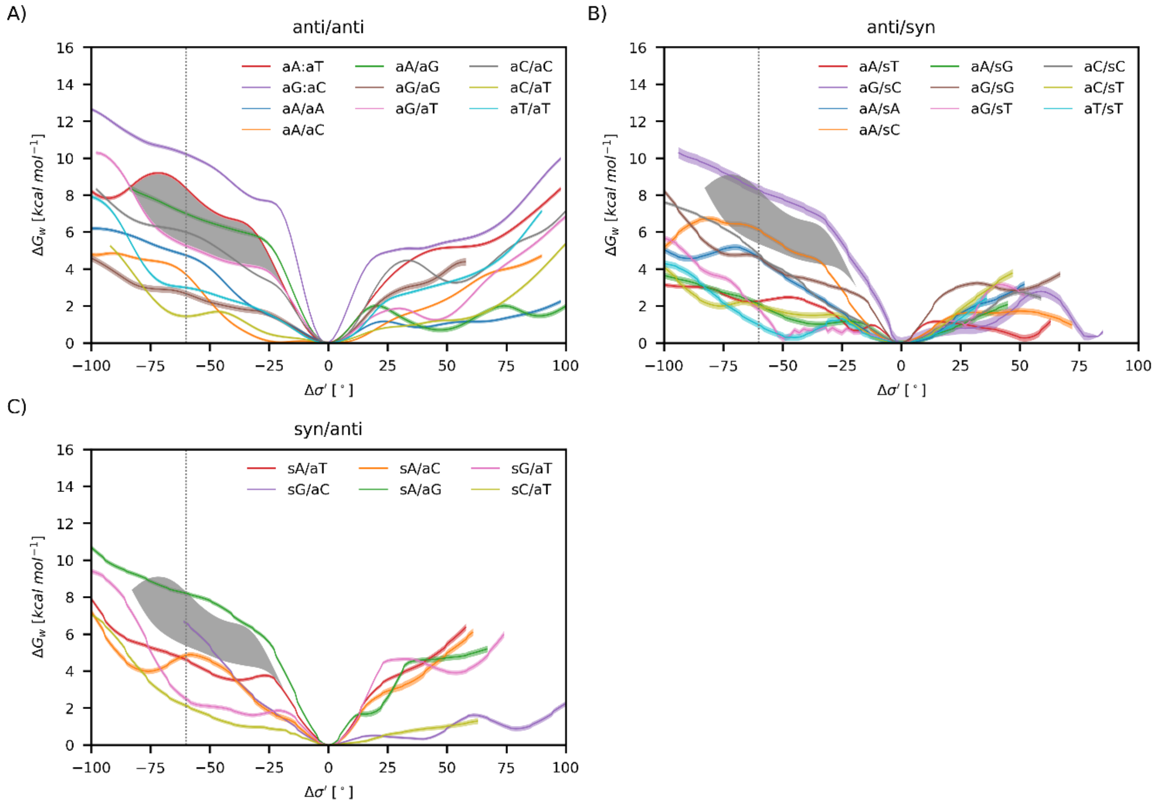

Figure SA13. The free energy cost for the opening of base pairs towards the minor ($\Delta\sigma' < 0$) and major ($\Delta\sigma' > 0$) grooves from the most stable thermodynamic state for the sequence class **I**. The vertical line at -60° corresponds to an average shift in Opening during recognition of best-repaired mismatches (aG/aT, aA/sA, aG/sG, and sA/aC). The gray strip corresponds to the discrimination gap, which is a gap between aA:aT and aG/aT.

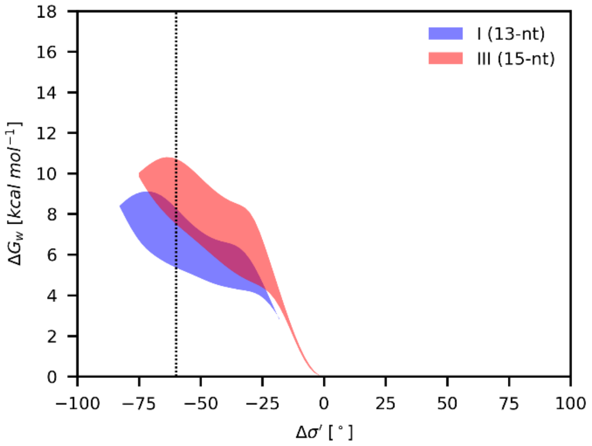

Figure SA14. Impact of DNA length on the recognition gap (aA:aT↔aG/aT) provided for the sequence classes **I** and **III**. The vertical line at -60° corresponds to an average shift in Opening during discrimination of best-repaired mismatches (aG/aT, aA/sA, aG/sG, and sA/aC).

DNA Bending Employing α. Table SA14 shows the stiffness constant K and equilibrium bending angle α_0 describing the DNA bending calculated within the limit of Hook's law:

$$\Delta G_r(\alpha) = \frac{1}{2}K(\alpha - \alpha_0)^2 \qquad (31)$$

Parameters K and α_0 were determined by non-linear regression in the interval from 130° to 170°. The regression, including error analysis, was done by the fit method implemented in gnuplot.

In comparison to the study of Sharma *et al.* (34), noticeable differences were found only for aA/sA and aG/sG, which are in our study more (by +1.0 kcal mol^{-1}) and less (by -1.9 kcal mol^{-1}) resilient to the bending (Table SA14).

Table SA14. Stiffness constants K and equilibrium bending angles α_0 for DNA bending. Comparison of propensities for the bending in this study and Sharma *et al*. (34).

	**K**			**α_0**			**Propensity for Bending [kcal mol^{-1}]**				
bp	**[kcal mol^{-1} rad^{-2}]**			**[°]**			**$\Delta G_r(\alpha=123°)^a$**			**$\langle\Delta G_r\rangle^b$**	**diff**
aA:aT	28.3	±	0.4	160.7	±	0.2	5.76	±	0.26	5.85	0.09
aG:aC	38.1	±	0.5	154.8	±	0.1	5.33	±	0.19	6.00	0.67
aG/aT	33.5	±	0.4	154.0	±	0.1	4.65	±	0.26	4.74	0.09
aA/sA	38.9	±	0.4	154.9	±	0.1	5.47	±	0.29	3.59	-1.88
aG/sG	36.0	±	0.2	154.3	±	0.1	5.13	±	0.25	6.15	1.02
sA/aC	46.0	±	1.0	157.6	±	0.2	7.43	±	0.31		
aA/aC	24.0	±	0.8	162.0	±	0.4	5.15	±	0.27	5.04	-0.11

a) this study; 13-nt long DNA, parmbsc1 force field, ABF/MWA biased simulations
b) Sharma *et al.*; 15-nt long DNA, CHARMM27 force field, umbrella sampling, average values in the range from 110° to 130°

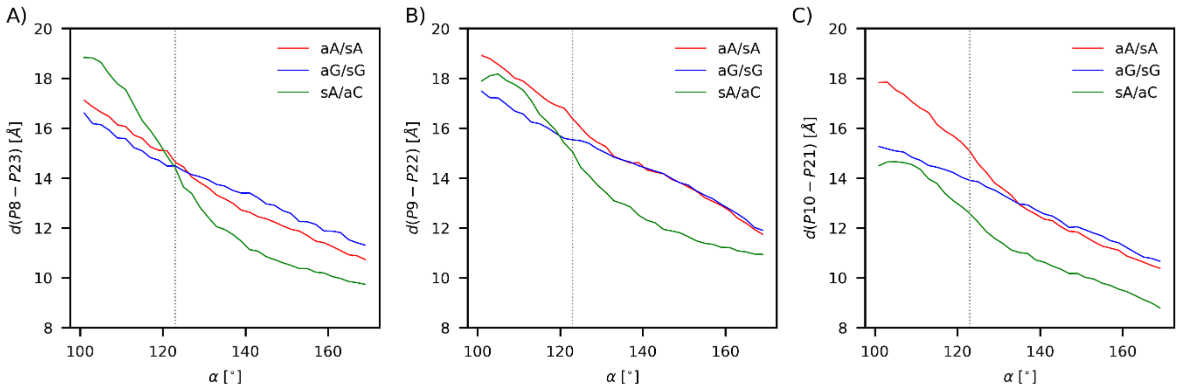

Figure SA15. Dependence of average minor groove widths expressed as distances between phosphorus atoms on the bending angle α (for numbering see Figure SA1). The threshold value of 123° shown as the dashed vertical line was taken from the experimental structure of MutSα/DNA (PDB ID: 2O8B).

Impact of Salt on DNA Bending. DNA is a heavily charged macromolecule with a negative charge uniformly distributed along the backbone. Previous studies showed that due to this feature and simplicity of force fields, which results in the various cancelation of errors, structural, dynamical, and mechanical characteristics of DNA are not too much influenced by the model of water solvent and type of ions (35, 36). However, different ionic strength affects cation–phosphate interactions. To test its influence on our models, we performed two additional simulations employing physiological NaCl concentration. We redid the DNA bending for aG:aC and aG/aT base pairs (Class I) and the α bending angle. The number of ions for c_0(NaCl)=154 mM was determined according to the work of Machado, M.R. *et al.* (37). They provided an accurate rule of thumb based on a recent thermodynamics method to define the electrolytic content in simulation boxes. The obtained results did not reveal any significant change within the error bars (Figure SA16).

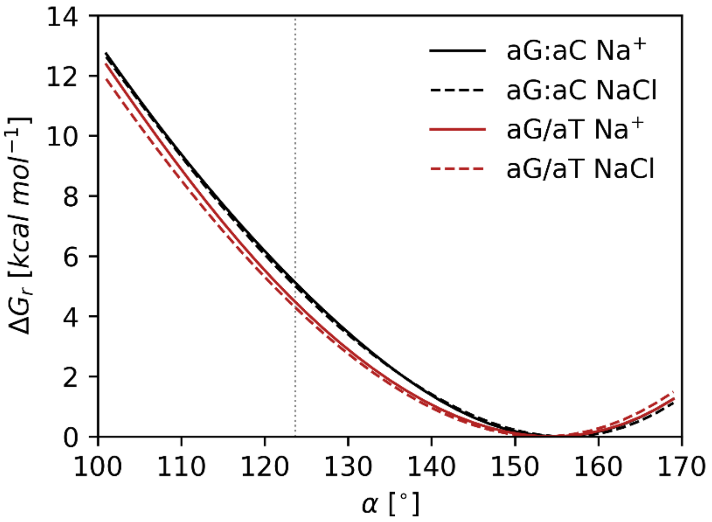

Figure SA16. Impact of counter ions on dsDNA bending of class **I** DNA with the central aG:aC (black) and aG/aT (red) base-pair for the net charge neutralization [solid line, c(DNA)=7 mM, number of ions $n(Na^+)$=24, real concentration $c(Na^+)$=173 mM] and physiological salt concentration (37) [dashed line, c(DNA)=7 mM, effective concentration c_0(NaCl)=154 mM, $n(Na^+)$=32, $n(Cl^-)$=8, $c(Na^+)$=231 mM, $c(Cl^-)$=58 mM]. Error bars are not shown for clarity, their magnitude is shown in Figure 7 and summarized in Table 2. The threshold value of 123° shown as the dotted vertical line was taken from the experimental structure of MutSα/DNA (PDB ID: 2O8B).

VII. REFERENCES

1. Lavery,R. and Sklenar,H. (1989) Defining the Structure of Irregular Nucleic-Acids - Conventions and Principles. *J. Biomol. Struct. Dyn.*, **6**, 655–667.

2. Lavery,R., Moakher,M., Maddocks,J.H., Petkeviciute,D. and Zakrzewska,K. (2009) Conformational Analysis of Nucleic Acids Revisited: Curves+. *Nucleic Acids Res.*, **37**, 5917–5929.

3. Lu,X.-J. and Olson,W.K. (2003) 3DNA: A Software Package for the Analysis, Rebuilding and Visualization of Three-Dimensional Nucleic Acid Structures. *Nucleic Acids Res.*, **31**, 5108–5121.

4. Lu,X.-J. and Olson,W.K. (2008) 3DNA: A Versatile, Integrated Software System for the Analysis, Rebuilding and Visualization of Three-Dimensional Nucleic-Acid Structures. *Nat. Protoc.*, **3**, 1213–1227.

5. Olson,W.K., Bansal,M., Burley,S.K., Dickerson,R.E., Gerstein,M., Harvey,S.C., Heinemann,U., Lu,X.-J., Neidle,S., Shakked,Z., *et al.* (2001) A Standard Reference Frame for the Description of Nucleic Acid Base-Pair Geometry. *J. Mol. Biol.*, **313**, 229–237.

6. Lu,X.-J. and Olson,W.K. (2016) Characterization of Base Pair Geometry. *Comput. Crystallogr. Newsl.*, **7**, 6–9.

7. Berman,H.M., Westbrook,J., Feng,Z., Gilliland,G., Bhat,T.N., Weissig,H., Shindyalov,I.N. and Bourne,P.E. (2000) The Protein Data Bank. *Nucleic Acids Res.*, **28**, 235–242.

8. Coimbatore Narayanan,B., Westbrook,J., Ghosh,S., Petrov,A.I., Sweeney,B., Zirbel,C.L., Leontis,N.B. and Berman,H.M. (2014) The Nucleic Acid Database: New Features and Capabilities. *Nucleic Acids Res.*, **42**, D114–D122.

9. Pan,F., Man,V.H., Roland,C. and Sagui,C. (2017) Structure and Dynamics of DNA and RNA Double Helices of CAG and GAC Trinucleotide Repeats. *Biophys. J.*, **113**, 19–36.

10. Case,D.A., Babin,V., Berryman,J.T., Betz,R.M., Cai,Q., Cerutti,D.S., Cheatham III,T.E., Darden,T.A., Duke,R.E., Gohlke,H., *et al.* (2016) AMBER 16 University of California, San Francisco.

11. Salomon-Ferrer,R., Götz,A.W., Poole,D., Le Grand,S. and Walker,R.C. (2013) Routine Microsecond Molecular Dynamics Simulations with AMBER on GPUs. 2. Explicit Solvent Particle Mesh Ewald. *J. Chem. Theory Comput.*, **9**, 3878–3888.

12. Ivani,I., Dans,P.D., Noy,A., Perez,A., Faustino,I., Hospital,A., Walther,J., Andrio,P., Goni,R., Balaceanu,A., *et al.* (2016) Parmbsc1: A Refined Force Field for DNA Simulations. *Nat. Methods*, **13**, 55-+.

13. Joung,I.S. and Cheatham,T.E. (2008) Determination of Alkali and Halide Monovalent Ion Parameters for Use in Explicitly Solvated Biomolecular Simulations. *J. Phys. Chem. B*, **112**, 9020–9041.

14. Jorgensen,W., Chandrasekhar,J., Madura,J., Impey,R. and Klein,M. (1983) Comparison of Simple Potential Functions for Simulating Liquid Water. *J. Chem. Phys.*, **79**, 935, 926.

15. Darden,T., York,D. and Pedersen,L. (1993) Particle Mesh Ewald - an N.log(N) Method for Ewald Sums in Large Systems. *J. Chem. Phys.*, **98**, 10089–10092.

16. Ryckaert,J., Ciccotti,G. and Berendsen,H. (1977) Numerical Integration of the Cartesian Equations of Motion of a System with Constraints: Molecular Dynamics of n-Alkanes. *J. Comput. Phys.*, **23**, 341, 327.

17. Comer,J., Gumbart,J.C., Hénin,J., Lelièvre,T., Pohorille,A. and Chipot,C. (2015) The Adaptive Biasing Force Method: Everything You Always Wanted To Know but Were Afraid To Ask. *J. Phys. Chem. B*, **119**, 1129–1151.

18. Darve,E., Rodríguez-Gómez,D. and Pohorille,A. (2008) Adaptive Biasing Force Method for Scalar and Vector Free Energy Calculations. *J. Chem. Phys.*, **128**, 144120.

19. Kulhánek, P., Bouchal,T., Durník,I., Štěpán, J., Fuxreiter, M., Mones, L., Petřek, M. and Střelcová, Z. (2018) PMFLib - A Toolkit for Free Energy Calculations Masaryk University, Brno.

20. Raiteri,P., Laio,A., Gervasio,F., Micheletti,C. and Parrinello,M. (2006) Efficient Reconstruction of Complex Free Energy Landscapes by Multiple Walkers Metadynamics. *J. Phys. Chem. B*, **110**, 3533–3539.

21. Minoukadeh,K., Chipot,C. and Lelièvre,T. (2010) Potential of Mean Force Calculations: A Multiple-Walker Adaptive Biasing Force Approach. *J. Chem. Theory Comput.*, **6**, 1008–1017.

22. Sindhikara,D.J., Kim,S., Voter,A.F. and Roitberg,A.E. (2009) Bad Seeds Sprout Perilous Dynamics: Stochastic Thermostat Induced Trajectory Synchronization in Biomolecules. *J. Chem. Theory Comput.*, **5**, 1624–1631.

23. Blanc,E. and Paciorek,W. (2001) On Planarity and Similarity Restraints. *J. Appl. Crystallogr.*, **34**, 480–483.

24. Stecher,T., Bernstein,N. and Csányi,G. (2014) Free Energy Surface Reconstruction from Umbrella Samples Using Gaussian Process Regression. *J. Chem. Theory Comput.*, **10**, 4079–4097.

25. Mones,L., Bernstein,N. and Csányi,G. (2016) Exploration, Sampling, And Reconstruction of Free Energy Surfaces with Gaussian Process Regression. *J. Chem. Theory Comput.*, **12**, 5100–5110.

26. Carl Edward Rasmussen and Christopher K. I. Williams Gaussian Processes for Machine Learning MIT Press.

27. Carl Edward Rasmussen and Christopher K. I. Williams Model Selection and Adaptation of Hyperparameters. In *Gaussian Processes for Machine Learning*, Adaptive Computation and Machine Learning. MIT Press, Vol. 2006.

28. Liu,D. and Nocedal,J. (1989) On the Limited Memory BFGS Method for Large-Scale Optimization. *Math. Program.*, **45**, 503–528.

29. Shirts,M.R. and Chodera,J.D. (2008) Statistically Optimal Analysis of Samples from Multiple Equilibrium States. *J. Chem. Phys.*, **129**, 124105.

30. Flyvbjerg,H. and Petersen,H.G. (1989) Error Estimates on Averages of Correlated Data. *J. Chem. Phys.*, **91**, 461–466.

31. Okui,R. (2010) Asymptotically Unbiased Estimation of Autocovariances and Autocorrelations with Long Panel Data. *Econom. Theory*, **26**, 1263–1304.

32. Chipot,C. and Pohorille,A. (2007) Free Energy Calculations Theory and Applications in Chemistry and Biology Springer, Berlin.

33. Kulhánek,P., Štěpán,J., Oľha,J., Růžička,M. and Illík,V. (2016) CATs - Conversion and Analysis Tools Masaryk University, Brno.

34. Sharma,M., Predeus,A.V., Mukherjee,S. and Feig,M. (2013) DNA Bending Propensity in the Presence of Base Mismatches: Implications for DNA Repair. *J. Phys. Chem. B*, **117**, 6194–6205.

35. Noy,A., Soteras,I., Luque,F. and Orozco,M. (2009) The Impact of Monovalent Ion Force Field Model in Nucleic Acids Simulations. *Phys. Chem. Chem. Phys.*, **11**, 10596–10607.

36. Dans,P.D., Danilāne,L., Ivani,I., Dršata,T., Lankaš,F., Hospital,A., Walther,J., Pujagut,R.I., Battistini,F., Gelpí,J.L., *et al.* (2016) Long-timescale dynamics of the Drew–Dickerson dodecamer. *Nucleic Acids Res.*, **44**, 4052–4066.

37. Machado,M.R. and Pantano,S. (2020) Split the Charge Difference in Two! A Rule of Thumb for Adding Proper Amounts of Ions in MD Simulations. *J. Chem. Theory Comput.*, **16**, 1367–1372.
