# Supplementary material for: Importance of base-pair opening for mismatch recognition
Source: Nucleic Acids Res. 2020 Oct 20;48(20):11322–34. doi: 10.1093/nar/gkaa896 (PMC7672436; doi:10.1093/nar/gkaa896)
Supplement: gkaa896_Supplemental_Files [file gkaa896_supplemental_files.zip › DNAMismatches006_SD_PartB.pdf]

Importance of Base-pair Opening for Mismatch Recognition

Tomas Bouchal,[†,‡] Ivo Durnik,[†,‡] Viktor Illik,[‡] Kamila Reblova,[†] and Petr Kulhanek[*,†,‡]

[†]) CEITEC - Central European Institute of Technology, Masaryk University, Kamenice 5, 625 00 Brno, Czech Republic
[‡]) National Centre for Biomolecular Research, Faculty of Science, Masaryk University, Kamenice 5, 625 00 Brno, Czech Republic
[*]) Corresponding author: e-mail: kulhanek@chemi.muni.cz; phone: +420 549 495 459; ORCID: 0000-0002-4152-6514

Supplementary data - part B

Contents

1 Free Energy Surface Overview **3**
1.1 *anti/anti* Orientations . . . 4
1.2 *anti/syn* Orientations . . . 5
1.3 *syn/anti* Orientations . . . 6

2 Base Pairs in the *anti/anti* Orientations **7**
2.1 aA/aA . . . 8
2.2 aA/aC . . . 13
2.3 aA/aG . . . 18
2.4 aA:aT . . . 23
2.5 aG:aC . . . 28
2.6 aG/aG . . . 33
2.7 aG/aT . . . 38
2.8 aC/aC . . . 43
2.9 aC/aT . . . 48
2.10 aT/aT . . . 53

3 Base Pairs in the *anti/syn* Orientations **58**
3.1 aA/sA . . . 59
3.2 aA/sC . . . 64
3.3 aA/sG . . . 69
3.4 aA/sT . . . 74
3.5 aG/sC . . . 79
3.6 aG/sG . . . 84
3.7 aG/sT . . . 89
3.8 aC/sC . . . 94
3.9 aC/sT . . . 99
3.10 aT/sT . . . 104

4 Base Pairs in the *syn/anti* Orientations **109**
4.1 sA/aC . . . 110
4.2 sA/aG . . . 115
4.3 sA/aT . . . 120
4.4 sG/aC . . . 125
4.5 sG/aT . . . 130
4.6 sC/aT . . . 135

1 Free Energy Surface Overview

1.1 *anti/anti* Orientations

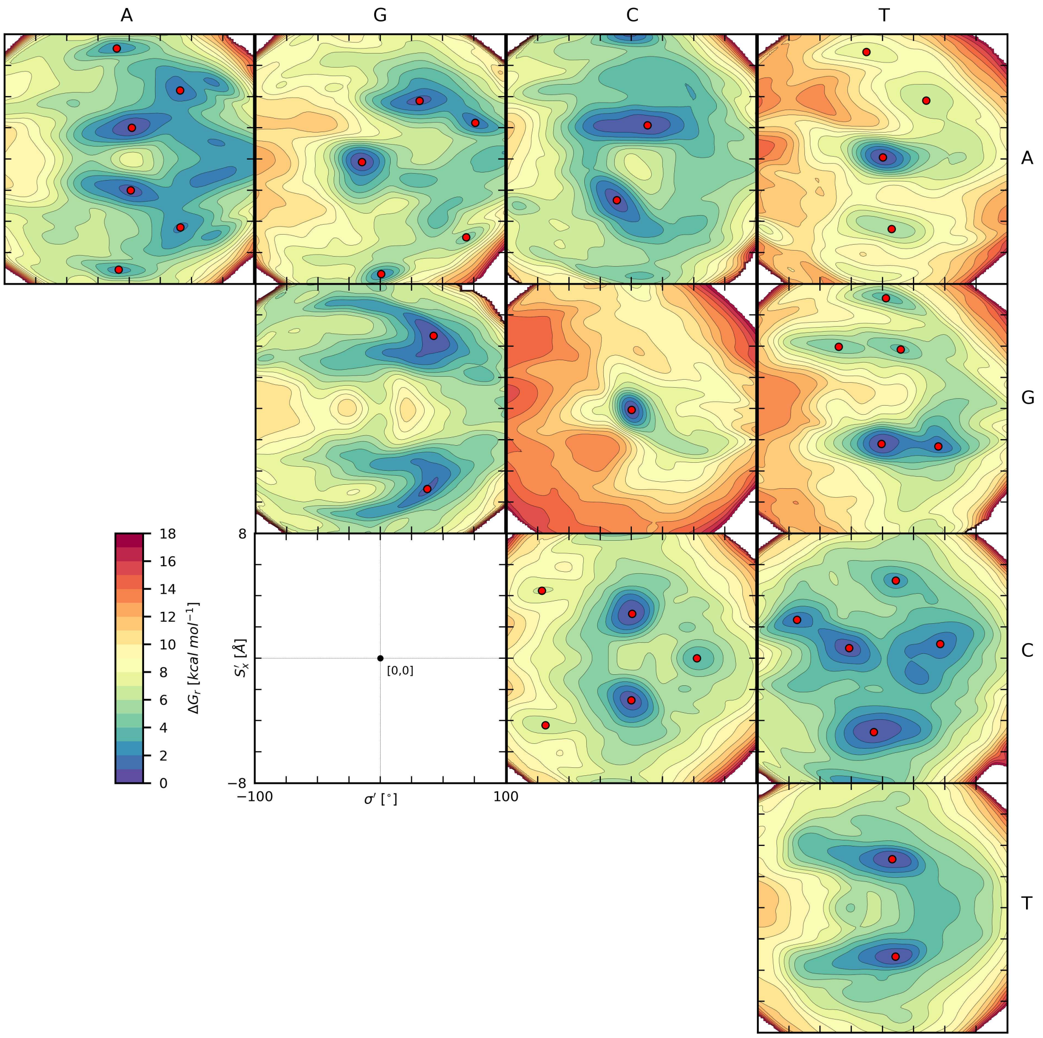

Figure SB0a: Free energy surfaces for the *anti/anti* mismatches and canonical base pairs. Selected free energy minima (thermodynamic states) are highlighted as red dots. Free energy isolines are spaced by 1 kcal mol^{-1}.

1.2 *anti/syn* Orientations

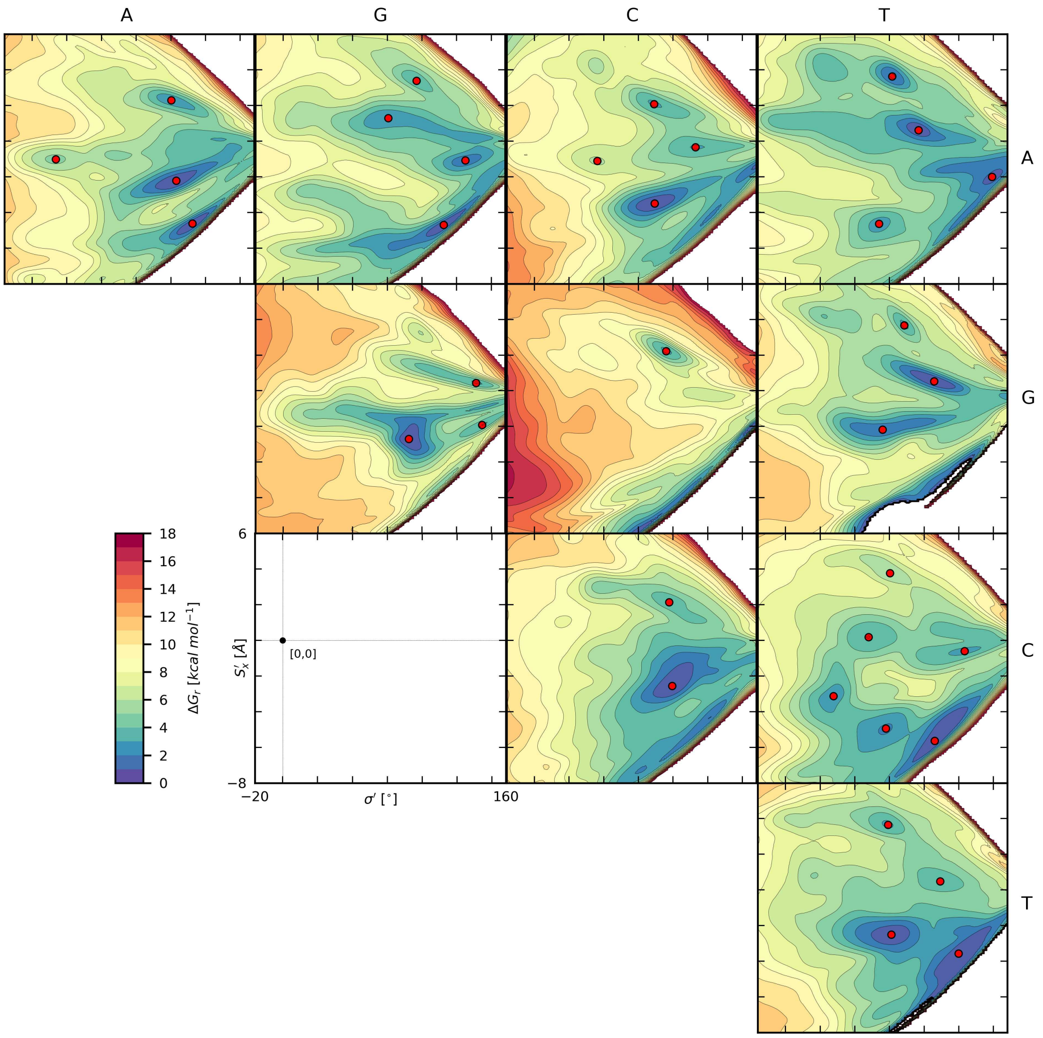

Figure SB0b: Free energy surfaces for the *anti/syn* mismatches. Selected free energy minima (thermodynamic states) are highlighted as red dots. Free energy isolines are spaced by 1 kcal mol^{-1}.

1.3 *syn/anti* Orientations

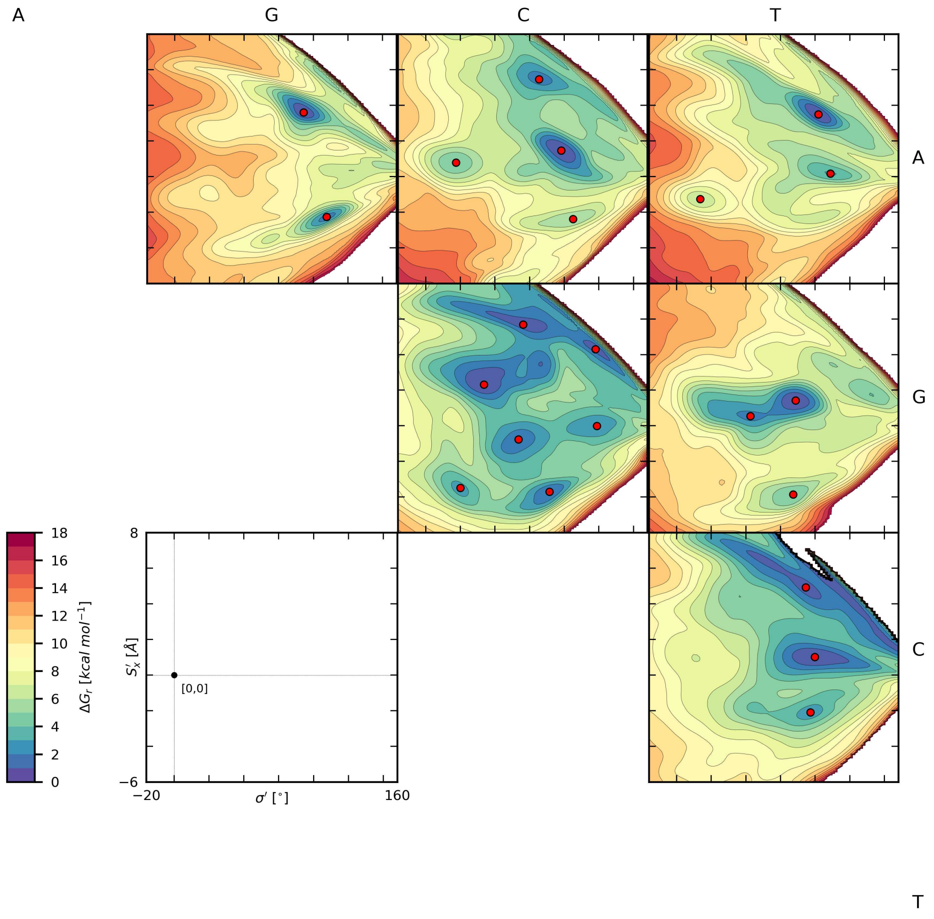

Figure SB0c: Free energy surfaces for the *syn/anti* mismatches. Selected free energy minima (thermodynamic states) are highlighted as red dots. Free energy isolines are spaced by 1 kcal mol^{-1}.

2 Base Pairs in the *anti/anti* Orientations

2.1 aA/aA

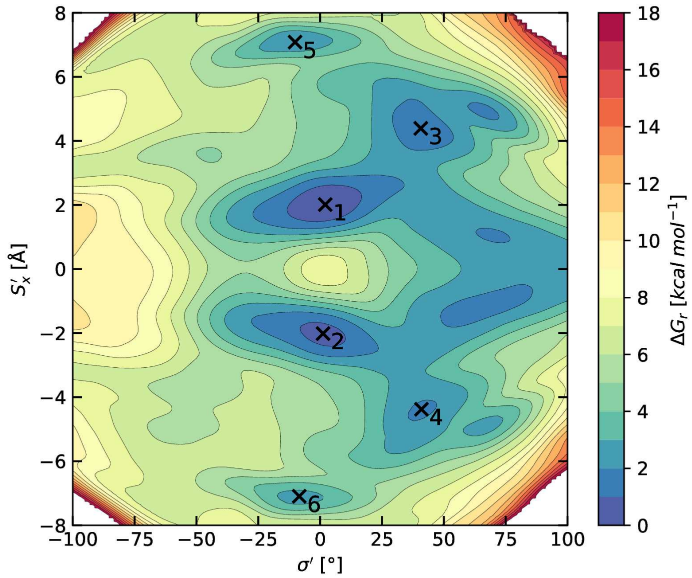

Figure SB1.1: Free energy surface for the aA/aA base pair. Labeled crosses show position of selected free energy minima (thermodynamic states). Free energy isolines are spaced by 1 kcal mol^{-1}.

Table SB1.1: Positions of selected free minima on the free energy surface for the aA/aA base pair. IDs correspond to the selected free energy minima shown in Figure SB1.1. Confidence interval of the free energy ΔG_r is provided at three standard deviations.

ID	σ' [°]	S'_x [Å]	ΔG_r [$kcal\ mol^{-1}$]
1	2.1	2.01	0.00±0.00
2	1.2	-2.01	0.61±0.10
3	40.7	4.39	1.35±0.11
4	40.9	-4.38	1.87±0.12
5	-10.1	7.08	2.11±0.12
6	-8.4	-7.09	2.61±0.13

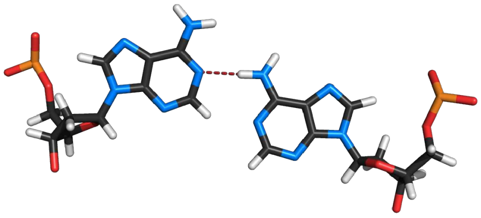

ID 1: $\sigma' = 2.1°$, $S'_x = 2.01$ Å

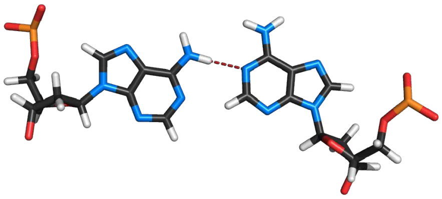

ID 2: $\sigma' = 1.2°$, $S'_x =$ -2.01 Å

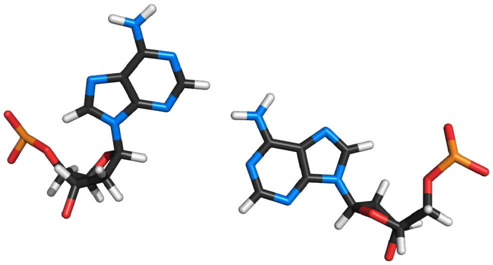

ID 3: $\sigma' = 40.7°$, $S'_x = 4.39$ Å

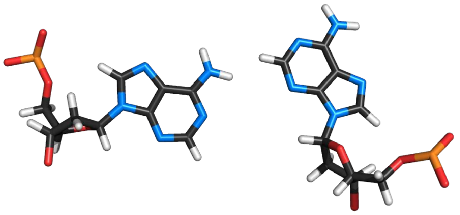

ID 4: $\sigma' = 40.9°$, $S'_x =$ -4.38 Å

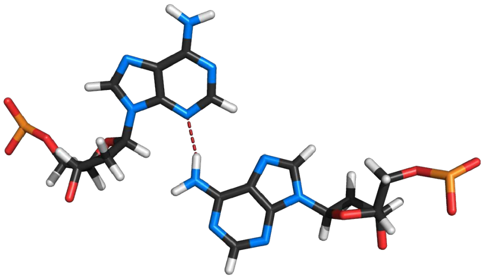

ID 5: $\sigma' =$ -10.1°, $S'_x = 7.08$ Å

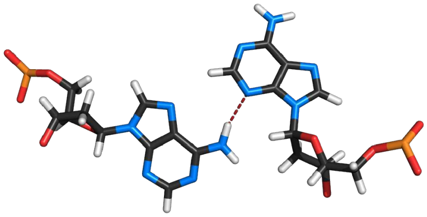

ID 6: $\sigma' =$ -8.4°, $S'_x =$ -7.09 Å

Figure SB1.2: Average geometries representing selected free energy minima for the aA/aA base pair. IDs correspond to the selected free energy minima shown in Figure SB1.1. Major and minor grooves are top and bottom, respectively. View direction is along the z-axis of DNA.

Table SB1.2: Number of analysed snapshots (N_{snap}), abundances (abu), average hydrogen bond distances and angles from hydrogen bond analysis provided by cpptraj for selected states of the aA/aA base pair. IDs correspond to the selected free energy minima shown in Figure SB1.1. Residue and atom numbering are provided in Figure SA1.

ID	N_{snap}	acceptor	H-donor	donor	abu [%]	d_{avg} [Å]	a_{avg} [°]
1	256	A7@N1	A20@H61	A20@N6	44.9	2.9	164.4
		A7@N1	A20@H62	A20@N6	1.2	2.9	169.0
2	313	A20@N1	A7@H61	A7@N6	45.4	2.9	163.2
3	349	—	—	—	—	—	—
4	410	—	—	—	—	—	—
5	181	A7@N3	A20@H62	A20@N6	58.6	2.9	156.2
6	245	A20@N3	A7@H62	A7@N6	52.2	2.9	156.1

Table SB1.3: *Simple* base-pair parameters for selected states of the aA/aA base pair. IDs corresponds to the free energy minima shown in Figure SB1.1. Residue numbering in base pairs A/B are provided in Figure SA1. Abundances (*abu*), average values ($\langle X \rangle$) and standard deviations of samples ($s(X)$) are calculated by 3DNA for Shear (S_x), Stretch (S_y), Stagger (S_z), Buckle (κ), Propeller (π), Opening (σ) employing the standard reference frames for the nucleobases. Only five central base pairs were included in the analysis for each state. Base pairs with a mismatch are highlighted in gray.

ID	A/B	abu [%]	$\langle S_x \rangle$	$s(S_x)$	$\langle S_y \rangle$ [Å]	$s(S_y)$	$\langle S_z \rangle$	$s(S_z)$	$\langle \kappa \rangle$	$s(\kappa)$	$\langle \pi \rangle$ [°]	$s(\pi)$	$\langle \sigma \rangle$	$s(\sigma)$
	5/22	100.0	-0.18	0.27	0.02	0.11	0.02	0.40	-0.6	10.2	-17.9	6.8	-0.5	4.8
	6/21	100.0	-0.13	0.25	0.03	0.11	-0.06	0.38	-8.1	9.8	-15.8	7.9	-0.4	5.4
1	7/20	100.0	2.03	0.16	2.07	0.17	-0.56	0.42	1.8	11.0	-9.9	8.9	2.5	3.4
	8/19	100.0	0.24	0.26	0.04	0.12	0.02	0.41	3.5	9.7	-13.2	7.9	-0.9	5.2
	9/18	100.0	0.23	0.27	0.04	0.12	0.05	0.36	-0.0	9.1	-18.2	7.1	-0.3	4.7
	5/22	100.0	-0.19	0.28	0.04	0.13	0.10	0.40	-1.7	8.8	-17.9	7.1	-0.1	5.6
	6/21	100.0	-0.21	0.27	0.05	0.12	0.04	0.42	-6.4	10.3	-12.9	8.2	-0.1	5.4
2	7/20	100.0	-2.01	0.16	2.03	0.17	-0.61	0.43	-3.5	11.9	-8.2	8.5	1.1	4.0
	8/19	100.0	0.12	0.27	0.04	0.14	-0.06	0.37	7.6	9.7	-14.8	8.4	-1.0	5.6
	9/18	100.0	0.20	0.26	0.02	0.11	-0.01	0.36	1.2	8.4	-19.2	6.8	-1.5	5.0
	5/22	100.0	-0.20	0.28	0.04	0.12	-0.04	0.39	1.0	8.7	-17.2	7.7	-0.0	4.9
	6/21	100.0	-0.19	0.28	0.03	0.13	-0.25	0.46	-5.7	10.7	-15.9	8.3	0.2	4.9
3	7/20	18.9	4.54	0.14	3.42	0.30	-0.57	0.87	2.8	11.5	-18.5	11.1	39.1	3.4
	8/19	100.0	0.13	0.28	0.03	0.11	-0.07	0.40	5.2	11.6	-10.4	8.9	2.3	5.1
	9/18	100.0	0.21	0.27	0.05	0.13	0.00	0.38	1.9	9.7	-18.1	7.2	-1.1	4.9
	5/22	100.0	-0.21	0.26	0.04	0.13	0.05	0.40	-2.8	9.5	-17.5	7.2	-0.7	4.9
	6/21	100.0	-0.14	0.26	0.02	0.12	-0.06	0.44	-6.1	10.5	-10.9	8.7	2.3	5.3
4	7/20	20.0	-4.57	0.17	3.40	0.26	-0.46	0.82	-3.6	13.8	-18.5	10.9	38.2	3.6
	8/19	100.0	0.17	0.27	0.03	0.13	-0.21	0.43	5.2	10.6	-16.5	8.4	-0.5	5.6
	9/18	100.0	0.19	0.25	0.03	0.11	-0.06	0.37	-1.2	8.5	-17.8	7.0	0.1	4.8
	5/22	100.0	-0.18	0.30	0.04	0.13	0.00	0.42	-3.2	11.1	-16.6	6.9	-1.0	5.2
	6/21	100.0	-0.14	0.28	0.01	0.13	-0.28	0.40	-7.7	9.8	-8.9	10.8	-0.9	5.0
5	7/20	100.0	7.09	0.09	2.14	0.26	-0.07	0.56	8.1	10.4	-10.6	15.4	-9.9	3.5
	8/19	100.0	-0.01	0.27	-0.01	0.14	-0.23	0.56	0.9	12.3	-11.5	9.7	2.2	5.2
	9/18	100.0	0.20	0.26	0.04	0.12	-0.19	0.38	-0.7	9.4	-14.6	7.6	-0.5	4.8
	5/22	100.0	-0.17	0.27	0.03	0.12	-0.11	0.41	1.1	10.0	-14.9	7.8	-0.1	5.2
	6/21	100.0	-0.06	0.29	-0.01	0.16	-0.24	0.60	-1.0	11.6	-12.0	9.9	3.5	6.2
6	7/20	100.0	-7.09	0.09	2.08	0.29	-0.36	0.51	-9.4	9.5	-19.5	14.1	-8.8	3.8
	8/19	100.0	0.14	0.26	0.03	0.13	-0.30	0.39	6.3	9.9	-13.2	9.1	-1.8	4.8
	9/18	100.0	0.19	0.25	0.03	0.12	-0.05	0.35	0.5	9.2	-18.2	7.5	-0.8	4.6

Table SB1.4: *Simple* step parameters for selected states of the aA/aA base pair. IDs corresponds to the free energy minima shown in Figure SB1.1. Residue numbering in base pairs and steps A1-B1/A2-B2 are provided in Figure SA1. Abundances (*abu*), average values ($\langle X \rangle$) and standard deviations of samples ($s(X)$) are calculated by 3DNA for Shift (D_x), Slide (D_y), Rise (D_z), Tilt (τ), Roll (ρ), Twist (ω) employing the standard reference frames for the nucleobases. Only five central base pairs were included in the analysis for each state. Steps including a mismatch are highlighted in gray.

ID	A1-B1 A2-B2	abu [%]	$\langle D_x \rangle$	$s(D_x)$	$\langle D_y \rangle$ [Å]	$s(D_y)$	$\langle D_z \rangle$	$s(D_z)$	$\langle \tau \rangle$	$s(\tau)$	$\langle \rho \rangle$ [°]	$s(\rho)$	$\langle \omega \rangle$	$s(\omega)$
	5-6/21-22	100.0	0.50	0.61	-0.36	0.59	3.42	0.28	3.2	4.0	1.3	5.1	38.8	4.2
1	6-7/20-21	100.0	-0.03	0.61	0.56	0.62	3.30	0.28	5.6	3.9	6.3	5.3	25.8	4.6
	7-8/19-20	100.0	0.95	0.68	-0.06	0.52	3.10	0.27	-1.1	5.2	4.9	7.3	31.8	5.7
	8-9/18-19	100.0	-0.58	0.52	-0.28	0.60	3.37	0.29	-2.3	4.0	0.1	5.4	38.3	4.6
	5-6/21-22	100.0	0.70	0.54	-0.19	0.51	3.37	0.30	3.4	3.9	0.8	5.9	38.5	4.2
2	6-7/20-21	100.0	-0.78	0.68	0.00	0.59	3.08	0.29	2.1	5.3	6.4	7.5	32.5	6.9
	7-8/19-20	100.0	0.17	0.62	0.60	0.57	3.26	0.25	-6.0	4.2	5.4	5.5	26.5	4.8
	8-9/18-19	100.0	-0.54	0.54	-0.34	0.52	3.41	0.28	-2.5	4.3	0.8	5.0	38.3	4.1
	5-6/21-22	100.0	0.50	0.49	-0.34	0.52	3.42	0.29	3.7	4.5	2.5	5.7	38.5	3.8
	6-7/20-21	18.9	0.95	0.58	1.58	0.59	3.64	0.35	7.2	5.7	5.3	5.5	23.3	4.0
3	6-8/19-21	80.2	0.24	0.77	-0.84	1.04	6.35	0.40	-0.5	6.4	20.3	9.3	52.6	8.6
	7-8/19-20	18.9	-0.91	0.63	-1.06	0.66	2.66	0.30	-5.2	6.6	18.2	8.9	31.8	7.8
	8-9/18-19	100.0	-0.87	0.57	0.03	0.44	3.35	0.27	-3.3	4.1	0.1	5.4	37.8	4.3
	5-6/21-22	100.0	0.88	0.58	0.03	0.44	3.35	0.27	3.8	3.9	-0.1	5.4	38.3	3.8
	6-7/20-21	20.0	0.95	0.68	-1.08	0.69	2.68	0.29	4.5	7.0	18.6	9.5	30.5	7.8
4	6-8/19-21	79.3	-0.21	0.77	-0.86	1.03	6.35	0.40	0.6	6.3	20.1	8.2	52.0	8.0
	7-8/19-20	20.0	-0.91	0.57	1.33	0.54	3.64	0.32	-5.9	4.9	3.8	4.8	23.4	4.6
	8-9/18-19	100.0	-0.39	0.52	-0.42	0.49	3.41	0.29	-3.0	4.2	2.7	5.4	38.3	3.9
	5-6/21-22	100.0	0.51	0.58	-0.28	0.55	3.41	0.30	3.9	3.9	1.3	5.2	37.3	4.0
5	6-7/20-21	100.0	-0.75	0.52	0.37	0.38	3.50	0.47	-2.0	4.0	4.5	4.9	16.4	5.6
	7-8/19-20	100.0	0.85	0.99	-2.09	0.67	3.25	0.49	0.1	6.6	9.1	6.6	45.5	7.0
	8-9/18-19	100.0	-0.52	0.55	-0.73	0.62	3.44	0.34	-0.6	4.6	0.6	5.8	30.8	5.6
	5-6/21-22	100.0	0.65	0.60	-0.59	0.62	3.43	0.32	1.6	4.7	1.8	6.5	33.0	4.7
6	6-7/20-21	100.0	-1.38	0.79	-2.20	0.69	3.11	0.51	0.8	6.0	11.0	10.5	40.2	6.8
	7-8/19-20	100.0	0.82	0.46	0.37	0.42	3.73	0.41	0.8	4.1	3.7	5.1	19.3	5.5
	8-9/18-19	100.0	-0.34	0.51	-0.31	0.52	3.42	0.28	-3.6	4.1	1.1	5.5	37.4	4.1

2.2 aA/aC

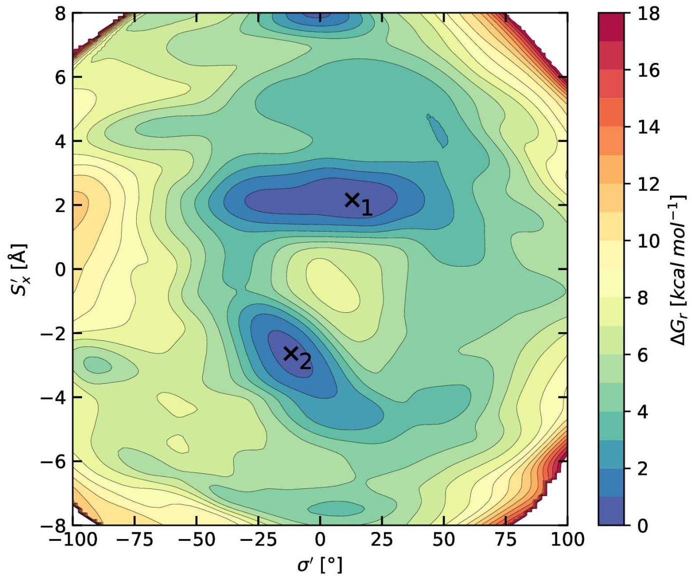

Figure SB2.1: Free energy surface for the aA/aC base pair. Labeled crosses show position of selected free energy minima (thermodynamic states). Free energy isolines are spaced by 1 kcal mol^{-1}.

Table SB2.1: Positions of selected free minima on the free energy surface for the aA/aC base pair. IDs correspond to the selected free energy minima shown in Figure SB2.1. Confidence interval of the free energy ΔG_r is provided at three standard deviations.

ID	σ' [°]	S'_x [Å]	ΔG_r [$kcal\ mol^{-1}$]
1	13.0	2.16	0.00±0.00
2	-11.8	-2.64	0.57±0.10

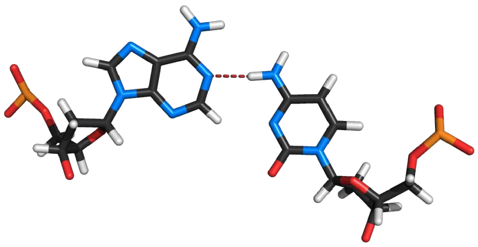

ID 1: $\sigma' = 13.0°$, $S'_x = 2.16$ Å

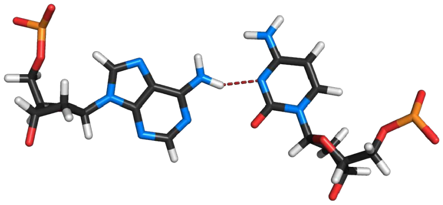

ID 2: $\sigma' = -11.8°$, $S'_x = -2.64$ Å

Figure SB2.2: Average geometries representing selected free energy minima for the aA/aC base pair. IDs correspond to the selected free energy minima shown in Figure SB2.1. Major and minor grooves are top and bottom, respectively. View direction is along the z-axis of DNA.

Table SB2.2: Number of analysed snapshots (N_{snap}), abundances (abu), average hydrogen bond distances and angles from hydrogen bond analysis provided by cpptraj for selected states of the aA/aC base pair. IDs correspond to the selected free energy minima shown in Figure SB2.1. Residue and atom numbering are provided in Figure SA1.

ID	N_{snap}	acceptor	H-donor	donor	abu [%]	d_{avg} [Å]	a_{avg} [°]
1	417	A7@N1	C20@H41	C20@N4	45.3	2.9	162.6
2	396	C20@N3	A7@H61	A7@N6	42.9	2.9	147.4

Table SB2.3: *Simple* base-pair parameters for selected states of the aA/aC base pair. IDs corresponds to the free energy minima shown in Figure SB2.1. Residue numbering in base pairs A/B are provided in Figure SA1. Abundances (*abu*), average values ($\langle X \rangle$) and standard deviations of samples ($s(X)$) are calculated by 3DNA for Shear (S_x), Stretch (S_y), Stagger (S_z), Buckle (κ), Propeller (π), Opening (σ) employing the standard reference frames for the nucleobases. Only five central base pairs were included in the analysis for each state. Base pairs with a mismatch are highlighted in gray.

ID	A/B	abu [%]	$\langle S_x \rangle$	$s(S_x)$	$\langle S_y \rangle$	$s(S_y)$ [Å]	$\langle S_z \rangle$	$s(S_z)$	$\langle \kappa \rangle$	$s(\kappa)$	$\langle \pi \rangle$	$s(\pi)$ [°]	$\langle \sigma \rangle$	$s(\sigma)$
	5/22	100.0	-0.19	0.26	0.03	0.11	-0.07	0.37	-1.7	9.2	-18.1	6.2	-0.4	4.6
	6/21	100.0	-0.19	0.29	0.02	0.12	-0.13	0.38	-7.8	8.9	-12.6	8.0	2.1	5.0
1	7/20	100.0	2.16	0.17	0.83	0.17	0.18	0.50	7.3	11.3	-6.3	9.5	12.5	5.1
	8/19	100.0	0.10	0.27	0.02	0.13	-0.08	0.44	1.9	11.3	-16.4	8.2	1.3	5.6
	9/18	100.0	0.16	0.25	0.02	0.11	0.00	0.40	-1.2	9.2	-16.0	7.5	0.9	4.8
	5/22	100.0	-0.21	0.27	0.06	0.13	0.02	0.41	-0.3	8.7	-14.4	7.4	-0.1	4.7
	6/21	100.0	-0.17	0.26	0.02	0.13	0.04	0.45	1.8	9.5	-7.6	8.5	4.8	5.2
2	7/20	100.0	-2.62	0.31	0.62	0.19	0.27	0.53	21.1	12.1	-10.1	8.5	-11.8	3.7
	8/19	100.0	0.23	0.28	0.05	0.13	0.00	0.42	15.8	10.0	-21.7	8.7	-2.6	5.7
	9/18	100.0	0.23	0.25	0.04	0.12	-0.02	0.38	1.8	9.2	-20.0	6.9	-0.9	4.8

Table SB2.4: *Simple* step parameters for selected states of the aA/aC base pair. IDs corresponds to the free energy minima shown in Figure SB2.1. Residue numbering in base pairs and steps A1-B1/A2-B2 are provided in Figure SA1. Abundances (abu), average values ($\langle X \rangle$) and standard deviations of samples ($s(X)$) are calculated by 3DNA for Shift (D_x), Slide (D_y), Rise (D_z), Tilt (τ), Roll (ρ), Twist (ω) employing the standard reference frames for the nucleobases. Only five central base pairs were included in the analysis for each state. Steps including a mismatch are highlighted in gray.

ID	A1-B1<br>A2-B2	abu<br>[%]	$\langle D_x \rangle$	$s(D_x)$	$\langle D_y \rangle$<br>[Å]	$s(D_y)$	$\langle D_z \rangle$	$s(D_z)$	$\langle \tau \rangle$	$s(\tau)$	$\langle \rho \rangle$	$s(\rho)$<br>[°]	$\langle \omega \rangle$	$s(\omega)$
	5-6/21-22	100.0	0.90	0.54	0.07	0.43	3.39	0.27	3.6	3.8	1.1	5.3	39.6	3.5
1	6-7/20-21	100.0	0.24	0.62	0.52	0.49	3.15	0.31	-1.9	5.5	2.1	6.0	25.3	4.9
	7-8/19-20	100.0	-0.40	1.18	-0.35	0.60	3.34	0.33	2.4	4.9	-0.8	7.5	41.3	7.3
	8-9/18-19	100.0	-0.32	0.66	-0.56	0.56	3.37	0.31	-1.7	4.2	1.3	4.9	34.6	4.1
	5-6/21-22	100.0	1.22	0.44	0.33	0.40	3.27	0.27	2.9	4.0	2.5	5.4	36.8	3.3
2	6-7/20-21	100.0	-2.06	0.48	0.29	0.44	2.74	0.32	-6.9	6.2	0.1	6.7	36.7	4.7
	7-8/19-20	100.0	0.70	0.68	-0.31	0.46	3.69	0.36	2.8	4.7	4.3	5.1	29.6	4.3
	8-9/18-19	100.0	-0.31	0.53	-0.42	0.47	3.56	0.32	-2.4	3.9	4.0	5.6	38.2	4.4

2.3 aA/aG

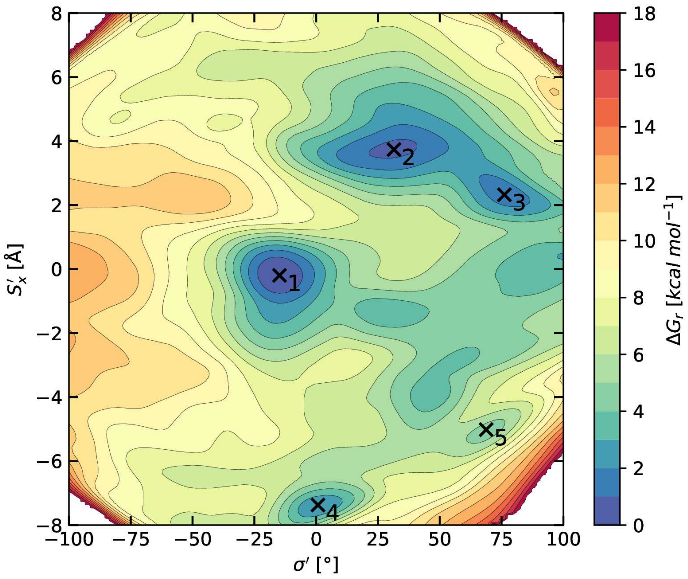

Figure SB3.1: Free energy surface for the aA/aG base pair. Labeled crosses show position of selected free energy minima (thermodynamic states). Free energy isolines are spaced by 1 kcal mol^{-1}.

Table SB3.1: Positions of selected free minima on the free energy surface for the aA/aG base pair. IDs correspond to the selected free energy minima shown in Figure SB3.1. Confidence interval of the free energy ΔG_r is provided at three standard deviations.

ID	σ' [°]	S'_x [Å]	ΔG_r [$kcal\ mol^{-1}$]
1	-14.8	-0.20	0.00±0.00
2	31.5	3.74	0.75±0.15
3	76.1	2.32	1.52±0.15
4	0.7	-7.37	2.07±0.19
5	68.8	-5.02	4.59±0.18

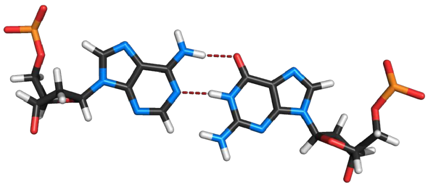

ID 1: σ' = -14.8°, S'_x = -0.20 Å

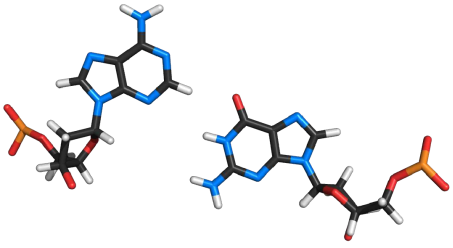

ID 2: σ' = 31.5°, S'_x = 3.74 Å

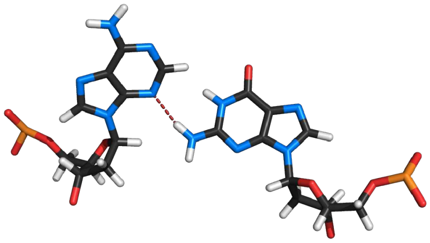

ID 3: σ' = 76.1°, S'_x = 2.32 Å

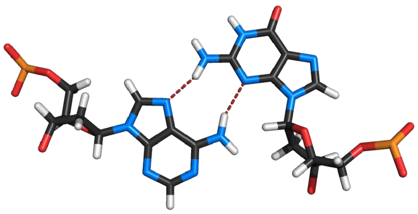

ID 4: σ' = 0.7°, S'_x = -7.37 Å

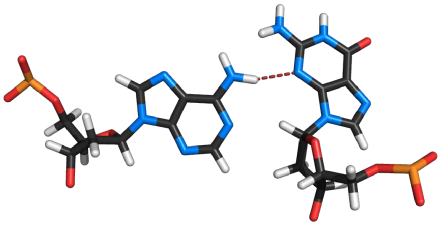

ID 5: σ' = 68.8°, S'_x = -5.02 Å

Figure SB3.2: Average geometries representing selected free energy minima for the aA/aG base pair. IDs correspond to the selected free energy minima shown in Figure SB3.1. Major and minor grooves are top and bottom, respectively. View direction is along the z-axis of DNA.

Table SB3.2: Number of analysed snapshots (N_{snap}), abundances (abu), average hydrogen bond distances and angles from hydrogen bond analysis provided by cpptraj for selected states of the aA/aG base pair. IDs correspond to the selected free energy minima shown in Figure SB3.1. Residue and atom numbering are provided in Figure SA1.

ID	N_{snap}	acceptor	H-donor	donor	abu [%]	d_{avg} [Å]	a_{avg} [°]
1	190	G20@O6	A7@H61	A7@N6	75.8	2.9	160.2
		A7@N1	G20@H1	G20@N1	67.4	2.9	161.6
		G20@O6	A7@H62	A7@N6	4.2	2.8	158.1
2	349	—	—	—	—	—	—
3	357	A7@N3	G20@H21	G20@N2	35.3	2.9	163.5
4	87	A7@N7	G20@H22	G20@N2	60.9	2.9	154.1
		G20@N3	A7@H62	A7@N6	24.1	2.9	154.1
5	253	G20@N3	A7@H61	A7@N6	34.4	2.9	152.0
		A7@N6	G20@H22	G20@N2	4.0	2.9	143.9

Table SB3.3: *Simple* base-pair parameters for selected states of the aA/aG base pair. IDs corresponds to the free energy minima shown in Figure SB3.1. Residue numbering in base pairs A/B are provided in Figure SA1. Abundances (*abu*), average values ($\langle X \rangle$) and standard deviations of samples ($s(X)$) are calculated by 3DNA for Shear (S_x), Stretch (S_y), Stagger (S_z), Buckle (κ), Propeller (π), Opening (σ) employing the standard reference frames for the nucleobases. Only five central base pairs were included in the analysis for each state. Base pairs with a mismatch are highlighted in gray.

ID	A/B	abu [%]	$\langle S_x \rangle$	$s(S_x)$	$\langle S_y \rangle$ [Å]	$s(S_y)$	$\langle S_z \rangle$	$s(S_z)$	$\langle \kappa \rangle$	$s(\kappa)$	$\langle \pi \rangle$ [°]	$s(\pi)$	$\langle \sigma \rangle$	$s(\sigma)$
	5/22	100.0	-0.16	0.28	0.03	0.12	0.05	0.41	-2.0	8.1	-18.7	7.5	-0.6	5.4
	6/21	100.0	-0.17	0.28	0.04	0.13	0.01	0.42	-5.5	10.1	-13.2	8.8	-0.9	5.8
1	7/20	100.0	-0.20	0.16	1.54	0.12	-0.56	0.37	-0.5	10.5	-5.9	9.1	-15.0	2.3
	8/19	100.0	0.14	0.26	0.04	0.11	0.06	0.42	8.0	10.0	-10.1	8.7	-2.2	5.1
	9/18	100.0	0.16	0.28	0.02	0.12	0.15	0.40	4.0	9.4	-18.4	7.4	-0.4	4.7
	5/22	100.0	-0.17	0.26	0.01	0.11	-0.03	0.42	-1.1	9.3	-17.5	7.5	0.4	4.8
	6/21	100.0	-0.15	0.29	0.04	0.12	0.04	0.50	-7.3	11.1	-11.1	8.6	0.1	5.3
2	7/20	28.4	3.66	0.14	2.86	0.23	0.22	0.89	1.4	12.2	-2.8	13.0	28.2	4.8
	8/19	99.7	0.06	0.30	0.02	0.12	-0.18	0.43	-0.5	12.6	-8.0	8.0	0.4	5.3
	9/18	100.0	0.16	0.26	0.02	0.11	0.01	0.41	-0.1	9.8	-14.6	8.0	-0.0	4.8
	5/22	100.0	-0.21	0.28	0.05	0.13	0.05	0.38	4.5	9.3	-15.9	7.4	-0.2	5.4
	6/21	99.4	-0.12	0.27	0.02	0.13	-0.19	0.45	2.7	12.4	-18.9	7.7	2.6	5.7
3	7/20	98.6	2.33	0.17	3.86	0.31	1.26	0.47	18.0	10.9	-31.1	9.5	76.2	3.8
	8/19	100.0	0.20	0.28	-0.03	0.11	-0.26	0.41	1.4	9.9	-12.9	7.2	3.7	4.5
	9/18	100.0	0.20	0.26	0.05	0.12	-0.04	0.38	-2.7	8.3	-16.9	7.1	-0.2	4.8
	5/22	100.0	-0.21	0.28	0.04	0.12	0.06	0.41	2.1	9.3	-12.5	7.6	-1.3	5.4
	6/21	100.0	0.11	0.26	0.02	0.18	0.02	0.54	4.5	11.0	-14.1	9.3	5.2	6.5
4	7/20	100.0	-7.38	0.09	2.39	0.21	0.14	0.61	-5.2	8.2	-12.2	15.2	0.8	2.3
	8/19	100.0	0.05	0.24	-0.03	0.13	-0.13	0.42	10.7	8.0	-12.2	10.8	1.1	5.4
	9/18	100.0	0.20	0.25	0.04	0.11	0.01	0.39	2.3	9.4	-18.6	6.7	-0.3	4.2
	5/22	100.0	-0.15	0.26	0.06	0.13	-0.03	0.43	7.4	9.8	-13.1	8.3	-0.8	5.3
	6/21	99.6	-0.07	0.29	0.04	0.17	-0.22	0.58	9.1	13.9	-11.5	8.0	8.2	6.3
5	7/20	97.2	-5.00	0.16	3.65	0.25	0.56	0.57	-21.1	13.2	-25.4	8.9	69.3	3.1
	8/19	100.0	-0.03	0.28	0.01	0.14	-0.28	0.52	-3.3	11.8	-16.8	8.7	7.2	6.0
	9/18	100.0	0.22	0.26	0.06	0.12	0.00	0.36	-6.5	8.9	-14.5	7.8	-0.2	5.5

Table SB3.4: *Simple* step parameters for selected states of the aA/aG base pair. IDs corresponds to the free energy minima shown in Figure SB3.1. Residue numbering in base pairs and steps A1-B1/A2-B2 are provided in Figure SA1. Abundances (*abu*), average values ($\langle X \rangle$) and standard deviations of samples ($s(X)$) are calculated by 3DNA for Shift (D_x), Slide (D_y), Rise (D_z), Tilt (τ), Roll (ρ), Twist (ω) employing the standard reference frames for the nucleobases. Only five central base pairs were included in the analysis for each state. Steps including a mismatch are highlighted in gray.

ID	A1-B1 A2-B2	abu [%]	$\langle D_x \rangle$	$s(D_x)$	$\langle D_y \rangle$ [Å]	$s(D_y)$	$\langle D_z \rangle$	$s(D_z)$	$\langle \tau \rangle$	$s(\tau)$	$\langle \rho \rangle$ [°]	$s(\rho)$	$\langle \omega \rangle$	$s(\omega)$
1	5-6/21-22	100.0	0.54	0.55	-0.36	0.50	3.37	0.29	2.2	4.5	0.4	5.8	37.7	4.7
	6-7/20-21	100.0	-1.24	0.62	-0.15	0.56	3.21	0.28	2.9	5.2	5.3	6.8	30.1	5.9
	7-8/19-20	100.0	1.14	0.74	-0.12	0.57	3.15	0.26	-3.7	4.7	4.2	7.1	28.3	5.8
	8-9/18-19	100.0	-0.54	0.59	-0.27	0.54	3.40	0.31	-3.1	4.0	0.5	6.1	38.4	4.3
2	5-6/21-22	100.0	0.51	0.59	-0.41	0.55	3.47	0.33	1.9	4.4	1.0	5.5	37.9	4.0
	6-7/20-21	28.4	1.42	0.66	1.00	0.74	3.40	0.30	0.8	6.1	2.8	5.4	21.9	6.2
	6-8/19-21	66.2	-0.25	0.86	-1.50	0.99	6.50	0.41	1.3	7.4	11.7	9.6	59.9	9.4
	7-8/19-20	28.4	-2.20	1.08	-1.40	0.65	2.97	0.45	-1.1	7.3	9.3	10.8	38.1	8.2
	8-9/18-19	99.7	-0.41	0.64	-0.52	0.59	3.35	0.30	-2.5	4.3	-0.6	5.5	34.6	4.9
3	5-6/21-22	99.4	0.36	0.56	-0.52	0.55	3.32	0.29	3.2	4.2	0.3	5.5	33.1	5.9
	6-7/20-21	98.6	2.44	0.51	1.97	0.64	3.51	0.32	3.3	5.6	8.1	4.5	26.6	6.3
	6-8/19-21	0.8	-0.38	0.67	-2.16	1.42	6.96	0.39	-0.8	5.2	15.4	10.1	59.3	6.4
	7-8/19-20	98.6	-2.55	0.48	-1.55	0.52	2.87	0.28	-1.3	6.1	16.1	6.1	31.2	6.4
	8-9/18-19	100.0	-0.79	0.56	-0.01	0.50	3.33	0.25	-4.6	3.7	0.7	4.9	37.3	4.4
4	5-6/21-22	100.0	0.43	0.62	-0.93	0.52	3.34	0.34	0.3	4.6	-0.6	5.9	29.1	4.7
	6-7/20-21	100.0	-0.55	1.11	-2.16	0.83	3.12	0.43	-0.6	6.5	9.1	6.8	48.3	7.2
	7-8/19-20	100.0	0.54	0.39	0.86	0.41	3.54	0.47	2.1	4.7	5.7	5.3	16.9	6.0
	8-9/18-19	100.0	-0.75	0.50	-0.02	0.49	3.51	0.27	-4.0	3.4	1.5	5.8	38.0	3.1
5	5-6/7-22	0.4	1.87	0.00	-1.68	0.00	6.37	0.00	-8.6	0.0	-6.7	0.0	16.6	0.0
	5-6/21-22	99.6	0.46	0.72	-0.66	0.60	3.34	0.35	1.6	4.5	-1.0	5.2	27.6	6.9
	6-7/20-21	97.2	1.08	0.73	-3.12	0.89	2.90	0.48	2.8	8.8	19.7	7.4	41.9	6.2
	6-8/19-7	0.4	0.63	0.00	-0.81	0.00	3.21	0.00	10.2	0.0	19.1	0.0	75.4	0.0
	6-8/19-21	2.4	-0.47	1.19	-1.71	1.38	6.21	0.32	-4.6	8.0	19.1	8.9	55.3	6.3
	7-8/19-20	97.2	-0.78	0.52	3.24	0.48	3.94	0.34	-7.8	6.4	8.0	5.0	20.1	4.6
	8-9/18-19	100.0	-0.55	0.65	-0.39	0.59	3.39	0.29	-4.2	4.3	1.9	5.5	31.8	6.2

2.4 aA:aT

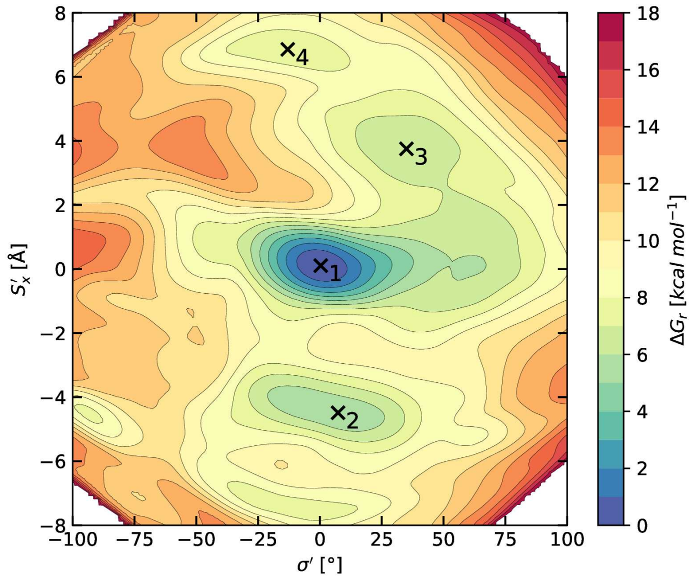

Figure SB4.1: Free energy surface for the aA:aT base pair. Labeled crosses show position of selected free energy minima (thermodynamic states). Free energy isolines are spaced by 1 kcal mol^{-1}.

Table SB4.1: Positions of selected free minima on the free energy surface for the aA:aT base pair. IDs correspond to the selected free energy minima shown in Figure SB4.1. Confidence interval of the free energy ΔG_r is provided at three standard deviations.

ID	σ' [°]	S'_x [Å]	ΔG_r [$kcal\ mol^{-1}$]
1	0.3	0.11	0.00±0.00
2	7.3	-4.49	5.23±0.11
3	35.0	3.75	6.19±0.11
4	-13.0	6.86	7.14±0.14

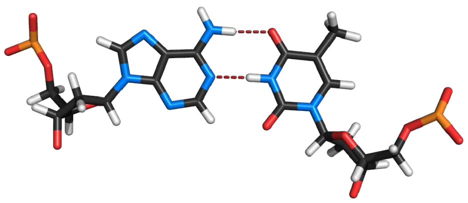

ID 1: $\sigma' = 0.3°$, $S'_x = 0.11$ Å

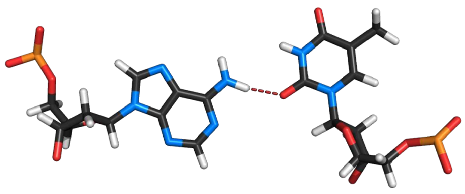

ID 2: $\sigma' = 7.3°$, $S'_x =$ -4.49 Å

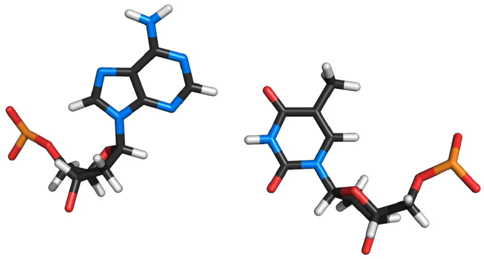

ID 3: $\sigma' = 35.0°$, $S'_x = 3.75$ Å

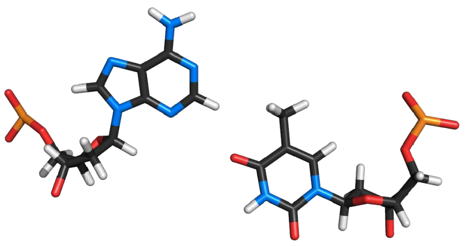

ID 4: $\sigma' =$ -13.0°, $S'_x = 6.86$ Å

Figure SB4.2: Average geometries representing selected free energy minima for the aA:aT base pair. IDs correspond to the selected free energy minima shown in Figure SB4.1. Major and minor grooves are top and bottom, respectively. View direction is along the z-axis of DNA.

Table SB4.2: Number of analysed snapshots (N_{snap}), abundances (abu), average hydrogen bond distances and angles from hydrogen bond analysis provided by cpptraj for selected states of the aA:aT base pair. IDs correspond to the selected free energy minima shown in Figure SB4.1. Residue and atom numbering are provided in Figure SA1.

ID	N_{snap}	acceptor	H-donor	donor	abu [%]	d_{avg} [Å]	a_{avg} [°]
1	210	T20@O4	A7@H61	A7@N6	73.8	2.9	164.9
		A7@N1	T20@H3	T20@N3	71.0	2.9	166.3
2	385	T20@O2	A7@H61	A7@N6	59.0	2.9	160.7
3	553	—	—	—	—	—	—
4	289	—	—	—	—	—	—

Table SB4.3: *Simple* base-pair parameters for selected states of the aA:aT base pair. IDs corresponds to the free energy minima shown in Figure SB4.1. Residue numbering in base pairs A/B are provided in Figure SA1. Abundances (*abu*), average values ($\langle X\rangle$) and standard deviations of samples ($s(X)$) are calculated by 3DNA for Shear (S_x), Stretch (S_y), Stagger (S_z), Buckle (κ), Propeller (π), Opening (σ) employing the standard reference frames for the nucleobases. Only five central base pairs were included in the analysis for each state. Base pairs with a mismatch are highlighted in gray.

ID	A/B	abu [%]	$\langle S_x\rangle$	$s(S_x)$	$\langle S_y\rangle$ [Å]	$s(S_y)$	$\langle S_z\rangle$	$s(S_z)$	$\langle\kappa\rangle$	$s(\kappa)$	$\langle\pi\rangle$ [°]	$s(\pi)$	$\langle\sigma\rangle$	$s(\sigma)$
	5/22	100.0	-0.17	0.26	0.03	0.11	0.04	0.38	-1.3	9.9	-15.4	7.3	-0.5	5.2
	6/21	100.0	-0.14	0.27	0.03	0.12	-0.09	0.43	-1.5	11.6	-8.5	9.4	2.4	5.6
1	7/20	100.0	0.11	0.13	-0.00	0.11	-0.24	0.40	1.2	9.8	-8.3	7.4	0.0	2.7
	8/19	100.0	0.12	0.26	-0.00	0.11	-0.06	0.40	6.8	8.8	-12.0	7.1	0.6	4.9
	9/18	100.0	0.18	0.25	0.01	0.11	-0.03	0.38	0.3	8.9	-18.0	7.2	-0.3	4.7
	5/22	100.0	-0.17	0.28	0.02	0.12	0.09	0.39	0.9	8.7	-16.3	8.0	-0.8	5.0
	6/21	100.0	-0.06	0.43	0.01	0.13	-0.04	0.46	0.9	11.4	-12.4	8.7	0.4	5.9
2	7/20	100.0	-4.47	0.18	1.76	0.31	0.30	0.66	5.8	11.1	-8.5	11.5	7.0	5.7
	8/19	100.0	0.19	0.35	0.06	0.14	0.18	0.46	11.0	10.8	-14.5	9.3	-0.7	5.6
	9/18	100.0	0.20	0.26	0.03	0.13	-0.01	0.42	3.0	9.5	-20.2	7.4	-0.7	5.2
	5/22	100.0	-0.20	0.26	0.04	0.11	-0.07	0.39	2.5	9.8	-16.8	7.0	0.6	4.9
	6/21	100.0	-0.20	0.28	0.03	0.14	-0.28	0.46	-3.5	11.8	-15.0	9.0	3.6	6.8
3	7/20	36.9	3.70	0.24	1.87	0.20	0.07	0.73	4.1	12.2	-13.9	12.9	30.6	4.8
	8/19	100.0	0.07	0.28	-0.01	0.12	-0.27	0.42	2.5	10.7	-8.8	8.3	0.6	4.9
	9/18	100.0	0.19	0.27	0.04	0.13	-0.05	0.37	-0.9	9.1	-15.6	7.6	-0.8	5.1
	5/22	100.0	-0.16	0.26	0.02	0.12	-0.00	0.36	-1.6	8.9	-19.0	7.6	-0.6	4.6
	6/21	100.0	-0.22	0.26	0.08	0.13	-0.10	0.39	-10.8	10.3	-16.6	9.4	-2.8	5.5
4	7/20	77.2	6.85	0.16	3.26	0.28	-0.59	0.71	0.4	10.2	-13.5	12.3	-12.2	6.8
	8/19	100.0	0.10	0.28	0.02	0.13	-0.37	0.49	-3.8	12.4	-9.9	8.0	2.4	5.5
	9/18	100.0	0.18	0.25	0.03	0.12	-0.03	0.40	-1.0	9.4	-14.5	8.1	-0.7	4.9

Table SB4.4: *Simple* step parameters for selected states of the aA:aT base pair. IDs corresponds to the free energy minima shown in Figure SB4.1. Residue numbering in base pairs and steps A1-B1/A2-B2 are provided in Figure SA1. Abundances (*abu*), average values ($\langle X \rangle$) and standard deviations of samples ($s(X)$) are calculated by 3DNA for Shift (D_x), Slide (D_y), Rise (D_z), Tilt (τ), Roll (ρ), Twist (ω) employing the standard reference frames for the nucleobases. Only five central base pairs were included in the analysis for each state. Steps including a mismatch are highlighted in gray.

ID	A1-B1 A2-B2	abu [%]	$\langle D_x \rangle$	$s(D_x)$	$\langle D_y \rangle$ [Å]	$s(D_y)$	$\langle D_z \rangle$	$s(D_z)$	$\langle \tau \rangle$	$s(\tau)$	$\langle \rho \rangle$ [°]	$s(\rho)$	$\langle \omega \rangle$	$s(\omega)$
	5-6/21-22	100.0	0.82	0.68	-0.14	0.58	3.32	0.28	2.8	4.4	0.8	5.8	36.3	4.0
1	6-7/20-21	100.0	-0.57	1.13	0.10	0.78	3.31	0.36	-0.9	4.8	6.9	7.8	31.8	7.1
	7-8/19-20	100.0	-0.07	0.69	-0.41	0.53	3.22	0.28	-1.9	3.8	1.2	5.4	31.9	5.6
	8-9/18-19	100.0	-0.60	0.64	-0.36	0.56	3.43	0.26	-2.6	4.0	0.3	5.0	38.0	4.0
	5-6/21-22	100.0	0.35	0.66	-0.38	0.59	3.31	0.30	2.1	4.0	-0.2	5.2	35.5	5.0
2	6-7/20-21	100.0	0.89	1.18	-0.79	0.62	2.76	0.39	-0.8	5.9	13.3	8.7	38.7	7.9
	7-8/19-20	100.0	-0.62	0.76	0.28	0.65	3.49	0.34	-0.7	4.9	4.8	5.0	21.0	5.0
	8-9/18-19	100.0	-0.58	0.57	-0.34	0.49	3.50	0.31	-1.2	4.5	1.1	6.1	38.0	4.0
	5-6/21-22	100.0	0.75	0.59	-0.14	0.48	3.38	0.30	4.7	4.3	2.9	5.4	38.8	3.8
	6-7/20-21	36.9	0.62	0.65	1.19	0.51	3.44	0.41	0.2	5.9	3.2	5.8	23.6	5.4
3	6-8/19-21	61.3	0.01	1.04	-1.36	0.92	6.52	0.40	0.2	6.4	12.7	9.0	59.7	8.2
	7-8/19-20	36.9	-1.87	0.84	-1.37	0.54	3.08	0.46	-1.6	6.7	5.7	8.7	39.8	8.5
	8-9/18-19	100.0	-0.44	0.59	-0.51	0.59	3.42	0.28	-2.7	3.9	-1.1	5.4	35.0	4.7
	5-6/21-22	100.0	0.29	0.57	-0.48	0.57	3.48	0.30	2.9	3.8	2.5	5.7	38.8	3.9
	6-7/20-21	77.2	-0.96	0.57	0.20	0.63	3.72	0.43	1.5	4.3	2.4	4.6	20.4	5.1
4	6-8/19-21	21.4	0.12	0.94	-0.07	1.33	6.68	0.36	1.0	7.2	9.9	8.0	60.2	8.2
	7-8/19-20	77.2	1.30	0.93	-0.55	0.74	3.20	0.51	1.0	7.0	10.4	9.6	41.1	9.8
	8-9/18-19	100.0	-0.42	0.62	-0.41	0.63	3.26	0.30	-3.5	4.2	-1.5	5.8	33.7	6.8

2.5 aG:aC

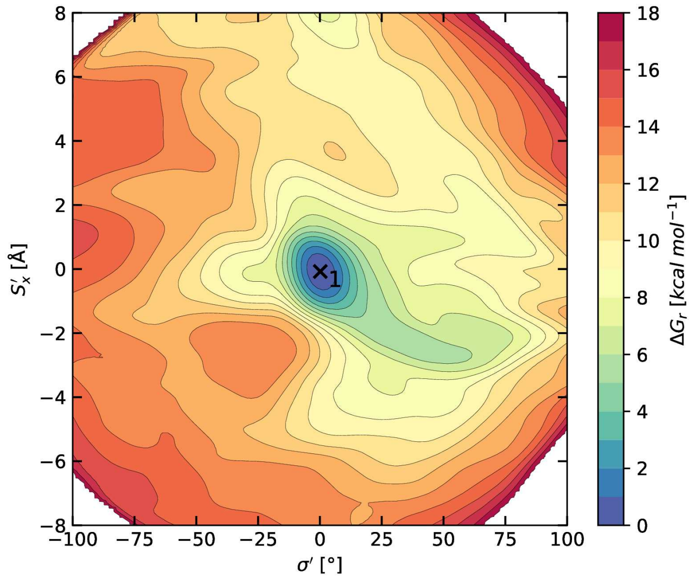

Figure SB5.1: Free energy surface for the aG:aC base pair. Labeled crosses show position of selected free energy minima (thermodynamic states). Free energy isolines are spaced by 1 kcal mol^{-1}.

Table SB5.1: Positions of selected free minima on the free energy surface for the aG:aC base pair. IDs correspond to the selected free energy minima shown in Figure SB5.1. Confidence interval of the free energy ΔG_r is provided at three standard deviations.

ID	σ' [°]	S'_x [Å]	ΔG_r [$kcal\ mol^{-1}$]
1	0.2	-0.08	0.00±0.00

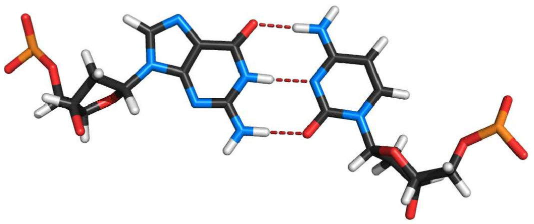

ID 1: $\sigma' = 0.2°$, S'_x = -0.08 Å

Figure SB5.2: Average geometries representing selected free energy minima for the aG:aC base pair. IDs correspond to the selected free energy minima shown in Figure SB5.1. Major and minor grooves are top and bottom, respectively. View direction is along the z-axis of DNA.

Table SB5.2: Number of analysed snapshots (N_{snap}), abundances (abu), average hydrogen bond distances and angles from hydrogen bond analysis provided by cpptraj for selected states of the aG:aC base pair. IDs correspond to the selected free energy minima shown in Figure SB5.1. Residue and atom numbering are provided in Figure SA1.

ID	N_{snap}	acceptor	H-donor	donor	abu [%]	d_{avg} [Å]	a_{avg} [°]
		C20@O2	G7@H21	G7@N2	89.1	2.8	164.3
		C20@N3	G7@H1	G7@N1	74.8	2.9	167.1
1	147	G7@O6	C20@H41	C20@N4	73.5	2.9	163.6
		C20@O2	G7@H22	G7@N2	3.4	2.9	161.4
		G7@O6	C20@H42	C20@N4	2.0	2.8	161.7

Table SB5.3: *Simple* base-pair parameters for selected states of the aG:aC base pair. IDs corresponds to the free energy minima shown in Figure SB5.1. Residue numbering in base pairs A/B are provided in Figure SA1. Abundances (*abu*), average values ($\langle X \rangle$) and standard deviations of samples ($s(X)$) are calculated by 3DNA for Shear (S_x), Stretch (S_y), Stagger (S_z), Buckle (κ), Propeller (π), Opening (σ) employing the standard reference frames for the nucleobases. Only five central base pairs were included in the analysis for each state. Base pairs with a mismatch are highlighted in gray.

ID	A/B	abu	$\langle S_x \rangle$	$s(S_x)$	$\langle S_y \rangle$	$s(S_y)$	$\langle S_z \rangle$	$s(S_z)$	$\langle \kappa \rangle$	$s(\kappa)$	$\langle \pi \rangle$	$s(\pi)$	$\langle \sigma \rangle$	$s(\sigma)$
		[%]			[Å]						[°]			
	5/22	100.0	-0.15	0.26	0.02	0.12	0.05	0.39	-1.6	9.0	-16.0	7.0	-0.6	5.3
	6/21	100.0	-0.11	0.29	0.01	0.13	-0.14	0.42	-3.2	9.1	-6.2	8.0	-0.1	5.6
1	7/20	100.0	-0.07	0.17	-0.04	0.09	-0.04	0.39	5.3	10.6	-4.4	8.8	0.2	1.5
	8/19	100.0	0.16	0.26	0.03	0.13	-0.05	0.36	6.5	9.1	-14.2	7.2	-1.9	4.9
	9/18	100.0	0.18	0.26	0.02	0.10	-0.04	0.36	1.6	7.7	-17.7	7.5	-0.3	4.4

Table SB5.4: *Simple* step parameters for selected states of the aG:aC base pair. IDs corresponds to the free energy minima shown in Figure SB5.1. Residue numbering in base pairs and steps A1-B1/A2-B2 are provided in Figure SA1. Abundances (*abu*), average values ($\langle X \rangle$) and standard deviations of samples ($s(X)$) are calculated by 3DNA for Shift (D_x), Slide (D_y), Rise (D_z), Tilt (τ), Roll (ρ), Twist (ω) employing the standard reference frames for the nucleobases. Only five central base pairs were included in the analysis for each state. Steps including a mismatch are highlighted in gray.

ID	A1-B1<br>A2-B2	abu<br>[%]	$\langle D_x \rangle$	$s(D_x)$	$\langle D_y \rangle$	$s(D_y)$<br>[Å]	$\langle D_z \rangle$	$s(D_z)$	$\langle \tau \rangle$	$s(\tau)$	$\langle \rho \rangle$	$s(\rho)$<br>[°]	$\langle \omega \rangle$	$s(\omega)$
	5-6/21-22	100.0	0.55	0.74	-0.39	0.61	3.36	0.28	3.5	4.2	-0.5	5.3	36.9	4.5
1	6-7/20-21	100.0	0.31	0.91	-0.25	0.46	3.17	0.33	-0.2	5.5	9.6	5.9	26.0	6.6
	7-8/19-20	100.0	-0.66	0.62	-0.06	0.59	3.35	0.28	-1.2	4.5	0.6	5.1	36.7	6.2
	8-9/18-19	100.0	-0.24	0.56	-0.53	0.48	3.40	0.25	-1.3	3.8	0.4	4.1	36.5	3.7

2.6 aG/aG

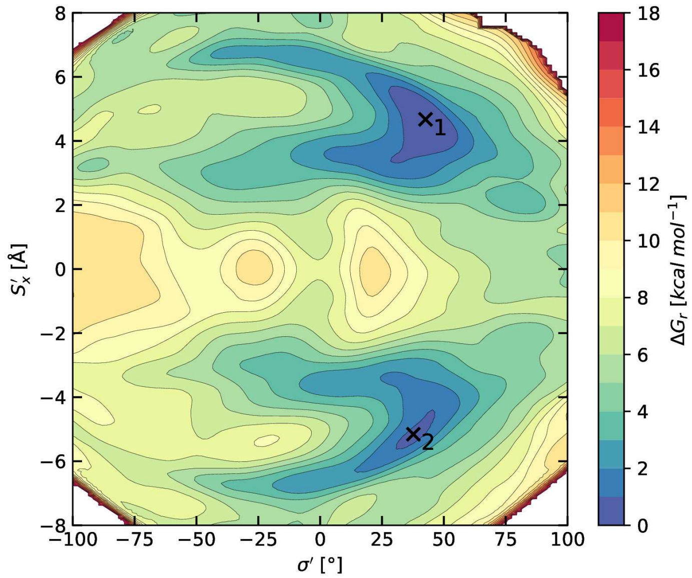

Figure SB6.1: Free energy surface for the aG/aG base pair. Labeled crosses show position of selected free energy minima (thermodynamic states). Free energy isolines are spaced by 1 kcal mol^{-1}.

Table SB6.1: Positions of selected free minima on the free energy surface for the aG/aG base pair. IDs correspond to the selected free energy minima shown in Figure SB6.1. Confidence interval of the free energy ΔG_r is provided at three standard deviations.

ID	σ' [°]	S'_x [Å]	ΔG_r [$kcal\ mol^{-1}$]
1	42.6	4.67	0.00±0.00
2	37.7	-5.16	0.72±0.23

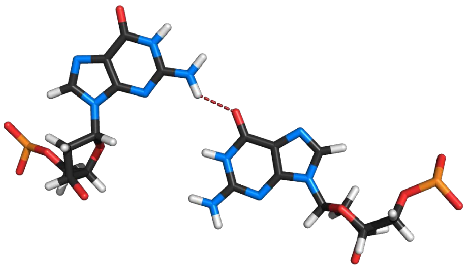

ID 1: $\sigma' = 42.6°$, $S'_x = 4.67$ Å

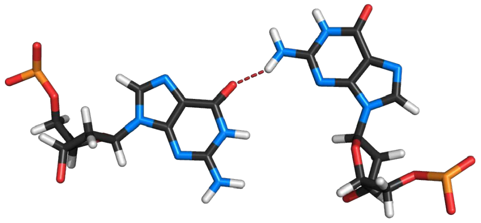

ID 2: $\sigma' = 37.7°$, $S'_x = $ -5.16 Å

Figure SB6.2: Average geometries representing selected free energy minima for the aG/aG base pair. IDs correspond to the selected free energy minima shown in Figure SB6.1. Major and minor grooves are top and bottom, respectively. View direction is along the z-axis of DNA.

Table SB6.2: Number of analysed snapshots (N_{snap}), abundances (abu), average hydrogen bond distances and angles from hydrogen bond analysis provided by cpptraj for selected states of the aG/aG base pair. IDs correspond to the selected free energy minima shown in Figure SB6.1. Residue and atom numbering are provided in Figure SA1.

ID	N_{snap}	acceptor	H-donor	donor	abu [%]	d_{avg} [Å]	a_{avg} [°]
1	243	G20@O6	G7@H22	G7@N2	33.7	2.9	145.0
2	339	G7@O6	G20@H22	G20@N2	64.6	2.8	151.6

Table SB6.3: *Simple* base-pair parameters for selected states of the aG/aG base pair. IDs corresponds to the free energy minima shown in Figure SB6.1. Residue numbering in base pairs A/B are provided in Figure SA1. Abundances (*abu*), average values ($\langle X \rangle$) and standard deviations of samples ($s(X)$) are calculated by 3DNA for Shear (S_x), Stretch (S_y), Stagger (S_z), Buckle (κ), Propeller (π), Opening (σ) employing the standard reference frames for the nucleobases. Only five central base pairs were included in the analysis for each state. Base pairs with a mismatch are highlighted in gray.

ID	A/B	abu [%]	$\langle S_x \rangle$	$s(S_x)$	$\langle S_y \rangle$ [Å]	$s(S_y)$	$\langle S_z \rangle$	$s(S_z)$	$\langle \kappa \rangle$	$s(\kappa)$	$\langle \pi \rangle$ [°]	$s(\pi)$	$\langle \sigma \rangle$	$s(\sigma)$
	5/22	100.0	-0.13	0.25	0.02	0.13	-0.01	0.41	-1.7	9.5	-18.1	7.5	-0.3	5.3
	6/21	99.6	-0.14	0.50	0.06	0.27	0.10	0.51	-6.7	10.7	-9.8	9.2	2.8	6.5
1	7/20	100.0	4.65	0.32	4.19	0.26	0.51	0.75	2.4	9.6	-2.4	11.5	43.0	3.8
	8/19	100.0	0.06	0.26	0.01	0.12	-0.33	0.44	-7.7	12.0	-11.5	7.5	1.5	5.1
	9/18	100.0	0.15	0.26	0.03	0.11	-0.01	0.36	-4.3	9.0	-12.4	8.2	1.0	5.1
	5/22	100.0	-0.18	0.26	0.02	0.12	-0.04	0.41	2.0	10.1	-13.6	8.1	-0.4	5.0
	6/20	5.9	-3.54	0.29	2.29	0.38	-0.53	0.61	-0.1	9.7	-22.0	12.6	18.0	5.8
	6/21	92.3	1.50	2.81	0.47	0.81	-0.11	0.56	8.0	12.0	-9.4	9.0	-3.8	14.7
2	7/20	92.9	-5.16	0.24	4.02	0.23	0.10	0.69	0.7	11.6	-1.7	11.4	37.4	3.8
	8/19	100.0	0.04	0.26	0.04	0.14	-0.01	0.50	9.1	11.0	-8.4	10.4	1.8	5.9
	9/18	100.0	0.17	0.27	0.01	0.11	-0.00	0.41	3.0	10.1	-17.7	7.8	0.3	5.2

Table SB6.4: *Simple* step parameters for selected states of the aG/aG base pair. IDs corresponds to the free energy minima shown in Figure SB6.1. Residue numbering in base pairs and steps A1-B1/A2-B2 are provided in Figure SA1. Abundances (*abu*), average values ($\langle X \rangle$) and standard deviations of samples ($s(X)$) are calculated by 3DNA for Shift (D_x), Slide (D_y), Rise (D_z), Tilt (τ), Roll (ρ), Twist (ω) employing the standard reference frames for the nucleobases. Only five central base pairs were included in the analysis for each state. Steps including a mismatch are highlighted in gray.

ID	A1-B1 A2-B2	abu [%]	$\langle D_x \rangle$	$s(D_x)$	$\langle D_y \rangle$ [Å]	$s(D_y)$	$\langle D_z \rangle$	$s(D_z)$	$\langle \tau \rangle$	$s(\tau)$	$\langle \rho \rangle$ [°]	$s(\rho)$	$\langle \omega \rangle$	$s(\omega)$
	5-6/21-22	99.6	0.79	0.65	-0.27	0.51	3.45	0.30	2.2	4.5	-0.0	5.2	37.5	4.5
	5-7/20-22	0.4	2.32	0.00	0.93	0.00	6.64	0.00	1.6	0.0	10.7	0.0	61.8	0.0
1	6-7/20-21	99.6	1.58	0.61	1.77	0.64	3.41	0.33	-0.4	4.9	4.6	4.4	18.4	5.2
	7-8/19-20	100.0	-2.80	0.85	-2.62	0.82	3.19	0.55	3.6	6.3	7.0	7.3	44.3	5.6
	8-9/18-19	100.0	-0.17	0.52	-0.66	0.51	3.24	0.28	-2.5	4.1	-0.7	4.9	32.4	4.2
	5-6/7-22	1.2	1.90	0.51	0.09	0.34	5.30	0.24	-12.4	2.5	-1.2	4.4	-11.9	6.1
	5-6/20-22	5.9	4.94	0.52	-0.26	0.52	4.11	0.31	-9.0	5.9	2.5	5.7	62.2	3.1
	5-6/21-22	92.3	0.14	0.89	-0.55	0.60	3.36	0.34	0.3	4.7	-0.4	5.6	29.2	7.0
	6-7/20-21	92.3	2.41	1.31	-2.65	1.12	3.12	0.57	-2.4	6.9	7.5	8.6	47.7	10.6
2	6-8/19-7	1.2	0.25	0.30	-0.07	0.51	4.20	0.62	3.4	2.6	8.3	4.8	111.6	6.4
	6-8/19-20	5.9	-3.09	0.64	1.54	0.65	4.47	0.34	10.7	5.4	13.9	6.2	35.5	5.2
	7-8/19-20	92.9	-1.40	0.65	1.69	0.62	3.39	0.34	-1.0	4.9	3.9	4.5	17.9	6.0
	8-9/18-19	100.0	-0.77	0.58	-0.25	0.50	3.49	0.28	-2.9	4.3	0.7	5.9	37.9	4.0

2.7 aG/aT

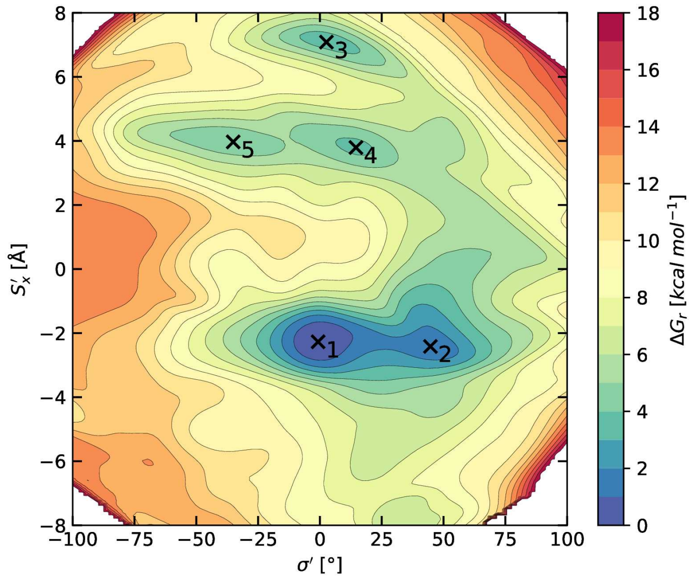

Figure SB7.1: Free energy surface for the aG/aT base pair. Labeled crosses show position of selected free energy minima (thermodynamic states). Free energy isolines are spaced by 1 kcal mol^{-1}.

Table SB7.1: Positions of selected free minima on the free energy surface for the aG/aT base pair. IDs correspond to the selected free energy minima shown in Figure SB7.1. Confidence interval of the free energy ΔG_r is provided at three standard deviations.

ID	σ' [°]	S'_x [Å]	ΔG_r [$kcal\ mol^{-1}$]
1	-0.7	-2.27	0.00±0.00
2	44.7	-2.42	1.26±0.09
3	2.6	7.08	3.41±0.13
4	14.6	3.79	3.78±0.11
5	-35.1	3.97	4.08±0.11

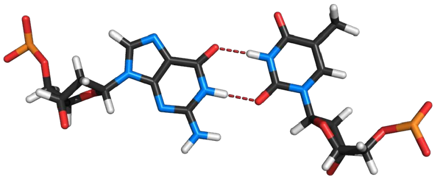

ID 1: $\sigma' = \text{-}0.7°$, $S'_x = \text{-}2.27$ Å

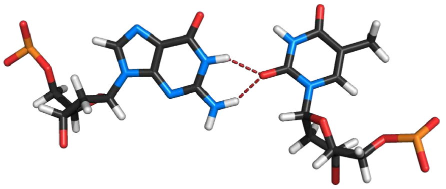

ID 2: $\sigma' = 44.7°$, $S'_x = \text{-}2.42$ Å

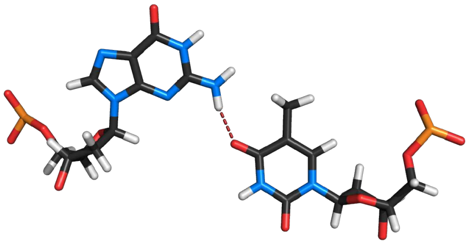

ID 3: $\sigma' = 2.6°$, $S'_x = 7.08$ Å

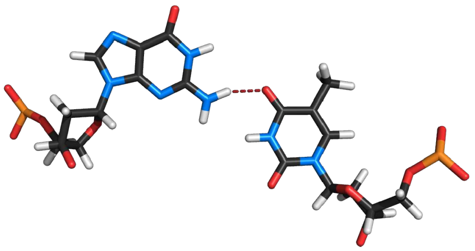

ID 4: $\sigma' = 14.6°$, $S'_x = 3.79$ Å

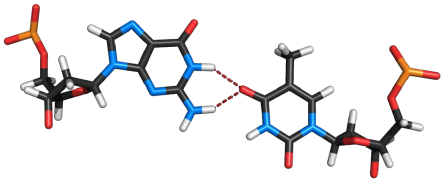

ID 5: $\sigma' = \text{-}35.1°$, $S'_x = 3.97$ Å

Figure SB7.2: Average geometries representing selected free energy minima for the aG/aT base pair. IDs correspond to the selected free energy minima shown in Figure SB7.1. Major and minor grooves are top and bottom, respectively. View direction is along the z-axis of DNA.

Table SB7.2: Number of analysed snapshots (N_{snap}), abundances (abu), average hydrogen bond distances and angles from hydrogen bond analysis provided by cpptraj for selected states of the aG/aT base pair. IDs correspond to the selected free energy minima shown in Figure SB7.1. Residue and atom numbering are provided in Figure SA1.

ID	N_{snap}	acceptor	H-donor	donor	abu [%]	d_{avg} [Å]	a_{avg} [°]
1	315	T20@O2	G7@H1	G7@N1	89.2	2.8	166.2
		G7@O6	T20@H3	T20@N3	82.9	2.9	164.3
2	252	T20@O2	G7@H21	G7@N2	81.8	2.9	153.8
		T20@O2	G7@H1	G7@N1	49.2	2.9	145.6
		T20@O2	G7@H22	G7@N2	1.6	2.9	149.6
3	205	T20@O4	G7@H22	G7@N2	61.5	2.9	157.7
4	284	T20@O4	G7@H21	G7@N2	76.1	2.8	152.7
		T20@O4	G7@H22	G7@N2	2.5	2.8	153.9
5	278	T20@O4	G7@H21	G7@N2	82.4	2.8	150.5
		T20@O4	G7@H1	G7@N1	57.2	2.9	145.0

Table SB7.3: *Simple* base-pair parameters for selected states of the aG/aT base pair. IDs corresponds to the free energy minima shown in Figure SB7.1. Residue numbering in base pairs A/B are provided in Figure SA1. Abundances (*abu*), average values ($\langle X \rangle$) and standard deviations of samples ($s(X)$) are calculated by 3DNA for Shear (S_x), Stretch (S_y), Stagger (S_z), Buckle (κ), Propeller (π), Opening (σ) employing the standard reference frames for the nucleobases. Only five central base pairs were included in the analysis for each state. Base pairs with a mismatch are highlighted in gray.

ID	A/B	abu [%]	$\langle S_x \rangle$	$s(S_x)$	$\langle S_y \rangle$ [Å]	$s(S_y)$	$\langle S_z \rangle$	$s(S_z)$	$\langle \kappa \rangle$	$s(\kappa)$	$\langle \pi \rangle$ [°]	$s(\pi)$	$\langle \sigma \rangle$	$s(\sigma)$
	5/22	100.0	-0.17	0.28	0.03	0.12	0.02	0.40	-1.5	9.6	-16.0	7.3	-0.6	5.0
	6/21	100.0	0.03	0.70	0.04	0.24	-0.10	0.39	-1.3	10.9	-9.1	8.5	0.6	6.0
1	7/20	100.0	-2.26	0.17	0.10	0.13	0.09	0.41	3.0	11.2	-5.2	10.5	-0.8	2.7
	8/19	100.0	0.13	0.27	0.03	0.12	0.04	0.45	7.6	10.4	-13.3	8.5	-0.7	5.1
	9/18	100.0	0.17	0.28	0.01	0.11	0.00	0.40	1.5	10.2	-17.2	7.6	-0.1	5.0
	5/22	100.0	-0.20	0.26	0.04	0.13	0.01	0.37	-0.7	9.3	-15.8	7.0	-0.7	4.7
	6/21	100.0	0.03	0.76	0.04	0.30	-0.27	0.41	-0.8	10.2	-7.3	9.5	2.6	5.6
2	7/20	100.0	-2.41	0.14	1.94	0.21	0.63	0.52	-3.0	10.3	-19.2	8.9	44.7	3.4
	8/19	100.0	0.16	0.28	0.01	0.12	-0.13	0.39	1.8	10.8	-15.8	7.8	2.6	4.9
	9/18	100.0	0.20	0.25	0.04	0.13	-0.00	0.38	-3.4	9.1	-17.8	6.9	0.2	4.8
	5/22	100.0	-0.17	0.25	0.01	0.11	0.01	0.39	-2.8	8.8	-18.2	7.1	-0.6	5.1
	6/21	100.0	-0.15	0.24	0.06	0.13	-0.10	0.37	-9.7	8.6	-14.6	9.8	-1.6	5.3
3	7/20	100.0	7.10	0.11	3.72	0.20	-0.33	0.57	4.7	8.2	-12.8	11.6	2.4	4.0
	8/19	100.0	0.06	0.27	0.01	0.14	-0.23	0.47	-0.5	11.2	-10.1	7.8	3.8	5.6
	9/18	100.0	0.15	0.26	0.02	0.14	-0.04	0.44	-1.4	9.4	-14.8	7.3	0.1	4.7
	5/22	100.0	-0.20	0.25	0.02	0.12	0.04	0.41	-2.9	9.2	-17.1	6.8	-0.7	4.6
	6/21	100.0	-0.21	0.26	0.05	0.11	0.06	0.38	-6.0	9.1	-6.4	7.7	0.1	4.7
4	7/20	100.0	3.80	0.16	2.39	0.23	-0.01	0.57	2.1	10.2	-9.5	8.2	14.5	4.0
	8/19	99.3	0.21	0.62	0.07	0.30	-0.09	0.40	0.5	10.2	-12.5	7.3	0.1	5.9
	9/18	100.0	0.11	0.26	0.01	0.11	0.06	0.40	-1.5	9.4	-14.0	7.3	-0.1	4.6
	5/22	100.0	-0.12	0.28	0.02	0.12	0.02	0.42	-4.4	8.8	-17.7	7.3	-0.7	5.2
	6/21	99.6	-0.12	0.39	0.09	0.22	-0.09	0.43	-8.8	9.4	-13.9	8.8	-4.3	6.9
5	7/20	100.0	3.96	0.12	2.08	0.19	-0.75	0.44	1.1	10.9	-10.2	8.6	-35.4	5.0
	8/19	100.0	0.20	0.27	0.05	0.14	-0.09	0.46	4.6	10.7	-13.5	8.2	0.8	5.5
	9/18	100.0	0.13	0.26	0.02	0.12	0.07	0.39	0.6	8.6	-17.3	6.7	0.1	5.5

Table SB7.4: *Simple* step parameters for selected states of the aG/aT base pair. IDs corresponds to the free energy minima shown in Figure SB7.1. Residue numbering in base pairs and steps A1-B1/A2-B2 are provided in Figure SA1. Abundances (*abu*), average values ($\langle X \rangle$) and standard deviations of samples ($s(X)$) are calculated by 3DNA for Shift (D_x), Slide (D_y), Rise (D_z), Tilt (τ), Roll (ρ), Twist (ω) employing the standard reference frames for the nucleobases. Only five central base pairs were included in the analysis for each state. Steps including a mismatch are highlighted in gray.

ID	A1-B1<br>A2-B2	abu<br>[%]	$\langle D_x \rangle$	$s(D_x)$	$\langle D_y \rangle$<br>[Å]	$s(D_y)$	$\langle D_z \rangle$	$s(D_z)$	$\langle \tau \rangle$	$s(\tau)$	$\langle \rho \rangle$<br>[°]	$s(\rho)$	$\langle \omega \rangle$	$s(\omega)$
	5-6/21-22	100.0	0.50	0.62	-0.40	0.62	3.34	0.27	2.3	4.0	-0.2	4.9	34.8	5.0
1	6-7/20-21	100.0	0.63	0.86	-0.47	0.56	3.08	0.42	-1.1	5.2	10.0	7.8	33.9	8.8
	7-8/19-20	100.0	-0.71	0.66	-0.09	0.71	3.39	0.28	-0.9	4.0	2.2	4.8	29.1	4.7
	8-9/18-19	100.0	-0.32	0.57	-0.56	0.53	3.45	0.32	-1.6	3.9	0.9	5.3	36.6	3.9
	5-6/21-22	100.0	0.75	0.72	-0.16	0.67	3.35	0.27	3.6	3.7	-0.1	5.5	35.0	4.8
2	6-7/20-21	100.0	1.60	0.94	-0.85	0.64	2.89	0.40	-4.0	5.6	14.7	6.7	35.0	7.9
	7-8/19-20	100.0	-2.04	0.52	0.52	0.63	3.44	0.30	-0.5	4.5	4.4	4.4	22.9	5.2
	8-9/18-19	100.0	-0.74	0.50	-0.17	0.51	3.36	0.28	-3.7	3.8	1.3	5.2	37.9	4.1
	5-6/21-22	100.0	0.47	0.57	-0.34	0.48	3.43	0.29	3.2	4.0	1.8	6.0	39.2	3.7
3	6-7/20-21	100.0	-0.39	0.45	0.66	0.49	3.62	0.40	1.6	4.3	2.9	4.3	18.1	5.0
	7-8/19-20	100.0	0.76	1.01	-1.27	0.93	3.06	0.45	0.7	5.9	10.3	6.7	39.9	9.2
	8-9/18-19	100.0	-0.70	0.60	-0.31	0.57	3.34	0.28	-3.0	4.6	-0.6	6.1	36.3	5.0
	5-6/21-22	100.0	0.70	0.53	-0.22	0.44	3.40	0.28	2.1	3.7	-0.8	5.9	39.7	3.3
	6-7/20-21	100.0	0.68	0.62	0.50	0.54	3.41	0.31	3.2	4.5	8.3	5.0	17.1	4.4
4	7-8/19-20	99.3	-1.54	0.82	-1.12	0.51	3.03	0.31	-4.2	4.9	1.6	6.1	41.8	5.2
	7-9/18-20	0.7	-1.25	0.07	-1.43	0.09	6.25	0.10	-7.8	0.1	6.0	2.5	73.0	7.3
	8-9/18-19	99.3	-0.23	0.59	-0.89	0.50	3.35	0.33	-2.0	4.0	-0.9	4.9	35.1	4.2
	5-6/21-22	99.6	0.27	0.69	-0.43	0.49	3.36	0.27	2.9	4.4	1.6	6.1	38.5	4.0
5	6-7/20-21	99.6	-1.84	0.70	-0.38	0.58	3.50	0.35	2.8	4.2	7.1	6.0	22.1	5.6
	7-8/19-20	100.0	1.98	0.78	-0.15	0.59	3.11	0.41	-3.8	5.1	1.6	6.0	34.0	6.1
	8-9/18-19	100.0	-0.44	0.56	-0.33	0.52	3.35	0.27	-3.3	4.6	-0.4	5.3	39.0	4.5

2.8 aC/aC

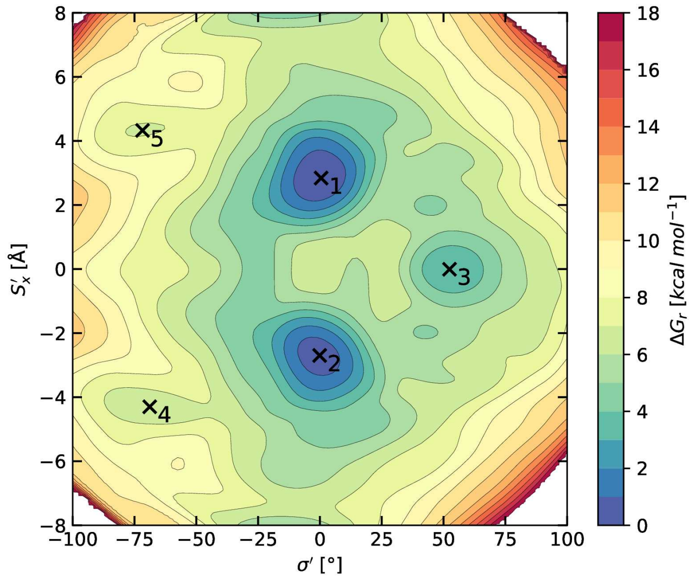

Figure SB8.1: Free energy surface for the aC/aC base pair. Labeled crosses show position of selected free energy minima (thermodynamic states). Free energy isolines are spaced by 1 kcal mol^{-1}.

Table SB8.1: Positions of selected free minima on the free energy surface for the aC/aC base pair. IDs correspond to the selected free energy minima shown in Figure SB8.1. Confidence interval of the free energy ΔG_r is provided at three standard deviations.

ID	σ' [°]	S'_x [Å]	ΔG_r [$kcal\ mol^{-1}$]
1	0.5	2.84	0.00±0.00
2	-0.1	-2.70	0.37±0.08
3	52.4	-0.01	3.05±0.07
4	-68.8	-4.30	6.29±0.10
5	-71.8	4.33	6.89±0.09

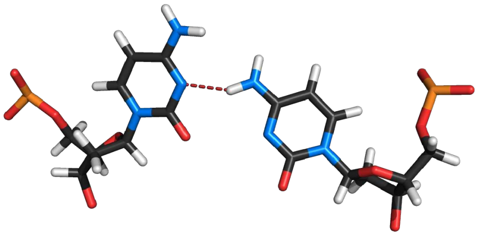

ID 1: $\sigma' = 0.5°$, $S'_x = 2.84$ Å

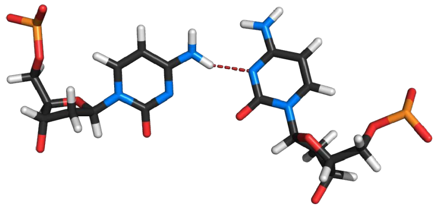

ID 2: $\sigma' = -0.1°$, $S'_x = -2.70$ Å

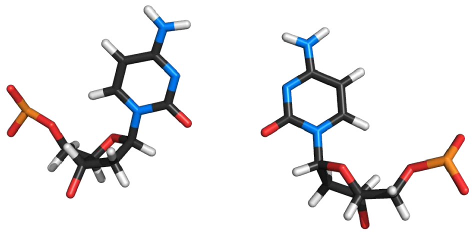

ID 3: $\sigma' = 52.4°$, $S'_x = -0.01$ Å

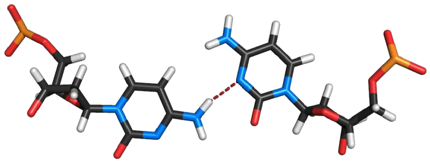

ID 4: $\sigma' = -68.8°$, $S'_x = -4.30$ Å

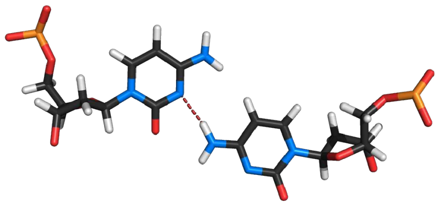

ID 5: $\sigma' = -71.8°$, $S'_x = 4.33$ Å

Figure SB8.2: Average geometries representing selected free energy minima for the aC/aC base pair. IDs correspond to the selected free energy minima shown in Figure SB8.1. Major and minor grooves are top and bottom, respectively. View direction is along the z-axis of DNA.

Table SB8.2: Number of analysed snapshots (N_{snap}), abundances (abu), average hydrogen bond distances and angles from hydrogen bond analysis provided by cpptraj for selected states of the aC/aC base pair. IDs correspond to the selected free energy minima shown in Figure SB8.1. Residue and atom numbering are provided in Figure SA1.

ID	N_{snap}	acceptor	H-donor	donor	abu [%]	d_{avg} [Å]	a_{avg} [°]
1	255	C7@N3	C20@H41	C20@N4	55.7	2.9	150.8
2	272	C20@N3	C7@H41	C7@N4	43.0	2.9	151.4
		C20@N3	C7@H42	C7@N4	1.1	3.0	153.6
3	748	—	—	—	—	—	—
4	387	C20@N3	C7@H42	C7@N4	47.8	2.9	158.9
		C20@N3	C7@H41	C7@N4	1.8	2.9	159.1
5	565	C7@N3	C20@H42	C20@N4	50.4	2.9	162.2

Table SB8.3: *Simple* base-pair parameters for selected states of the aC/aC base pair. IDs corresponds to the free energy minima shown in Figure SB8.1. Residue numbering in base pairs A/B are provided in Figure SA1. Abundances (*abu*), average values ($\langle X \rangle$) and standard deviations of samples ($s(X)$) are calculated by 3DNA for Shear (S_x), Stretch (S_y), Stagger (S_z), Buckle (κ), Propeller (π), Opening (σ) employing the standard reference frames for the nucleobases. Only five central base pairs were included in the analysis for each state. Base pairs with a mismatch are highlighted in gray.

ID	A/B	abu [%]	$\langle S_x \rangle$	$s(S_x)$	$\langle S_y \rangle$ [Å]	$s(S_y)$	$\langle S_z \rangle$	$s(S_z)$	$\langle \kappa \rangle$	$s(\kappa)$	$\langle \pi \rangle$ [°]	$s(\pi)$	$\langle \sigma \rangle$	$s(\sigma)$
	5/22	100.0	-0.23	0.25	0.03	0.12	-0.03	0.38	4.8	8.7	-16.7	7.2	0.8	4.7
	6/21	100.0	-0.08	0.28	-0.04	0.13	-0.29	0.39	-6.2	9.5	-23.9	7.0	2.6	5.4
1	7/20	100.0	2.83	0.21	-0.50	0.15	0.24	0.37	-19.8	10.2	-20.1	7.6	0.8	2.7
	8/19	99.6	0.09	0.25	0.12	0.16	-0.24	0.44	-15.6	8.8	-13.1	7.6	8.7	5.4
	9/18	100.0	0.18	0.28	0.04	0.12	0.07	0.40	-7.1	8.5	-13.3	7.1	-1.3	5.2
	5/22	100.0	-0.18	0.27	0.07	0.12	-0.04	0.37	5.4	9.0	-13.9	7.0	-0.6	5.4
	6/21	100.0	-0.12	0.27	0.05	0.13	-0.12	0.42	11.2	8.7	-10.9	8.5	8.1	5.2
2	7/20	100.0	-2.69	0.19	-0.53	0.13	0.12	0.42	18.9	10.8	-20.2	7.0	0.0	3.0
	8/19	100.0	0.18	0.30	-0.02	0.12	-0.22	0.39	7.2	9.0	-22.5	7.5	1.7	5.0
	9/18	100.0	0.18	0.26	0.03	0.12	0.03	0.36	-4.0	8.4	-17.5	8.0	1.0	5.5
	5/22	100.0	-0.19	0.26	0.03	0.12	-0.09	0.37	-0.9	9.1	-17.0	7.1	0.9	4.9
	6/21	100.0	-0.21	0.30	-0.03	0.13	-0.40	0.35	-10.3	10.3	-15.0	7.1	4.0	5.0
3	7/20	98.4	0.00	0.20	1.12	0.20	1.09	0.51	-0.3	10.9	-26.3	8.2	52.5	3.3
	8/19	100.0	0.18	0.52	-0.01	0.18	-0.38	0.34	9.1	10.8	-14.5	7.0	4.0	5.5
	9/18	100.0	0.19	0.27	0.03	0.12	-0.06	0.38	1.0	8.6	-17.6	6.6	-0.0	5.2
	5/22	100.0	-0.14	0.26	0.02	0.11	-0.02	0.43	-0.2	9.9	-17.8	8.0	0.2	4.9
	6/21	98.5	-0.11	0.30	0.02	0.18	0.03	0.58	-8.8	11.6	-16.2	11.5	1.1	7.1
4	7/20	100.0	-4.30	0.18	0.96	0.26	-0.81	0.90	-11.2	13.5	-1.8	16.1	-69.2	6.1
	8/19	100.0	0.12	0.43	0.02	0.19	-0.45	0.47	11.0	10.7	-13.0	10.1	-1.2	5.7
	9/18	100.0	0.15	0.27	0.02	0.12	-0.04	0.38	3.4	8.5	-17.2	6.8	-0.4	5.2
	5/22	99.7	-0.15	0.26	0.02	0.12	-0.04	0.40	-3.2	9.2	-18.6	7.3	-0.9	5.0
	6/21	100.0	-0.12	0.39	0.02	0.17	-0.40	0.46	-11.8	10.1	-14.7	10.4	-1.4	6.1
5	7/20	99.5	4.32	0.18	1.03	0.24	-0.57	0.96	9.1	13.6	0.4	17.7	-71.9	5.6
	8/19	99.7	0.10	0.34	0.03	0.18	0.01	0.61	6.1	13.5	-15.9	11.8	1.6	7.4
	9/18	100.0	0.17	0.27	0.03	0.12	0.02	0.41	0.5	9.4	-17.6	8.2	-1.0	5.6

Table SB8.4: *Simple* step parameters for selected states of the aC/aC base pair. IDs corresponds to the free energy minima shown in Figure SB8.1. Residue numbering in base pairs and steps A1-B1/A2-B2 are provided in Figure SA1. Abundances (*abu*), average values ($\langle X \rangle$) and standard deviations of samples ($s(X)$) are calculated by 3DNA for Shift (D_x), Slide (D_y), Rise (D_z), Tilt (τ), Roll (ρ), Twist (ω) employing the standard reference frames for the nucleobases. Only five central base pairs were included in the analysis for each state. Steps including a mismatch are highlighted in gray.

ID	A1-B1 A2-B2	abu [%]	$\langle D_x \rangle$	$s(D_x)$	$\langle D_y \rangle$ [Å]	$s(D_y)$	$\langle D_z \rangle$	$s(D_z)$	$\langle \tau \rangle$	$s(\tau)$	$\langle \rho \rangle$ [°]	$s(\rho)$	$\langle \omega \rangle$	$s(\omega)$
	5-6/21-22	100.0	0.23	0.49	-0.40	0.50	3.48	0.25	4.1	3.9	4.3	4.6	35.1	4.4
	6-7/20-21	100.0	-0.22	0.46	-0.03	0.43	4.04	0.38	-6.4	4.3	5.8	4.9	32.6	3.6
1	7-8/19-20	99.6	1.68	0.36	-0.24	0.35	2.82	0.35	13.3	6.0	-0.1	4.9	44.2	2.9
	7-9/18-20	0.4	1.77	0.00	-2.71	0.00	5.18	0.00	20.1	0.0	-3.1	0.0	62.3	0.0
	8-9/18-19	99.6	-0.45	0.78	-0.68	0.67	3.17	0.26	-1.9	4.3	-0.5	4.6	25.9	6.2
	5-6/21-22	100.0	1.16	0.79	-0.04	0.67	3.17	0.24	2.6	3.8	0.5	4.7	32.1	5.9
2	6-7/20-21	100.0	-1.66	0.38	-0.20	0.37	2.75	0.32	-10.1	6.0	-1.7	5.6	42.8	3.5
	7-8/19-20	100.0	0.51	0.52	0.05	0.43	3.98	0.41	6.1	4.6	4.7	5.1	33.2	3.3
	8-9/18-19	100.0	-0.26	0.56	-0.46	0.49	3.43	0.28	-4.5	3.9	3.9	5.0	36.6	4.7
	5-6/21-22	100.0	0.76	0.50	-0.10	0.45	3.44	0.30	5.6	3.8	4.0	5.3	38.5	3.8
	6-7/20-21	98.4	1.89	0.47	0.14	0.35	2.87	0.31	-5.5	5.1	9.7	4.6	28.4	4.8
3	6-8/19-21	1.6	-0.58	0.76	-1.04	1.63	5.64	0.68	-0.6	4.8	15.9	6.4	59.6	8.0
	7-8/19-20	98.4	-1.87	0.48	0.13	0.33	2.89	0.32	5.4	5.1	9.7	4.8	28.7	4.7
	8-9/18-19	100.0	-0.62	0.57	-0.17	0.54	3.45	0.31	-4.8	3.8	2.9	5.6	37.1	5.3
	5-6/21-22	98.5	0.50	0.63	-0.39	0.58	3.51	0.35	1.9	4.8	2.3	6.2	36.0	4.5
	6-7/20-21	83.7	-4.23	0.90	-0.80	1.04	3.54	0.55	0.6	5.7	12.8	10.3	37.1	5.4
4	6-8/19-21	14.5	-1.59	1.18	0.64	1.20	6.01	0.58	-5.0	6.7	29.3	16.8	58.8	8.9
	7-8/19-20	85.5	3.58	0.42	-1.03	0.45	3.28	0.44	0.6	5.1	9.8	7.2	22.3	6.9
	7-9/18-20	0.3	2.68	0.00	-3.40	0.00	6.26	0.00	-1.2	0.0	10.6	0.0	63.3	0.0
	8-9/18-19	85.8	-0.40	0.62	-0.27	0.49	3.43	0.31	-5.5	4.2	1.1	5.6	38.2	4.2
	5-6/21-22	99.7	0.34	0.61	-0.26	0.53	3.45	0.31	5.2	4.1	2.1	6.3	37.6	4.3
	6-7/20-21	95.8	-3.65	0.43	-1.03	0.43	3.29	0.43	-1.9	5.3	9.3	7.7	22.8	7.6
5	6-8/19-21	4.1	2.89	0.74	2.23	1.04	5.10	0.60	6.4	7.5	43.2	8.8	63.1	6.3
	7-8/19-20	88.0	4.67	0.90	-0.93	0.97	3.65	0.53	0.7	6.2	14.3	9.9	37.6	4.8
	8-9/18-19	82.3	-0.42	0.70	-0.53	0.61	3.48	0.37	-1.8	4.7	1.8	6.2	34.1	5.4

2.9 aC/aT

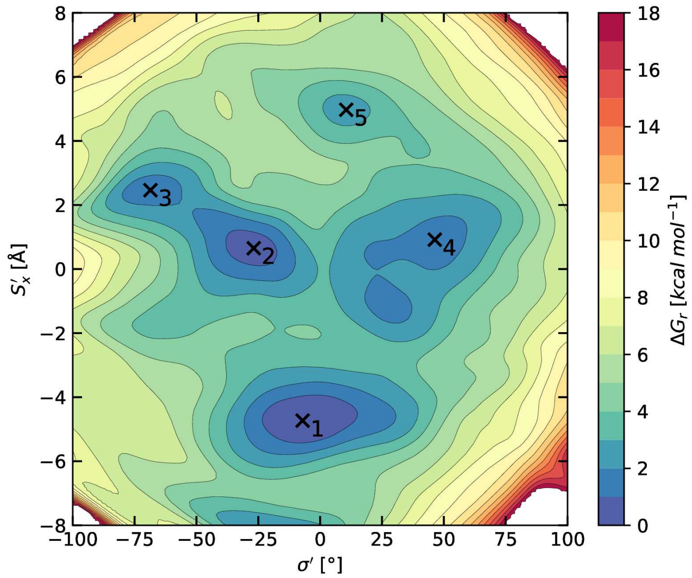

Figure SB9.1: Free energy surface for the aC/aT base pair. Labeled crosses show position of selected free energy minima (thermodynamic states). Free energy isolines are spaced by 1 kcal mol^{-1}.

Table SB9.1: Positions of selected free minima on the free energy surface for the aC/aT base pair. IDs correspond to the selected free energy minima shown in Figure SB9.1. Confidence interval of the free energy ΔG_r is provided at three standard deviations.

ID	σ' [°]	S'_x [Å]	ΔG_r [$kcal\ mol^{-1}$]
1	-7.1	-4.73	0.00±0.00
2	-26.8	0.65	0.46±0.08
3	-68.6	2.46	1.32±0.09
4	46.3	0.92	1.64±0.09
5	10.5	4.97	2.60±0.10

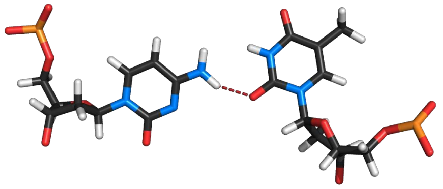

ID 1: σ' = -7.1°, S'_x = -4.73 Å

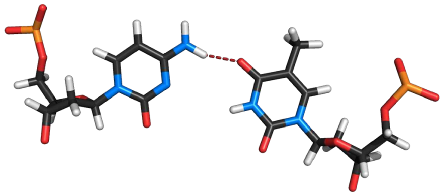

ID 2: σ' = -26.8°, S'_x = 0.65 Å

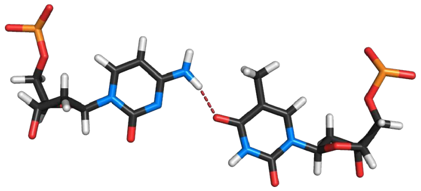

ID 3: σ' = -68.6°, S'_x = 2.46 Å

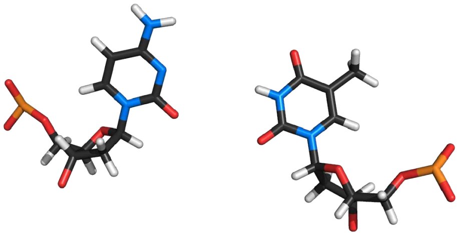

ID 4: σ' = 46.3°, S'_x = 0.92 Å

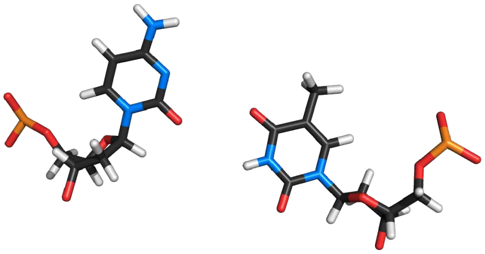

ID 5: σ' = 10.5°, S'_x = 4.97 Å

Figure SB9.2: Average geometries representing selected free energy minima for the aC/aT base pair. IDs correspond to the selected free energy minima shown in Figure SB9.1. Major and minor grooves are top and bottom, respectively. View direction is along the z-axis of DNA.

Table SB9.2: Number of analysed snapshots (N_{snap}), abundances (abu), average hydrogen bond distances and angles from hydrogen bond analysis provided by cpptraj for selected states of the aC/aT base pair. IDs correspond to the selected free energy minima shown in Figure SB9.1. Residue and atom numbering are provided in Figure SA1.

ID	N_{snap}	acceptor	H-donor	donor	abu [%]	d_{avg} [Å]	a_{avg} [°]
1	457	T20@O2	C7@H41	C7@N4	55.1	2.9	149.4
		T20@O2	C7@H42	C7@N4	3.5	2.8	147.9
2	386	T20@O4	C7@H41	C7@N4	54.7	2.9	153.6
		T20@O4	C7@H42	C7@N4	1.3	2.8	151.1
3	307	T20@O4	C7@H41	C7@N4	59.0	2.9	159.5
4	909	—	—	—	—	—	—
5	322	—	—	—	—	—	—

Table SB9.3: *Simple* base-pair parameters for selected states of the aC/aT base pair. IDs corresponds to the free energy minima shown in Figure SB9.1. Residue numbering in base pairs A/B are provided in Figure SA1. Abundances (*abu*), average values ($\langle X \rangle$) and standard deviations of samples ($s(X)$) are calculated by 3DNA for Shear (S_x), Stretch (S_y), Stagger (S_z), Buckle (κ), Propeller (π), Opening (σ) employing the standard reference frames for the nucleobases. Only five central base pairs were included in the analysis for each state. Base pairs with a mismatch are highlighted in gray.

ID	A/B	abu [%]	$\langle S_x \rangle$	$s(S_x)$	$\langle S_y \rangle$ [Å]	$s(S_y)$	$\langle S_z \rangle$	$s(S_z)$	$\langle \kappa \rangle$	$s(\kappa)$	$\langle \pi \rangle$ [°]	$s(\pi)$	$\langle \sigma \rangle$	$s(\sigma)$
	5/22	100.0	-0.17	0.27	0.04	0.13	0.04	0.41	1.1	9.1	-15.3	7.7	-0.2	5.2
	6/21	100.0	-0.09	0.27	0.00	0.12	-0.18	0.49	1.2	12.1	-11.4	8.8	3.9	5.9
1	7/20	100.0	-4.73	0.18	0.64	0.29	-0.08	0.55	3.5	10.8	-13.3	9.9	-6.7	4.5
	8/19	100.0	0.21	0.25	0.03	0.11	-0.04	0.39	11.6	9.4	-15.9	7.9	1.4	4.9
	9/18	100.0	0.20	0.26	0.03	0.12	-0.03	0.38	-0.2	8.9	-19.0	6.7	-0.3	5.1
	5/22	100.0	-0.19	0.27	0.04	0.12	-0.05	0.39	0.8	9.1	-16.2	7.7	0.3	5.1
	6/21	100.0	-0.20	0.28	0.03	0.13	-0.19	0.45	-4.8	11.2	-15.2	9.1	1.2	6.3
2	7/20	100.0	0.65	0.20	-0.16	0.22	-0.64	0.52	-1.8	10.1	-11.3	8.8	-26.8	3.7
	8/19	99.7	0.17	0.54	0.04	0.26	-0.00	0.44	9.2	10.3	-11.4	8.4	2.0	6.0
	9/18	100.0	0.17	0.27	0.03	0.12	0.00	0.38	1.7	9.7	-17.4	7.5	-0.4	5.1
	5/22	100.0	-0.15	0.27	0.03	0.11	0.03	0.39	-3.6	9.9	-18.9	7.6	-1.1	4.5
	6/21	100.0	-0.19	0.38	0.06	0.15	-0.01	0.43	-13.1	11.3	-17.5	10.1	-1.9	5.5
3	7/20	100.0	2.45	0.14	1.01	0.21	-0.35	0.78	-0.4	11.9	0.5	12.6	-68.9	3.6
	8/19	99.3	0.18	0.29	0.00	0.13	-0.31	0.43	5.4	10.1	-13.5	9.1	2.1	5.0
	9/18	100.0	0.20	0.26	0.03	0.11	-0.04	0.40	-0.8	8.8	-18.3	7.7	-0.6	5.0
	5/22	100.0	-0.22	0.26	0.04	0.12	-0.02	0.38	0.7	8.9	-17.7	6.9	-0.1	4.8
	6/21	100.0	-0.27	0.30	0.04	0.13	-0.12	0.42	-4.6	10.3	-13.8	7.7	2.4	5.5
4	7/20	5.3	0.73	0.31	1.08	0.61	1.15	0.54	1.8	11.2	-26.7	8.6	48.4	6.2
	8/19	100.0	0.19	0.29	0.02	0.13	-0.18	0.38	7.2	9.5	-12.8	7.7	2.6	5.2
	9/18	100.0	0.18	0.26	0.03	0.12	-0.02	0.38	0.2	9.3	-17.9	7.4	0.0	5.0
	5/22	100.0	-0.17	0.27	0.03	0.12	-0.06	0.37	0.1	9.4	-17.9	6.6	0.5	5.0
	6/21	100.0	-0.47	0.36	0.06	0.14	-0.11	0.44	-12.1	9.3	-19.2	8.1	0.1	6.0
5	7/20	95.0	4.98	0.21	1.49	0.30	-0.00	0.73	-1.5	11.6	-17.2	11.0	10.8	4.1
	8/19	98.1	0.14	0.30	-0.00	0.14	-0.18	0.46	5.3	10.4	-11.6	8.9	1.8	6.2
	8/20	1.9	-0.12	0.27	-0.22	0.18	0.63	0.69	-14.1	15.4	10.7	17.8	9.0	6.1
	9/18	100.0	0.20	0.29	0.04	0.13	0.03	0.38	1.8	9.3	-15.6	7.3	-0.2	5.3

Table SB9.4: *Simple* step parameters for selected states of the aC/aT base pair. IDs corresponds to the free energy minima shown in Figure SB9.1. Residue numbering in base pairs and steps A1-B1/A2-B2 are provided in Figure SA1. Abundances (*abu*), average values ($\langle X \rangle$) and standard deviations of samples ($s(X)$) are calculated by 3DNA for Shift (D_x), Slide (D_y), Rise (D_z), Tilt (τ), Roll (ρ), Twist (ω) employing the standard reference frames for the nucleobases. Only five central base pairs were included in the analysis for each state. Steps including a mismatch are highlighted in gray.

ID	A1-B1	abu	$\langle D_x \rangle$	$s(D_x)$	$\langle D_y \rangle$	$s(D_y)$	$\langle D_z \rangle$	$s(D_z)$	$\langle \tau \rangle$	$s(\tau)$	$\langle \rho \rangle$	$s(\rho)$	$\langle \omega \rangle$	$s(\omega)$
	A2-B2	[%]				[Å]						[°]		
	5-6/21-22	100.0	0.68	0.75	-0.29	0.62	3.30	0.29	3.5	4.1	-0.7	5.3	34.7	5.6
1	6-7/20-21	100.0	-0.96	0.91	-0.37	0.70	2.95	0.45	-5.3	6.5	8.9	9.5	41.5	9.0
	7-8/19-20	100.0	0.67	0.53	-0.11	0.40	3.57	0.37	1.8	4.6	4.0	5.5	22.2	3.6
	8-9/18-19	100.0	-0.75	0.50	-0.23	0.46	3.50	0.29	-3.9	3.9	2.5	5.3	39.9	3.2
	5-6/21-22	100.0	0.66	0.60	-0.32	0.51	3.38	0.30	3.7	4.2	1.2	5.7	37.2	4.5
	6-7/20-21	100.0	-2.43	0.71	-0.39	0.43	3.33	0.36	-2.7	5.9	2.9	6.7	32.3	5.4
2	7-8/19-20	99.7	2.12	0.73	-0.34	0.45	3.13	0.39	-1.6	4.8	1.8	6.7	30.4	4.9
	7-9/18-20	0.3	2.84	0.00	1.48	0.00	6.33	0.00	-1.4	0.0	9.8	0.0	74.5	0.0
	8-9/18-19	99.7	-0.91	0.58	-0.16	0.47	3.45	0.30	-3.2	4.2	-0.3	5.6	39.2	3.9
	5-6/21-22	100.0	0.40	0.57	-0.27	0.52	3.48	0.30	3.0	4.1	1.7	6.0	38.5	4.5
	6-7/20-21	99.7	-3.94	0.62	-0.71	0.54	3.41	0.42	-1.7	5.5	7.1	8.2	27.9	7.1
3	6-8/19-21	0.3	2.30	0.00	2.41	0.00	5.81	0.00	0.5	0.0	43.8	0.0	60.8	0.0
	7-8/19-20	97.7	4.77	0.84	0.07	0.44	3.59	0.52	2.4	5.5	8.7	11.7	30.4	5.0
	8-9/18-19	86.0	-0.78	0.48	-0.02	0.46	3.39	0.29	-5.0	4.3	-1.0	5.8	40.2	3.6
	5-6/21-22	100.0	0.71	0.55	-0.12	0.45	3.37	0.27	3.5	3.8	1.4	5.2	38.9	3.7
	6-7/20-21	5.3	1.61	0.77	0.39	0.44	3.23	0.35	-3.5	4.7	8.7	7.4	28.1	6.1
4	6-8/19-21	94.6	-0.22	0.77	-1.02	0.58	6.20	0.40	-0.5	5.9	20.4	6.6	56.3	6.3
	7-8/19-20	5.3	-2.00	0.59	-0.40	0.32	2.90	0.41	3.0	4.8	13.1	7.2	26.8	4.3
	8-9/18-19	100.0	-0.69	0.58	-0.20	0.50	3.41	0.28	-4.1	3.9	1.8	5.2	38.3	4.0
	5-6/21-22	100.0	0.67	0.54	-0.27	0.42	3.47	0.31	4.2	4.2	4.2	5.3	39.7	3.5
	6-7/20-21	95.0	0.12	0.62	0.40	0.44	3.52	0.34	0.1	5.8	5.6	4.2	20.5	4.2
	6-8/19-21	3.1	-0.82	0.99	-0.81	0.58	5.82	0.55	-2.9	6.5	13.8	9.7	56.5	8.6
5	6-8/20-21	1.9	-2.55	0.34	-1.04	0.43	3.65	0.50	4.1	7.3	13.9	3.8	53.3	3.8
	7-8/19-20	95.0	-0.46	1.26	-0.94	0.45	2.68	0.42	-0.4	5.7	10.0	9.8	38.4	8.7
	8-9/18-19	98.1	-0.52	0.71	-0.38	0.57	3.36	0.27	-3.5	4.2	-0.2	5.6	36.1	4.9
	8-9/18-20	1.9	0.64	0.61	-0.01	0.27	4.78	0.36	-13.8	7.7	-4.7	5.6	47.1	5.0

2.10 aT/aT

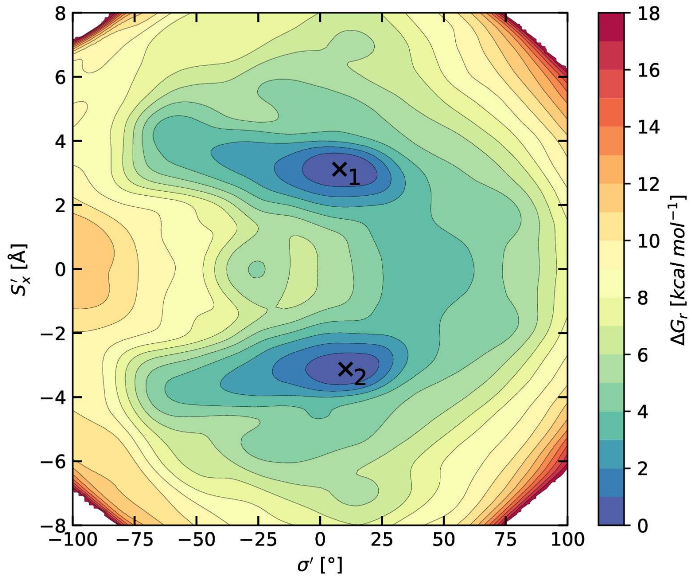

Figure SB10.1: Free energy surface for the aT/aT base pair. Labeled crosses show position of selected free energy minima (thermodynamic states). Free energy isolines are spaced by 1 kcal mol^{-1}.

Table SB10.1: Positions of selected free minima on the free energy surface for the aT/aT base pair. IDs correspond to the selected free energy minima shown in Figure SB10.1. Confidence interval of the free energy ΔG_r is provided at three standard deviations.

ID	σ' [°]	S'_x [Å]	ΔG_r [$kcal\ mol^{-1}$]
1	7.8	3.12	0.00±0.00
2	10.3	-3.12	0.10±0.10

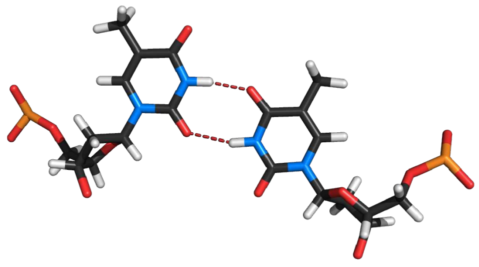

ID 1: $\sigma' = 7.8°$, $S'_x = 3.12$ Å

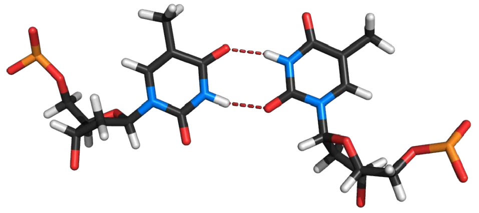

ID 2: $\sigma' = 10.3°$, $S'_x = -3.12$ Å

Figure SB10.2: Average geometries representing selected free energy minima for the aT/aT base pair. IDs correspond to the selected free energy minima shown in Figure SB10.1. Major and minor grooves are top and bottom, respectively. View direction is along the z-axis of DNA.

Table SB10.2: Number of analysed snapshots (N_{snap}), abundances (abu), average hydrogen bond distances and angles from hydrogen bond analysis provided by cpptraj for selected states of the aT/aT base pair. IDs correspond to the selected free energy minima shown in Figure SB10.1. Residue and atom numbering are provided in Figure SA1.

ID	N_{snap}	acceptor	H-donor	donor	abu [%]	d_{avg} [Å]	a_{avg} [°]
1	233	T20@O4	T7@H3	T7@N3	42.1	2.9	163.0
		T7@O2	T20@H3	T20@N3	13.7	2.9	160.9
2	447	T7@O4	T20@H3	T20@N3	38.3	2.9	159.7
		T20@O2	T7@H3	T7@N3	26.2	2.9	160.8

Table SB10.3: *Simple* base-pair parameters for selected states of the aT/aT base pair. IDs corresponds to the free energy minima shown in Figure SB10.1. Residue numbering in base pairs A/B are provided in Figure SA1. Abundances (*abu*), average values ($\langle X \rangle$) and standard deviations of samples ($s(X)$) are calculated by 3DNA for Shear (S_x), Stretch (S_y), Stagger (S_z), Buckle (κ), Propeller (π), Opening (σ) employing the standard reference frames for the nucleobases. Only five central base pairs were included in the analysis for each state. Base pairs with a mismatch are highlighted in gray.

ID	A/B	abu [%]	$\langle S_x \rangle$	$s(S_x)$	$\langle S_y \rangle$ [Å]	$s(S_y)$	$\langle S_z \rangle$	$s(S_z)$	$\langle \kappa \rangle$	$s(\kappa)$	$\langle \pi \rangle$ [°]	$s(\pi)$	$\langle \sigma \rangle$	$s(\sigma)$
	5/22	100.0	-0.20	0.29	0.04	0.13	-0.12	0.39	0.6	8.1	-17.0	6.6	0.6	4.7
	6/21	100.0	-0.20	0.28	-0.01	0.12	-0.24	0.39	-10.5	9.5	-16.1	6.6	5.2	4.7
1	7/20	100.0	3.10	0.14	-0.64	0.11	0.22	0.35	1.3	11.6	-6.4	9.9	7.7	4.3
	8/19	100.0	0.01	0.28	-0.03	0.13	-0.43	0.48	-4.3	11.6	-8.6	8.0	1.9	5.2
	9/18	100.0	0.14	0.27	0.03	0.13	-0.10	0.40	-3.2	9.2	-10.1	8.0	-0.2	4.7
	5/22	100.0	-0.15	0.27	0.04	0.11	-0.07	0.39	1.4	8.7	-11.3	8.4	-0.2	5.0
	6/21	100.0	-0.03	0.35	-0.02	0.14	-0.40	0.46	1.5	11.1	-9.0	7.4	1.4	5.3
2	7/20	100.0	-3.11	0.14	-0.62	0.13	0.23	0.36	3.1	12.0	-11.6	10.4	10.3	4.2
	8/19	100.0	0.18	0.27	-0.01	0.12	-0.18	0.40	9.5	10.5	-17.0	7.3	4.3	5.4
	9/18	100.0	0.20	0.26	0.03	0.12	-0.04	0.37	-0.3	8.7	-17.6	6.6	0.6	4.7

Table SB10.4: *Simple* step parameters for selected states of the aT/aT base pair. IDs corresponds to the free energy minima shown in Figure SB10.1. Residue numbering in base pairs and steps A1-B1/A2-B2 are provided in Figure SA1. Abundances (*abu*), average values ($\langle X \rangle$) and standard deviations of samples ($s(X)$) are calculated by 3DNA for Shift (D_x), Slide (D_y), Rise (D_z), Tilt (τ), Roll (ρ), Twist (ω) employing the standard reference frames for the nucleobases. Only five central base pairs were included in the analysis for each state. Steps including a mismatch are highlighted in gray.

ID	A1-B1<br>A2-B2	abu<br>[%]	$\langle D_x \rangle$	$s(D_x)$	$\langle D_y \rangle$<br>[Å]	$s(D_y)$	$\langle D_z \rangle$	$s(D_z)$	$\langle \tau \rangle$	$s(\tau)$	$\langle \rho \rangle$<br>[°]	$s(\rho)$	$\langle \omega \rangle$	$s(\omega)$
1	5-6/21-22	100.0	0.93	0.53	-0.01	0.46	3.47	0.29	4.8	3.7	3.5	5.1	38.8	3.6
	6-7/20-21	100.0	0.23	0.43	0.39	0.39	3.29	0.37	-4.3	4.0	1.1	4.5	24.7	3.7
	7-8/19-20	100.0	-0.93	0.64	-0.93	0.56	3.39	0.52	2.8	5.1	3.0	5.8	46.8	4.7
	8-9/18-19	100.0	-0.19	0.51	-0.81	0.51	3.34	0.29	-2.8	4.1	-0.8	4.7	30.7	4.5
2	5-6/21-22	100.0	0.25	0.58	-0.75	0.54	3.35	0.30	3.1	4.0	-0.7	4.9	30.7	5.2
	6-7/20-21	100.0	0.72	0.64	-0.90	0.51	3.18	0.53	-4.3	5.3	5.8	6.3	43.4	6.5
	7-8/19-20	100.0	-0.33	0.53	0.40	0.45	3.46	0.41	3.4	4.4	1.8	4.7	25.8	4.4
	8-9/18-19	100.0	-0.84	0.57	-0.07	0.47	3.46	0.30	-4.7	4.0	3.4	5.4	38.4	4.0

3 Base Pairs in the *anti/syn* Orientations

3.1 aA/sA

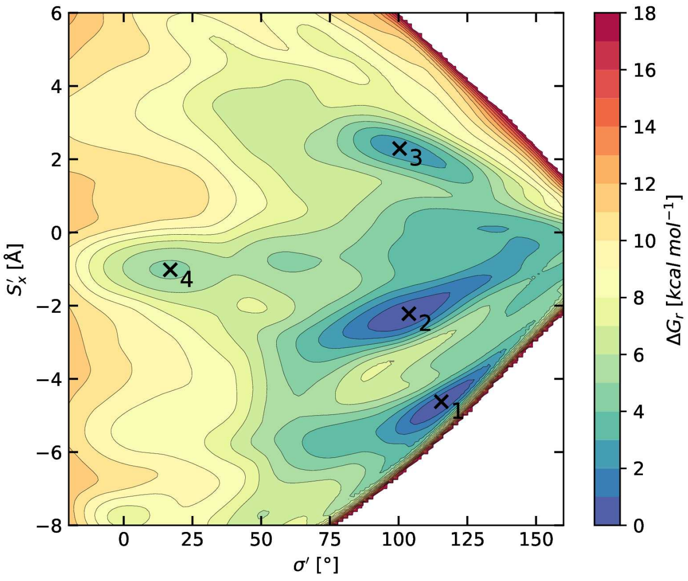

Figure SB11.1: Free energy surface for the aA/sA base pair. Labeled crosses show position of selected free energy minima (thermodynamic states). Free energy isolines are spaced by 1 kcal mol^{-1}.

Table SB11.1: Positions of selected free minima on the free energy surface for the aA/sA base pair. IDs correspond to the selected free energy minima shown in Figure SB11.1. Confidence interval of the free energy ΔG_r is provided at three standard deviations.

ID	σ' [°]	S_x' [Å]	ΔG_r [$kcal\ mol^{-1}$]
1	115.5	-4.62	0.00±0.00
2	103.7	-2.22	0.09±0.17
3	100.4	2.29	2.36±0.20
4	16.9	-1.02	4.76±0.18

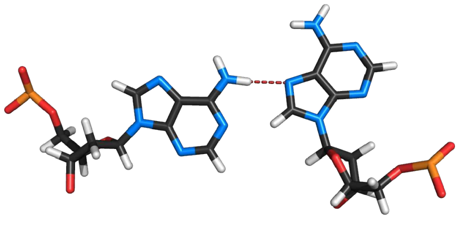

ID 1: $\sigma' = 115.5°$, S'_x = -4.62 Å

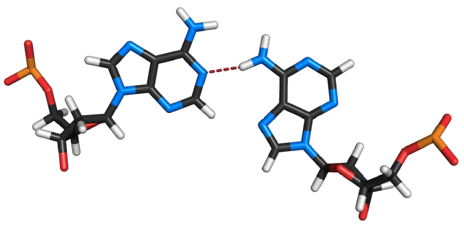

ID 2: $\sigma' = 103.7°$, S'_x = -2.22 Å

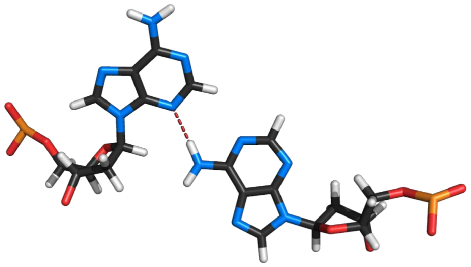

ID 3: $\sigma' = 100.4°$, S'_x = 2.29 Å

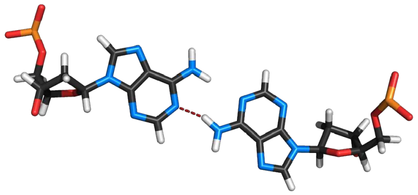

ID 4: $\sigma' = 16.9°$, S'_x = -1.02 Å

Figure SB11.2: Average geometries representing selected free energy minima for the aA/sA base pair. IDs correspond to the selected free energy minima shown in Figure SB11.1. Major and minor grooves are top and bottom, respectively. View direction is along the z-axis of DNA.

Table SB11.2: Number of analysed snapshots (N_{snap}), abundances (abu), average hydrogen bond distances and angles from hydrogen bond analysis provided by cpptraj for selected states of the aA/sA base pair. IDs correspond to the selected free energy minima shown in Figure SB11.1. Residue and atom numbering are provided in Figure SA1.

ID	N_{snap}	acceptor	H-donor	donor	abu [%]	d_{avg} [Å]	a_{avg} [°]
1	173	A20@N7	A7@H61	A7@N6	23.7	2.9	159.0
2	228	A7@N1	A20@H62	A20@N6	62.3	2.9	157.0
3	228	A7@N3	A20@H61	A20@N6	49.6	2.9	164.4
4	250	A7@N1	A20@H61	A20@N6	76.4	2.9	154.9
		A20@N1	A7@H61	A7@N6	8.0	2.9	162.4

Table SB11.3: *Simple* base-pair parameters for selected states of the aA/sA base pair. IDs corresponds to the free energy minima shown in Figure SB11.1. Residue numbering in base pairs A/B are provided in Figure SA1. Abundances (*abu*), average values ($\langle X \rangle$) and standard deviations of samples ($s(X)$) are calculated by 3DNA for Shear (S_x), Stretch (S_y), Stagger (S_z), Buckle (κ), Propeller (π), Opening (σ) employing the standard reference frames for the nucleobases. Only five central base pairs were included in the analysis for each state. Base pairs with a mismatch are highlighted in gray.

ID	A/B	abu [%]	$\langle S_x \rangle$	$s(S_x)$	$\langle S_y \rangle$	$s(S_y)$ [Å]	$\langle S_z \rangle$	$s(S_z)$	$\langle \kappa \rangle$	$s(\kappa)$	$\langle \pi \rangle$	$s(\pi)$ [°]	$\langle \sigma \rangle$	$s(\sigma)$
	5/22	100.0	-0.17	0.28	0.03	0.13	0.00	0.41	6.2	9.0	-13.8	7.2	0.8	5.2
	6/21	100.0	-0.01	0.25	0.01	0.13	-0.27	0.42	9.6	10.6	-12.6	8.3	2.1	5.2
1	7/20	100.0	-4.61	0.16	5.08	0.26	0.81	0.58	-6.3	11.5	-8.3	9.5	-64.4	3.2
	8/19	100.0	0.11	0.25	0.01	0.13	0.14	0.52	1.9	11.1	-9.2	9.2	2.7	5.2
	9/18	100.0	0.19	0.28	0.03	0.12	0.09	0.41	-0.1	10.0	-18.1	8.1	-1.2	5.2
	5/22	100.0	-0.16	0.26	0.04	0.13	0.00	0.40	0.4	9.5	-17.0	7.2	0.1	5.0
	6/21	100.0	-0.14	0.28	0.05	0.12	-0.02	0.39	-1.9	11.6	-12.8	9.1	1.9	5.6
2	7/20	100.0	-2.21	0.14	3.36	0.21	0.09	0.54	3.7	10.9	-7.7	10.2	-76.2	3.6
	8/19	100.0	0.16	0.28	0.03	0.12	-0.07	0.39	4.4	10.0	-17.4	7.5	2.1	5.6
	9/18	100.0	0.17	0.26	0.04	0.12	-0.09	0.36	-2.8	9.2	-17.2	6.6	0.3	5.1
	5/22	100.0	-0.17	0.26	0.02	0.12	-0.04	0.41	-0.8	9.7	-17.4	7.5	-0.6	4.5
	6/21	100.0	-0.05	0.49	0.04	0.16	-0.21	0.48	-4.1	12.5	-12.6	9.2	-1.1	6.2
3	7/20	100.0	2.29	0.12	4.07	0.24	-0.17	0.58	14.4	14.2	-8.3	11.7	-79.4	4.0
	8/19	100.0	-0.09	0.98	0.05	0.29	0.12	0.53	2.6	11.6	-11.3	9.8	2.1	7.1
	9/18	100.0	0.19	0.28	0.05	0.12	0.03	0.40	-1.5	9.9	-16.2	7.4	-0.9	5.2
	5/22	100.0	-0.15	0.26	0.03	0.13	0.12	0.38	-7.6	10.0	-19.9	7.4	-1.7	5.3
	6/21	99.2	-0.21	0.34	0.14	0.29	0.08	0.44	-13.0	11.2	-21.0	11.4	-4.9	9.3
4	7/20	100.0	-1.01	0.14	1.67	0.21	-0.78	0.65	1.0	13.6	-6.4	20.9	-162.7	3.9
	8/19	100.0	0.25	0.30	0.06	0.14	0.06	0.48	7.9	10.7	-17.2	9.6	-2.9	6.1
	9/18	100.0	0.14	0.30	0.02	0.13	0.15	0.42	2.1	9.6	-19.1	7.9	-1.0	5.3

Table SB11.4: *Simple* step parameters for selected states of the aA/sA base pair. IDs corresponds to the free energy minima shown in Figure SB11.1. Residue numbering in base pairs and steps A1-B1/A2-B2 are provided in Figure SA1. Abundances (abu), average values ($\langle X \rangle$) and standard deviations of samples ($s(X)$) are calculated by 3DNA for Shift (D_x), Slide (D_y), Rise (D_z), Tilt (τ), Roll (ρ), Twist (ω) employing the standard reference frames for the nucleobases. Only five central base pairs were included in the analysis for each state. Steps including a mismatch are highlighted in gray.

ID	A1-B1 A2-B2	abu [%]	$\langle D_x \rangle$	$s(D_x)$	$\langle D_y \rangle$ [Å]	$s(D_y)$	$\langle D_z \rangle$	$s(D_z)$	$\langle \tau \rangle$	$s(\tau)$	$\langle \rho \rangle$ [°]	$s(\rho)$	$\langle \omega \rangle$	$s(\omega)$
1	5-6/21-22	100.0	0.26	0.50	-0.69	0.48	3.27	0.24	1.7	4.4	-1.1	5.0	32.8	4.1
	6-7/20-21	100.0	2.96	0.65	-2.96	0.38	2.73	0.41	-0.2	5.5	14.8	7.7	43.6	6.3
	7-8/19-20	100.0	-1.11	0.52	3.19	0.55	3.56	0.35	0.1	5.4	5.5	4.8	23.6	4.7
	8-9/18-19	100.0	-0.55	0.67	-0.23	0.59	3.41	0.29	-1.6	4.2	-0.4	5.2	35.9	5.1
2	5-6/21-22	100.0	0.77	0.56	-0.20	0.53	3.33	0.27	2.3	4.5	1.2	5.3	37.4	4.5
	6-7/20-21	100.0	1.67	0.62	-0.81	0.71	3.20	0.31	1.3	4.7	4.2	5.7	25.7	5.7
	7-8/19-20	100.0	-0.92	0.64	1.00	0.58	3.19	0.26	0.2	4.6	6.5	6.6	34.7	5.5
	8-9/18-19	100.0	-0.78	0.57	-0.28	0.56	3.41	0.28	-2.7	4.4	1.0	5.5	37.1	4.2
3	5-6/21-22	100.0	0.35	0.58	-0.40	0.51	3.39	0.29	2.6	4.1	1.4	5.4	36.8	4.3
	6-7/20-21	100.0	1.55	0.60	1.43	0.62	3.49	0.31	3.5	4.9	1.4	4.7	15.4	4.5
	7-8/19-20	98.2	-0.90	0.67	-1.77	0.79	2.70	0.41	-3.3	8.1	23.8	8.9	38.9	7.4
	8-9/18-19	100.0	-0.84	0.70	-0.38	0.61	3.45	0.32	-1.8	4.5	-0.8	6.2	33.7	5.8
4	5-6/21-22	99.2	-0.05	0.73	-0.50	0.51	3.40	0.31	0.4	4.6	4.2	6.7	37.4	4.0
	5-7/20-22	0.8	-2.59	0.89	1.29	0.07	7.15	0.28	-7.7	5.2	32.9	4.5	48.0	14.2
	6-7/20-21	98.4	-1.61	0.65	-0.37	0.47	3.11	0.34	3.7	5.2	17.6	9.1	19.8	7.3
	6-8/19-21	0.8	2.08	1.02	1.98	0.79	4.96	0.34	4.2	1.5	47.1	16.7	47.5	19.6
	7-8/19-20	99.2	1.31	1.05	1.09	0.59	3.18	0.34	-4.9	5.5	10.7	11.9	28.8	6.6
	8-9/18-19	99.2	-0.19	0.64	-0.33	0.52	3.38	0.31	-3.1	4.3	1.0	5.8	38.0	4.4

3.2 aA/sC

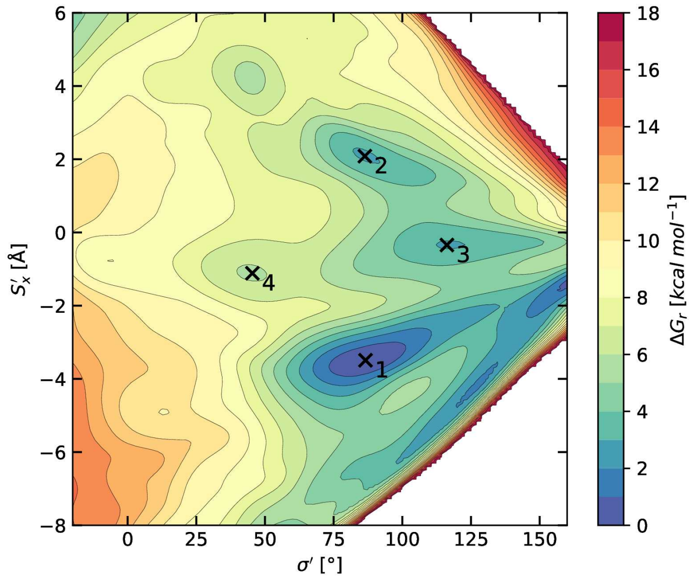

Figure SB12.1: Free energy surface for the aA/sC base pair. Labeled crosses show position of selected free energy minima (thermodynamic states). Free energy isolines are spaced by 1 kcal mol^{-1}.

Table SB12.1: Positions of selected free minima on the free energy surface for the aA/sC base pair. IDs correspond to the selected free energy minima shown in Figure SB12.1. Confidence interval of the free energy ΔG_r is provided at three standard deviations.

ID	σ' [°]	S'_x [Å]	ΔG_r [$kcal\ mol^{-1}$]
1	86.6	-3.49	0.00±0.00
2	86.4	2.08	2.88±0.16
3	116.2	-0.34	2.94±0.16
4	45.4	-1.11	5.90±0.13

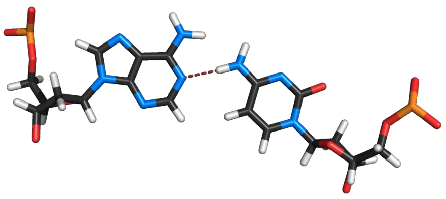

ID 1: $\sigma' = 86.6°$, S'_x = -3.49 Å

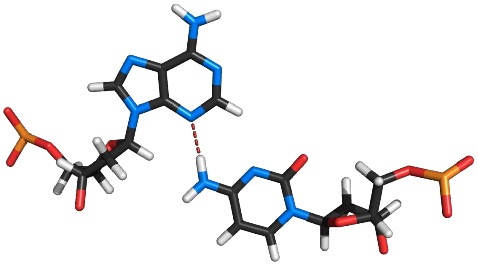

ID 2: $\sigma' = 86.4°$, S'_x = 2.08 Å

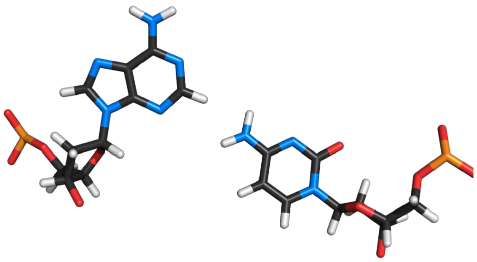

ID 3: $\sigma' = 116.2°$, S'_x = -0.34 Å

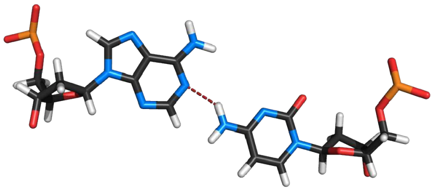

ID 4: $\sigma' = 45.4°$, S'_x = -1.11 Å

Figure SB12.2: Average geometries representing selected free energy minima for the aA/sC base pair. IDs correspond to the selected free energy minima shown in Figure SB12.1. Major and minor grooves are top and bottom, respectively. View direction is along the z-axis of DNA.

Table SB12.2: Number of analysed snapshots (N_{snap}), abundances (abu), average hydrogen bond distances and angles from hydrogen bond analysis provided by cpptraj for selected states of the aA/sC base pair. IDs correspond to the selected free energy minima shown in Figure SB12.1. Residue and atom numbering are provided in Figure SA1.

ID	N_{snap}	acceptor	H-donor	donor	abu [%]	d_{avg} [Å]	a_{avg} [°]
1	289	A7@N1	C20@H42	C20@N4	56.4	2.9	163.1
		C20@N4	A7@H61	A7@N6	3.1	2.9	147.7
2	328	A7@N3	C20@H41	C20@N4	55.5	2.9	161.7
3	463	—	—	—	—	—	—
4	436	A7@N1	C20@H41	C20@N4	24.1	2.9	143.3

Table SB12.3: *Simple* base-pair parameters for selected states of the aA/sC base pair. IDs corresponds to the free energy minima shown in Figure SB12.1. Residue numbering in base pairs A/B are provided in Figure SA1. Abundances (*abu*), average values ($\langle X \rangle$) and standard deviations of samples ($s(X)$) are calculated by 3DNA for Shear (S_x), Stretch (S_y), Stagger (S_z), Buckle (κ), Propeller (π), Opening (σ) employing the standard reference frames for the nucleobases. Only five central base pairs were included in the analysis for each state. Base pairs with a mismatch are highlighted in gray.

ID	A/B	abu	$\langle S_x \rangle$	$s(S_x)$	$\langle S_y \rangle$	$s(S_y)$	$\langle S_z \rangle$	$s(S_z)$	$\langle \kappa \rangle$	$s(\kappa)$	$\langle \pi \rangle$	$s(\pi)$	$\langle \sigma \rangle$	$s(\sigma)$
		[%]			[Å]						[°]			
	5/22	100.0	-0.21	0.24	0.03	0.11	-0.00	0.37	-1.4	8.9	-18.8	6.4	-0.1	4.7
	6/21	100.0	-0.21	0.27	0.06	0.12	-0.04	0.39	-5.3	9.0	-11.2	8.8	-0.3	5.3
1	7/20	100.0	-3.48	0.11	3.07	0.21	-0.15	0.56	-1.8	9.6	-5.4	9.5	-93.1	3.6
	8/19	100.0	0.14	0.27	0.04	0.13	0.10	0.38	7.8	9.5	-16.9	7.5	-0.1	6.3
	9/18	100.0	0.20	0.26	0.04	0.12	0.03	0.37	-1.3	9.3	-18.9	7.0	-0.4	4.8
	5/22	100.0	-0.23	0.26	0.04	0.11	-0.02	0.39	-3.1	9.3	-17.5	7.4	-0.5	4.6
	6/21	100.0	-0.05	0.58	0.02	0.20	-0.26	0.42	-9.5	10.2	-10.3	9.5	-1.6	5.5
2	7/19	23.2	0.32	0.27	0.00	0.17	-0.48	0.76	-1.1	9.2	-31.2	15.2	2.4	4.9
	7/20	76.8	2.06	0.17	2.38	0.28	-0.01	0.76	6.3	12.8	-3.4	12.0	-93.0	3.7
	8/19	62.5	-0.59	1.53	0.12	0.58	-0.64	0.70	-4.7	12.1	-9.0	9.2	6.1	7.4
	9/18	100.0	-0.37	1.51	0.19	0.54	-0.28	0.55	-6.0	11.3	-7.1	12.6	2.5	6.6
	5/22	100.0	-0.16	0.27	0.03	0.12	-0.06	0.40	-0.6	9.8	-19.9	7.1	0.2	4.9
	6/21	100.0	-0.23	0.27	0.03	0.14	0.01	0.50	-10.2	9.8	-17.8	9.7	0.3	6.2
3	7/20	17.5	-0.32	0.13	4.70	0.43	0.24	0.91	0.9	12.0	-15.4	16.0	-64.8	4.8
	8/19	81.9	0.08	0.29	0.02	0.12	0.11	0.43	4.3	10.4	-16.3	7.5	1.7	5.9
	8/20	4.1	-0.02	0.11	6.12	0.36	2.04	0.39	-3.0	10.8	-19.4	7.1	-18.6	4.6
	9/18	99.8	-0.57	1.75	0.25	0.58	-0.11	0.58	-2.4	9.4	-14.2	12.4	-0.7	7.0
	5/22	100.0	-0.21	0.28	0.04	0.20	0.01	0.39	-5.2	9.8	-20.6	7.2	-1.8	6.6
	6/21	100.0	0.02	1.01	0.25	0.47	-0.10	0.56	-13.0	11.6	-16.9	11.2	-5.6	12.5
4	7/20	99.8	-1.11	0.19	1.50	0.34	-0.30	0.75	-1.8	16.2	-7.3	26.5	-134.4	4.7
	8/19	100.0	0.20	0.28	0.04	0.12	-0.04	0.45	2.7	11.5	-13.5	9.7	0.2	5.5
	9/18	100.0	0.21	0.26	0.04	0.13	-0.01	0.38	-1.9	9.4	-18.7	6.9	-0.8	5.0

Table SB12.4: *Simple* step parameters for selected states of the aA/sC base pair. IDs corresponds to the free energy minima shown in Figure SB12.1. Residue numbering in base pairs and steps A1-B1/A2-B2 are provided in Figure SA1. Abundances (*abu*), average values ($\langle X \rangle$) and standard deviations of samples ($s(X)$) are calculated by 3DNA for Shift (D_x), Slide (D_y), Rise (D_z), Tilt (τ), Roll (ρ), Twist (ω) employing the standard reference frames for the nucleobases. Only five central base pairs were included in the analysis for each state. Steps including a mismatch are highlighted in gray.

ID	A1-B1 A2-B2	abu [%]	$\langle D_x \rangle$	$s(D_x)$	$\langle D_y \rangle$ [Å]	$s(D_y)$	$\langle D_z \rangle$	$s(D_z)$	$\langle \tau \rangle$	$s(\tau)$	$\langle \rho \rangle$ [°]	$s(\rho)$	$\langle \omega \rangle$	$s(\omega)$
1	5-6/21-22	100.0	0.77	0.55	-0.25	0.45	3.36	0.28	2.9	4.1	-0.5	5.2	38.3	3.8
	6-7/20-21	100.0	0.59	0.65	-0.80	0.51	3.19	0.30	-0.3	4.8	1.3	6.1	30.9	4.4
	7-8/19-20	100.0	-0.77	0.49	1.22	0.53	3.14	0.29	-3.6	4.3	5.9	6.6	28.9	4.8
	8-9/18-19	100.0	-0.64	0.62	-0.34	0.53	3.46	0.27	-2.4	4.0	-0.6	5.9	38.7	4.1
2	5-6/21-22	100.0	0.50	0.58	-0.24	0.49	3.46	0.30	4.2	4.0	0.1	5.2	37.1	3.6
	6-7/19-21	23.2	2.84	0.62	0.10	0.60	3.84	0.39	-8.9	6.1	20.7	10.6	47.1	6.4
	6-7/20-21	76.8	1.89	0.55	0.83	0.46	3.36	0.30	-1.1	4.7	0.9	4.8	15.6	5.5
	7-8/19-20	62.2	-1.25	0.97	-1.34	0.77	3.88	0.72	-1.0	7.2	7.1	11.6	49.0	7.4
	7-9/18-19	23.2	-2.44	0.58	-1.14	0.51	4.74	0.81	14.1	6.5	8.4	10.4	53.9	4.4
	7-9/18-20	2.7	0.01	0.87	-3.32	0.53	4.79	1.16	10.3	10.7	44.1	22.3	83.5	9.4
	8-9/18-19	62.5	-0.66	0.62	-0.62	0.64	3.41	0.33	-3.7	4.5	2.0	5.9	27.5	6.2
3	5-6/21-22	100.0	0.49	0.54	-0.28	0.52	3.50	0.33	1.8	4.0	3.6	6.6	38.3	4.4
	6-7/20-21	17.5	2.59	0.57	0.68	0.56	3.30	0.30	4.5	7.3	2.6	5.8	22.1	6.2
	6-8/19-21	69.3	-0.57	0.73	-0.82	0.80	6.21	0.40	-1.3	5.8	9.6	9.3	63.2	7.9
	6-8/20-21	4.1	2.04	0.47	1.46	0.64	5.16	0.47	13.2	5.2	-0.7	3.5	34.0	4.8
	7-8/19-20	12.1	-3.01	0.75	0.27	0.84	2.91	0.33	-3.3	7.2	5.7	10.3	41.9	9.5
	7-9/18-20	0.4	-3.14	0.64	-4.89	0.50	2.92	0.11	-13.9	0.3	20.1	5.3	76.0	1.9
	8-9/18-19	81.9	-0.43	0.57	-0.56	0.57	3.43	0.30	-1.9	4.1	-0.5	4.9	35.0	4.5
	8-9/18-20	0.4	-3.45	0.25	-4.97	0.06	4.28	0.71	-36.3	2.0	-1.4	3.5	67.7	0.9
4	5-6/21-22	99.8	0.19	0.97	-0.33	0.52	3.47	0.34	2.8	4.5	2.8	6.2	37.2	4.4
	5-7/20-22	0.2	-1.30	0.00	1.15	0.00	6.38	0.00	3.3	0.0	21.9	0.0	44.4	0.0
	6-7/20-21	99.5	-0.30	0.99	0.33	0.68	3.10	0.48	0.8	5.9	9.2	9.5	23.3	9.6
	6-8/19-21	0.2	1.70	0.00	1.54	0.00	6.70	0.00	3.6	0.0	21.6	0.0	47.3	0.0
	7-8/19-20	99.5	1.10	0.90	1.10	0.76	3.36	0.43	-2.8	7.2	8.9	13.2	32.2	6.7
	8-9/18-19	100.0	-0.65	0.59	-0.11	0.56	3.38	0.29	-3.0	4.0	-0.8	5.8	38.4	4.7

3.3 aA/sG

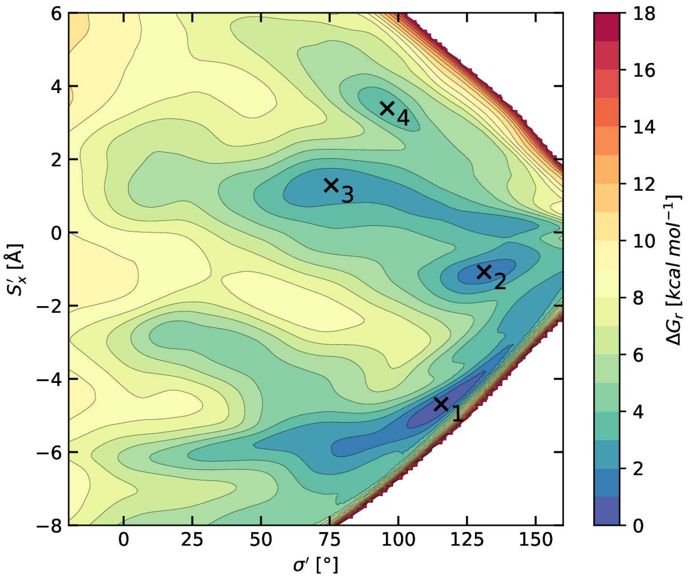

Figure SB13.1: Free energy surface for the aA/sG base pair. Labeled crosses show position of selected free energy minima (thermodynamic states). Free energy isolines are spaced by 1 kcal mol^{-1}.

Table SB13.1: Positions of selected free minima on the free energy surface for the aA/sG base pair. IDs correspond to the selected free energy minima shown in Figure SB13.1. Confidence interval of the free energy ΔG_r is provided at three standard deviations.

ID	σ' [°]	S'_x [Å]	ΔG_r [$kcal\ mol^{-1}$]
1	115.6	-4.69	0.00±0.00
2	131.2	-1.08	1.21±0.20
3	75.6	1.29	2.14±0.19
4	95.9	3.39	3.00±0.21

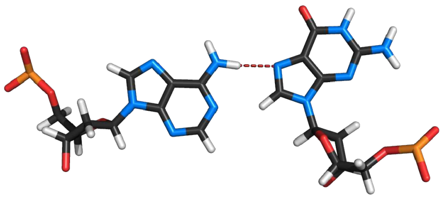

ID 1: $\sigma' = 115.6°$, S'_x = -4.69 Å

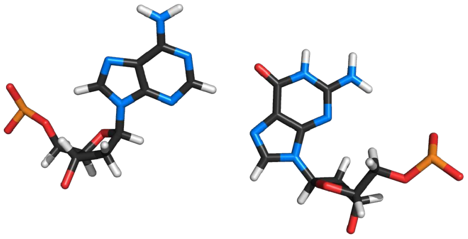

ID 2: $\sigma' = 131.2°$, S'_x = -1.08 Å

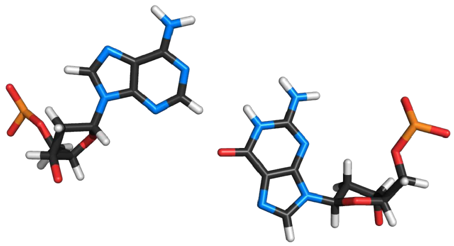

ID 3: $\sigma' = 75.6°$, S'_x = 1.29 Å

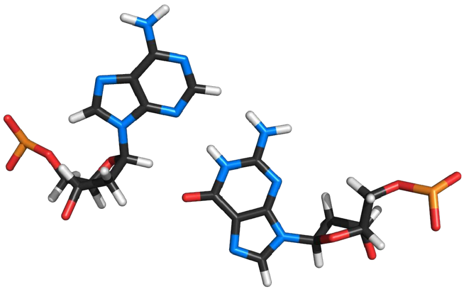

ID 4: $\sigma' = 95.9°$, S'_x = 3.39 Å

Figure SB13.2: Average geometries representing selected free energy minima for the aA/sG base pair. IDs correspond to the selected free energy minima shown in Figure SB13.1. Major and minor grooves are top and bottom, respectively. View direction is along the z-axis of DNA.

Table SB13.2: Number of analysed snapshots (N_{snap}), abundances (abu), average hydrogen bond distances and angles from hydrogen bond analysis provided by cpptraj for selected states of the aA/sG base pair. IDs correspond to the selected free energy minima shown in Figure SB13.1. Residue and atom numbering are provided in Figure SA1.

ID	N_{snap}	acceptor	H-donor	donor	abu [%]	d_{avg} [Å]	a_{avg} [°]
1	140	G20@N7	A7@H61	A7@N6	23.6	2.9	156.6
2	104	—	—	—	—	—	—
3	418	G20@OP2	G20@H22	G20@N2	16.3	2.8	166.1
4	167	A7@N3	G20@H1	G20@N1	7.2	3.0	158.9

Table SB13.3: *Simple* base-pair parameters for selected states of the aA/sG base pair. IDs corresponds to the free energy minima shown in Figure SB13.1. Residue numbering in base pairs A/B are provided in Figure SA1. Abundances (*abu*), average values ($\langle X \rangle$) and standard deviations of samples ($s(X)$) are calculated by 3DNA for Shear (S_x), Stretch (S_y), Stagger (S_z), Buckle (κ), Propeller (π), Opening (σ) employing the standard reference frames for the nucleobases. Only five central base pairs were included in the analysis for each state. Base pairs with a mismatch are highlighted in gray.

ID	A/B	abu [%]	$\langle S_x \rangle$	$s(S_x)$	$\langle S_y \rangle$	$s(S_y)$ [Å]	$\langle S_z \rangle$	$s(S_z)$	$\langle \kappa \rangle$	$s(\kappa)$	$\langle \pi \rangle$	$s(\pi)$ [°]	$\langle \sigma \rangle$	$s(\sigma)$
	5/22	100.0	-0.19	0.28	0.03	0.11	-0.03	0.35	5.9	9.5	-13.7	7.1	-0.1	5.0
	6/21	100.0	-0.03	0.27	0.01	0.12	-0.23	0.46	7.5	11.1	-12.2	8.0	0.3	5.7
1	7/20	100.0	-4.70	0.19	5.12	0.32	0.46	0.65	-0.0	10.5	-4.1	10.5	-64.7	3.5
	8/19	100.0	0.10	0.29	0.02	0.13	0.13	0.52	5.3	10.7	-7.3	10.2	2.5	5.5
	9/18	100.0	0.20	0.30	0.04	0.12	0.06	0.42	1.1	9.8	-17.7	7.0	0.5	5.4
	5/22	100.0	-0.20	0.27	0.03	0.11	0.02	0.39	3.1	8.2	-18.1	6.9	0.6	4.7
	6/21	100.0	-0.26	0.24	0.01	0.11	-0.20	0.38	-3.9	9.4	-15.4	8.1	2.0	4.8
2	7/20	97.1	-1.08	0.09	4.83	0.21	0.51	0.56	8.6	9.9	-16.7	8.5	-48.8	3.2
	8/19	100.0	0.23	0.30	0.00	0.12	-0.06	0.42	4.4	9.5	-14.3	8.0	2.2	5.3
	9/18	100.0	0.21	0.25	0.03	0.10	-0.05	0.34	-2.3	8.9	-16.7	7.2	0.0	4.3
	5/22	100.0	-0.18	0.27	0.03	0.12	-0.03	0.41	-5.8	10.3	-20.2	7.4	-0.8	5.1
	6/21	100.0	-0.16	0.35	0.08	0.17	-0.04	0.45	-16.0	11.1	-21.3	11.0	-1.3	6.9
	7/19	0.5	0.26	0.04	0.17	0.06	-0.89	0.38	1.4	0.5	-4.4	3.0	-7.3	3.9
3	7/20	70.1	1.28	0.13	3.72	0.29	-0.17	0.72	-1.5	12.1	-12.0	11.3	-102.9	4.8
	8/18	0.2	1.24	0.00	0.81	0.00	-1.00	0.00	16.4	0.0	-17.7	0.0	-19.2	0.0
	8/19	99.0	0.12	0.27	0.03	0.13	-0.04	0.47	-1.4	10.9	-9.3	12.4	1.3	6.0
	9/18	99.8	0.10	0.51	0.05	0.17	0.07	0.42	0.4	9.8	-14.7	7.6	0.0	5.1
	5/22	100.0	-0.20	0.27	0.02	0.10	0.03	0.40	-1.9	10.1	-18.2	6.5	0.4	5.1
	6/21	100.0	-0.02	0.53	0.01	0.20	-0.13	0.49	-11.9	11.1	-17.6	7.9	-1.0	6.2
4	7/20	97.0	3.39	0.16	4.23	0.23	0.05	0.74	10.2	11.7	-6.2	10.3	-83.8	2.6
	8/18	1.8	0.02	0.15	0.09	0.15	0.12	0.40	-27.9	9.7	10.8	6.4	-5.8	5.3
	8/19	95.8	0.05	0.24	0.00	0.13	0.07	0.51	5.4	10.8	-14.5	9.9	3.2	5.7
	9/18	98.2	0.07	0.93	0.06	0.25	0.03	0.42	2.1	10.2	-17.1	8.3	-1.4	6.5

Table SB13.4: *Simple* step parameters for selected states of the aA/sG base pair. IDs corresponds to the free energy minima shown in Figure SB13.1. Residue numbering in base pairs and steps A1-B1/A2-B2 are provided in Figure SA1. Abundances (*abu*), average values ($\langle X \rangle$) and standard deviations of samples ($s(X)$) are calculated by 3DNA for Shift (D_x), Slide (D_y), Rise (D_z), Tilt (τ), Roll (ρ), Twist (ω) employing the standard reference frames for the nucleobases. Only five central base pairs were included in the analysis for each state. Steps including a mismatch are highlighted in gray.

ID	A1-B1 A2-B2	abu [%]	$\langle D_x \rangle$	$s(D_x)$	$\langle D_y \rangle$ [Å]	$s(D_y)$	$\langle D_z \rangle$	$s(D_z)$	$\langle \tau \rangle$	$s(\tau)$	$\langle \rho \rangle$ [°]	$s(\rho)$	$\langle \omega \rangle$	$s(\omega)$
	5-6/21-22	100.0	0.27	0.59	-0.66	0.54	3.31	0.28	1.1	4.0	-1.8	5.3	33.0	4.4
1	6-7/20-21	100.0	2.93	1.05	-2.95	0.56	2.95	0.46	-1.6	6.0	10.1	8.5	43.2	8.0
	7-8/19-20	100.0	-1.14	0.50	2.78	0.64	3.41	0.34	0.9	5.3	4.7	5.0	21.4	4.7
	8-9/18-19	100.0	-0.76	0.57	-0.09	0.48	3.44	0.29	-2.2	4.0	0.1	5.6	38.7	3.6
	5-6/21-22	100.0	0.68	0.48	-0.05	0.38	3.37	0.27	4.4	3.6	1.7	5.3	39.8	3.4
	6-7/20-21	97.1	2.82	0.39	-0.39	0.41	3.17	0.25	1.9	4.2	1.7	4.8	20.9	3.6
2	6-8/19-21	2.9	-0.10	0.32	-2.47	0.02	6.58	0.20	-2.4	3.2	10.3	7.0	48.9	5.9
	7-8/19-20	97.1	-1.93	0.64	0.40	0.51	3.01	0.25	-0.7	4.8	13.9	5.5	34.5	5.6
	8-9/18-19	100.0	-0.83	0.50	-0.38	0.46	3.43	0.28	-2.8	4.5	0.9	5.0	37.6	3.9
	5-6/21-22	100.0	0.17	0.59	-0.46	0.51	3.55	0.34	1.3	4.2	6.0	6.1	37.0	4.0
	6-7/19-21	0.5	3.53	0.15	2.41	0.51	3.92	0.72	-2.9	1.3	4.3	0.1	55.7	6.3
	6-7/20-21	70.1	1.01	0.67	0.87	0.62	3.30	0.28	2.2	4.9	4.8	5.0	20.2	5.2
3	6-8/19-21	28.7	0.76	1.25	0.71	1.18	6.42	0.39	1.1	6.3	8.8	8.0	59.1	7.6
	7-8/18-19	0.2	-1.61	0.00	0.18	0.00	2.94	0.00	-6.5	0.0	3.3	0.0	33.4	0.0
	7-8/19-20	69.9	-0.48	1.10	0.53	0.87	3.09	0.33	-2.6	6.3	6.9	8.6	40.8	8.7
	7-9/18-19	0.2	-0.92	0.00	-1.51	0.00	5.93	0.00	13.8	0.0	3.3	0.0	36.7	0.0
	8-9/18-19	99.0	-0.73	0.73	-0.19	0.64	3.31	0.29	-1.9	4.4	-0.5	6.1	36.1	4.3
	5-6/21-22	100.0	0.29	0.60	-0.34	0.47	3.51	0.31	3.7	4.4	2.9	5.3	36.3	3.8
	6-7/20-21	97.0	1.22	0.51	2.23	0.48	3.42	0.32	0.4	4.3	2.0	3.8	16.8	3.9
	6-8/19-21	2.4	0.29	1.35	-1.49	0.75	6.40	0.45	-3.1	6.0	14.6	7.8	61.4	4.1
4	7-8/18-20	1.8	2.21	0.71	0.96	0.78	5.73	0.55	-20.1	6.0	28.2	1.8	73.7	3.5
	7-8/19-20	93.4	-0.74	0.72	-1.85	0.50	2.89	0.38	-0.8	6.1	17.0	8.2	45.6	6.5
	7-9/18-20	0.6	-1.34	0.00	0.07	0.00	7.16	0.00	12.8	0.0	6.0	0.0	93.7	0.0
	8-9/18-19	95.8	-0.79	0.66	-0.30	0.58	3.41	0.25	-1.6	4.6	1.6	5.3	33.2	5.1

3.4 aA/sT

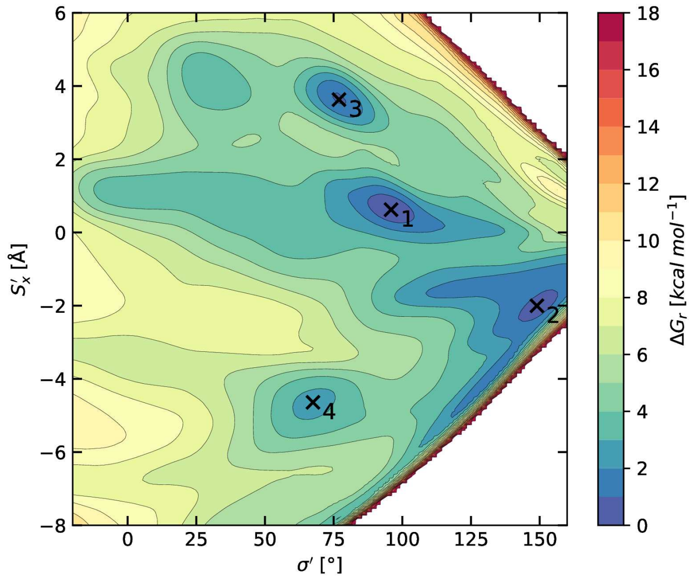

Figure SB14.1: Free energy surface for the aA/sT base pair. Labeled crosses show position of selected free energy minima (thermodynamic states). Free energy isolines are spaced by 1 kcal mol^{-1}.

Table SB14.1: Positions of selected free minima on the free energy surface for the aA/sT base pair. IDs correspond to the selected free energy minima shown in Figure SB14.1. Confidence interval of the free energy ΔG_r is provided at three standard deviations.

ID	σ' [°]	S'_x [Å]	ΔG_r [$kcal\ mol^{-1}$]
1	95.9	0.62	0.00±0.00
2	149.0	-2.00	0.50±0.23
3	76.9	3.63	0.88±0.12
4	67.5	-4.64	2.61±0.14

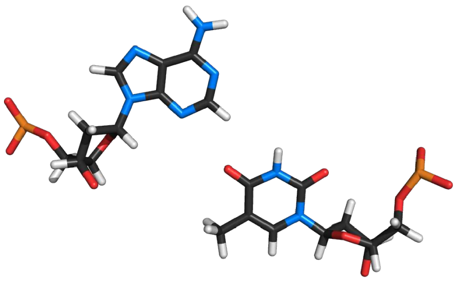

ID 1: $\sigma' = 95.9°$, $S'_x = 0.62$ Å

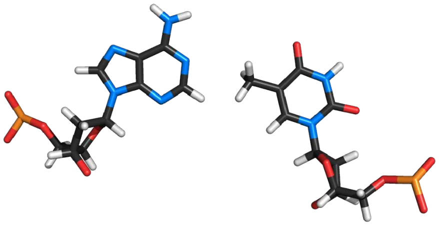

ID 2: $\sigma' = 149.0°$, $S'_x = -2.00$ Å

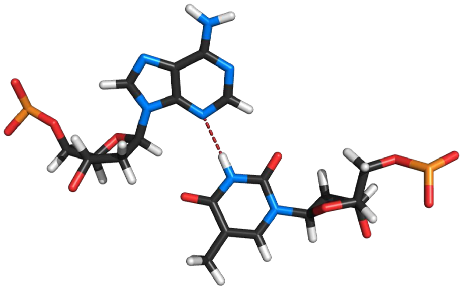

ID 3: $\sigma' = 76.9°$, $S'_x = 3.63$ Å

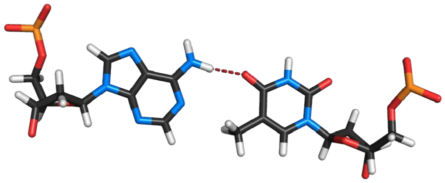

ID 4: $\sigma' = 67.5°$, $S'_x = -4.64$ Å

Figure SB14.2: Average geometries representing selected free energy minima for the aA/sT base pair. IDs correspond to the selected free energy minima shown in Figure SB14.1. Major and minor grooves are top and bottom, respectively. View direction is along the z-axis of DNA.

Table SB14.2: Number of analysed snapshots (N_{snap}), abundances (abu), average hydrogen bond distances and angles from hydrogen bond analysis provided by cpptraj for selected states of the aA/sT base pair. IDs correspond to the selected free energy minima shown in Figure SB14.1. Residue and atom numbering are provided in Figure SA1.

ID	N_{snap}	acceptor	H-donor	donor	abu [%]	d_{avg} [Å]	a_{avg} [°]
1	306	—	—	—	—	—	—
2	77	—	—	—	—	—	—
3	133	A7@N3	T20@H3	T20@N3	28.6	2.9	163.4
4	289	T20@O4	A7@H61	A7@N6	51.6	2.9	157.1

Table SB14.3: *Simple* base-pair parameters for selected states of the aA/sT base pair. IDs corresponds to the free energy minima shown in Figure SB14.1. Residue numbering in base pairs A/B are provided in Figure SA1. Abundances (*abu*), average values ($\langle X \rangle$) and standard deviations of samples ($s(X)$) are calculated by 3DNA for Shear (S_x), Stretch (S_y), Stagger (S_z), Buckle (κ), Propeller (π), Opening (σ) employing the standard reference frames for the nucleobases. Only five central base pairs were included in the analysis for each state. Base pairs with a mismatch are highlighted in gray.

ID	A/B	abu [%]	$\langle S_x \rangle$	$s(S_x)$	$\langle S_y \rangle$ [Å]	$s(S_y)$	$\langle S_z \rangle$	$s(S_z)$	$\langle \kappa \rangle$	$s(\kappa)$	$\langle \pi \rangle$ [°]	$s(\pi)$	$\langle \sigma \rangle$	$s(\sigma)$
1	5/22	100.0	-0.20	0.24	0.02	0.12	-0.03	0.38	-5.5	8.4	-18.8	7.1	-0.9	4.6
	6/21	100.0	-0.19	0.27	0.04	0.13	-0.22	0.39	-13.3	9.2	-6.1	8.3	-2.4	4.8
	7/19	80.7	0.45	0.25	0.08	0.12	-0.01	0.54	19.4	8.8	-5.1	9.9	0.7	3.3
	7/20	16.7	0.61	0.10	3.80	0.29	-0.03	0.76	-1.9	12.6	-1.0	10.4	-84.2	2.5
	8/19	18.6	-0.12	0.88	0.02	0.26	-0.03	0.48	1.1	11.3	-11.6	7.4	-1.7	6.3
	9/18	100.0	0.31	0.31	0.07	0.15	0.26	0.45	10.7	10.5	-19.9	7.5	-3.8	5.7
2	5/22	100.0	-0.21	0.27	0.01	0.12	-0.09	0.40	5.3	10.0	-10.9	8.0	0.3	4.9
	6/21	100.0	-0.05	0.27	0.06	0.15	-0.44	0.41	12.4	14.4	-10.2	10.9	3.0	6.9
	8/19	100.0	-0.04	0.34	-0.03	0.13	-0.63	0.43	-5.3	14.1	-14.6	12.8	1.8	6.0
	9/18	100.0	0.20	0.28	0.03	0.12	-0.14	0.38	-5.3	10.1	-8.5	8.5	-0.3	4.4
3	5/22	100.0	-0.17	0.26	0.02	0.12	0.03	0.44	-2.2	10.2	-17.4	7.2	-1.3	5.0
	6/21	100.0	-0.01	0.57	-0.03	0.22	-0.30	0.39	-8.2	10.1	-14.9	7.3	-0.4	4.9
	7/19	31.6	0.28	0.36	-0.01	0.17	-0.68	0.58	5.9	9.1	-25.9	11.9	1.9	3.5
	7/20	66.9	3.64	0.13	1.79	0.16	0.46	0.67	18.8	11.2	-6.0	8.9	-102.6	2.0
	8/18	0.8	-0.07	0.00	-0.15	0.00	-1.24	0.00	-5.0	0.0	-16.0	0.0	-0.1	0.0
	8/19	66.9	-0.72	1.57	0.20	0.78	-0.35	0.76	3.9	12.9	-17.6	9.2	14.3	11.0
	8/20	0.8	2.61	0.00	0.71	0.00	2.45	0.00	2.9	0.0	11.0	0.0	-95.8	0.0
	9/18	99.2	0.15	0.29	0.04	0.11	-0.02	0.53	-0.5	13.5	-12.9	9.7	-0.3	5.9
4	5/22	100.0	-0.19	0.25	0.03	0.12	0.04	0.35	-2.8	9.5	-19.4	6.8	-0.1	4.9
	6/21	100.0	-0.25	0.31	0.08	0.21	0.13	0.45	-10.6	10.4	-14.8	8.0	-2.9	7.2
	7/20	99.7	-4.65	0.19	2.98	0.23	-0.66	0.53	-1.1	10.8	-7.7	9.1	-112.5	3.4
	8/19	99.3	0.28	0.31	0.09	0.15	0.12	0.44	10.0	9.6	-14.0	8.7	-1.0	6.4
	9/18	100.0	0.18	0.29	0.02	0.13	0.12	0.39	3.1	9.1	-20.0	6.8	-1.3	5.0

Table SB14.4: *Simple* step parameters for selected states of the aA/sT base pair. IDs corresponds to the free energy minima shown in Figure SB14.1. Residue numbering in base pairs and steps A1-B1/A2-B2 are provided in Figure SA1. Abundances (*abu*), average values ($\langle X \rangle$) and standard deviations of samples ($s(X)$) are calculated by 3DNA for Shift (D_x), Slide (D_y), Rise (D_z), Tilt (τ), Roll (ρ), Twist (ω) employing the standard reference frames for the nucleobases. Only five central base pairs were included in the analysis for each state. Steps including a mismatch are highlighted in gray.

ID	A1-B1 A2-B2	abu [%]	$\langle D_x \rangle$	$s(D_x)$	$\langle D_y \rangle$ [Å]	$s(D_y)$	$\langle D_z \rangle$	$s(D_z)$	$\langle \tau \rangle$	$s(\tau)$	$\langle \rho \rangle$ [°]	$s(\rho)$	$\langle \omega \rangle$	$s(\omega)$
	5-6/21-22	100.0	0.71	0.47	-0.13	0.39	3.50	0.28	3.9	3.5	0.1	5.3	39.9	2.9
	6-7/19-21	80.7	3.54	0.44	0.48	0.46	3.08	0.42	-10.4	5.2	2.6	6.3	46.1	3.0
	6-7/20-21	16.7	2.72	0.53	0.43	0.48	3.31	0.28	0.7	4.3	2.8	5.1	13.9	4.6
1	6-8/19-21	2.3	0.67	0.47	-2.15	0.71	6.10	0.37	-1.5	4.8	11.6	7.7	56.8	5.9
	7-8/19-20	16.3	-2.47	0.59	-1.03	0.48	2.81	0.38	-1.8	5.5	12.4	9.3	46.4	7.5
	7-9/18-19	80.4	-4.12	0.43	0.91	0.52	5.08	0.50	11.5	4.8	12.7	5.7	56.2	2.6
	8-9/18-19	18.6	-0.19	0.50	-0.87	0.50	3.39	0.35	0.2	4.4	-0.3	5.0	32.6	4.3
	5-6/21-22	100.0	0.42	0.53	-0.90	0.45	3.30	0.33	0.6	4.6	-0.7	7.3	32.7	3.2
2	6-8/19-21	83.1	0.16	0.83	-3.14	1.13	5.90	0.61	3.9	9.1	-5.6	10.1	68.7	6.5
	8-9/18-19	100.0	-0.37	0.41	-0.83	0.54	3.43	0.36	-2.6	4.2	2.5	7.7	32.5	4.6
	5-6/21-22	100.0	0.21	0.56	-0.27	0.54	3.44	0.29	4.2	4.1	3.1	5.3	34.3	4.3
	6-7/19-21	31.6	3.41	0.49	0.12	0.52	4.06	0.45	-12.7	5.1	6.0	6.8	56.3	4.5
	6-7/20-21	66.9	0.87	0.55	1.30	0.50	3.27	0.28	-5.0	4.5	1.7	5.2	20.7	4.9
	6-8/19-21	1.5	0.44	0.37	-1.47	1.93	5.19	0.13	0.4	0.0	12.2	0.3	67.8	5.8
	7-8/18-19	0.8	-0.88	0.00	-0.93	0.00	4.61	0.00	10.4	0.0	-1.7	0.0	33.7	0.0
3	7-8/19-20	65.4	0.01	0.74	-1.33	0.95	3.48	0.59	5.0	7.3	12.2	7.3	49.5	6.6
	7-8/20-19	0.8	-2.02	0.00	0.76	0.00	0.91	0.00	16.8	0.0	-6.8	0.0	-21.4	0.0
	7-9/18-19	30.1	-2.78	0.47	0.04	0.71	5.03	0.66	11.2	6.6	8.8	8.6	49.0	4.4
	8-9/18-19	66.9	-1.03	0.77	-0.37	0.59	3.55	0.37	-4.2	4.9	7.8	6.6	27.1	5.9
	8-9/18-20	0.8	0.12	0.00	-1.96	0.00	4.58	0.00	-1.6	0.0	15.7	0.0	73.8	0.0
	5-6/21-22	99.7	0.45	0.52	-0.35	0.52	3.43	0.30	1.9	4.1	0.1	4.8	39.8	4.0
	5-7/20-22	0.3	-2.72	0.00	-0.04	0.00	4.14	0.00	-22.8	0.0	47.3	0.0	54.4	0.0
	6-7/20-21	99.3	0.22	0.59	-0.78	0.57	2.84	0.34	5.8	5.4	5.4	6.6	27.8	6.0
4	7-8/19-20	99.3	0.25	0.79	1.77	0.66	3.26	0.31	-6.3	4.2	7.3	6.1	25.8	5.6
	7-9/18-20	0.3	0.07	0.00	0.36	0.00	6.17	0.00	-12.2	0.0	5.8	0.0	56.1	0.0
	8-9/18-19	99.3	-0.60	0.47	-0.19	0.45	3.40	0.29	-3.0	4.0	-0.2	6.0	40.4	3.6

3.5 aG/sC

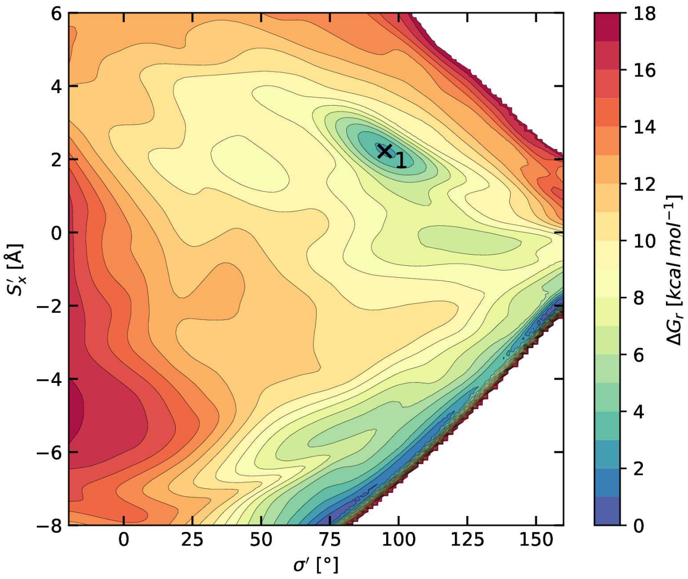

Figure SB15.1: Free energy surface for the aG/sC base pair. Labeled crosses show position of selected free energy minima (thermodynamic states). Free energy isolines are spaced by 1 kcal mol^{-1}.

Table SB15.1: Positions of selected free minima on the free energy surface for the aG/sC base pair. IDs correspond to the selected free energy minima shown in Figure SB15.1. Confidence interval of the free energy ΔG_r is provided at three standard deviations.

ID	σ' [°]	S'_x [Å]	ΔG_r [$kcal\ mol^{-1}$]
1	95.0	2.22	2.78±0.30

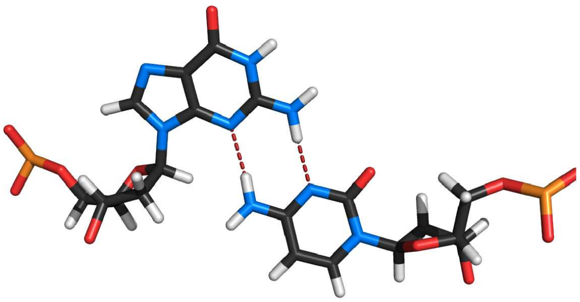

ID 1: $\sigma' = 95.0°$, $S'_x = 2.22$ Å

Figure SB15.2: Average geometries representing selected free energy minima for the aG/sC base pair. IDs correspond to the selected free energy minima shown in Figure SB15.1. Major and minor grooves are top and bottom, respectively. View direction is along the z-axis of DNA.

Table SB15.2: Number of analysed snapshots (N_{snap}), abundances (abu), average hydrogen bond distances and angles from hydrogen bond analysis provided by cpptraj for selected states of the aG/sC base pair. IDs correspond to the selected free energy minima shown in Figure SB15.1. Residue and atom numbering are provided in Figure SA1.

ID	N_{snap}	acceptor	H-donor	donor	abu [%]	d_{avg} [Å]	a_{avg} [°]
1	122	C20@N3	G7@H22	G7@N2	68.0	2.9	162.7
		G7@N3	C20@H41	C20@N4	45.1	2.9	164.0

Table SB15.3: *Simple* base-pair parameters for selected states of the aG/sC base pair. IDs corresponds to the free energy minima shown in Figure SB15.1. Residue numbering in base pairs A/B are provided in Figure SA1. Abundances (*abu*), average values ($\langle X \rangle$) and standard deviations of samples ($s(X)$) are calculated by 3DNA for Shear (S_x), Stretch (S_y), Stagger (S_z), Buckle (κ), Propeller (π), Opening (σ) employing the standard reference frames for the nucleobases. Only five central base pairs were included in the analysis for each state. Base pairs with a mismatch are highlighted in gray.

ID	A/B	abu [%]	$\langle S_x \rangle$	$s(S_x)$	$\langle S_y \rangle$ [Å]	$s(S_y)$	$\langle S_z \rangle$	$s(S_z)$	$\langle \kappa \rangle$	$s(\kappa)$	$\langle \pi \rangle$ [°]	$s(\pi)$	$\langle \sigma \rangle$	$s(\sigma)$
	5/22	100.0	-0.18	0.25	0.05	0.14	0.03	0.34	-1.9	9.3	-18.3	7.4	-1.0	5.3
	6/21	100.0	-0.00	0.28	-0.01	0.13	-0.20	0.38	-10.7	8.7	-12.0	9.0	0.3	4.6
1	7/20	100.0	2.24	0.12	2.71	0.14	0.16	0.77	4.3	10.0	-6.8	13.0	-85.6	2.3
	8/19	98.4	-0.04	0.28	-0.05	0.22	-0.54	0.65	-2.2	12.7	-10.0	9.6	7.8	6.5
	9/18	100.0	0.20	0.28	0.03	0.12	-0.19	0.43	-1.6	10.2	-8.1	10.3	0.9	4.6

Table SB15.4: *Simple* step parameters for selected states of the aG/sC base pair. IDs corresponds to the free energy minima shown in Figure SB15.1. Residue numbering in base pairs and steps A1-B1/A2-B2 are provided in Figure SA1. Abundances (abu), average values ($\langle X \rangle$) and standard deviations of samples ($s(X)$) are calculated by 3DNA for Shift (D_x), Slide (D_y), Rise (D_z), Tilt (τ), Roll (ρ), Twist (ω) employing the standard reference frames for the nucleobases. Only five central base pairs were included in the analysis for each state. Steps including a mismatch are highlighted in gray.

ID	A1-B1<br>A2-B2	abu<br>[%]	$\langle D_x \rangle$	$s(D_x)$	$\langle D_y \rangle$	$s(D_y)$<br>[Å]	$\langle D_z \rangle$	$s(D_z)$	$\langle \tau \rangle$	$s(\tau)$	$\langle \rho \rangle$	$s(\rho)$<br>[°]	$\langle \omega \rangle$	$s(\omega)$
	5-6/21-22	100.0	0.63	0.54	-0.21	0.48	3.50	0.29	4.8	3.5	1.0	5.3	37.0	3.6
1	6-7/20-21	100.0	1.96	0.46	1.06	0.50	3.32	0.26	-1.7	3.8	1.1	4.2	16.5	4.4
	7-8/19-20	98.4	-0.68	0.94	-1.48	1.05	3.51	0.55	3.2	6.0	11.8	9.4	43.0	6.8
	8-9/18-19	98.4	-0.88	0.64	-0.55	0.57	3.39	0.35	-3.2	4.3	1.5	6.6	30.7	6.2

3.6 aG/sG

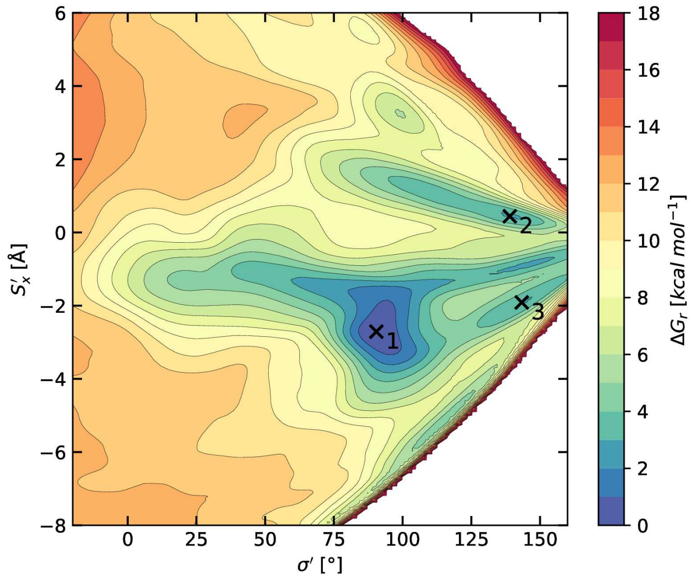

Figure SB16.1: Free energy surface for the aG/sG base pair. Labeled crosses show position of selected free energy minima (thermodynamic states). Free energy isolines are spaced by 1 kcal mol^{-1}.

Table SB16.1: Positions of selected free minima on the free energy surface for the aG/sG base pair. IDs correspond to the selected free energy minima shown in Figure SB16.1. Confidence interval of the free energy ΔG_r is provided at three standard deviations.

ID	σ' [°]	S'_x [Å]	ΔG_r [$kcal\ mol^{-1}$]
1	90.5	-2.71	0.00±0.00
2	139.0	0.44	2.88±0.14
3	143.3	-1.91	2.94±0.16

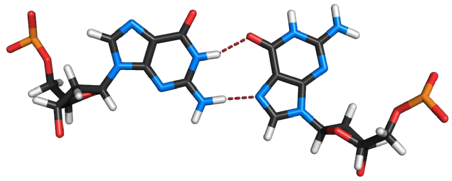

ID 1: $\sigma' = 90.5°$, S'_x = -2.71 Å

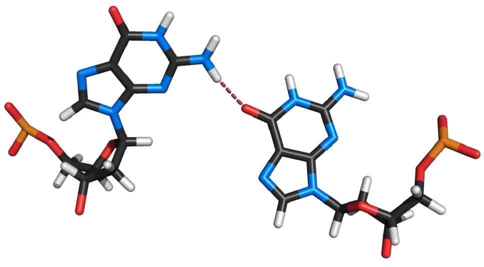

ID 2: $\sigma' = 139.0°$, S'_x = 0.44 Å

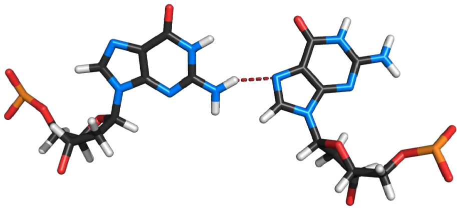

ID 3: $\sigma' = 143.3°$, S'_x = -1.91 Å

Figure SB16.2: Average geometries representing selected free energy minima for the aG/sG base pair. IDs correspond to the selected free energy minima shown in Figure SB16.1. Major and minor grooves are top and bottom, respectively. View direction is along the z-axis of DNA.

Table SB16.2: Number of analysed snapshots (N_{snap}), abundances (abu), average hydrogen bond distances and angles from hydrogen bond analysis provided by cpptraj for selected states of the aG/sG base pair. IDs correspond to the selected free energy minima shown in Figure SB16.1. Residue and atom numbering are provided in Figure SA1.

ID	N_{snap}	acceptor	H-donor	donor	abu [%]	d_{avg} [Å]	a_{avg} [°]
1	208	G20@N7	G7@H21	G7@N2	72.6	2.9	163.7
		G20@O6	G7@H1	G7@N1	54.8	2.9	154.3
		G20@OP2	G20@H22	G20@N2	1.4	2.8	168.5
2	396	G20@O6	G7@H22	G7@N2	79.5	2.8	159.0
		G20@OP2	G20@H22	G20@N2	20.4	2.9	166.2
3	183	G20@N7	G7@H21	G7@N2	26.2	2.9	149.2

Table SB16.3: *Simple* base-pair parameters for selected states of the aG/sG base pair. IDs corresponds to the free energy minima shown in Figure SB16.1. Residue numbering in base pairs A/B are provided in Figure SA1. Abundances (*abu*), average values ($\langle X \rangle$) and standard deviations of samples ($s(X)$) are calculated by 3DNA for Shear (S_x), Stretch (S_y), Stagger (S_z), Buckle (κ), Propeller (π), Opening (σ) employing the standard reference frames for the nucleobases. Only five central base pairs were included in the analysis for each state. Base pairs with a mismatch are highlighted in gray.

ID	A/B	abu [%]	$\langle S_x \rangle$	$s(S_x)$	$\langle S_y \rangle$ [Å]	$s(S_y)$	$\langle S_z \rangle$	$s(S_z)$	$\langle \kappa \rangle$	$s(\kappa)$	$\langle \pi \rangle$ [°]	$s(\pi)$	$\langle \sigma \rangle$	$s(\sigma)$
	5/22	100.0	-0.18	0.26	0.04	0.13	-0.01	0.39	2.8	9.4	-18.1	7.1	0.0	5.0
	6/21	100.0	-0.14	0.28	0.03	0.12	0.02	0.43	-1.3	10.6	-15.6	8.6	0.0	5.3
1	7/20	100.0	-2.72	0.12	2.73	0.14	0.10	0.36	3.2	8.4	-8.8	8.6	-89.5	1.9
	8/19	99.5	0.22	0.29	0.04	0.13	0.16	0.40	7.7	7.9	-17.9	7.5	-0.8	6.1
	9/18	100.0	0.23	0.24	0.06	0.12	0.03	0.34	0.6	9.0	-19.5	6.2	-1.7	4.8
	5/22	100.0	-0.22	0.26	0.04	0.12	-0.02	0.38	6.4	7.8	-17.2	6.6	0.7	4.8
	6/21	100.0	-0.00	0.30	-0.01	0.15	-0.32	0.41	-1.3	10.1	-25.0	8.1	2.7	6.6
2	7/20	100.0	0.44	0.07	6.29	0.21	0.50	0.51	13.4	8.8	-26.2	11.0	-40.6	3.2
	8/19	99.8	0.11	0.29	0.03	0.13	0.22	0.45	3.1	9.5	-12.6	9.0	5.5	5.1
	9/18	100.0	0.21	0.26	0.06	0.12	0.02	0.38	0.8	9.0	-18.1	6.8	-0.8	4.8
	5/22	100.0	-0.22	0.25	0.04	0.12	0.03	0.40	-2.4	9.3	-17.6	6.8	-0.1	4.9
	6/21	99.5	-0.20	0.28	0.05	0.13	-0.10	0.41	-4.8	9.3	-7.9	8.7	2.2	6.0
3	7/20	100.0	-1.91	0.20	6.41	0.37	0.84	0.58	-3.6	12.5	-22.9	10.4	-36.7	4.5
	8/19	100.0	0.15	0.29	0.07	0.19	-0.22	0.47	-0.7	10.7	-12.4	7.6	2.4	7.6
	9/18	100.0	0.18	0.28	0.04	0.11	-0.02	0.36	-0.2	9.2	-16.2	6.8	-0.0	4.8

Table SB16.4: *Simple* step parameters for selected states of the aG/sG base pair. IDs corresponds to the free energy minima shown in Figure SB16.1. Residue numbering in base pairs and steps A1-B1/A2-B2 are provided in Figure SA1. Abundances (abu), average values ($\langle X \rangle$) and standard deviations of samples ($s(X)$) are calculated by 3DNA for Shift (D_x), Slide (D_y), Rise (D_z), Tilt (τ), Roll (ρ), Twist (ω) employing the standard reference frames for the nucleobases. Only five central base pairs were included in the analysis for each state. Steps including a mismatch are highlighted in gray.

ID	A1-B1 A2-B2	abu [%]	$\langle D_x \rangle$	$s(D_x)$	$\langle D_y \rangle$	$s(D_y)$ [Å]	$\langle D_z \rangle$	$s(D_z)$	$\langle \tau \rangle$	$s(\tau)$	$\langle \rho \rangle$ [°]	$s(\rho)$	$\langle \omega \rangle$	$s(\omega)$
	5-6/21-22	100.0	0.57	0.59	-0.31	0.49	3.35	0.25	2.2	3.9	1.5	5.2	37.5	4.4
	6-7/20-21	100.0	1.27	0.63	-0.86	0.54	3.08	0.27	0.7	4.2	8.1	5.7	26.2	5.5
1	7-8/19-20	99.5	-0.93	0.52	0.90	0.61	3.16	0.26	-2.7	4.1	6.4	4.8	33.2	4.8
	7-9/18-20	0.5	0.66	0.00	0.08	0.00	6.53	0.00	-0.7	0.0	20.7	0.0	47.6	0.0
	8-9/18-19	99.5	-0.71	0.61	-0.34	0.43	3.38	0.24	-2.1	3.8	-0.9	5.4	37.9	4.0
	5-6/21-22	100.0	0.32	0.56	-0.32	0.43	3.40	0.27	4.3	4.1	4.9	5.2	36.3	4.1
	6-7/20-21	100.0	2.57	0.54	1.76	0.56	3.49	0.29	6.9	4.7	3.5	4.6	21.7	4.2
2	7-8/19-20	99.8	-1.36	0.57	-0.36	0.64	2.81	0.28	-2.5	5.0	9.4	6.5	43.3	4.9
	7-9/18-20	0.2	-0.46	0.00	-2.85	0.00	5.86	0.00	1.1	0.0	11.3	0.0	60.4	0.0
	8-9/18-19	99.8	-1.24	0.70	0.02	0.55	3.34	0.25	-1.7	4.0	0.0	5.2	35.5	4.2
	5-6/21-22	99.5	0.78	0.56	-0.15	0.44	3.37	0.31	3.3	3.8	-0.3	5.1	38.2	3.8
	5-7/20-22	0.6	4.82	0.00	-1.92	0.00	5.70	0.00	9.8	0.0	18.0	0.0	65.1	0.0
3	6-7/20-21	99.5	3.15	1.16	-1.65	0.52	2.74	0.33	3.8	4.9	12.5	9.1	24.9	8.7
	7-8/19-20	100.0	-2.53	0.68	1.80	0.55	3.38	0.35	-2.6	6.3	8.9	5.3	26.8	7.8
	8-9/18-19	100.0	-0.53	0.64	-0.35	0.55	3.28	0.30	-3.1	4.0	0.8	5.9	36.8	5.0

3.7 aG/sT

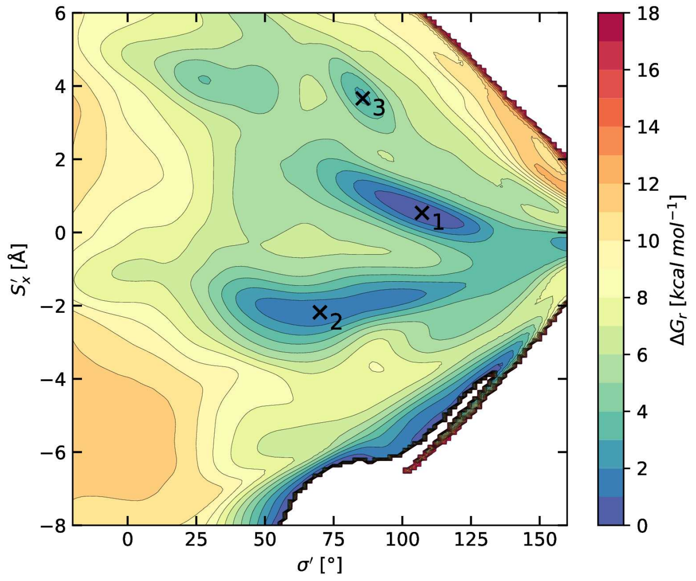

Figure SB17.1: Free energy surface for the aG/sT base pair. Labeled crosses show position of selected free energy minima (thermodynamic states). Free energy isolines are spaced by 1 kcal mol^{-1}.

Table SB17.1: Positions of selected free minima on the free energy surface for the aG/sT base pair. IDs correspond to the selected free energy minima shown in Figure SB17.1. Confidence interval of the free energy ΔG_r is provided at three standard deviations.

ID	σ' [°]	S'_x [Å]	ΔG_r [$kcal\ mol^{-1}$]
1	107.2	0.53	0.00±0.00
2	70.0	-2.18	1.08±0.16
3	85.6	3.66	2.77±0.19

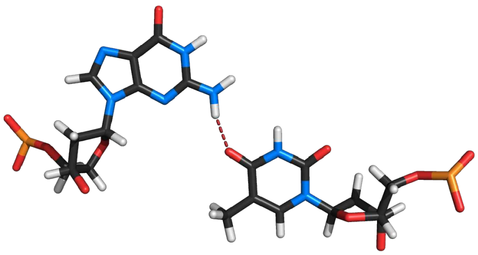

ID 1: $\sigma' = 107.2°$, $S'_x = 0.53$ Å

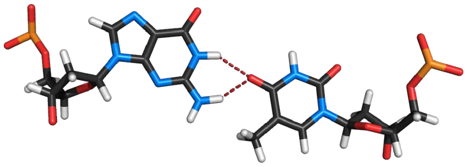

ID 2: $\sigma' = 70.0°$, $S'_x =$ -2.18 Å

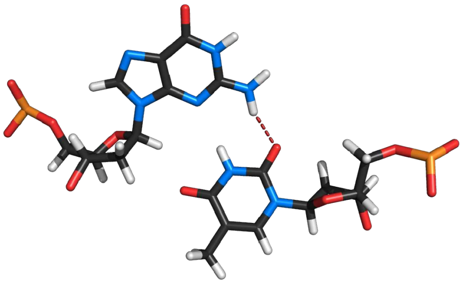

ID 3: $\sigma' = 85.6°$, $S'_x = 3.66$ Å

Figure SB17.2: Average geometries representing selected free energy minima for the aG/sT base pair. IDs correspond to the selected free energy minima shown in Figure SB17.1. Major and minor grooves are top and bottom, respectively. View direction is along the z-axis of DNA.

Table SB17.2: Number of analysed snapshots (N_{snap}), abundances (abu), average hydrogen bond distances and angles from hydrogen bond analysis provided by cpptraj for selected states of the aG/sT base pair. IDs correspond to the selected free energy minima shown in Figure SB17.1. Residue and atom numbering are provided in Figure SA1.

ID	N_{snap}	acceptor	H-donor	donor	abu [%]	d_{avg} [Å]	a_{avg} [°]
1	231	T20@O4	G7@H22	G7@N2	81.4	2.8	159.0
2	227	T20@O4	G7@H21	G7@N2	78.0	2.9	153.0
		T20@O4	G7@H1	G7@N1	47.6	2.9	146.3
3	59	T20@O2	G7@H22	G7@N2	69.5	2.8	152.1

Table SB17.3: *Simple* base-pair parameters for selected states of the aG/sT base pair. IDs corresponds to the free energy minima shown in Figure SB17.1. Residue numbering in base pairs A/B are provided in Figure SA1. Abundances (*abu*), average values ($\langle X \rangle$) and standard deviations of samples ($s(X)$) are calculated by 3DNA for Shear (S_x), Stretch (S_y), Stagger (S_z), Buckle (κ), Propeller (π), Opening (σ) employing the standard reference frames for the nucleobases. Only five central base pairs were included in the analysis for each state. Base pairs with a mismatch are highlighted in gray.

ID	A/B	abu [%]	$\langle S_x \rangle$	$s(S_x)$	$\langle S_y \rangle$ [Å]	$s(S_y)$	$\langle S_z \rangle$	$s(S_z)$	$\langle \kappa \rangle$	$s(\kappa)$	$\langle \pi \rangle$ [°]	$s(\pi)$	$\langle \sigma \rangle$	$s(\sigma)$
	5/22	100.0	-0.20	0.27	0.02	0.12	0.01	0.41	-6.5	8.5	-17.5	7.7	-0.9	4.7
	6/21	100.0	-0.08	0.28	0.04	0.14	-0.06	0.41	-10.8	9.1	-4.7	9.3	-1.2	4.6
1	7/20	99.6	0.53	0.14	4.68	0.32	-0.18	0.65	-1.4	9.9	1.1	10.2	-72.7	4.2
	8/19	99.6	-0.06	0.69	0.08	0.26	0.05	0.49	-1.8	10.8	-12.1	9.0	1.2	6.3
	9/18	100.0	0.17	0.27	0.03	0.14	-0.02	0.42	-2.1	10.3	-14.3	7.9	-0.3	5.2
	5/22	100.0	-0.16	0.27	0.02	0.13	-0.02	0.37	-3.2	8.8	-17.3	7.8	-0.7	5.3
	6/21	100.0	-0.10	0.30	0.06	0.19	-0.20	0.42	-8.4	9.6	-14.7	9.4	-2.6	6.4
2	7/20	100.0	-2.19	0.10	2.73	0.32	-0.39	0.49	-2.2	9.6	-5.8	9.7	-110.5	4.8
	8/19	100.0	0.25	0.30	0.07	0.15	0.18	0.46	8.9	10.7	-12.6	8.4	-0.9	5.9
	9/18	100.0	0.13	0.27	0.01	0.12	0.09	0.44	3.1	8.4	-18.0	8.0	-0.9	4.8
	5/22	100.0	-0.17	0.27	0.03	0.14	-0.00	0.43	-3.4	9.6	-17.8	5.2	0.1	6.2
	6/21	100.0	-0.04	0.31	-0.02	0.13	-0.23	0.36	-11.5	8.6	-15.6	6.9	1.5	5.5
3	7/20	100.0	3.65	0.14	2.39	0.13	0.66	0.50	17.1	7.8	-8.2	7.7	-94.2	1.8
	8/19	98.3	-0.02	0.30	0.09	0.26	-0.08	0.51	2.4	13.0	-22.5	11.1	11.9	10.2
	9/18	100.0	0.16	0.30	0.05	0.13	-0.08	0.48	0.3	10.2	-15.9	9.4	0.1	5.3

Table SB17.4: *Simple* step parameters for selected states of the aG/sT base pair. IDs corresponds to the free energy minima shown in Figure SB17.1. Residue numbering in base pairs and steps A1-B1/A2-B2 are provided in Figure SA1. Abundances (*abu*), average values ($\langle X \rangle$) and standard deviations of samples ($s(X)$) are calculated by 3DNA for Shift (D_x), Slide (D_y), Rise (D_z), Tilt (τ), Roll (ρ), Twist (ω) employing the standard reference frames for the nucleobases. Only five central base pairs were included in the analysis for each state. Steps including a mismatch are highlighted in gray.

ID	A1-B1 A2-B2	abu [%]	$\langle D_x \rangle$	$s(D_x)$	$\langle D_y \rangle$ [Å]	$s(D_y)$	$\langle D_z \rangle$	$s(D_z)$	$\langle \tau \rangle$	$s(\tau)$	$\langle \rho \rangle$ [°]	$s(\rho)$	$\langle \omega \rangle$	$s(\omega)$
1	5-6/21-22	100.0	0.74	0.55	-0.21	0.48	3.47	0.27	3.4	3.9	0.5	5.7	38.3	3.1
	6-7/20-21	99.6	2.82	0.49	0.66	0.51	3.22	0.27	1.4	4.2	4.0	4.3	13.0	5.4
	7-8/19-20	99.1	-2.62	0.84	-1.05	1.08	2.96	0.49	-2.0	5.9	10.3	8.5	45.8	6.0
	8-9/18-19	99.6	-0.40	0.54	-0.87	0.58	3.41	0.34	0.1	4.5	-1.1	5.5	31.9	5.0
2	5-6/21-22	100.0	0.19	0.59	-0.46	0.51	3.41	0.29	2.9	4.0	2.2	5.8	38.3	3.8
	6-7/20-21	100.0	1.19	0.62	-0.48	0.54	3.12	0.29	4.2	4.2	9.1	5.6	23.3	5.8
	7-8/19-20	100.0	-0.74	0.77	1.07	0.72	3.10	0.32	-6.2	4.5	5.8	7.5	29.0	6.1
	8-9/18-19	100.0	-0.45	0.55	-0.37	0.57	3.42	0.32	-2.1	4.5	0.0	6.3	40.5	4.2
3	5-6/21-22	100.0	0.38	0.72	-0.30	0.55	3.45	0.27	4.2	3.6	3.6	4.3	34.0	4.2
	6-7/20-21	100.0	1.23	0.44	1.61	0.46	3.20	0.22	-4.1	4.2	2.6	4.5	20.0	4.2
	7-8/19-20	98.3	-0.33	0.61	-1.17	0.64	3.27	0.44	7.0	5.5	12.9	7.0	51.1	4.0
	8-9/18-19	98.3	-0.67	0.65	-0.38	0.70	3.45	0.39	-0.7	4.0	5.7	7.7	27.4	3.5

3.8 aC/sC

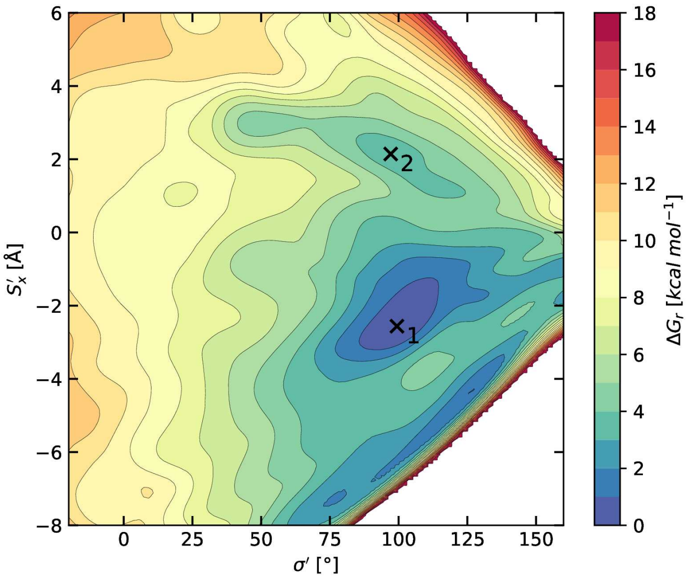

Figure SB18.1: Free energy surface for the aC/sC base pair. Labeled crosses show position of selected free energy minima (thermodynamic states). Free energy isolines are spaced by 1 kcal mol^{-1}.

Table SB18.1: Positions of selected free minima on the free energy surface for the aC/sC base pair. IDs correspond to the selected free energy minima shown in Figure SB18.1. Confidence interval of the free energy ΔG_r is provided at three standard deviations.

ID	σ' [°]	S'_x [Å]	ΔG_r [$kcal\ mol^{-1}$]
1	99.4	-2.56	0.00±0.00
2	97.1	2.14	3.47±0.11

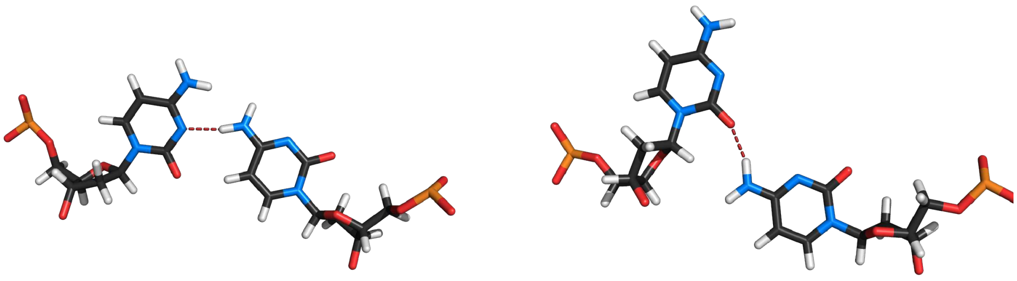

ID 1: $\sigma' = 99.4°$, S'_x = -2.56 Å   ID 2: $\sigma' = 97.1°$, S'_x = 2.14 Å

Figure SB18.2: Average geometries representing selected free energy minima for the aC/sC base pair. IDs correspond to the selected free energy minima shown in Figure SB18.1. Major and minor grooves are top and bottom, respectively. View direction is along the z-axis of DNA.

Table SB18.2: Number of analysed snapshots (N_{snap}), abundances (abu), average hydrogen bond distances and angles from hydrogen bond analysis provided by cpptraj for selected states of the aC/sC base pair. IDs correspond to the selected free energy minima shown in Figure SB18.1. Residue and atom numbering are provided in Figure SA1.

ID	N_{snap}	acceptor	H-donor	donor	abu [%]	d_{avg} [Å]	a_{avg} [°]
1	425	C7@N3	C20@H42	C20@N4	56.5	2.9	160.1
		C20@N4	C7@H41	C7@N4	1.4	3.0	150.4
2	414	C7@O2	C20@H41	C20@N4	65.7	2.8	156.5

Table SB18.3: *Simple* base-pair parameters for selected states of the aC/sC base pair. IDs corresponds to the free energy minima shown in Figure SB18.1. Residue numbering in base pairs A/B are provided in Figure SA1. Abundances (*abu*), average values ($\langle X \rangle$) and standard deviations of samples ($s(X)$) are calculated by 3DNA for Shear (S_x), Stretch (S_y), Stagger (S_z), Buckle (κ), Propeller (π), Opening (σ) employing the standard reference frames for the nucleobases. Only five central base pairs were included in the analysis for each state. Base pairs with a mismatch are highlighted in gray.

ID	A/B	abu [%]	$\langle S_x \rangle$	$s(S_x)$	$\langle S_y \rangle$ [Å]	$s(S_y)$	$\langle S_z \rangle$	$s(S_z)$	$\langle \kappa \rangle$	$s(\kappa)$	$\langle \pi \rangle$ [°]	$s(\pi)$	$\langle \sigma \rangle$	$s(\sigma)$
1	5/22	100.0	-0.18	0.27	0.03	0.12	-0.01	0.40	4.0	8.8	-17.7	6.8	-0.4	5.0
	6/21	100.0	-0.18	0.29	0.02	0.14	-0.05	0.42	-2.7	10.1	-18.0	7.9	3.2	6.8
	7/19	1.6	0.74	0.61	-0.02	0.17	-1.66	0.38	-9.9	6.1	-11.1	8.3	-32.6	3.0
	7/20	98.3	-2.55	0.19	1.81	0.22	0.60	0.67	-0.9	10.6	-14.9	9.2	-80.8	3.5
	8/19	98.1	0.12	0.48	0.03	0.20	0.02	0.48	1.6	11.0	-17.2	8.1	0.8	6.5
	9/18	100.0	0.18	0.27	0.04	0.13	0.01	0.38	-2.2	8.8	-16.0	6.9	-0.4	5.1
2	5/22	100.0	-0.20	0.26	0.02	0.12	0.05	0.40	-2.9	9.7	-18.1	7.5	-0.1	5.4
	6/21	100.0	-0.03	0.64	0.03	0.21	-0.03	0.56	-10.6	11.4	-10.2	11.8	-1.3	5.9
	7/20	99.5	2.18	0.21	2.93	0.38	0.29	0.82	-5.2	13.4	-1.3	13.4	-83.5	4.6
	8/18	5.6	0.16	0.92	0.03	0.14	-0.37	0.40	-11.6	24.6	11.8	32.6	-0.9	9.1
	8/19	82.4	-3.16	2.51	1.20	1.02	-0.21	0.76	-6.8	13.2	-2.9	13.1	6.7	9.8
	9/18	94.2	-0.45	1.62	0.27	0.59	0.09	0.57	-1.1	10.4	-7.5	10.2	-0.1	6.4
	9/19	4.3	-6.77	0.25	2.97	0.47	1.53	0.31	0.5	6.3	28.3	9.1	46.5	5.9

Table SB18.4: *Simple* step parameters for selected states of the aC/sC base pair. IDs corresponds to the free energy minima shown in Figure SB18.1. Residue numbering in base pairs and steps A1-B1/A2-B2 are provided in Figure SA1. Abundances (*abu*), average values ($\langle X \rangle$) and standard deviations of samples ($s(X)$) are calculated by 3DNA for Shift (D_x), Slide (D_y), Rise (D_z), Tilt (τ), Roll (ρ), Twist (ω) employing the standard reference frames for the nucleobases. Only five central base pairs were included in the analysis for each state. Steps including a mismatch are highlighted in gray.

ID	A1-B1<br>A2-B2	abu<br>[%]	$\langle D_x \rangle$	$s(D_x)$	$\langle D_y \rangle$<br>[Å]	$s(D_y)$	$\langle D_z \rangle$	$s(D_z)$	$\langle \tau \rangle$	$s(\tau)$	$\langle \rho \rangle$<br>[°]	$s(\rho)$	$\langle \omega \rangle$	$s(\omega)$
	5-6/21-22	100.0	0.62	0.71	-0.32	0.53	3.38	0.28	3.0	4.0	2.4	5.2	37.0	4.5
	6-7/19-21	1.6	0.65	0.43	-0.83	0.22	5.28	0.35	-15.7	4.9	4.7	5.2	56.6	2.7
	6-7/20-21	98.3	1.85	0.63	-1.10	0.51	3.22	0.29	-1.6	4.8	0.5	5.5	30.7	5.1
1	7-8/19-20	98.1	-1.13	0.62	1.65	0.59	3.16	0.34	1.6	5.5	9.9	6.7	37.5	6.5
	7-9/18-19	1.6	-0.74	0.50	-1.86	0.43	4.54	0.39	9.8	4.7	-4.1	5.0	52.8	3.6
	7-9/18-20	0.2	-0.26	0.00	0.72	0.00	6.71	0.00	9.1	0.0	1.5	0.0	78.1	0.0
	8-9/18-19	98.1	-0.62	0.71	-0.45	0.66	3.36	0.28	-2.1	4.4	-1.8	5.8	34.5	5.0
	5-6/21-22	100.0	0.61	0.64	-0.26	0.51	3.54	0.31	3.4	4.1	0.1	6.0	36.4	3.9
	6-7/20-21	99.5	2.43	0.55	1.00	0.61	3.53	0.36	-3.6	5.4	-0.1	5.0	13.3	5.4
	6-8/19-21	0.2	-0.63	0.00	-1.03	0.00	5.86	0.00	2.1	0.0	15.8	0.0	49.0	0.0
2	7-8/19-20	76.6	-1.88	1.17	-2.12	0.93	3.84	1.29	0.2	7.4	-1.4	15.7	56.3	9.6
	7-9/18-20	1.0	-1.74	0.50	-2.75	1.41	6.30	0.30	-16.4	16.2	1.3	16.1	93.7	7.2
	7-9/19-20	0.7	-1.39	0.28	-3.06	1.36	5.32	0.53	-27.2	4.1	-11.2	7.2	101.3	7.5
	8-9/18-19	82.4	-1.03	0.71	-0.09	0.76	3.42	0.37	-1.9	4.7	0.8	5.9	22.0	7.2
	9-8/18-19	4.3	1.66	0.25	-0.33	0.29	2.07	0.24	-5.1	4.5	5.2	12.3	-41.4	3.1

3.9 aC/sT

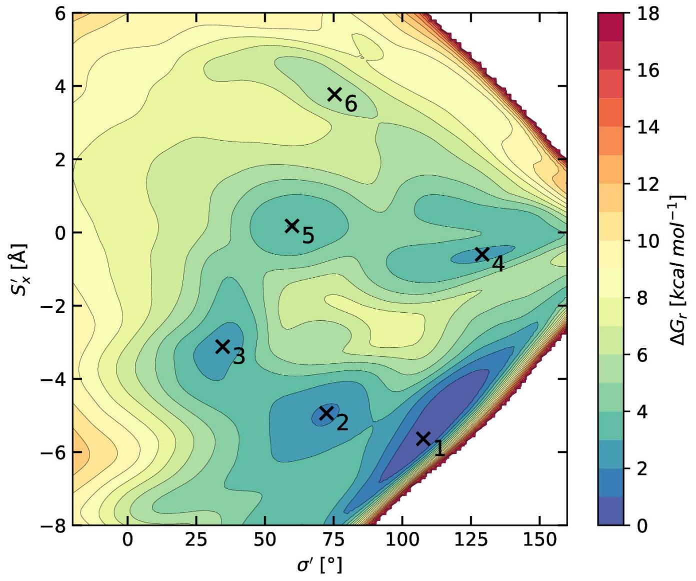

Figure SB19.1: Free energy surface for the aC/sT base pair. Labeled crosses show position of selected free energy minima (thermodynamic states). Free energy isolines are spaced by 1 kcal mol^{-1}.

Table SB19.1: Positions of selected free minima on the free energy surface for the aC/sT base pair. IDs correspond to the selected free energy minima shown in Figure SB19.1. Confidence interval of the free energy ΔG_r is provided at three standard deviations.

ID	σ' [°]	S'_x [Å]	ΔG_r [$kcal\ mol^{-1}$]
1	107.6	-5.64	0.00±0.00
2	72.4	-4.94	1.88±0.17
3	34.6	-3.12	2.47±0.18
4	129.1	-0.60	2.76±0.19
5	59.9	0.18	2.99±0.19
6	75.4	3.77	5.04±0.20

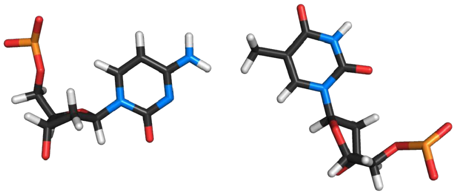

ID 1: $\sigma' = 107.6°$, S'_x = -5.64 Å

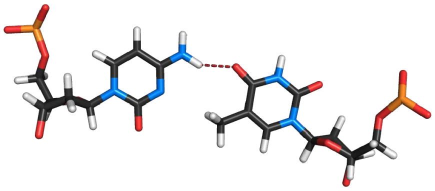

ID 2: $\sigma' = 72.4°$, S'_x = -4.94 Å

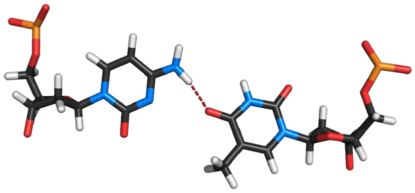

ID 3: $\sigma' = 34.6°$, S'_x = -3.12 Å

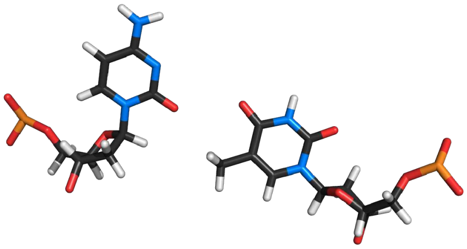

ID 4: $\sigma' = 129.1°$, S'_x = -0.60 Å

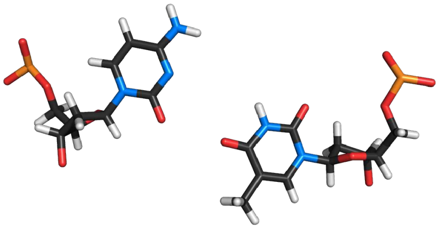

ID 5: $\sigma' = 59.9°$, S'_x = 0.18 Å

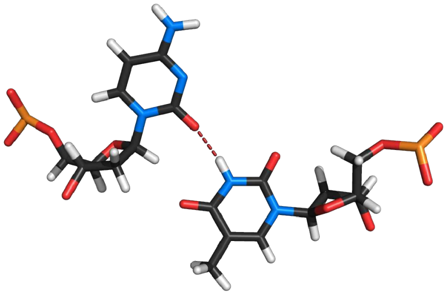

ID 6: $\sigma' = 75.4°$, S'_x = 3.77 Å

Figure SB19.2: Average geometries representing selected free energy minima for the aC/sT base pair. IDs correspond to the selected free energy minima shown in Figure SB19.1. Major and minor grooves are top and bottom, respectively. View direction is along the z-axis of DNA.

Table SB19.2: Number of analysed snapshots (N_{snap}), abundances (abu), average hydrogen bond distances and angles from hydrogen bond analysis provided by cpptraj for selected states of the aC/sT base pair. IDs correspond to the selected free energy minima shown in Figure SB19.1. Residue and atom numbering are provided in Figure SA1.

ID	N_{snap}	acceptor	H-donor	donor	abu [%]	d_{avg} [Å]	a_{avg} [°]
1	228	—	—	—	—	—	—
2	584	T20@O4	C7@H41	C7@N4	45.2	2.9	146.9
3	366	T20@O4	C7@H41	C7@N4	33.9	2.9	160.7
4	696	—	—	—	—	—	—
5	375	—	—	—	—	—	—
6	473	C7@O2	T20@H3	T20@N3	75.7	2.9	162.6

Table SB19.3: *Simple* base-pair parameters for selected states of the aC/sT base pair. IDs corresponds to the free energy minima shown in Figure SB19.1. Residue numbering in base pairs A/B are provided in Figure SA1. Abundances (*abu*), average values ($\langle X \rangle$) and standard deviations of samples ($s(X)$) are calculated by 3DNA for Shear (S_x), Stretch (S_y), Stagger (S_z), Buckle (κ), Propeller (π), Opening (σ) employing the standard reference frames for the nucleobases. Only five central base pairs were included in the analysis for each state. Base pairs with a mismatch are highlighted in gray.

ID	A/B	abu [%]	$\langle S_x \rangle$	$s(S_x)$	$\langle S_y \rangle$ [Å]	$s(S_y)$	$\langle S_z \rangle$	$s(S_z)$	$\langle \kappa \rangle$	$s(\kappa)$	$\langle \pi \rangle$ [°]	$s(\pi)$	$\langle \sigma \rangle$	$s(\sigma)$
1	5/22	100.0	-0.16	0.26	0.02	0.11	-0.00	0.39	3.0	9.4	-13.2	7.9	0.6	5.1
	6/21	100.0	-0.08	0.27	0.03	0.12	-0.22	0.44	4.8	10.8	-10.7	7.6	1.8	5.0
	8/19	100.0	0.19	0.24	0.05	0.13	-0.18	0.43	10.6	10.2	-11.5	8.1	0.7	5.3
	9/18	100.0	0.17	0.26	0.02	0.13	-0.04	0.42	3.6	9.7	-17.4	7.2	0.1	5.0
2	5/22	100.0	-0.17	0.26	0.02	0.12	0.02	0.41	-0.4	9.4	-16.7	7.1	-0.1	5.0
	6/21	100.0	-0.17	0.28	0.02	0.12	-0.10	0.46	-5.0	11.1	-12.8	9.7	0.8	5.8
	7/20	99.8	-4.94	0.25	2.13	0.27	-0.34	0.61	-4.4	12.0	-3.5	11.5	-107.8	4.2
	8/19	100.0	0.16	0.28	0.05	0.14	0.10	0.43	8.6	10.7	-9.1	8.3	1.8	5.6
	9/18	100.0	0.15	0.26	0.03	0.12	0.07	0.38	3.2	9.3	-18.6	7.2	-0.6	4.9
3	5/22	100.0	-0.20	0.27	0.03	0.12	0.02	0.39	-1.2	8.7	-19.6	6.9	0.0	5.4
	6/21	100.0	-0.23	0.27	0.03	0.11	-0.09	0.43	-11.7	10.0	-20.9	7.7	-1.8	5.3
	7/20	100.0	-3.13	0.25	0.27	0.22	-0.31	0.52	-1.6	9.5	-20.2	15.0	-145.2	3.2
	8/19	100.0	0.30	0.28	0.05	0.12	0.12	0.47	8.8	10.0	-11.5	7.4	1.3	4.7
	9/18	100.0	0.20	0.27	0.03	0.12	0.15	0.42	1.5	9.7	-19.2	6.9	-1.2	5.3
4	5/22	100.0	-0.21	0.26	0.04	0.12	0.01	0.41	3.0	9.2	-18.0	6.7	0.5	4.8
	6/21	100.0	-0.44	0.35	0.05	0.14	-0.10	0.46	-2.9	10.4	-15.3	8.3	1.8	5.3
	7/20	93.5	-0.60	0.14	4.57	0.42	0.07	0.79	12.8	13.3	-12.8	9.1	-51.4	6.3
	8/19	99.4	-0.01	0.83	0.05	0.30	-0.04	0.45	6.6	10.5	-8.7	9.2	2.9	5.5
	9/18	100.0	0.20	0.27	0.04	0.12	0.05	0.39	1.8	9.9	-17.5	7.2	-0.4	4.9
5	5/22	100.0	-0.18	0.27	0.02	0.12	0.02	0.40	-6.4	10.0	-22.0	7.5	-1.9	4.9
	6/21	99.2	-0.27	0.30	0.09	0.19	-0.23	0.39	-25.9	9.2	-30.7	8.7	-4.0	7.0
	7/20	99.2	0.15	0.23	1.38	0.32	0.14	0.70	-22.2	11.0	-18.1	10.0	-119.9	4.3
	7/21	0.3	4.19	0.00	0.99	0.00	2.20	0.00	-23.9	0.0	-34.5	0.0	-1.6	0.0
	8/19	100.0	0.20	0.27	0.01	0.11	0.05	0.45	4.7	9.4	-8.7	8.3	3.0	4.8
	9/18	100.0	0.20	0.28	0.06	0.13	0.04	0.39	3.6	8.6	-14.8	7.2	-1.1	4.8
6	5/22	100.0	-0.20	0.31	0.03	0.14	-0.10	0.41	-5.6	10.1	-20.0	8.0	-1.8	5.5
	6/21	100.0	2.81	3.11	0.85	0.98	-0.22	0.49	-5.4	11.2	-21.2	10.2	-13.2	18.1
	7/19	0.8	0.71	0.59	-0.66	0.28	-0.54	0.77	4.3	18.4	-31.1	4.5	-5.8	2.2
	7/20	97.7	3.78	0.16	1.67	0.24	0.52	0.71	8.1	12.5	-11.8	10.3	-104.6	3.4
	8/19	96.4	-0.89	1.88	0.28	0.72	-0.28	0.66	2.5	14.6	-11.2	13.8	6.8	11.3
	9/18	100.0	0.17	0.26	0.03	0.13	-0.09	0.46	1.0	11.0	-12.9	9.4	0.4	5.8

Table SB19.4: *Simple* step parameters for selected states of the aC/sT base pair. IDs corresponds to the free energy minima shown in Figure SB19.1. Residue numbering in base pairs and steps A1-B1/A2-B2 are provided in Figure SA1. Abundances (*abu*), average values ($\langle X \rangle$) and standard deviations of samples ($s(X)$) are calculated by 3DNA for Shift (D_x), Slide (D_y), Rise (D_z), Tilt (τ), Roll (ρ), Twist (ω) employing the standard reference frames for the nucleobases. Only five central base pairs were included in the analysis for each state. Steps including a mismatch are highlighted in gray.

ID	A1-B1	abu	$\langle D_x \rangle$	$s(D_x)$	$\langle D_y \rangle$	$s(D_y)$	$\langle D_z \rangle$	$s(D_z)$	$\langle \tau \rangle$	$s(\tau)$	$\langle \rho \rangle$	$s(\rho)$	$\langle \omega \rangle$	$s(\omega)$
	A2-B2	[%]			[Å]						[°]			
	5-6/21-22	100.0	0.31	0.62	-0.69	0.57	3.32	0.30	2.3	4.1	0.2	5.0	32.4	4.8
1	6-8/19-21	98.7	-0.05	0.79	-0.46	0.96	6.64	0.39	-3.0	6.5	4.3	7.3	64.5	6.1
	8-9/18-19	100.0	-0.68	0.54	-0.21	0.47	3.43	0.27	-3.9	4.1	2.0	6.0	40.1	3.1
	5-6/21-22	100.0	0.61	0.62	-0.38	0.57	3.39	0.30	3.1	4.4	0.3	5.8	37.2	4.7
	6-7/20-21	99.8	0.00	0.72	-1.25	0.57	3.19	0.41	0.8	6.0	5.5	6.9	31.6	5.9
2	6-8/19-21	0.2	-1.02	0.00	0.50	0.00	5.90	0.00	4.9	0.0	7.9	0.0	51.0	0.0
	7-8/19-20	99.8	0.50	0.78	1.93	0.65	3.28	0.42	-3.6	5.0	9.7	6.3	26.4	5.3
	8-9/18-19	100.0	-0.85	0.59	-0.13	0.53	3.40	0.28	-3.0	4.0	-0.1	5.8	39.1	4.0
	5-6/21-22	100.0	0.18	0.50	-0.46	0.46	3.47	0.28	3.1	4.2	2.3	5.0	36.5	4.2
3	6-7/20-21	100.0	-1.70	0.59	-0.17	0.44	2.83	0.43	-5.8	6.4	5.4	6.9	34.8	6.4
	7-8/19-20	100.0	1.72	0.71	0.89	0.39	3.37	0.42	-2.6	5.1	-3.4	10.2	28.7	3.8
	8-9/18-19	100.0	-0.77	0.46	-0.04	0.38	3.42	0.29	-4.0	4.0	-3.9	5.8	42.2	3.0
	5-6/21-22	100.0	0.84	0.45	-0.15	0.43	3.33	0.29	4.2	4.0	0.9	5.5	39.2	3.6
	6-7/20-21	93.5	3.03	0.55	-0.29	0.44	3.15	0.32	4.2	5.2	5.2	4.4	18.7	4.0
4	6-8/19-21	2.6	-0.26	0.86	-2.70	1.36	6.51	0.43	5.3	4.1	7.7	10.4	57.2	3.7
	7-8/19-20	93.1	-2.26	0.68	0.45	0.69	2.65	0.46	-2.9	6.7	22.7	8.7	34.1	6.7
	7-9/18-20	0.3	-2.39	0.68	-1.90	1.90	6.16	0.05	-0.8	5.5	14.7	4.8	65.8	7.6
	8-9/18-19	99.4	-0.87	0.61	-0.13	0.53	3.42	0.29	-3.5	3.8	-0.5	5.4	36.6	4.8
	5-6/21-22	99.2	-0.04	0.56	-0.40	0.47	3.73	0.36	3.2	4.3	7.8	6.3	37.2	3.8
	5-7/21-22	0.3	-2.32	0.00	-0.64	0.00	5.73	0.00	-1.6	0.0	8.1	0.0	48.1	0.0
	6-7/20-21	98.7	0.46	0.51	0.00	0.38	3.46	0.38	-4.4	4.7	3.4	5.5	30.5	3.6
5	6-8/19-21	0.5	1.96	0.69	1.26	0.60	6.01	0.46	-1.5	12.8	-4.8	5.9	69.4	5.8
	7-8/19-20	99.2	1.08	0.50	0.95	0.45	2.54	0.39	2.5	6.1	1.5	6.4	34.6	5.2
	7-8/19-21	0.3	2.78	0.00	0.13	0.00	3.60	0.00	-0.9	0.0	-7.0	0.0	58.0	0.0
	8-9/18-19	100.0	-1.12	0.47	0.31	0.40	3.32	0.27	-3.1	4.0	1.8	5.4	38.2	3.6
	5-6/21-22	100.0	-0.38	1.08	-0.43	0.62	3.66	0.41	0.8	5.5	5.7	5.2	30.0	5.7
	6-7/19-21	0.8	3.71	0.52	-0.66	0.23	3.90	0.81	-8.6	6.4	4.3	6.5	59.9	10.3
	6-7/20-21	97.7	1.59	0.88	0.91	0.49	3.19	0.40	-2.0	7.0	2.6	5.3	24.6	6.4
	6-8/19-21	0.8	0.88	0.73	-1.98	2.22	6.04	0.61	-9.7	6.4	11.0	10.7	64.5	1.9
6	7-8/19-20	94.9	0.47	1.32	-0.47	1.78	3.22	0.65	5.7	8.0	11.6	12.1	48.4	7.0
	7-9/18-8	0.2	0.70	0.00	-0.42	0.00	2.64	0.00	-8.8	0.0	23.1	0.0	130.6	0.0
	7-9/18-19	0.8	-1.90	0.93	-0.06	0.69	5.13	1.36	15.1	5.3	11.5	3.0	48.0	3.3
	7-9/18-20	1.1	1.36	1.14	-1.53	1.28	6.78	0.33	17.5	7.8	-20.3	21.1	82.8	6.0
	8-9/18-19	96.4	-0.89	0.84	-0.14	0.64	3.50	0.37	-2.9	4.9	3.7	7.9	29.9	6.6

3.10 aT/sT

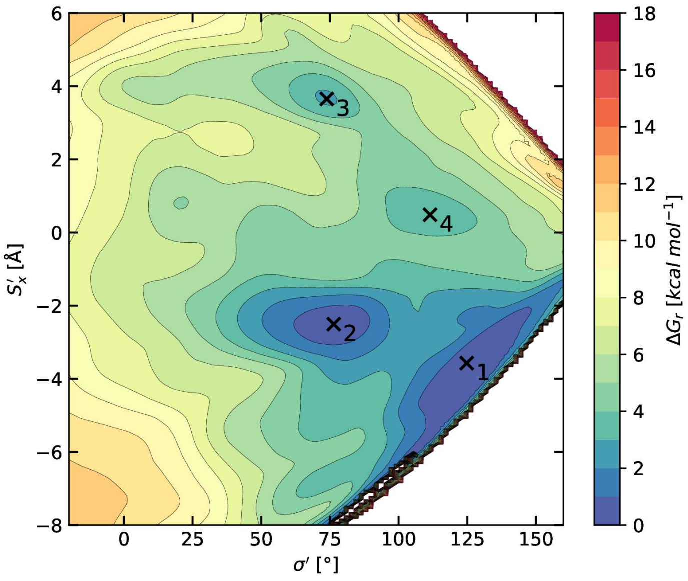

Figure SB20.1: Free energy surface for the aT/sT base pair. Labeled crosses show position of selected free energy minima (thermodynamic states). Free energy isolines are spaced by 1 kcal mol^{-1}.

Table SB20.1: Positions of selected free minima on the free energy surface for the aT/sT base pair. IDs correspond to the selected free energy minima shown in Figure SB20.1. Confidence interval of the free energy ΔG_r is provided at three standard deviations.

ID	σ' [°]	S'_x [Å]	ΔG_r [$kcal\ mol^{-1}$]
1	124.8	-3.57	0.00±0.00
2	76.4	-2.51	0.25±0.19
3	73.9	3.65	2.83±0.21
4	111.4	0.49	3.09±0.20

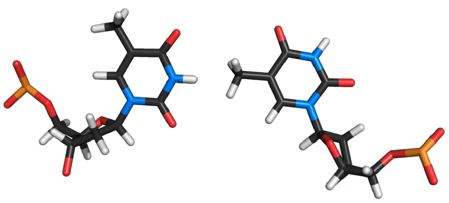

ID 1: $\sigma' = 124.8°$, S'_x = -3.57 Å

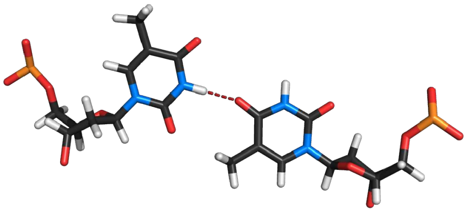

ID 2: $\sigma' = 76.4°$, S'_x = -2.51 Å

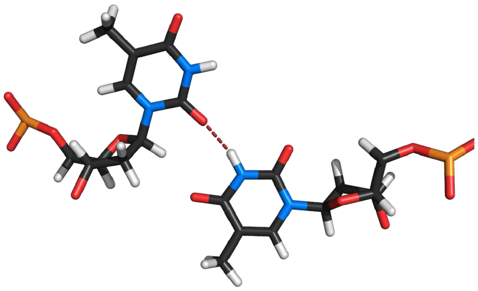

ID 3: $\sigma' = 73.9°$, S'_x = 3.65 Å

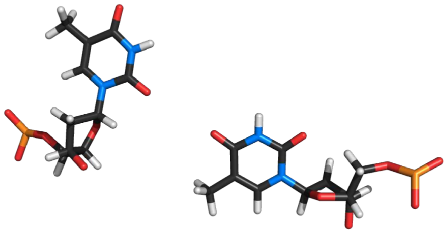

ID 4: $\sigma' = 111.4°$, S'_x = 0.49 Å

Figure SB20.2: Average geometries representing selected free energy minima for the aT/sT base pair. IDs correspond to the selected free energy minima shown in Figure SB20.1. Major and minor grooves are top and bottom, respectively. View direction is along the z-axis of DNA.

Table SB20.2: Number of analysed snapshots (N_{snap}), abundances (abu), average hydrogen bond distances and angles from hydrogen bond analysis provided by cpptraj for selected states of the aT/sT base pair. IDs correspond to the selected free energy minima shown in Figure SB20.1. Residue and atom numbering are provided in Figure SA1.

ID	N_{snap}	acceptor	H-donor	donor	abu [%]	d_{avg} [Å]	a_{avg} [°]
1	467	—	—	—	—	—	—
2	316	T20@O4	T7@H3	T7@N3	76.9	2.9	163.7
3	121	T7@O2	T20@H3	T20@N3	76.9	2.9	161.4
4	314	—	—	—	—	—	—

Table SB20.3: *Simple* base-pair parameters for selected states of the aT/sT base pair. IDs corresponds to the free energy minima shown in Figure SB20.1. Residue numbering in base pairs A/B are provided in Figure SA1. Abundances (*abu*), average values ($\langle X \rangle$) and standard deviations of samples ($s(X)$) are calculated by 3DNA for Shear (S_x), Stretch (S_y), Stagger (S_z), Buckle (κ), Propeller (π), Opening (σ) employing the standard reference frames for the nucleobases. Only five central base pairs were included in the analysis for each state. Base pairs with a mismatch are highlighted in gray.

ID	A/B	abu [%]	$\langle S_x \rangle$	$s(S_x)$	$\langle S_y \rangle$ [Å]	$s(S_y)$	$\langle S_z \rangle$	$s(S_z)$	$\langle \kappa \rangle$	$s(\kappa)$	$\langle \pi \rangle$ [°]	$s(\pi)$	$\langle \sigma \rangle$	$s(\sigma)$
1	5/22	100.0	-0.17	0.28	0.03	0.12	-0.02	0.39	3.5	8.8	-15.9	6.7	-0.2	4.9
	6/21	100.0	-0.16	0.27	0.03	0.13	-0.11	0.40	-0.8	10.5	-16.0	7.5	1.4	5.0
	8/19	100.0	0.19	0.27	0.03	0.13	-0.23	0.40	5.6	9.9	-9.5	7.6	1.6	5.2
	9/18	100.0	0.18	0.27	0.03	0.12	-0.07	0.38	1.6	9.5	-18.0	7.4	-0.5	5.3
2	5/22	100.0	-0.16	0.26	0.02	0.12	-0.07	0.39	-1.5	9.0	-16.2	7.2	-0.7	4.8
	6/21	100.0	-0.09	0.29	0.02	0.12	-0.13	0.42	-7.3	10.7	-11.0	9.0	-0.9	5.0
	7/20	100.0	-2.51	0.18	1.07	0.25	-0.23	0.59	1.4	10.4	-7.2	9.9	-103.8	4.1
	8/19	100.0	0.08	0.28	0.01	0.13	0.04	0.48	5.0	10.7	-11.2	9.1	2.6	5.9
	9/18	100.0	0.17	0.25	0.02	0.12	0.08	0.38	1.5	9.6	-16.5	7.7	-0.5	5.0
3	5/22	100.0	-0.18	0.27	0.02	0.12	0.02	0.38	-3.7	8.2	-18.5	7.5	-0.5	5.7
	6/21	100.0	0.70	1.94	0.22	0.66	-0.23	0.43	-10.3	10.2	-17.3	8.2	-2.6	11.9
	7/20	99.2	3.66	0.14	1.55	0.18	0.62	0.74	11.7	12.6	-10.8	10.4	-106.3	2.7
	8/19	100.0	-0.23	0.86	-0.01	0.35	-0.55	0.59	2.1	13.2	-16.3	12.6	11.6	10.0
	9/18	100.0	0.17	0.29	0.06	0.14	-0.22	0.40	0.9	11.7	-10.2	9.8	-0.2	5.9
4	5/22	100.0	-0.20	0.26	0.01	0.11	0.03	0.39	-6.7	9.7	-19.6	7.4	-0.6	4.8
	6/21	100.0	-0.19	0.28	0.09	0.15	0.14	0.48	-13.2	10.6	-5.8	10.4	-1.6	5.3
	7/20	24.5	0.49	0.16	4.65	0.34	0.03	0.93	-13.6	10.8	2.6	10.7	-69.9	4.2
	8/19	97.5	-0.53	1.50	0.19	0.56	-0.09	0.55	-0.1	11.6	-11.3	8.6	-0.1	6.1
	9/18	100.0	0.18	0.29	0.03	0.12	-0.00	0.40	-0.4	9.1	-15.6	7.7	-0.7	5.4

Table SB20.4: *Simple* step parameters for selected states of the aT/sT base pair. IDs corresponds to the free energy minima shown in Figure SB20.1. Residue numbering in base pairs and steps A1-B1/A2-B2 are provided in Figure SA1. Abundances (*abu*), average values ($\langle X \rangle$) and standard deviations of samples ($s(X)$) are calculated by 3DNA for Shift (D_x), Slide (D_y), Rise (D_z), Tilt (τ), Roll (ρ), Twist (ω) employing the standard reference frames for the nucleobases. Only five central base pairs were included in the analysis for each state. Steps including a mismatch are highlighted in gray.

ID	A1-B1<br>A2-B2	abu<br>[%]	$\langle D_x \rangle$	$s(D_x)$	$\langle D_y \rangle$<br>[Å]	$s(D_y)$	$\langle D_z \rangle$	$s(D_z)$	$\langle \tau \rangle$	$s(\tau)$	$\langle \rho \rangle$<br>[°]	$s(\rho)$	$\langle \omega \rangle$	$s(\omega)$
1	5-6/21-22	100.0	0.22	0.49	-0.69	0.47	3.39	0.29	1.6	3.9	0.5	4.7	34.8	4.0
	6-8/19-21	99.8	0.38	0.74	-1.16	0.73	6.56	0.38	1.8	5.5	8.7	6.9	62.9	5.0
	8-9/18-19	100.0	-0.71	0.54	-0.11	0.51	3.38	0.28	-3.6	3.9	1.3	5.3	38.9	3.5
2	5-6/21-22	100.0	0.36	0.56	-0.64	0.52	3.45	0.31	2.1	4.2	0.9	5.4	36.8	4.0
	6-7/20-21	100.0	1.19	0.48	-1.19	0.57	3.16	0.35	2.3	4.1	2.1	5.0	27.5	4.5
	7-8/19-20	100.0	-0.39	0.83	1.05	0.95	3.22	0.38	-2.8	5.3	14.9	9.1	31.1	6.3
	8-9/18-19	100.0	-0.69	0.62	-0.30	0.56	3.39	0.27	-2.5	4.4	-0.2	6.1	37.3	4.5
3	5-6/21-22	100.0	0.17	0.81	-0.33	0.55	3.52	0.29	4.0	4.0	3.4	5.1	33.0	5.4
	6-7/20-21	99.2	1.33	0.73	0.79	0.51	3.23	0.31	-4.6	5.1	2.9	5.6	19.6	4.7
	6-8/19-21	0.8	-0.02	0.00	-1.71	0.00	7.52	0.00	-13.2	0.0	23.2	0.0	70.4	0.0
	7-8/19-20	99.2	0.16	0.63	-1.21	0.58	3.30	0.56	8.2	6.9	13.4	9.1	48.4	6.4
	8-9/18-19	100.0	-0.95	0.71	-0.27	0.61	3.47	0.36	-3.3	4.8	7.1	7.2	28.3	5.4
4	5-6/21-22	100.0	0.82	0.59	-0.22	0.49	3.52	0.33	2.3	4.1	-0.7	6.0	38.3	3.2
	6-7/20-21	24.5	3.20	0.55	0.27	0.48	3.45	0.45	-0.5	5.2	2.2	5.2	10.9	4.6
	6-8/19-21	58.9	0.09	0.86	-2.91	1.06	6.21	0.48	0.3	6.2	15.5	9.0	55.3	5.3
	7-8/19-20	24.5	-3.03	0.86	-1.83	0.95	2.73	0.66	2.4	5.9	7.0	10.6	47.1	5.8
	8-9/18-19	97.5	-0.47	0.61	-0.68	0.58	3.41	0.29	-1.2	4.4	-0.8	5.5	30.8	5.2

4 Base Pairs in the *syn/anti* Orientations

4.1 sA/aC

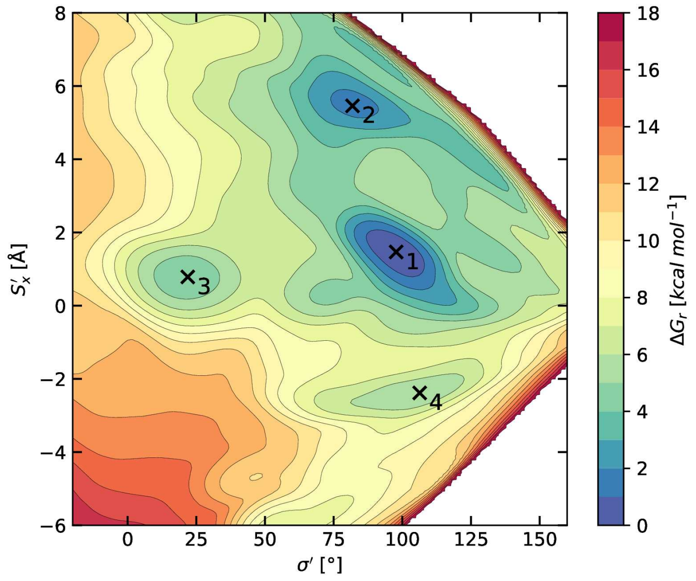

Figure SB21.1: Free energy surface for the sA/aC base pair. Labeled crosses show position of selected free energy minima (thermodynamic states). Free energy isolines are spaced by 1 kcal mol^{-1}.

Table SB21.1: Positions of selected free minima on the free energy surface for the sA/aC base pair. IDs correspond to the selected free energy minima shown in Figure SB21.1. Confidence interval of the free energy ΔG_r is provided at three standard deviations.

ID	σ' [°]	S'_x [Å]	ΔG_r [$kcal\ mol^{-1}$]
1	97.8	1.47	0.00±0.00
2	81.9	5.46	1.61±0.19
3	21.9	0.78	4.17±0.15
4	106.3	-2.38	5.14±0.17

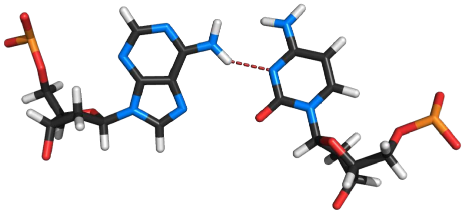

ID 1: $\sigma' = 97.8°$, $S'_x = 1.47$ Å

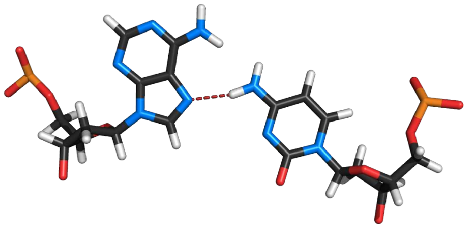

ID 2: $\sigma' = 81.9°$, $S'_x = 5.46$ Å

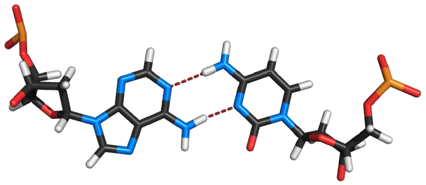

ID 3: $\sigma' = 21.9°$, $S'_x = 0.78$ Å

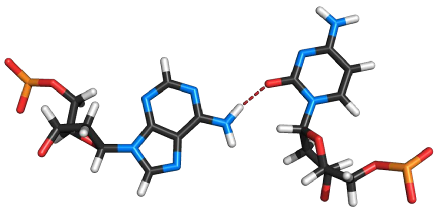

ID 4: $\sigma' = 106.3°$, $S'_x =$ -2.38 Å

Figure SB21.2: Average geometries representing selected free energy minima for the sA/aC base pair. IDs correspond to the selected free energy minima shown in Figure SB21.1. Major and minor grooves are top and bottom, respectively. View direction is along the z-axis of DNA.

Table SB21.2: Number of analysed snapshots (N_{snap}), abundances (abu), average hydrogen bond distances and angles from hydrogen bond analysis provided by cpptraj for selected states of the sA/aC base pair. IDs correspond to the selected free energy minima shown in Figure SB21.1. Residue and atom numbering are provided in Figure SA1.

ID	N_{snap}	acceptor	H-donor	donor	abu [%]	d_{avg} [Å]	a_{avg} [°]
1	155	C20@N3	A7@H62	A7@N6	34.8	2.9	147.8
2	248	A7@N7	C20@H41	C20@N4	36.3	2.9	159.3
3	311	C20@N3	A7@H61	A7@N6	65.9	2.9	161.7
		A7@N1	C20@H41	C20@N4	31.8	2.9	164.7
4	309	C20@O2	A7@H61	A7@N6	74.1	2.8	160.2

Table SB21.3: *Simple* base-pair parameters for selected states of the sA/aC base pair. IDs corresponds to the free energy minima shown in Figure SB21.1. Residue numbering in base pairs A/B are provided in Figure SA1. Abundances (*abu*), average values ($\langle X \rangle$) and standard deviations of samples ($s(X)$) are calculated by 3DNA for Shear (S_x), Stretch (S_y), Stagger (S_z), Buckle (κ), Propeller (π), Opening (σ) employing the standard reference frames for the nucleobases. Only five central base pairs were included in the analysis for each state. Base pairs with a mismatch are highlighted in gray.

ID	A/B	abu [%]	$\langle S_x \rangle$	$s(S_x)$	$\langle S_y \rangle$ [Å]	$s(S_y)$	$\langle S_z \rangle$	$s(S_z)$	$\langle \kappa \rangle$	$s(\kappa)$	$\langle \pi \rangle$ [°]	$s(\pi)$	$\langle \sigma \rangle$	$s(\sigma)$
	5/22	100.0	-0.19	0.26	0.08	0.13	-0.05	0.39	0.8	9.2	-16.3	6.5	0.7	4.8
	6/21	100.0	-0.14	0.27	0.03	0.11	0.05	0.39	-0.9	8.0	-13.3	9.0	7.2	5.0
1	7/20	100.0	-1.46	0.17	1.74	0.25	-1.05	0.51	-9.6	9.8	-17.7	7.9	81.9	3.0
	8/19	100.0	0.06	0.26	-0.03	0.12	-0.22	0.40	3.8	9.4	-22.3	7.1	0.8	4.9
	9/18	100.0	0.17	0.27	0.03	0.12	0.00	0.37	-4.9	8.2	-16.2	7.8	-0.5	5.8
	5/22	100.0	-0.18	0.24	0.03	0.12	-0.04	0.38	0.7	8.5	-18.1	7.3	0.3	5.0
	6/21	100.0	-0.19	0.29	0.02	0.12	-0.05	0.39	-10.0	10.3	-16.8	7.7	1.3	5.0
2	7/20	99.6	-5.44	0.16	2.04	0.31	0.20	0.47	6.6	11.6	-7.9	10.4	97.6	3.9
	8/19	100.0	0.14	0.27	0.01	0.12	-0.14	0.46	-2.7	11.4	-10.8	9.8	5.0	6.0
	9/18	100.0	0.20	0.27	0.05	0.11	0.06	0.38	-3.1	8.6	-13.3	7.5	0.2	5.2
	5/22	100.0	-0.20	0.28	0.05	0.12	0.05	0.39	0.3	9.7	-16.8	7.5	-1.1	5.5
	6/21	100.0	-0.19	0.27	0.01	0.12	0.05	0.49	-3.9	12.2	-15.8	10.2	0.7	5.6
3	7/20	99.7	-0.74	0.18	0.14	0.13	0.46	0.55	-1.7	11.9	-2.0	19.9	158.7	3.5
	8/19	100.0	-0.05	0.97	0.11	0.38	-0.20	0.45	11.1	12.6	-20.5	11.3	-2.4	10.1
	9/18	100.0	0.16	0.27	0.03	0.13	0.08	0.44	4.8	10.7	-17.5	7.6	-1.6	5.2
	5/21	0.3	4.83	0.00	2.03	0.00	-0.94	0.00	-15.0	0.0	-20.6	0.0	-49.9	0.0
	5/22	99.3	0.07	1.10	0.12	0.39	-0.03	0.45	1.2	10.9	-13.7	10.4	-0.1	5.9
	6/21	96.8	1.00	2.31	0.36	0.88	0.09	0.63	-1.0	14.3	-9.7	12.0	4.2	10.2
4	7/20	99.0	2.39	0.15	4.52	0.32	-0.14	0.70	-1.2	13.3	-2.8	11.7	73.8	5.0
	8/19	100.0	-0.19	1.11	0.13	0.33	-0.00	0.46	10.7	11.3	-12.2	10.5	-1.4	6.6
	9/18	100.0	0.21	0.26	0.04	0.12	-0.02	0.40	3.1	9.8	-19.7	7.8	-0.8	5.2

Table SB21.4: *Simple* step parameters for selected states of the sA/aC base pair. IDs corresponds to the free energy minima shown in Figure SB21.1. Residue numbering in base pairs and steps A1-B1/A2-B2 are provided in Figure SA1. Abundances (*abu*), average values ($\langle X \rangle$) and standard deviations of samples ($s(X)$) are calculated by 3DNA for Shift (D_x), Slide (D_y), Rise (D_z), Tilt (τ), Roll (ρ), Twist (ω) employing the standard reference frames for the nucleobases. Only five central base pairs were included in the analysis for each state. Steps including a mismatch are highlighted in gray.

ID	A1-B1 A2-B2	abu [%]	$\langle D_x \rangle$	$s(D_x)$	$\langle D_y \rangle$ [Å]	$s(D_y)$	$\langle D_z \rangle$	$s(D_z)$	$\langle \tau \rangle$	$s(\tau)$	$\langle \rho \rangle$ [°]	$s(\rho)$	$\langle \omega \rangle$	$s(\omega)$
1	5-6/21-22	100.0	1.36	0.45	0.25	0.34	3.30	0.25	3.1	4.1	0.8	4.9	37.1	3.0
	6-7/20-21	100.0	-1.16	0.48	-0.23	0.37	2.96	0.24	-0.7	5.7	-7.7	5.0	-139.8	3.7
	7-8/19-20	100.0	-0.75	0.41	1.88	0.39	3.45	0.27	1.1	4.7	-5.0	4.0	-148.5	3.5
	8-9/18-19	100.0	-0.06	0.47	-0.71	0.54	3.44	0.31	-3.7	4.0	3.7	4.8	34.2	4.1
2	5-6/21-22	100.0	0.60	0.55	-0.30	0.52	3.49	0.30	3.2	4.1	2.6	5.3	38.8	3.9
	6-7/20-21	99.6	-1.73	0.60	-0.52	0.56	3.45	0.33	-5.7	5.8	-0.9	4.7	-149.7	3.7
	6-8/19-21	0.4	-0.32	0.00	-1.07	0.00	6.24	0.00	7.3	0.0	8.8	0.0	50.9	0.0
	7-8/19-20	99.6	-1.29	0.53	-0.31	0.56	3.11	0.40	2.6	6.3	-5.3	6.1	-143.8	5.9
	8-9/18-19	100.0	-1.09	0.65	0.05	0.55	3.29	0.28	-4.7	4.4	0.6	5.9	36.9	4.7
3	5-6/21-22	100.0	0.67	0.71	-0.10	0.67	3.39	0.31	3.0	4.3	-1.6	7.0	35.7	7.1
	6-7/20-21	99.7	-0.70	0.68	-2.50	1.10	3.25	0.36	-16.7	11.9	-1.6	7.9	-147.1	6.3
	6-8/19-21	0.3	-3.17	0.00	2.43	0.00	6.56	0.00	-10.0	0.0	18.4	0.0	48.2	0.0
	7-8/19-20	99.7	-0.43	0.57	-1.88	0.78	3.31	0.36	13.1	9.6	-1.6	6.2	-155.8	7.9
	8-9/18-19	98.4	-0.03	0.72	-0.46	0.58	3.45	0.36	-3.0	4.3	3.5	6.9	36.3	5.1
4	5-6/7-22	0.7	4.49	0.25	1.00	0.71	4.91	0.36	-12.7	2.3	1.6	0.4	0.8	2.5
	5-6/21-22	96.8	0.75	0.68	-0.34	0.75	3.44	0.31	0.7	4.7	0.1	5.7	30.5	7.9
	5-7/20-21	0.3	3.08	0.00	3.76	0.00	3.76	0.00	1.1	0.0	30.3	0.0	-92.8	0.0
	5-7/20-22	1.0	2.40	0.23	3.38	0.45	5.56	0.51	-11.1	3.9	12.4	7.6	-75.5	2.6
	6-7/20-21	92.9	2.19	0.92	1.66	0.92	2.79	0.71	-14.8	12.1	-0.1	7.3	-131.6	10.9
	6-8/19-7	0.7	-2.67	0.57	-1.66	0.08	3.49	0.12	15.3	1.7	3.5	1.6	113.4	5.5
	6-8/19-21	0.3	0.30	0.00	-3.58	0.00	6.49	0.00	-5.8	0.0	14.1	0.0	51.9	0.0
	7-8/19-20	99.0	1.52	0.66	2.20	0.75	3.38	0.35	2.4	4.6	-1.1	5.8	-164.6	34.5
	8-9/18-19	100.0	-0.60	0.68	-0.18	0.52	3.54	0.31	-2.4	3.8	1.7	5.8	36.2	4.7

4.2 sA/aG

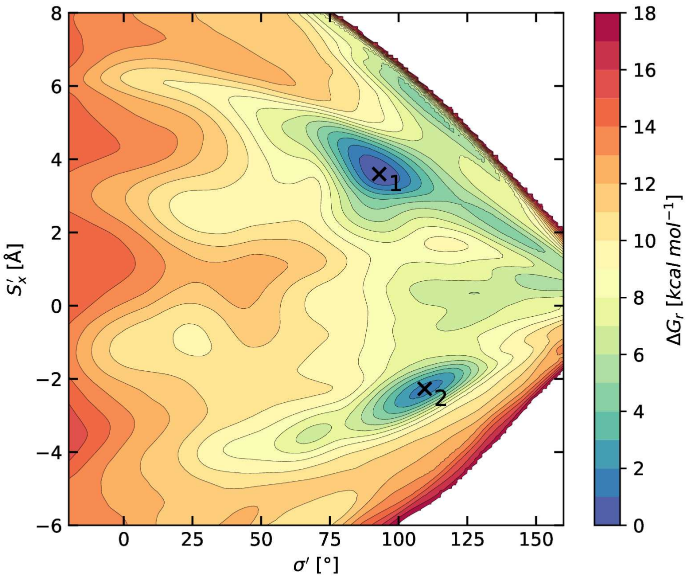

Figure SB22.1: Free energy surface for the sA/aG base pair. Labeled crosses show position of selected free energy minima (thermodynamic states). Free energy isolines are spaced by 1 kcal mol^{-1}.

Table SB22.1: Positions of selected free minima on the free energy surface for the sA/aG base pair. IDs correspond to the selected free energy minima shown in Figure SB22.1. Confidence interval of the free energy ΔG_r is provided at three standard deviations.

ID	σ' [°]	S'_x [Å]	ΔG_r [$kcal\ mol^{-1}$]
1	92.9	3.60	0.00±0.00
2	109.5	-2.27	1.53±0.17

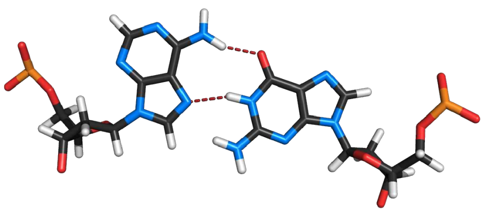

ID 1: $\sigma' = 92.9°$, $S'_x = 3.60$ Å

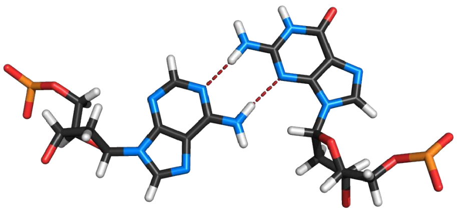

ID 2: $\sigma' = 109.5°$, $S'_x =$ -2.27 Å

Figure SB22.2: Average geometries representing selected free energy minima for the sA/aG base pair. IDs correspond to the selected free energy minima shown in Figure SB22.1. Major and minor grooves are top and bottom, respectively. View direction is along the z-axis of DNA.

Table SB22.2: Number of analysed snapshots (N_{snap}), abundances (abu), average hydrogen bond distances and angles from hydrogen bond analysis provided by cpptraj for selected states of the sA/aG base pair. IDs correspond to the selected free energy minima shown in Figure SB22.1. Residue and atom numbering are provided in Figure SA1.

ID	N_{snap}	acceptor	H-donor	donor	abu [%]	d_{avg} [Å]	a_{avg} [°]
1	125	G20@O6	A7@H62	A7@N6	76.0	2.9	155.0
		A7@N7	G20@H1	G20@N1	55.2	2.9	153.3
		A7@N7	G20@H21	G20@N2	8.0	3.0	149.4
2	109	A7@N1	G20@H22	G20@N2	67.9	2.9	161.0
		G20@N3	A7@H61	A7@N6	41.3	2.9	162.3

Table SB22.3: *Simple* base-pair parameters for selected states of the sA/aG base pair. IDs corresponds to the free energy minima shown in Figure SB22.1. Residue numbering in base pairs A/B are provided in Figure SA1. Abundances (*abu*), average values ($\langle X \rangle$) and standard deviations of samples ($s(X)$) are calculated by 3DNA for Shear (S_x), Stretch (S_y), Stagger (S_z), Buckle (κ), Propeller (π), Opening (σ) employing the standard reference frames for the nucleobases. Only five central base pairs were included in the analysis for each state. Base pairs with a mismatch are highlighted in gray.

ID	A/B	abu [%]	$\langle S_x \rangle$	$s(S_x)$	$\langle S_y \rangle$ [Å]	$s(S_y)$	$\langle S_z \rangle$	$s(S_z)$	$\langle \kappa \rangle$	$s(\kappa)$	$\langle \pi \rangle$ [°]	$s(\pi)$	$\langle \sigma \rangle$	$s(\sigma)$
	5/22	100.0	-0.17	0.26	0.03	0.11	-0.06	0.35	1.8	8.7	-17.3	6.7	0.7	5.2
	6/21	100.0	-0.13	0.27	0.04	0.13	-0.10	0.42	-5.4	10.2	-16.3	7.0	1.3	5.6
1	7/20	100.0	-3.59	0.10	2.63	0.15	0.18	0.37	6.3	8.4	-1.4	9.0	87.0	1.9
	8/19	100.0	0.11	0.29	0.02	0.12	-0.00	0.39	3.9	11.4	-7.9	7.6	1.1	5.0
	9/18	100.0	0.18	0.26	0.03	0.13	0.03	0.40	1.9	8.7	-17.2	8.1	-1.1	5.3
	5/22	100.0	-0.15	0.47	0.06	0.15	-0.03	0.44	0.0	11.0	-15.5	8.8	-0.1	5.0
	6/21	100.0	0.08	1.06	0.09	0.41	0.14	0.49	-4.2	12.4	-14.4	10.5	5.0	7.0
2	7/20	100.0	2.26	0.10	4.64	0.14	-0.02	0.58	11.5	9.8	-3.7	11.4	70.0	2.4
	8/19	100.0	0.01	0.52	0.02	0.14	-0.11	0.49	6.2	10.7	-10.5	9.5	-0.2	4.4
	9/18	100.0	0.19	0.25	0.04	0.12	-0.03	0.32	1.1	8.4	-19.2	7.0	-2.1	4.6

Table SB22.4: *Simple* step parameters for selected states of the sA/aG base pair. IDs corresponds to the free energy minima shown in Figure SB22.1. Residue numbering in base pairs and steps A1-B1/A2-B2 are provided in Figure SA1. Abundances (*abu*), average values ($\langle X \rangle$) and standard deviations of samples ($s(X)$) are calculated by 3DNA for Shift (D_x), Slide (D_y), Rise (D_z), Tilt (τ), Roll (ρ), Twist (ω) employing the standard reference frames for the nucleobases. Only five central base pairs were included in the analysis for each state. Steps including a mismatch are highlighted in gray.

ID	A1-B1 A2-B2	abu [%]	$\langle D_x \rangle$	$s(D_x)$	$\langle D_y \rangle$ [Å]	$s(D_y)$	$\langle D_z \rangle$	$s(D_z)$	$\langle \tau \rangle$	$s(\tau)$	$\langle \rho \rangle$	$s(\rho)$ [°]	$\langle \omega \rangle$	$s(\omega)$
1	5-6/21-22	100.0	0.40	0.59	-0.53	0.51	3.41	0.28	2.4	3.8	2.4	5.0	37.6	3.9
	6-7/20-21	100.0	-1.20	0.68	0.54	0.71	3.30	0.27	-4.5	5.2	0.3	4.4	-145.6	4.3
	7-8/19-20	100.0	-1.11	0.60	1.02	0.50	3.11	0.28	4.4	6.9	-0.2	5.0	-152.6	6.7
	8-9/18-19	100.0	-0.92	0.65	-0.08	0.49	3.35	0.26	-3.2	4.0	-0.3	5.4	36.6	5.4
2	5-6/21-22	100.0	0.87	0.63	-0.32	0.66	3.42	0.35	1.1	4.1	2.3	6.2	34.1	5.6
	6-7/20-21	98.2	1.93	0.87	0.80	0.69	2.85	0.38	-18.9	7.6	-0.4	5.7	-139.1	6.0
	7-8/19-20	100.0	2.03	0.77	1.80	0.62	3.47	0.28	1.1	3.9	1.8	4.0	-162.3	33.0
	8-9/18-19	100.0	-0.26	0.72	-0.34	0.59	3.48	0.31	-2.4	4.0	0.1	4.9	35.0	5.3

4.3 sA/aT

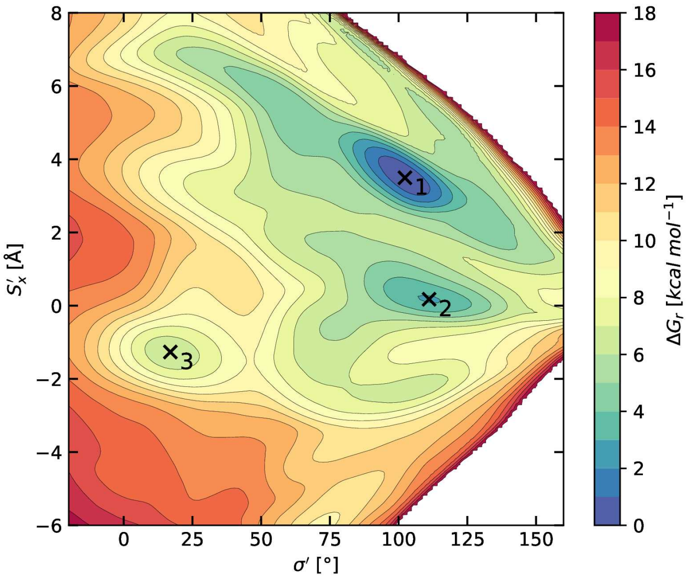

Figure SB23.1: Free energy surface for the sA/aT base pair. Labeled crosses show position of selected free energy minima (thermodynamic states). Free energy isolines are spaced by 1 kcal mol^{-1}.

Table SB23.1: Positions of selected free minima on the free energy surface for the sA/aT base pair. IDs correspond to the selected free energy minima shown in Figure SB23.1. Confidence interval of the free energy ΔG_r is provided at three standard deviations.

ID	σ' [°]	S'_x [Å]	ΔG_r [$kcal\ mol^{-1}$]
1	102.3	3.49	0.00±0.00
2	111.1	0.18	2.91±0.12
3	17.0	-1.27	6.30±0.12

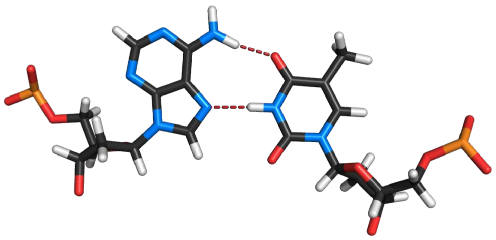

ID 1: $\sigma' = 102.3°$, $S'_x = 3.49$ Å

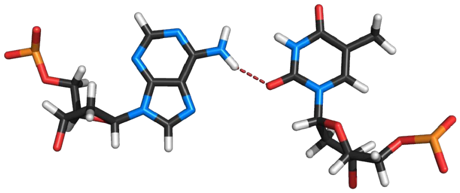

ID 2: $\sigma' = 111.1°$, $S'_x = 0.18$ Å

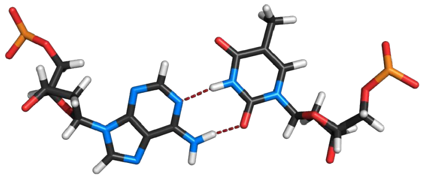

ID 3: $\sigma' = 17.0°$, $S'_x =$ -1.27 Å

Figure SB23.2: Average geometries representing selected free energy minima for the sA/aT base pair. IDs correspond to the selected free energy minima shown in Figure SB23.1. Major and minor grooves are top and bottom, respectively. View direction is along the z-axis of DNA.

Table SB23.2: Number of analysed snapshots (N_{snap}), abundances (abu), average hydrogen bond distances and angles from hydrogen bond analysis provided by cpptraj for selected states of the sA/aT base pair. IDs correspond to the selected free energy minima shown in Figure SB23.1. Residue and atom numbering are provided in Figure SA1.

ID	N_{snap}	acceptor	H-donor	donor	abu [%]	d_{avg} [Å]	a_{avg} [°]
1	189	T20@O4	A7@H62	A7@N6	87.3	2.8	160.0
		A7@N7	T20@H3	T20@N3	46.0	2.9	165.9
2	256	T20@O2	A7@H62	A7@N6	51.6	2.9	152.5
3	300	T20@O2	A7@H61	A7@N6	70.0	2.9	161.1
		A7@N1	T20@H3	T20@N3	69.0	2.9	161.3

Table SB23.3: *Simple* base-pair parameters for selected states of the sA/aT base pair. IDs corresponds to the free energy minima shown in Figure SB23.1. Residue numbering in base pairs A/B are provided in Figure SA1. Abundances (*abu*), average values ($\langle X \rangle$) and standard deviations of samples ($s(X)$) are calculated by 3DNA for Shear (S_x), Stretch (S_y), Stagger (S_z), Buckle (κ), Propeller (π), Opening (σ) employing the standard reference frames for the nucleobases. Only five central base pairs were included in the analysis for each state. Base pairs with a mismatch are highlighted in gray.

ID	A/B	abu [%]	$\langle S_x \rangle$	$s(S_x)$	$\langle S_y \rangle$ [Å]	$s(S_y)$	$\langle S_z \rangle$	$s(S_z)$	$\langle \kappa \rangle$	$s(\kappa)$	$\langle \pi \rangle$ [°]	$s(\pi)$	$\langle \sigma \rangle$	$s(\sigma)$
	5/22	100.0	-0.18	0.26	0.04	0.13	-0.08	0.41	3.8	8.4	-15.4	7.1	1.1	5.3
	6/21	100.0	-0.07	0.30	0.00	0.12	-0.19	0.43	-1.4	10.8	-14.3	6.4	4.2	5.9
1	7/20	100.0	-3.50	0.15	1.43	0.13	-0.11	0.53	6.1	8.7	-8.8	8.1	77.8	2.4
	8/19	100.0	0.12	0.26	-0.00	0.13	-0.13	0.41	3.4	8.9	-11.8	7.2	3.8	5.2
	9/18	100.0	0.21	0.26	0.06	0.13	-0.03	0.40	-0.6	8.0	-15.4	8.1	-0.7	4.7
	5/22	100.0	-0.16	0.28	0.03	0.12	-0.04	0.38	1.0	9.8	-14.4	7.4	-0.1	4.9
	6/21	100.0	-0.06	0.27	-0.03	0.13	-0.33	0.42	-1.5	9.9	-12.0	7.9	1.5	4.9
2	7/20	100.0	-0.17	0.11	3.20	0.33	-0.50	0.62	-0.1	10.7	-8.4	10.4	69.2	3.7
	8/19	100.0	0.15	0.27	0.03	0.12	0.06	0.41	10.5	10.4	-15.4	8.6	0.8	5.3
	9/18	100.0	0.22	0.23	0.03	0.12	-0.02	0.39	-0.8	8.9	-19.9	7.1	0.3	4.8
	5/22	100.0	-0.15	0.24	0.05	0.12	-0.05	0.41	-0.9	9.8	-14.1	7.9	0.5	5.3
	6/21	100.0	-0.08	0.28	-0.00	0.15	-0.13	0.63	-3.0	12.8	-12.2	10.2	3.1	6.6
	7/20	99.7	1.26	0.14	0.12	0.15	0.56	0.48	9.1	10.6	-12.4	14.6	163.1	3.0
3	8/19	98.3	-1.23	2.03	0.49	0.67	-0.17	0.62	11.6	11.5	-16.4	9.7	-11.6	18.6
	8/20	0.3	-0.19	0.00	-0.17	0.00	1.83	0.00	-4.0	0.0	-0.7	0.0	-8.1	0.0
	9/18	100.0	0.17	0.27	0.03	0.12	-0.01	0.38	7.9	8.9	-20.0	7.4	-2.3	5.0

Table SB23.4: *Simple* step parameters for selected states of the sA/aT base pair. IDs corresponds to the free energy minima shown in Figure SB23.1. Residue numbering in base pairs and steps A1-B1/A2-B2 are provided in Figure SA1. Abundances (*abu*), average values ($\langle X \rangle$) and standard deviations of samples ($s(X)$) are calculated by 3DNA for Shift (D_x), Slide (D_y), Rise (D_z), Tilt (τ), Roll (ρ), Twist (ω) employing the standard reference frames for the nucleobases. Only five central base pairs were included in the analysis for each state. Steps including a mismatch are highlighted in gray.

ID	A1-B1 A2-B2	abu [%]	$\langle D_x \rangle$	$s(D_x)$	$\langle D_y \rangle$ [Å]	$s(D_y)$	$\langle D_z \rangle$	$s(D_z)$	$\langle \tau \rangle$	$s(\tau)$	$\langle \rho \rangle$	$s(\rho)$ [°]	$\langle \omega \rangle$	$s(\omega)$
1	5-6/21-22	100.0	0.68	0.65	-0.47	0.65	3.38	0.27	2.8	4.2	1.9	5.0	35.6	4.5
	6-7/20-21	100.0	-1.83	0.65	0.47	0.78	3.47	0.32	-7.9	5.2	-1.1	5.0	-143.8	6.0
	7-8/19-20	100.0	-1.79	0.57	1.50	0.46	3.13	0.29	1.7	4.8	-1.3	4.3	-149.5	4.5
	8-9/18-19	100.0	-0.92	0.68	-0.16	0.58	3.35	0.25	-3.5	4.1	-0.3	5.3	36.6	5.0
2	5-6/21-22	100.0	0.44	0.66	-0.50	0.58	3.36	0.27	3.8	3.9	0.5	5.3	34.0	4.4
	6-7/20-21	100.0	-0.54	0.45	1.95	0.71	2.95	0.32	-12.0	8.6	-4.7	5.5	-136.1	7.9
	7-8/19-20	100.0	-0.17	0.55	2.67	0.47	3.26	0.29	0.8	5.5	0.3	4.5	-161.6	4.7
	8-9/18-19	100.0	-0.73	0.53	-0.14	0.46	3.52	0.26	-3.1	4.0	0.4	5.4	39.2	3.8
3	5-6/21-22	100.0	0.85	0.67	-0.20	0.58	3.38	0.35	2.6	5.0	3.0	6.3	34.9	4.7
	6-7/20-21	99.7	0.43	1.05	-3.25	0.93	3.59	0.60	-2.8	10.4	0.5	6.2	-138.0	5.8
	6-8/20-21	0.3	-3.01	0.00	0.03	0.00	4.41	0.00	9.4	0.0	8.6	0.0	45.5	0.0
	7-8/19-20	98.3	-0.63	0.67	-1.51	1.08	3.11	0.40	5.2	7.6	0.1	5.0	-155.5	5.3
	7-9/18-20	1.3	0.21	0.27	-1.67	0.26	6.62	0.31	1.6	3.2	1.5	3.1	-124.8	3.0
	8-9/18-19	98.3	0.25	1.14	-0.50	0.53	3.53	0.33	-1.5	4.5	3.1	5.7	33.7	5.0
	8-9/18-20	0.3	3.39	0.00	-1.04	0.00	4.13	0.00	-15.3	0.0	16.6	0.0	45.4	0.0

4.4 sG/aC

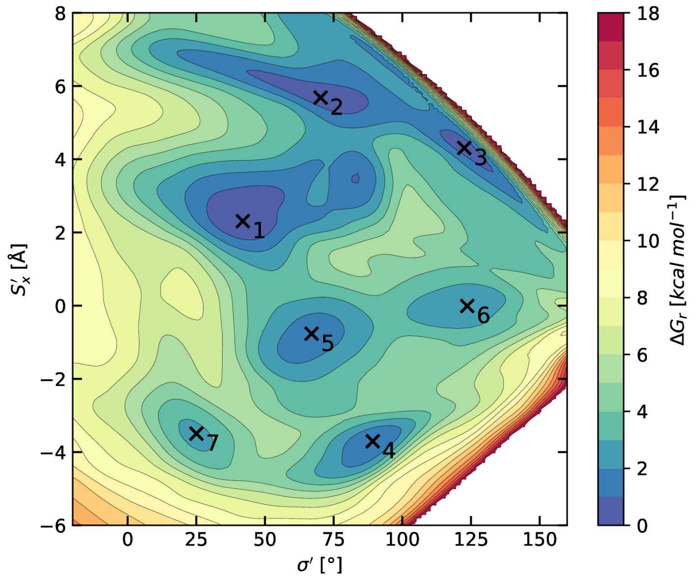

Figure SB24.1: Free energy surface for the sG/aC base pair. Labeled crosses show position of selected free energy minima (thermodynamic states). Free energy isolines are spaced by 1 kcal mol^{-1}.

Table SB24.1: Positions of selected free minima on the free energy surface for the sG/aC base pair. IDs correspond to the selected free energy minima shown in Figure SB24.1. Confidence interval of the free energy ΔG_r is provided at three standard deviations.

ID	σ' [°]	S'_x [Å]	ΔG_r [$kcal\ mol^{-1}$]
1	41.9	2.31	0.00±0.00
2	70.2	5.68	0.51±0.10
3	122.6	4.31	0.52±0.16
4	89.2	-3.70	0.99±0.12
5	67.0	-0.77	1.51±0.10
6	123.6	-0.01	2.20±0.11
7	25.0	-3.50	2.67±0.12

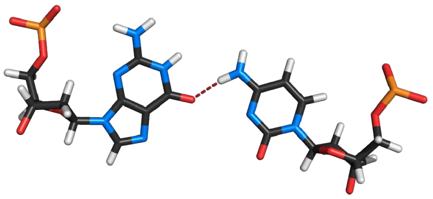

ID 1: $\sigma' = 41.9°$, $S'_x = 2.31$ Å

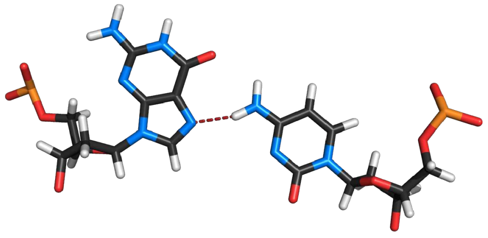

ID 2: $\sigma' = 70.2°$, $S'_x = 5.68$ Å

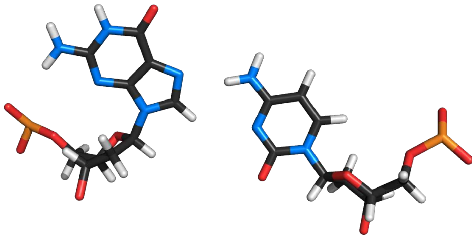

ID 3: $\sigma' = 122.6°$, $S'_x = 4.31$ Å

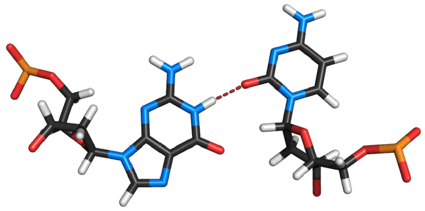

ID 4: $\sigma' = 89.2°$, $S'_x =$ -3.70 Å

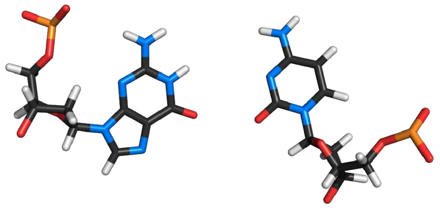

ID 5: $\sigma' = 67.0°$, $S'_x =$ -0.77 Å

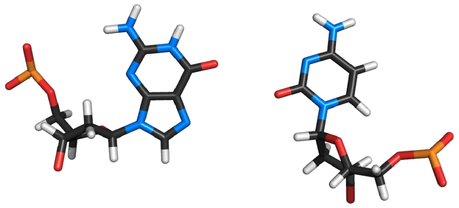

ID 6: $\sigma' = 123.6°$, $S'_x =$ -0.01 Å

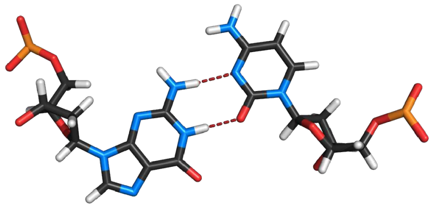

ID 7: $\sigma' = 25.0°$, $S'_x =$ -3.50 Å

Figure SB24.2: Average geometries representing selected free energy minima for the sG/aC base pair. IDs correspond to the selected free energy minima shown in Figure SB24.1. Major and minor grooves are top and bottom, respectively. View direction is along the z-axis of DNA.

Table SB24.2: Number of analysed snapshots (N_{snap}), abundances (abu), average hydrogen bond distances and angles from hydrogen bond analysis provided by cpptraj for selected states of the sG/aC base pair. IDs correspond to the selected free energy minima shown in Figure SB24.1. Residue and atom numbering are provided in Figure SA1.

ID	N_{snap}	acceptor	H-donor	donor	abu [%]	d_{avg} [Å]	a_{avg} [°]
1	313	G7@O6	C20@H41	C20@N4	65.5	2.9	160.7
		G7@OP2	G7@H22	G7@N2	16.9	2.9	166.2
2	729	G7@N7	C20@H41	C20@N4	40.2	2.9	154.0
		G7@O6	C20@H42	C20@N4	3.4	2.9	139.1
		G7@OP2	G7@H22	G7@N2	1.8	2.9	162.9
3	292	G7@N7	C20@H41	C20@N4	18.8	2.9	156.4
4	218	C20@O2	G7@H1	G7@N1	89.5	2.8	165.1
5	661	G7@OP2	G7@H22	G7@N2	13.2	2.9	167.0
6	600	G7@OP2	G7@H22	G7@N2	2.3	2.9	169.3
7	292	C20@O2	G7@H1	G7@N1	92.1	2.8	164.0
		C20@N3	G7@H21	G7@N2	49.0	2.9	164.9
		G7@OP2	G7@H22	G7@N2	7.5	2.9	167.4

Table SB24.3: *Simple* base-pair parameters for selected states of the sG/aC base pair. IDs corresponds to the free energy minima shown in Figure SB24.1. Residue numbering in base pairs A/B are provided in Figure SA1. Abundances (*abu*), average values ($\langle X\rangle$) and standard deviations of samples ($s(X)$) are calculated by 3DNA for Shear (S_x), Stretch (S_y), Stagger (S_z), Buckle (κ), Propeller (π), Opening (σ) employing the standard reference frames for the nucleobases. Only five central base pairs were included in the analysis for each state. Base pairs with a mismatch are highlighted in gray.

ID	A/B	abu [%]	$\langle S_x\rangle$	$s(S_x)$	$\langle S_y\rangle$ [Å]	$s(S_y)$	$\langle S_z\rangle$	$s(S_z)$	$\langle\kappa\rangle$	$s(\kappa)$	$\langle\pi\rangle$ [°]	$s(\pi)$	$\langle\sigma\rangle$	$s(\sigma)$
	5/22	100.0	-0.16	0.25	0.02	0.11	0.07	0.43	-3.6	9.3	-17.7	6.9	-0.4	5.2
	6/21	100.0	-0.19	0.28	0.04	0.12	-0.02	0.40	-3.9	8.6	-8.1	8.9	1.6	4.8
1	7/20	100.0	-2.31	0.14	0.67	0.19	0.27	0.39	-10.5	9.5	-9.9	9.0	138.0	3.2
	8/19	99.7	0.21	0.30	0.07	0.14	-0.01	0.41	15.9	10.8	-23.1	9.0	-0.6	7.4
	9/18	100.0	0.17	0.25	0.02	0.12	0.03	0.38	5.6	9.6	-20.2	7.5	-0.8	4.8
	5/22	100.0	-0.14	0.27	0.02	0.13	-0.02	0.40	0.7	9.2	-16.9	7.6	-0.2	5.2
	6/21	100.0	-0.22	0.29	0.03	0.12	-0.05	0.42	-8.8	11.2	-17.4	8.4	-0.1	5.5
2	7/20	99.9	-5.69	0.19	1.48	0.31	0.29	0.49	4.2	11.3	-9.1	10.2	109.8	6.3
	8/19	100.0	0.15	0.28	0.01	0.13	-0.04	0.45	0.6	10.8	-14.3	9.0	3.1	5.4
	9/18	100.0	0.24	0.25	0.06	0.12	0.03	0.39	-2.4	8.5	-17.1	7.2	-1.0	5.0
	5/22	100.0	-0.21	0.27	0.06	0.14	-0.10	0.37	2.6	8.9	-16.0	7.4	0.8	5.5
	6/21	100.0	-0.04	0.31	-0.02	0.16	-0.34	0.49	-3.1	11.4	-14.8	9.2	4.7	5.9
3	7/20	88.0	-4.30	0.20	4.43	0.46	-0.31	0.74	-6.5	10.6	-17.3	12.9	57.6	3.2
	8/19	100.0	0.06	0.27	-0.01	0.14	-0.34	0.49	-2.6	14.7	-10.5	9.8	3.4	6.1
	9/18	100.0	0.22	0.27	0.05	0.12	-0.13	0.43	-2.5	10.1	-13.9	8.2	-0.7	4.9
	5/21	4.1	-0.03	0.18	-0.24	0.16	-1.24	0.33	3.1	11.4	-3.7	10.6	2.7	4.2
	5/22	95.9	-0.17	0.26	0.03	0.13	-0.02	0.43	-2.4	10.1	-16.2	9.5	-0.4	5.3
	6/20	8.3	-0.44	0.32	-1.68	0.19	-0.70	0.54	-1.7	12.0	-19.7	7.3	11.6	4.7
4	6/21	91.3	0.06	0.79	0.05	0.36	-0.02	0.54	-5.3	12.4	-6.0	9.5	5.8	6.7
	7/20	91.3	3.71	0.14	3.83	0.17	-0.07	0.53	6.2	11.0	-6.8	8.7	90.6	2.5
	8/19	100.0	-0.14	1.05	0.08	0.31	-0.14	0.45	10.7	10.3	-19.7	7.7	-1.8	6.8
	9/18	100.0	0.26	0.26	0.05	0.12	0.05	0.35	2.3	8.7	-19.3	6.6	-0.8	4.7
	5/22	100.0	-0.19	0.26	0.05	0.12	0.06	0.41	-3.2	8.4	-13.2	6.9	-0.7	4.8
	6/21	100.0	-0.15	0.28	0.01	0.11	-0.02	0.47	0.7	8.5	-4.3	7.9	3.4	4.3
5	7/20	98.6	0.75	0.25	2.61	0.29	-0.54	0.60	-23.2	10.2	-24.0	8.9	112.9	4.5
	8/19	99.4	0.33	0.32	0.10	0.20	-0.10	0.45	25.8	8.9	-32.9	10.2	-2.9	7.3
	9/18	100.0	0.19	0.25	0.02	0.12	-0.01	0.37	7.5	9.6	-22.2	7.3	-1.3	4.9
	5/22	100.0	-0.17	0.26	0.04	0.12	-0.04	0.39	-3.6	9.6	-17.9	7.5	-0.6	5.3
	6/21	100.0	-0.12	0.47	0.01	0.16	-0.16	0.40	-9.9	10.7	-10.3	8.6	3.2	6.1
6	7/20	95.5	0.00	0.19	4.57	0.29	-0.55	0.64	9.4	10.1	-19.5	10.4	56.3	4.3
	8/19	100.0	0.31	0.30	0.04	0.13	0.07	0.44	5.8	11.0	-22.6	8.2	-0.2	6.3
	9/18	100.0	0.24	0.25	0.05	0.12	0.03	0.38	-3.3	8.9	-19.3	6.6	-0.5	4.9
	5/21	8.2	1.04	1.91	0.47	0.69	-1.57	0.59	42.0	12.1	-12.4	16.9	11.9	7.4
	5/22	89.4	-0.16	0.27	0.08	0.23	-0.03	0.42	3.4	14.8	-11.8	12.6	1.3	10.3
	6/21	80.5	-0.07	0.31	0.02	0.14	-0.06	0.60	0.5	13.0	-13.9	8.9	3.2	6.5
7	7/20	100.0	3.51	0.21	1.35	0.22	0.20	0.49	4.4	13.7	-4.3	11.7	154.7	2.9
	8/19	98.6	-1.39	2.17	0.54	0.76	-0.19	0.56	9.7	11.2	-16.4	8.2	-14.4	16.7
	9/18	100.0	0.22	0.28	0.05	0.12	0.04	0.36	8.1	10.1	-19.1	7.7	-2.8	5.2

Table SB24.4: *Simple* step parameters for selected states of the sG/aC base pair. IDs corresponds to the free energy minima shown in Figure SB24.1. Residue numbering in base pairs and steps A1-B1/A2-B2 are provided in Figure SA1. Abundances (*abu*), average values ($\langle X \rangle$) and standard deviations of samples ($s(X)$) are calculated by 3DNA for Shift (D_x), Slide (D_y), Rise (D_z), Tilt (τ), Roll (ρ), Twist (ω) employing the standard reference frames for the nucleobases. Only five central base pairs were included in the analysis for each state. Steps including a mismatch are highlighted in gray.

ID	A1-B1	abu	$\langle D_x \rangle$	$s(D_x)$	$\langle D_y \rangle$	$s(D_y)$	$\langle D_z \rangle$	$s(D_z)$	$\langle \tau \rangle$	$s(\tau)$	$\langle \rho \rangle$	$s(\rho)$	$\langle \omega \rangle$	$s(\omega)$
	A2-B2	[%]			[Å]							[°]		
	5-6/21-22	100.0	0.98	0.52	0.06	0.41	3.30	0.27	3.5	4.0	-0.2	5.5	38.8	3.9
1	6-7/20-21	100.0	-1.11	0.44	-2.08	0.85	3.10	0.29	1.5	5.9	0.4	4.1	-147.8	5.9
	7-8/19-20	99.7	-0.27	0.44	-1.35	0.64	3.19	0.29	5.2	5.7	-3.1	5.3	-148.8	4.8
	8-9/18-19	99.7	-0.29	0.51	-0.37	0.48	3.52	0.29	-1.7	4.2	5.1	6.0	36.1	4.5
	5-6/21-22	100.0	0.46	0.63	-0.47	0.57	3.47	0.30	2.8	4.5	1.7	6.0	37.1	4.9
	6-7/20-21	99.9	-1.40	0.71	-0.82	0.58	3.50	0.35	-6.4	5.6	0.7	4.2	-148.9	4.1
2	6-8/19-21	0.1	1.77	0.00	1.15	0.00	6.30	0.00	8.4	0.0	12.9	0.0	61.0	0.0
	7-8/19-20	99.9	-1.15	0.69	-0.41	0.75	3.02	0.38	3.9	6.7	-2.0	6.1	-145.9	6.5
	8-9/18-19	100.0	-0.77	0.61	-0.20	0.59	3.34	0.27	-2.9	4.2	-0.8	5.5	35.4	6.1
	5-6/21-22	100.0	0.81	0.60	-0.02	0.52	3.39	0.30	4.8	4.2	2.7	5.6	37.5	4.9
	6-7/20-21	88.0	-3.23	0.63	0.62	0.56	3.65	0.45	-6.5	5.9	0.1	6.4	-155.4	5.7
3	6-8/19-21	11.6	0.15	1.09	-0.99	0.98	6.52	0.46	0.4	6.2	15.6	10.7	58.6	6.0
	7-8/19-20	87.7	-3.11	0.63	1.44	1.35	2.80	0.58	15.7	9.3	-1.6	6.5	-144.4	9.6
	8-9/18-19	100.0	-0.77	0.68	-0.25	0.56	3.35	0.30	-2.8	4.3	-0.5	6.1	34.5	5.3
	5-6/20-21	4.1	0.85	0.41	0.50	0.80	3.51	0.31	0.0	4.0	19.8	7.2	32.2	2.6
	5-6/20-22	3.7	2.25	0.60	0.23	0.43	6.63	0.27	-22.2	6.4	17.8	4.4	45.5	4.1
	5-6/21-22	91.3	0.98	0.69	-0.15	0.57	3.44	0.30	1.8	4.2	-0.1	5.4	33.4	6.5
4	6-7/20-21	91.3	1.35	0.82	0.02	0.80	2.81	0.51	-18.9	8.1	-1.8	7.0	-133.5	7.1
	6-8/19-7	0.5	-1.79	0.00	-1.55	0.00	3.34	0.00	8.4	0.0	5.8	0.0	95.4	0.0
	6-8/19-20	8.3	-4.09	0.65	1.72	0.61	4.43	0.61	9.4	3.8	4.9	5.7	58.5	5.8
	7-8/19-20	91.3	1.78	0.53	1.46	0.52	3.44	0.33	2.0	4.5	-1.3	5.0	-164.1	3.7
	8-9/18-19	100.0	-0.18	0.59	-0.34	0.49	3.51	0.28	-3.4	4.0	2.6	5.7	35.0	4.4
	5-6/21-22	100.0	1.33	0.43	0.33	0.35	3.26	0.26	3.4	4.0	2.4	4.8	35.2	2.9
	6-7/20-21	98.6	-0.92	0.36	-1.44	0.40	2.60	0.32	0.7	5.1	-6.1	5.9	-139.5	3.9
5	6-8/19-21	1.4	-2.35	0.74	0.98	1.18	6.03	0.31	-4.9	5.9	4.3	4.5	64.5	3.3
	7-8/19-20	98.0	0.04	0.40	0.60	0.49	3.51	0.32	3.7	4.5	-4.0	4.7	-154.1	3.4
	7-9/18-20	0.3	-0.58	0.09	0.99	0.14	7.61	0.11	8.4	6.8	-6.6	1.9	-116.7	0.8
	8-9/18-19	99.4	0.03	0.48	-0.39	0.46	3.74	0.35	-0.8	4.6	9.9	6.3	37.1	3.9
	5-6/21-22	100.0	0.96	0.64	-0.06	0.50	3.41	0.28	4.0	4.0	0.6	5.9	37.3	4.0
	6-7/20-21	95.5	-0.21	0.68	2.10	0.50	2.54	0.32	-18.4	6.2	2.1	5.5	-144.2	5.8
6	6-8/19-21	4.3	-0.02	0.81	-2.11	1.02	6.26	0.51	-0.1	8.3	16.7	8.9	54.6	7.4
	7-8/19-20	95.5	0.21	0.45	2.93	0.51	3.10	0.29	6.3	4.8	5.2	4.7	-160.9	3.9
	8-9/18-19	100.0	-0.55	0.47	-0.39	0.46	3.42	0.31	-2.5	4.5	2.2	5.7	37.8	3.8
	5-6/21-22	80.5	0.47	0.67	-0.60	0.59	3.35	0.33	0.7	4.4	2.2	5.7	32.1	5.1
	6-7/20-21	80.5	0.50	0.76	-2.33	0.98	3.47	0.45	-7.4	7.9	0.1	6.9	-132.5	5.8
7	7-8/19-20	98.6	-0.20	0.70	-0.62	0.98	3.32	0.37	4.0	6.2	-2.2	5.4	-160.7	5.2
	7-9/18-20	1.4	-0.59	0.15	-1.42	1.10	6.61	0.20	9.6	5.7	1.2	5.7	-126.2	10.2
	8-9/18-19	98.6	0.56	0.88	-0.56	0.51	3.48	0.33	-1.8	4.4	4.2	6.0	33.0	5.6

4.5 sG/aT

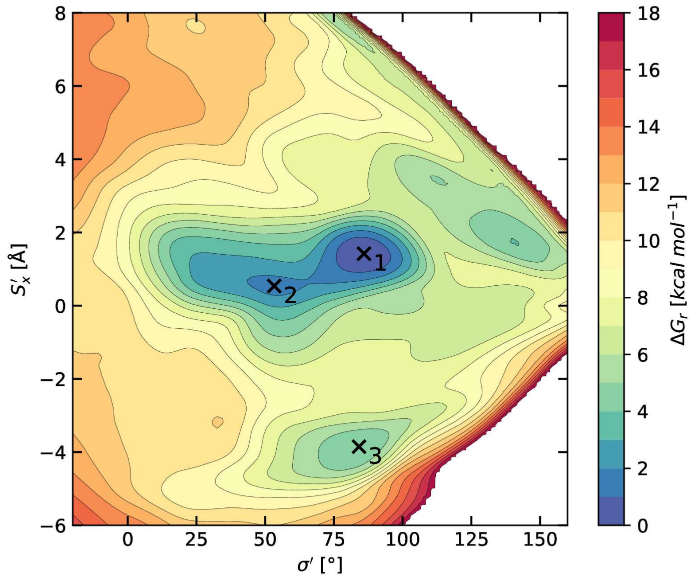

Figure SB25.1: Free energy surface for the sG/aT base pair. Labeled crosses show position of selected free energy minima (thermodynamic states). Free energy isolines are spaced by 1 kcal mol^{-1}.

Table SB25.1: Positions of selected free minima on the free energy surface for the sG/aT base pair. IDs correspond to the selected free energy minima shown in Figure SB25.1. Confidence interval of the free energy ΔG_r is provided at three standard deviations.

ID	σ' [°]	S'_x [Å]	ΔG_r [$kcal\ mol^{-1}$]
1	86.0	1.42	0.00±0.00
2	53.3	0.54	1.66±0.12
3	84.2	-3.85	4.08±0.15

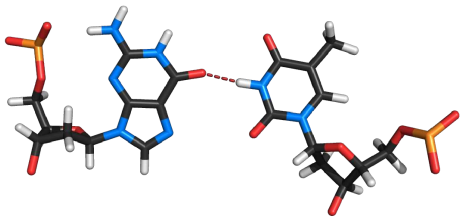

ID 1: $\sigma' = 86.0°$, $S'_x = 1.42$ Å

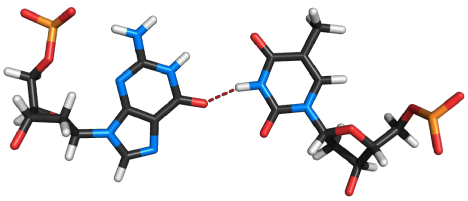

ID 2: $\sigma' = 53.3°$, $S'_x = 0.54$ Å

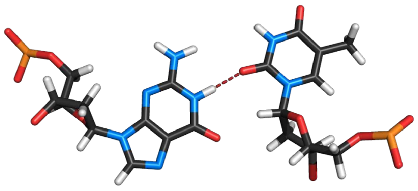

ID 3: $\sigma' = 84.2°$, $S'_x = -3.85$ Å

Figure SB25.2: Average geometries representing selected free energy minima for the sG/aT base pair. IDs correspond to the selected free energy minima shown in Figure SB25.1. Major and minor grooves are top and bottom, respectively. View direction is along the z-axis of DNA.

Table SB25.2: Number of analysed snapshots (N_{snap}), abundances (abu), average hydrogen bond distances and angles from hydrogen bond analysis provided by cpptraj for selected states of the sG/aT base pair. IDs correspond to the selected free energy minima shown in Figure SB25.1. Residue and atom numbering are provided in Figure SA1.

ID	N_{snap}	acceptor	H-donor	donor	abu [%]	d_{avg} [Å]	a_{avg} [°]
1	242	G7@O6	T20@H3	T20@N3	90.1	2.8	165.1
		G7@OP2	G7@H22	G7@N2	50.4	2.9	165.6
2	236	G7@O6	T20@H3	T20@N3	73.3	2.9	152.6
		G7@OP2	G7@H22	G7@N2	35.6	2.9	165.3
3	523	T20@O2	G7@H1	G7@N1	93.5	2.8	165.2

Table SB25.3: *Simple* base-pair parameters for selected states of the sG/aT base pair. IDs corresponds to the free energy minima shown in Figure SB25.1. Residue numbering in base pairs A/B are provided in Figure SA1. Abundances (*abu*), average values ($\langle X \rangle$) and standard deviations of samples ($s(X)$) are calculated by 3DNA for Shear (S_x), Stretch (S_y), Stagger (S_z), Buckle (κ), Propeller (π), Opening (σ) employing the standard reference frames for the nucleobases. Only five central base pairs were included in the analysis for each state. Base pairs with a mismatch are highlighted in gray.

ID	A/B	abu [%]	$\langle S_x \rangle$	$s(S_x)$	$\langle S_y \rangle$ [Å]	$s(S_y)$	$\langle S_z \rangle$	$s(S_z)$	$\langle \kappa \rangle$	$s(\kappa)$	$\langle \pi \rangle$	$s(\pi)$ [°]	$\langle \sigma \rangle$	$s(\sigma)$
	5/22	100.0	-0.14	0.25	0.02	0.11	-0.05	0.39	-5.9	7.9	-17.6	6.3	0.5	4.5
	6/21	100.0	-0.05	0.26	0.06	0.16	0.15	0.43	-8.9	8.4	-8.5	6.4	8.3	5.0
1	7/20	100.0	-1.42	0.13	1.28	0.16	-0.49	0.52	-2.9	8.9	-16.1	7.0	94.2	2.5
	8/19	100.0	0.10	0.26	-0.01	0.11	-0.06	0.44	10.3	10.0	-18.4	7.2	0.4	4.8
	9/18	100.0	0.14	0.27	0.01	0.12	-0.01	0.41	0.3	8.7	-18.1	7.7	-0.2	5.1
	5/22	100.0	-0.18	0.24	0.05	0.12	0.05	0.41	-4.2	8.0	-12.5	7.8	-0.5	4.6
	6/21	100.0	-0.07	0.24	0.02	0.14	-0.02	0.45	-3.5	9.3	-4.0	7.4	6.7	4.5
2	7/20	100.0	-0.53	0.12	0.75	0.21	0.04	0.45	-8.2	9.7	-15.1	8.6	126.3	4.3
	8/19	100.0	0.20	0.30	0.09	0.15	0.04	0.41	21.7	8.9	-19.9	8.6	-4.1	6.7
	9/18	100.0	0.14	0.26	0.02	0.12	0.05	0.44	10.7	9.4	-22.9	8.2	-1.3	5.1
	5/22	100.0	-0.18	0.26	0.04	0.11	-0.02	0.40	-3.4	9.1	-18.0	8.1	-0.5	5.2
	6/21	100.0	-0.08	0.28	0.03	0.14	0.04	0.49	-7.5	11.3	-9.4	9.2	4.6	6.0
3	7/20	99.8	3.87	0.16	3.63	0.20	0.04	0.52	7.0	11.6	-6.5	8.8	96.1	3.2
	8/19	100.0	-0.39	1.44	0.15	0.44	-0.13	0.46	12.2	11.0	-18.6	9.0	-3.0	9.8
	9/18	100.0	0.19	0.28	0.02	0.13	0.01	0.42	4.5	9.8	-19.6	8.0	-0.7	5.3

Table SB25.4: *Simple* step parameters for selected states of the sG/aT base pair. IDs corresponds to the free energy minima shown in Figure SB25.1. Residue numbering in base pairs and steps A1-B1/A2-B2 are provided in Figure SA1. Abundances (*abu*), average values ($\langle X \rangle$) and standard deviations of samples ($s(X)$) are calculated by 3DNA for Shift (D_x), Slide (D_y), Rise (D_z), Tilt (τ), Roll (ρ), Twist (ω) employing the standard reference frames for the nucleobases. Only five central base pairs were included in the analysis for each state. Steps including a mismatch are highlighted in gray.

ID	A1-B1 A2-B2	abu [%]	$\langle D_x \rangle$	$s(D_x)$	$\langle D_y \rangle$ [Å]	$s(D_y)$	$\langle D_z \rangle$	$s(D_z)$	$\langle \tau \rangle$	$s(\tau)$	$\langle \rho \rangle$	$s(\rho)$ [°]	$\langle \omega \rangle$	$s(\omega)$
	5-6/21-22	100.0	1.61	0.38	0.37	0.34	3.36	0.28	2.7	3.9	3.2	4.6	36.0	2.6
1	6-7/20-21	100.0	-1.59	0.41	-0.78	0.35	3.03	0.26	5.4	5.2	-4.2	5.0	-133.9	3.4
	7-8/19-20	100.0	-0.62	0.44	1.53	0.40	3.22	0.27	-0.1	4.7	-0.7	4.1	-153.0	3.5
	8-9/18-19	100.0	-0.41	0.56	-0.38	0.54	3.48	0.32	-3.5	4.2	3.7	5.3	37.8	3.9
	5-6/21-22	100.0	1.60	0.36	0.51	0.33	3.31	0.27	4.1	4.2	3.4	4.9	35.7	2.7
2	6-7/20-21	100.0	-1.67	0.39	-1.95	0.47	2.99	0.29	2.8	5.8	-3.4	5.0	-132.9	3.2
	7-8/19-20	100.0	-0.07	0.44	0.29	0.45	3.22	0.27	-0.2	4.6	-0.6	4.1	-154.5	3.3
	8-9/18-19	100.0	-0.07	0.55	-0.23	0.52	3.55	0.33	-2.0	4.3	6.4	6.1	37.3	4.0
	5-6/21-22	100.0	1.03	0.61	-0.09	0.56	3.43	0.29	2.2	4.1	1.0	5.5	35.5	4.5
	6-7/20-21	99.8	1.27	0.59	-0.19	0.81	2.72	0.44	-20.5	7.0	-2.6	6.5	-135.2	5.2
3	6-8/19-21	0.2	0.30	0.00	-3.11	0.00	6.52	0.00	9.7	0.0	10.4	0.0	56.3	0.0
	7-8/19-20	99.8	1.63	0.56	1.38	0.66	3.34	0.32	2.2	4.5	-0.5	4.6	-164.1	3.9
	8-9/18-19	100.0	-0.16	0.67	-0.31	0.51	3.53	0.37	-3.1	4.0	4.6	6.0	34.6	4.7

4.6 sC/aT

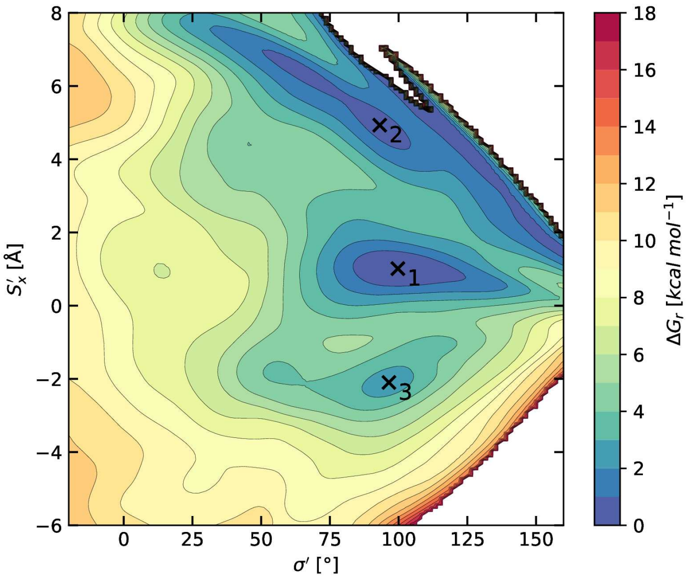

Figure SB26.1: Free energy surface for the sC/aT base pair. Labeled crosses show position of selected free energy minima (thermodynamic states). Free energy isolines are spaced by 1 kcal mol^{-1}.

Table SB26.1: Positions of selected free minima on the free energy surface for the sC/aT base pair. IDs correspond to the selected free energy minima shown in Figure SB26.1. Confidence interval of the free energy ΔG_r is provided at three standard deviations.

ID	σ' [°]	S'_x [Å]	ΔG_r [$kcal\ mol^{-1}$]
1	99.7	1.01	0.00±0.00
2	93.1	4.93	0.44±0.10
3	96.5	-2.10	2.58±0.09

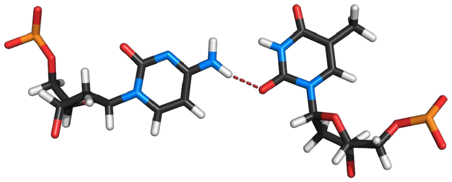

ID 1: $\sigma' = 99.7°$, $S'_x = 1.01$ Å

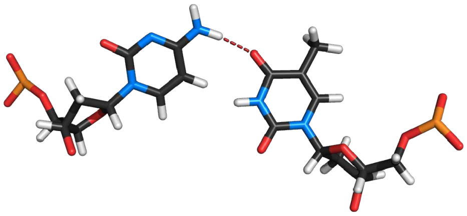

ID 2: $\sigma' = 93.1°$, $S'_x = 4.93$ Å

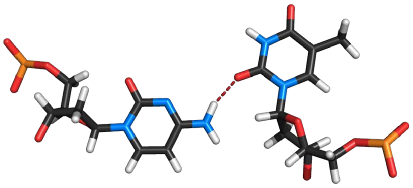

ID 3: $\sigma' = 96.5°$, $S'_x =$ -2.10 Å

Figure SB26.2: Average geometries representing selected free energy minima for the sC/aT base pair. IDs correspond to the selected free energy minima shown in Figure SB26.1. Major and minor grooves are top and bottom, respectively. View direction is along the z-axis of DNA.

Table SB26.2: Number of analysed snapshots (N_{snap}), abundances (abu), average hydrogen bond distances and angles from hydrogen bond analysis provided by cpptraj for selected states of the sC/aT base pair. IDs correspond to the selected free energy minima shown in Figure SB26.1. Residue and atom numbering are provided in Figure SA1.

ID	N_{snap}	acceptor	H-donor	donor	abu [%]	d_{avg} [Å]	a_{avg} [°]
1	381	T20@O2	C7@H42	C7@N4	71.4	2.9	158.1
2	386	T20@O4	C7@H42	C7@N4	56.5	2.9	163.3
3	319	T20@O2	C7@H41	C7@N4	72.1	2.8	154.7

Table SB26.3: *Simple* base-pair parameters for selected states of the sC/aT base pair. IDs corresponds to the free energy minima shown in Figure SB26.1. Residue numbering in base pairs A/B are provided in Figure SA1. Abundances (*abu*), average values ($\langle X \rangle$) and standard deviations of samples ($s(X)$) are calculated by 3DNA for Shear (S_x), Stretch (S_y), Stagger (S_z), Buckle (κ), Propeller (π), Opening (σ) employing the standard reference frames for the nucleobases. Only five central base pairs were included in the analysis for each state. Base pairs with a mismatch are highlighted in gray.

ID	A/B	abu [%]	$\langle S_x \rangle$	$s(S_x)$	$\langle S_y \rangle$ [Å]	$s(S_y)$	$\langle S_z \rangle$	$s(S_z)$	$\langle \kappa \rangle$	$s(\kappa)$	$\langle \pi \rangle$ [°]	$s(\pi)$	$\langle \sigma \rangle$	$s(\sigma)$
	5/22	99.7	-0.19	0.27	0.04	0.13	0.08	0.37	-2.0	9.6	-17.6	7.0	-0.7	5.2
	6/21	100.0	-0.12	0.31	0.01	0.16	0.08	0.47	-7.7	10.1	-13.8	7.7	3.0	7.1
1	7/20	100.0	-1.00	0.13	2.41	0.32	-0.55	0.70	-0.5	9.8	-14.3	10.0	80.3	5.0
	8/19	100.0	0.14	0.27	0.02	0.13	0.01	0.40	8.0	9.7	-19.4	8.2	1.2	5.4
	9/18	100.0	0.18	0.26	0.02	0.12	0.03	0.39	-1.3	8.6	-18.5	6.8	0.3	4.6
	5/22	100.0	-0.18	0.26	0.03	0.12	-0.10	0.38	2.9	8.7	-17.2	7.6	-0.1	5.3
	6/21	100.0	-0.15	0.27	-0.00	0.13	-0.37	0.40	-3.8	11.8	-16.6	7.5	2.6	5.6
2	7/20	99.5	-4.92	0.26	2.55	0.23	0.02	0.69	-4.0	12.6	-20.2	10.3	86.6	3.9
	8/19	99.7	0.06	0.26	0.01	0.12	-0.30	0.40	2.4	10.1	-9.5	7.4	-0.8	5.0
	8/20	0.3	-0.25	0.00	-0.51	0.00	1.58	0.00	2.7	0.0	-11.4	0.0	-9.2	0.0
	9/18	100.0	0.10	0.26	0.02	0.12	-0.03	0.39	-0.1	8.8	-14.5	7.3	1.1	5.2
	5/22	98.8	-0.13	0.26	0.03	0.12	-0.07	0.40	0.1	10.1	-12.5	10.3	0.9	5.2
	6/21	98.1	1.11	2.68	0.42	0.86	-0.22	0.67	-1.6	13.8	-8.3	12.4	5.6	12.0
3	7/20	100.0	2.09	0.16	2.92	0.30	0.02	0.79	1.9	11.6	-3.7	13.8	83.5	3.9
	8/19	100.0	0.07	0.28	0.01	0.13	-0.09	0.42	9.8	9.9	-13.9	10.5	-0.7	5.4
	9/18	100.0	0.22	0.28	0.04	0.11	0.03	0.40	1.1	9.3	-19.2	7.1	0.3	5.1

Table SB26.4: *Simple* step parameters for selected states of the sC/aT base pair. IDs corresponds to the free energy minima shown in Figure SB26.1. Residue numbering in base pairs and steps A1-B1/A2-B2 are provided in Figure SA1. Abundances (*abu*), average values ($\langle X \rangle$) and standard deviations of samples ($s(X)$) are calculated by 3DNA for Shift (D_x), Slide (D_y), Rise (D_z), Tilt (τ), Roll (ρ), Twist (ω) employing the standard reference frames for the nucleobases. Only five central base pairs were included in the analysis for each state. Steps including a mismatch are highlighted in gray.

ID	A1-B1 A2-B2	abu [%]	$\langle D_x \rangle$	$s(D_x)$	$\langle D_y \rangle$ [Å]	$s(D_y)$	$\langle D_z \rangle$	$s(D_z)$	$\langle \tau \rangle$	$s(\tau)$	$\langle \rho \rangle$	$s(\rho)$ [°]	$\langle \omega \rangle$	$s(\omega)$
	5-6/21-22	99.7	0.78	0.64	-0.24	0.56	3.41	0.29	3.0	4.0	-0.4	5.5	37.1	4.4
1	6-7/20-21	100.0	-0.91	0.56	1.40	0.57	2.78	0.35	-14.9	8.6	-1.1	5.7	-145.7	7.1
	7-8/19-20	100.0	-0.76	0.39	2.25	0.49	3.24	0.29	0.7	4.8	0.3	4.4	-155.7	4.7
	8-9/18-19	100.0	-0.47	0.51	-0.43	0.48	3.46	0.30	-2.8	4.0	2.1	5.3	38.1	4.0
	5-6/21-22	100.0	0.44	0.67	-0.19	0.54	3.38	0.27	4.5	4.3	3.4	5.2	37.4	4.4
	6-7/20-21	99.5	-2.73	0.59	1.18	0.73	3.64	0.38	-3.6	5.6	4.7	6.0	-150.8	5.4
	6-8/19-21	0.3	0.29	0.00	-0.36	0.00	5.55	0.00	7.4	0.0	-6.4	0.0	68.6	0.0
2	6-8/20-21	0.3	-1.04	0.00	0.69	0.00	4.87	0.00	14.1	0.0	14.2	0.0	47.9	0.0
	7-8/19-20	99.5	-1.53	0.46	2.37	0.58	2.73	0.33	6.9	6.7	5.5	4.8	-140.6	5.7
	8-9/18-19	99.7	-0.18	0.53	-0.68	0.52	3.40	0.32	-3.0	4.0	-0.9	5.1	34.6	4.0
	8-9/18-20	0.3	1.41	0.00	-1.86	0.00	4.55	0.00	-18.5	0.0	8.7	0.0	54.9	0.0
	5-6/21-22	96.9	0.76	0.70	-0.15	0.72	3.39	0.31	2.5	4.8	1.6	5.6	29.3	9.6
	5-7/20-22	0.6	3.70	0.34	0.95	0.70	5.16	0.58	-26.1	9.7	-4.9	3.9	-105.2	6.5
3	6-7/20-21	90.9	1.21	1.05	1.02	0.99	3.17	1.03	-14.4	15.4	-2.3	7.4	-133.9	10.7
	7-8/19-20	100.0	0.99	0.55	2.28	0.63	3.45	0.32	-0.0	5.1	-0.6	4.7	-163.0	27.4
	8-9/18-19	100.0	-0.57	0.56	-0.18	0.49	3.49	0.31	-4.1	4.2	1.4	5.5	37.0	3.5
